# Synthesis of Silylated Cyclobutanone and Cyclobutene Derivatives Involving 1,4‐Addition of Zinc‐Based Silicon Nucleophiles

**DOI:** 10.1002/chem.202102993

**Published:** 2021-10-07

**Authors:** Ming Cui, Martin Oestreich

**Affiliations:** ^1^ Institut für Chemie Technische Universität Berlin Strasse des 17. Juni 115 10623 Berlin Germany

**Keywords:** conjugate addition, copper, silicon, synthetic methods, zinc

## Abstract

A copper‐catalyzed conjugate silylation of various cyclobutenone derivatives with Me_2_PhSiZnCl ⋅ 2LiCl or (Me_2_PhSi)_2_Zn ⋅ *x*LiCl (*x*≤4) to generate β‐silylated cyclobutanones is reported. Trapping the intermediate enolate with ClP(O)(OPh)_2_ affords silylated enol phosphates that can be further engaged in Kumada cross‐coupling reactions to yield silylated cyclobutene derivatives.

## Graphical abstract

**A series of highly** substituted cyclobutenones undergoes 1,4‐addition with zinc‐based nucleophiles with and, for non‐hindered acceptors, without the aid of a copper catalyst. The intermediate metal enolate can be trapped as an enol phosphate and further reacted with Grignard reagents in Kumada cross‐coupling reactions. By this, a range of silylated cyclobutanone and cyclobutene derivatives becomes accessible.

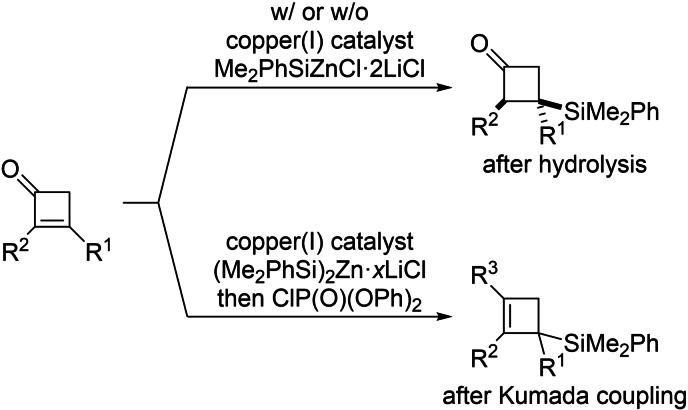

Conjugate addition of silicon nucleophiles to α,β‐unsaturated carbonyl compounds is one of the standard processes for the formation of C(sp^3^)−Si bonds.^[1]^ The resulting β‐silylated carbonyl compounds^[2]^ can be converted into the corresponding aldols by oxidative degradation of that C(sp^3^)−Si bond.^[3]^ As to cyclic acceptors, the vast majority of protocols are for cyclopentenone and ‐hexenone derivatives.[^4^, ^5^] Murakami and co‐workers reported the 1,4‐addition to cyclobutenone derivatives using Fleming's (Me_2_PhSi)_2_CuLi ⋅ LiCN[^4a^, ^4b^] to access functionalized 1,3‐dienes after trapping of the enolate intermediate and electrocyclic ring‐opening (Scheme 1, top).^[6]^ Aside from this isolated example, there are no further methods known, neither stoichiometric nor catalytic in copper.

**Scheme 1:**
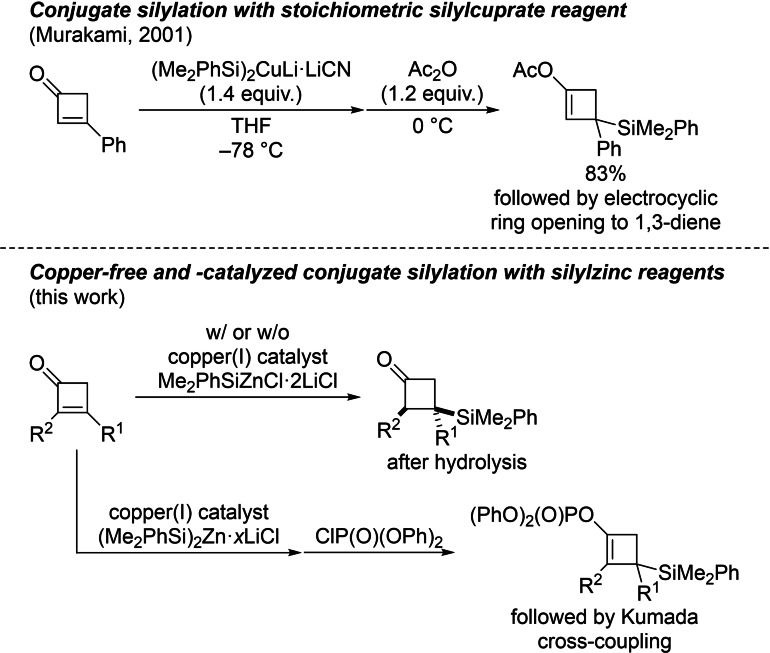
Conjugate silylation of cyclobutenone derivatives and follow‐up chemistry of the in situ‐formed enolates.

Almost 20 years ago, our laboratory introduced copper‐catalyzed and even copper‐free protocols for conjugate silylation employing bis(triorganosilyl)zinc and tris(triorganosilyl)zincate reagents.[^7^, ^8^, ^9^] We also found copper salts to accelerate these reactions and to be essential for hindered and β,β‐disubstituted acceptors, respectively.^[8]^ Zinc‐based silicon nucleophiles such as (Me_2_PhSi)_2_Zn ⋅ 4LiCl and also Me_2_PhSiZnCl ⋅ 2LiCl are in fact highly useful. Their functional‐group tolerance is substantially improved over that of the corresponding more reactive lithium compounds from which the zinc reagents are typically prepared by transmetalation. To date, none of these protocols have been applied to cyclobutenones. Moreover, the synthesis of cyclobutyl‐substituted silanes is limited to a few examples. In 2010, Ito and co‐workers reported a copper‐catalyzed borylation of silyl‐substituted homoallylic sulfonates, and cyclobutylsilane derivatives were obtained by insertion of the C−C double bond into an in situ formed Cu−B bond followed by an intramolecular S_N_2 reaction.^[10]^ The Fu group^[11]^ and our group^[12]^ reported single examples of the synthesis of cyclobutylsilanes by metal‐catalyzed radical cross‐coupling of a tertiary and a secondary cyclobutyl bromide with zinc‐ and magnesium‐based silicon reagents, respectively. In this work, we describe copper‐catalyzed conjugate silylations of highly substituted cyclobutenone derivatives with zinc‐based silicon reagents (Scheme 1, bottom). The intermediate metal enolates can either be hydrolyzed to afford 3‐silyl‐substituted cyclobutanones or captured with ClP(O)(OPh)_2_ as an electrophile to furnish cyclobutenyl phosphates. Subsequent Kumada cross‐coupling yields silicon‐containing cyclobutene derivatives.

Our study commenced with the conjugate silylation of cyclobutenone **1** **a** with 2.0 equiv. of Me_2_PhSiZnCl ⋅ 2LiCl in THF^[13]^ (Table 1). Using Cu(CH_3_CN)_4_PF_6_ as the catalyst in THF at room temperature, β‐silylated β‐phenylcyclobutanone **2** **a** was obtained in 95 % yield after hydrolysis (entry 1). Yields were slightly lower with less silicon nucleophile, for example 91 % yield with 1.5 equiv. of Me_2_PhSiZnCl ⋅ 2LiCl. Given the possibility of a copper‐free 1,4‐addition,^[8]^ we compared different β‐substituted and α,β‐disubstituted cyclobutenones in reactions with and without the copper catalyst. The silylation of **1** **a** in the absence of Cu(CH_3_CN)_4_PF_6_ did lead to **2** **a** yet with a substantial decrease in yield (entry 1). Other cyclobutenones such as β‐butyl‐substituted **1** **g** and α,β‐disubstituted **1** **o** and **1** **p** were tested, and the low yields of the copper‐free protocol confirmed the importance of a copper catalyst (entries 2–4).

**Table 1:** Comparison of copper‐catalyzed and copper‐free protocols with Me_2_PhSiZnCl ⋅ 2LiCl.^[a]^

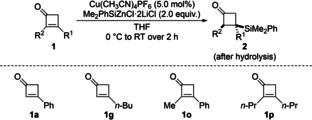				
Entry	Acceptor	Product	Yield of **2** [%]^[b]^ w/ Cu(CH_3_CN)_4_PF_6_	Yield of **2** [%]^[c]^ w/ o Cu(CH_3_CN)_4_PF_6_
1	**1** **a**	**2** **a**	95	71
2	**1** **g**	**2** **g**	quant.	24
3	**1** **o**	**2** **o**	95	30
4	**1** **p**	**2** **p**	95	0

[a] All reactions were performed on a 0.2 mmol scale for 2 h. [b] Isolated yield after flash chromatography on silica gel. [c] Determined by ^1^H NMR spectroscopy by using CH_2_Br_2_ as the internal standard.

We further tested the substrate scope of this conjugate silylation (Scheme 2). β‐Aryl‐substituted cyclobutenones were generally suitable substrates, affording the corresponding β‐silylated cyclobutanones in good to excellent yields (**1** **a**–**f→2** **a**–**f**). Electron‐donating groups at the aryl ring such as methyl and methoxy led to higher yields than halogenated derivatives. Likewise, cyclobutenones bearing a primary alkyl substituent in the β‐position furnished the corresponding products in equally high yields (**1** **g**–**k→2** **g**–**k**); the yield was lowest for **1** **k** containing a C(sp^3^)−Cl bond. With sterically more demanding secondary alkyl groups such as cyclopropyl and cyclohexyl, yields were still good (**1** **l**,**m→2** **l**,**m**). A silyl group in the β‐position was also compatible (**1** **n→2** **n**). The reactions of α,β‐disubstituted cyclobutenones **1** **o** and **1** **p** proceeded equally well. Product **2** **o** was obtained with high diastereoselectivity while **2** **p** formed with a poor diastereomeric ratio. We believe that the diastereoselectivity is mainly controlled by steric factors in the protolysis of the enolate intermediate.

**Scheme 2:**
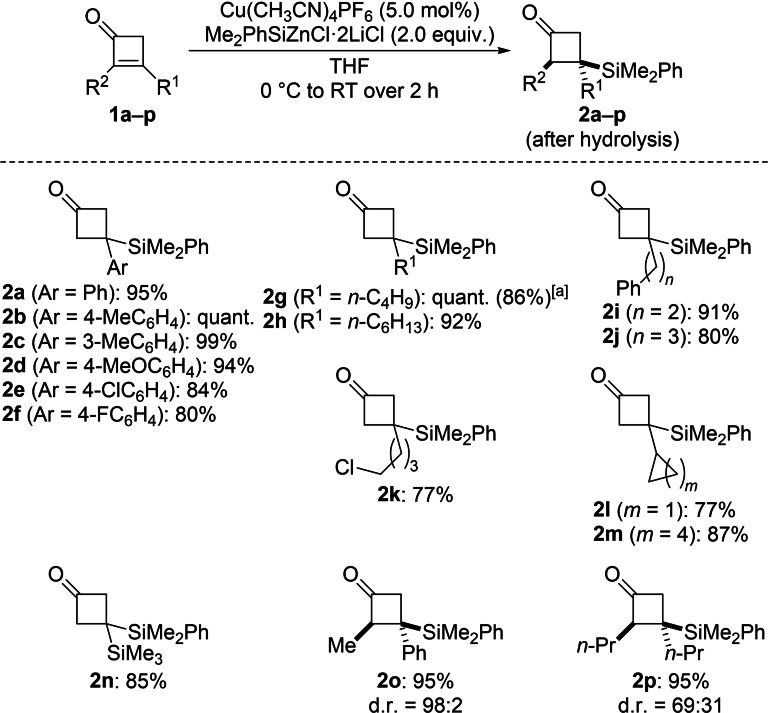
Synthesis of β‐silylated cyclobutanones by conjugate addition of Me_2_PhSiZnCl ⋅ 2LiCl. Unless otherwise noted, all reactions were performed on a 0.2 mmol scale for 2 h. Yields are of analytically pure product obtained after flash chromatography on silica gel. The relative configuration was assigned by ^1^H NMR spectroscopic analysis prior to purification (see the Supporting Information for details). [a] Value in parentheses for the reaction on a 1.0 mmol scale.

Next, we tried to capture the enolate intermediate as an enol phosphate,^[14]^ that is cyclobutenyl phosphates **3**, to allow for subsequent cross‐coupling reactions.^[15]^ The brief survey outlined in Table 2 shows that copper‐catalyzed 1,4‐addition of either Me_2_PhSiZnCl ⋅ 2LiCl or (Me_2_PhSi)_2_Zn ⋅ *x*LiCl (*x*≤4) to **1** **a** followed by enolate trapping with ClP(O)(OPh)_2_ furnishes the enol phosphate **3** **a** in moderate yields (entries 1 and 2). Relevant to an enantioselective variant, no uncatalyzed background reaction was seen with an almost salt‐free stock solution of (Me_2_PhSi)_2_Zn ⋅ *x*LiCl in Et_2_O^[16]^ (entry 2). In the light of our recent work about an enantioselective conjugate silylation with a zinc‐based silicon nucleophile,^[17]^ we decided to investigate the asymmetric version. The yield increased in the presence of the chiral phosphoramidite ligand (*S*,*R*,*R*)‐**L1** but enantioinduction was low, even at −78 °C (entries 3 and 4). A systematic screening of various chiral ligands was completely unsuccessful (see the Supporting Information for the details). However, the yield could be improved to 76 % with no enantioselectivity with (*R*,*R*,*R*)‐**L2** (see Scheme 3), and we continued using this ligand for the reaction scope (a racemic ligand such as *rac*‐binap afforded significantly lower yields; 19 % yield). For completion, the corresponding 1,4‐addition of Me_2_PhSiZnCl ⋅ 2LiCl in the presence of (*S*,*R*,*R*)‐**L1** proceeded with no enantioinduction.

**Table 2:** Comparison of copper‐catalyzed and copper‐free protocols with enolate trapping.^[a]^

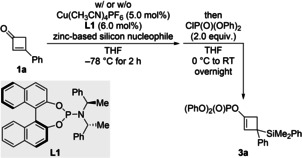			
Entry	Zinc‐based silicon nucleophile	Yield of **3** **a** [%]^[b]^ w/ Cu(CH_3_CN)_4_PF_6_	Yield of **3** **a** [%]^[b]^ w/ o Cu(CH_3_CN)_4_PF_6_
1	Me_2_PhSiZnCl ⋅ 2LiCl (2.0 equiv.)	56	18
2	(Me_2_PhSi)_2_Zn ⋅ *x*LiCl (1.2 equiv.)	36	0
3	(Me_2_PhSi)_2_Zn ⋅ *x*LiCl (1.2 equiv.)	53 (6 % *ee*)^[c]^ w/ **L1**	–
4^[d]^	(Me_2_PhSi)_2_Zn ⋅ *x*LiCl (1.2 equiv.)	55 (15 % *ee*)^[c]^ w/ **L1**	–

[a] All reactions were performed on a 0.2 mmol scale for 2 h. [b] Determined by ^1^H NMR spectroscopy by using CH_2_Br_2_ as the internal standard. [c] Determined by HPLC analysis on a chiral stationary phase. [d] The 1,4‐addition was conducted at −78 °C for 16 h prior to the addition of ClP(O)(OPh)_2_.

**Scheme 3:**
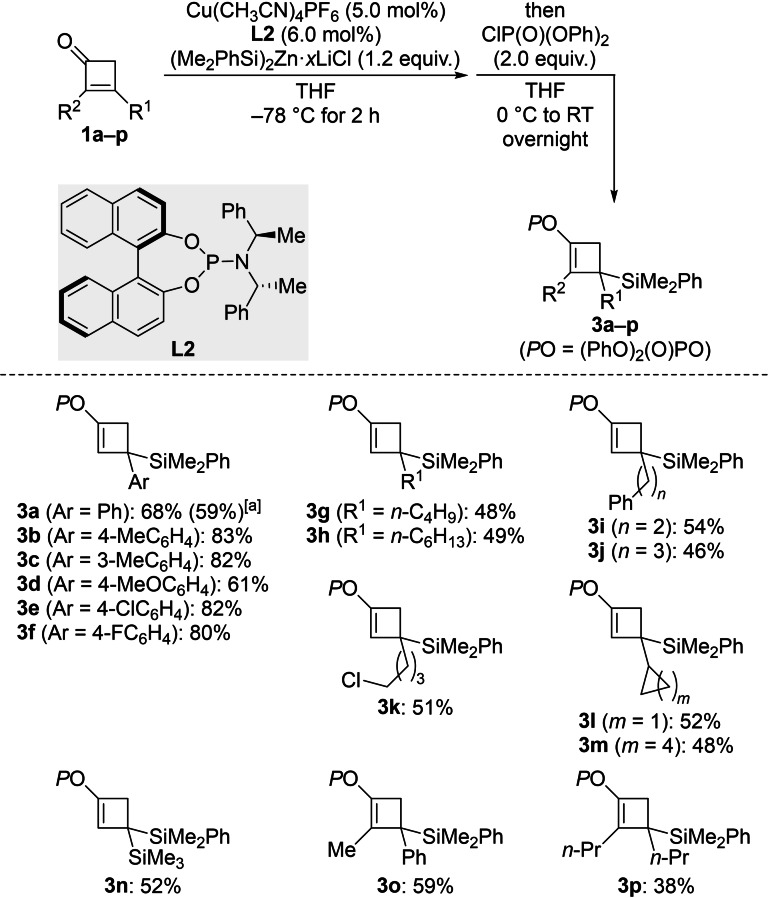
Synthesis of silylated cyclobutenyl phosphates by sequential conjugate addition of (Me_2_PhSi)_2_Zn ⋅ *x*LiCl (*x*≤4) and enolate trapping. Unless otherwise noted, all reactions were performed on a 0.2 mmol scale. Yields are of analytically pure product obtained after flash chromatography on silica gel. [a] Value in parentheses for the reaction on a 1.5 mmol scale.

The optimized reaction conditions are 5.0 mol% of Cu(CH_3_CN)_4_PF_6_ and 6.0 mol% of **L2** in THF with 1.2 equiv. of (Me_2_PhSi)_2_Zn ⋅ *x*LiCl as the silicon source and ClP(O)(OPh)_2_ as the electrophilic trapping reagent (Scheme 3). The reaction scope was done with the same set of cyclobutenones **1** **a**–**p** (cf. Scheme 2). Yields were good throughout with β‐aryl‐substituted cyclobutenones (**1** **a**–**f→3** **a**–**f**). Conversely, the β‐alkyl‐substituted derivatives were less reactive, and moderate yields were obtained (**1** **g**–**m→3** **g**–**m**). Again, a silyl group as in **1** **n** was tolerated to give **3** **n** in 52 % yield. Both α,β‐disubstituted substrates **1** **o** and **1** **p** did react in acceptable yields, affording fully substituted enol phosphates **3** **o** and **3** **p**, respectively.

Enol phosphates can serve as electrophiles in cross‐coupling reactions,^[15]^ and we tested several of the above cyclobutenyl phosphates in Kumada coupling reactions (**3→4**, Scheme 4). These representative reactions proceeded in moderate yields in the presence of catalytic amounts of (dppe)NiCl_2_.^[18]^ Arylation with PhMgBr reliably gave the corresponding silylated cyclobutenes. In turn, alkylation with the primary alkyl Grignard reagent *n*‐HexMgBr was low yielding but an acceptable yield was restored with secondary CyMgBr.

**Scheme 4:**
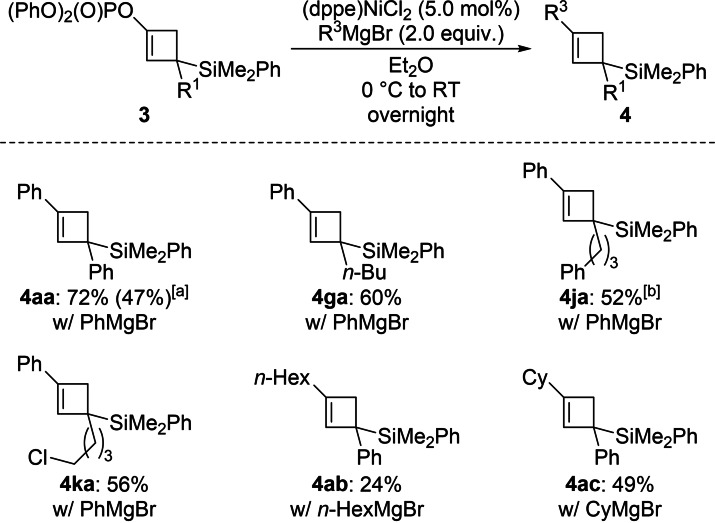
Nickel‐catalyzed Kumada cross‐coupling of silylated cyclobutenyl phosphates and Grignard reagents. Unless otherweise noted, all reactions were performed on a 0.10 mmol scale. Yields are of analytically pure product obtained after flash chromatography on silica gel. [a] Value in parentheses for the reaction on a 1.0 mmol scale. [b] Performed on a 0.065 mmol scale.

To summarize, we reported here a copper‐catalyzed conjugate addition of zinc‐based silicon reagents to highly substituted cyclobutenones, providing a general and efficient method to access various β‐silylated cyclobutanones. Moreover, the enolate intermediate can be trapped with a phosphorus electrophile to arrive at silylated enol phosphates, and these can be converted into the corresponding cyclobutenes by Kumada cross‐coupling.

## Conflict of interest

The authors declare no conflict of interest.

## Supporting information

As a service to our authors and readers, this journal provides supporting information supplied by the authors. Such materials are peer reviewed and may be re‐organized for online delivery, but are not copy‐edited or typeset. Technical support issues arising from supporting information (other than missing files) should be addressed to the authors.

Supporting Information
