# Supplementary material for: Synthesis of Silylated Cyclobutanone and Cyclobutene Derivatives Involving 1,4‐Addition of Zinc‐Based Silicon Nucleophiles
Source: Chemistry. 2021 Oct 7;27(65):16103–6. doi: 10.1002/chem.202102993 (PMC9292915; doi:10.1002/chem.202102993)
Supplement: Supplementary file 1 — Supporting Information [file CHEM-27-16103-s001.pdf]

Chemistry–A European Journal

Supporting Information

Synthesis of Silylated Cyclobutanone and Cyclobutene Derivatives Involving 1,4-Addition of Zinc-Based Silicon Nucleophiles

Ming Cui and Martin Oestreich*

Table of Contents

1. General Information **2**

2. General Procedures **3**

3. Optimization Study **5**

4. General Procedures for Conjugate Silyl Additions to Cyclobutenones (GP1) **6**

5. General Procedures for Conjugate Silylation/Trapping Reactions (GP2) **6**

6. General Procedures for Kumada Coupling of Enol Phosphates (GP3) **7**

7. Experimental Details for Conjugate Silylation of Cyclobutenones **8**

8. Experimental Details for Conjugate Silylation/Trapping Reactions **16**

9. Experimental Details for Kumada Coupling of Cyclobutenyl Phosphates **26**

10. 1.0 mmol Scale Reactions **30**

11. NMR Spectra **32**

12. References **164**

1. General Information

Reactions were performed in flame-dried glassware using conventional Schlenk techniques under a static pressure of nitrogen unless stated otherwise. Liquids and solutions were transferred with syringes. Copper salts and $NiCl_2$(dppe) were purchased from commercial suppliers and used as received. All ligands were directly purchased from *TCI*, *ABCR*, *Sigma Aldrich*, and *Solvias*. PhMgBr and *n*-hexylMgBr were purchased from *Sigma Aldrich*, and CyMgBr was prepared according to the reported procedure.[S1] All solvents (toluene, Et_2O, and THF) were dried and purified following standard procedures. Technical grade solvents for extraction or chromatography (cyclohexane, methyl *tert*-butyl ether, and *n*-pentane) were distilled prior to use. Analytical thin layer chromatography (TLC) was performed on *ALUGRAM*® Xtra SIL G/UV_{254} TLC-Sheets by *Macherey-Nagel*. Flash column chromatography was performed on silica gel 60 (40–63 µm, 230–400 mesh, ASTM) by Grace using the indicated solvents. Automatic column chromatography was performed on an Isolera One™ (*Biotage*) using KP Sil columns (10 g, 25 g). ^{1}H, ^{13}C, ^{19}F, and ^{29}Si NMR spectra were recorded in $CDCl_3$ on Bruker AV400 or AV500 instruments. Chemical shifts are reported in parts per million (ppm) and are referenced to the residual solvent resonance as the internal standard ($CHCl_3$: δ = 7.26 ppm for ^{1}H NMR and $CDCl_3$: δ = 77.16 ppm for ^{13}C NMR). Chemical shifts are reported to 0.01 ppm for ^{1}H NMR, ^{29}Si{^{1}H} DEPT NMR and ^{31}P NMR and to 0.1 ppm for ^{13}C NMR and ^{19}F NMR spectra. Peaks that are within 0.01 ppm for ^{1}H NMR or 0.1 ppm for ^{13}C NMR but are still distinguishable are reported to 0.001 ppm and 0.01 ppm, respectively. Data are reported as follows: chemical shift, multiplicity (br = broad signal, s = singlet, d = doublet, t = triplet, q = quartet, sept = septet, m = multiplet, m_c = centrosymmetric multiplet), coupling constants (Hz), and integration. Infrared (IR) spectra were recorded on an *Agilent Technologies Cary* 630 FT-IR spectrometer equipped with an ATR unit and the signals are reported in wave-numbers (cm^{-1}). Melting points (m.p.) were determined with a *Stuart Scientific* SMP20 melting point apparatus and are not corrected. High resolution mass spectrometry (HRMS) analysis was performed by the Analytical Facility at the *Institut für Chemie, Technische Universität Berlin*. Enantiomeric excesses were determined by analytical high performance liquid chromatography (HPLC) analysis on an *Agilent Technologies* 1290 Infinity instrument with a chiral stationary phase using a *Daicel* Chiralcel OD-H column, (*n*-heptane/ *i*-PrOH mixtures as solvent).

2. General Procedures

2.1 Preparation of $Me_2PhSiZnCl \cdot 2LiCl$

$Me_2PhSiZnCl \cdot 2LiCl$ was prepared according to the following reported procedure.[S2]
An oven-dried 100-mL Schlenk flask equipped with a magnetic stir bar was charged with activated lithium chunks (1.50 g, 210 mmol, 6.40 equiv.) suspended in THF (33 mL) under an atmosphere of nitrogen, the corresponding chlorosilane (33.0 mmol, 1.00 equiv.) was then added and the reaction mixture was maintained at –5 °C overnight to give $Me_2PhSiLi$. The concentration of $Me_2PhSiLi$ was determined by titration against diphenylacetic acid according to Kofron's method (~1.0 M in THF).[S3] The supernatant was then transferred to another oven-dried 100-mL Schlenk flask equipped with a stir bar under nitrogen atmosphere at –5 °C. A solution of $ZnCl_2$ (~1.0 M, 1.00 equiv., $ZnCl_2$ was dried by a heat gun for 20 min under high vacuum) was added, and the reaction mixture was stirred at this temperature for 30 min. The $Me_2PhSiZnCl \cdot 2LiCl$ solution was next allowed to warm to room temperature and immediately filtered under nitrogen atmosphere. The concentration of $Me_2PhSiZnCl \cdot 2LiCl$ was determined by titration against I_2 according to Knochel's method (~0.83 M in THF).[S4]

2.2 Preparation of $(Me_2PhSi)_2Zn \cdot xLiCl$ ($x \leqslant 4$)

$(Me_2PhSi)_2Zn \cdot xLiCl$ ($x \leqslant 4$) was prepared according to the following reported procedure.[S5]

The addition of $ZnCl_2$ (1.0 M in Et_2O, 1.00 equiv.) to a solution of $Me_2PhSiLi$ (~2.00 equiv.) at –5 °C yielded a yellow solution of $(Me_2PhSi)_2Zn \cdot 4LiCl$ (~1.00 equiv.) which was maintained at –5 °C for further 0.5 h. After warming to room temperature, the solvents were removed under high vacuum, yielding a dark brown residue. Et_2O was then added to this residue, and the suspension was immediately filtered under nitrogen atmosphere to remove undissolved LiCl. The resulting clear yellow solution in Et_2O (contaminated with traces of THF) was used directly. The concentration of $(Me_2PhSi)_2Zn \cdot xLiCl$ ($x \leqslant 4$) was determined by titration against I_2 according to Knochel's method (~0.31-0.34 M in Et_2O).[S4]

2.3 Preparation of cyclobutenones

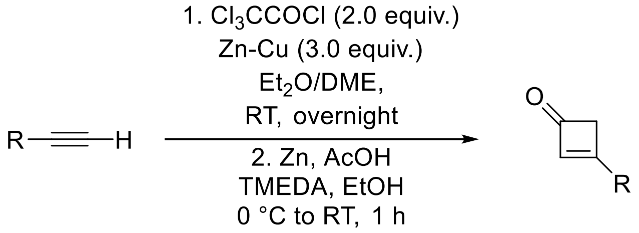

Cyclobutenones **1** were prepared according to the following modified, reported procedure.[S6] The spectroscopic data of cyclobutenones **1f**, **1c**, **1l**, **1o**, and **1p** matched those reported.[S7]

A flame-dried 100-mL three-necked round-bottom flask with an addition funnel was charged with the indicated acetylene derivative (2.20 mL, 2.04 g, 20.0 mmol), zinc-copper couple (3.90 g, 60.0 mmol), and 30 mL of Et_2O. A solution of Cl_3CCOCl (4.40 mL, 7.27 g, 40.0 mmol) in DME (10 mL) was added dropwise via the funnel over 0.5 h at 0 °C. After stirring overnight, the reaction mixture was filtered through a pad of Celite®. The filtrate was washed with ice-cold HCl (0.5 M, 50 mL), ice-cold NaOH (15%, 30 mL), brine, dried over Na_2SO_4, filtered and evaporated under vacuum to afford the corresponding dichlorocyclobutenone, which was directly utilized for next step without purification.

To a suspension of activated zinc dust (activated by stirring with dilute HCl; 7.88 g, 121 mmol), TMEDA (18.0 mL, 14.1 g, 121 mmol), and EtOH (30 mL) was added HOAc (3.30 mL, 3.46 g, 57.6 mmol) dropwise at 0 °C. The indicated dichlorocyclobutenone from previous step in EtOH (12 mL) was added dropwise via an addition funnel over 0.5 h. The reaction mixture was stirred at 0 °C for 20 min before warmed to room temperature. After strring untill TLC analysis indicated full conversion (1 to 2 h), the mixture was filtered through a pad of Celite®, concentrated, washed with HCl (1N, 30 mL), and extracted with Et_2O. The combined organic phases were washed with a saturated aqueous solution of $NaHCO_3$ (50 mL) and brine, dried over Na_2SO_4, concentrated, and purified by column chromatography on silica gel (cyclohexane:methyl *tert*-butyl ether = 5:1 to 20:1) to afford the corresponding cyclobutenones.

3. Optimization Study

Table S1. Optimization of the 1,4-addition/trapping reaction of **1a**.

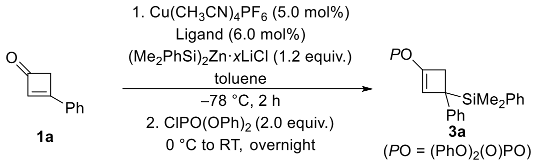

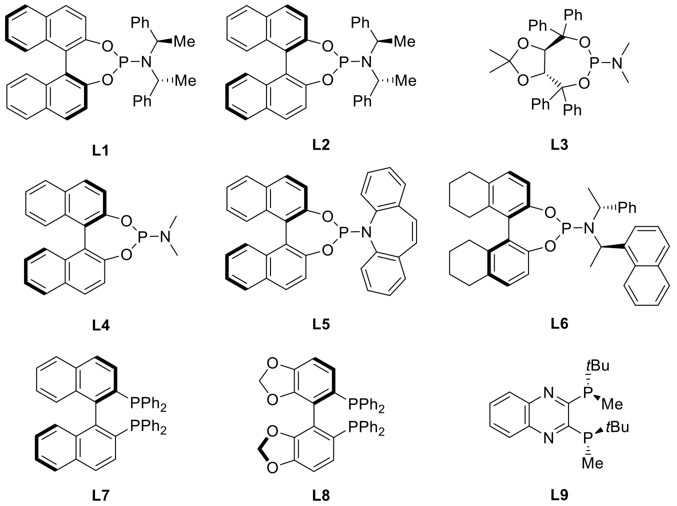

Entry	Copper salt	Ligand	Solvent	T [°C]	Yield (%)[a]	ee (%)[b]
1[c]	$Cu(CH_3CN)_4PF_6$	**L1**	Toluene	–78 °C	53	15
2	$Cu(CH_3CN)_4PF_6$	**L1**	Toluene	–78 °C	55	6
3	$Cu(CH_3CN)_4PF_6$	**L2**	Toluene	–78 °C	70	0
4	$Cu(CH_3CN)_4PF_6$	**L3**	Toluene	–78 °C	55	0
5	$Cu(CH_3CN)_4PF_6$	**L4**	Toluene	–78 °C	32	0
6	$Cu(CH_3CN)_4PF_6$	**L5**	Toluene	–78 °C	24	0
7	$Cu(CH_3CN)_4PF_6$	**L6**	Toluene	–78 °C	51	0
8	$Cu(CH_3CN)_4PF_6$	**L7**	Toluene	–78 °C	26	0
9	$Cu(CH_3CN)_4PF_6$	**L8**	Toluene	–78 °C	31	0
10	$Cu(CH_3CN)_4PF_6$	**L9**	Toluene	–78 °C	35	0
11	$Cu(CH_3CN)_4BF_4$	**L2**	Toluene	–78 °C	60	0
12	$Cu(OTf)_2$	**L2**	Toluene	–78 °C	45	0
13	CuBr	**L2**	Toluene	–78 °C	69	0
14	CuCl	**L2**	Toluene	–78 °C	45	0
15	CuCN	**L2**	Toluene	–78 °C	68	0
16	$Cu(CH_3CN)_4PF_6$	**L2**	THF	–78 °C	76	0
17[d]	$Cu(CH_3CN)_4PF_6$	**L2**	THF	–78 °C	67	0

[a] Determined by ^1H NMR spectroscopy by the addition of CH_2Br_2 as the internal standard. [b] Enantiomeric excesses determined by HPLC on chiral stationary phases (*Daicel* Chiralcel OD-H column, column temperature 20°C, solvent *n*-heptane:*i*-PrOH = 98:2, flow rate 0.3 mL/min): t_R = 48.4 min (major), t_R = 55.5 min (minor). [c] The first step was conducted at –78 °C for 16 h and then $ClPO(OPh)_2$ was added. [d] $(Me_2PhSi)_2Zn\cdot xLiCl$ (x ≤ 4; 1.5 equiv.) was added.

4. General Procedures for Conjugate Silyl Additions to Cyclobutenones (GP1)

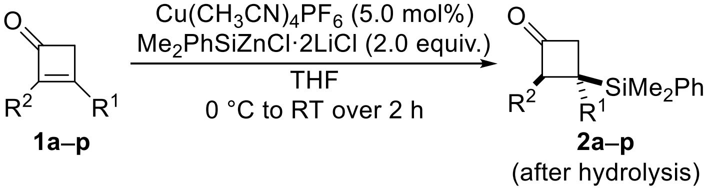

An oven-dried 10-mL Schlenk tube equipped with a magnetic stir bar was charged with $Cu(CH_3CN)_4PF_6$ (3.73 mg, 5.00 mol%) and the indicated cyclobutenone **1** (0.200 mmol, 1.00 equiv.). The tube was evacuated under high vacuum and backfilled with nitrogen gas (3 times). THF (1 mL) was added to the tube, and the resulting suspension was stirred under room temperature for 5 min. The mixture was then cooled to 0 °C, and a solution of $Me_2PhSiZnCl\cdot 2LiCl$ (0.482 mL, 0.400 mmol, 0.83 M in THF, 2.00 equiv.) was added dropwise under a static pressure of nitrogen gas, and then the mixture was stirred under room temperature for 2 h. After the indicated reaction time, the reaction mixture was quenched with a saturated aqueous solution of NH_4Cl, and extracted with Et_2O. The combined organic phases were washed with brine, dried over anhydrous Na_2SO_4, and concentrated under reduced pressure. The residue was purified by flash column chromatography on silica gel using the indicated mixture of cyclohexane and methyl *tert*-butyl ether.

5. General Procedures for Conjugate Silylation/Trapping Reactions (GP2)

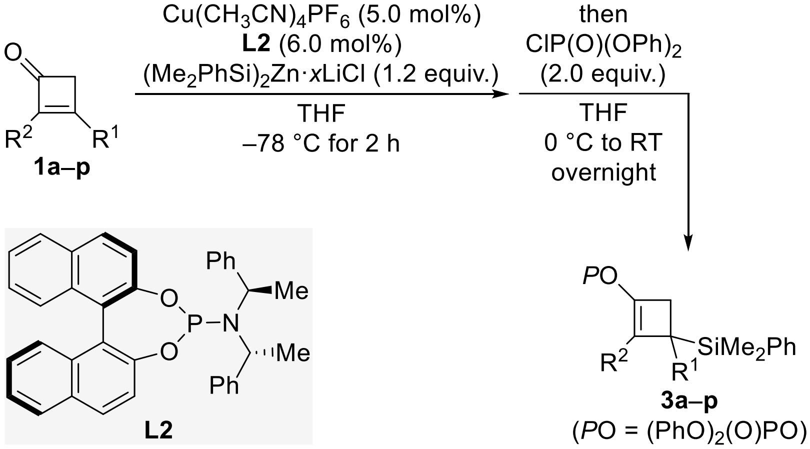

An oven-dried 10-mL Schlenk tube equipped with a magnetic stir bar was charged with $Cu(CH_3CN)_4PF_6$ (3.73 mg, 5.00 mol%), **L2** (6.48 mg, 6.00 mol%). The tube was evacuated under high vacuum and backfilled with nitrogen (3 times). THF (1 mL) was added to the tube, and the resulting suspension was stirred under room temperature for 30 min before the indicated cyclobutenone **1** (0.200 mmol, 1.00 equiv.) were added. The mixture was then cooled to –78 °C in a dry ice/acetone bath, and a solution of $(Me_2PhSi)_2Zn\cdot x LiCl$ ($x \leqslant 4$) (0.71–0.77 mL, 0.240 mmol, 1.20 equiv., ~0.31–0.34 M in

Et_2O) was added dropwise under a static pressure of nitrogen gas, and the mixture was maintained at –78 °C for 2 h. After the indicated reaction time, $ClPO(OPh)_2$ (108 mg, 0.400 mmol, 2.00 equiv.) and THF (1 mL) were added at –78 °C. The reaction mixture was then warmed to 0 °C and stirred at room temperature overnight. The reaction mixture was quenched with a saturated aqueous solution of NH_4Cl, and extracted with Et_2O. The combined organic phase was washed with brine, dried over anhydrous Na_2SO_4, and concentrated under reduced pressure, and the residue was purified by automatic column chromatography using Isolera™ One with cyclohexane/ethyl acetate as eluents affords cyclobutenyl phosphates.

6. General Procedures for Kumada Coupling of Enol Phosphates (GP3)

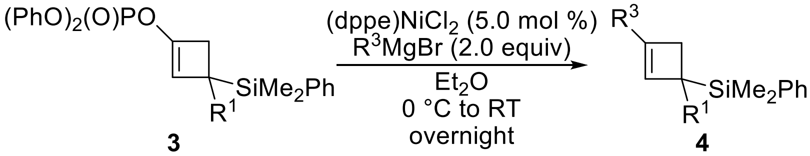

3 **4**

An oven-dried 10-mL Schlenk tube equipped with a magnetic stir bar was charged with $NiCl_2$(dppe) (2.64 mg, 5.00 mol%). The tube was evacuated under high vacuum and backfilled with nitrogen gas (3 times). Et_2O (1 mL) was added to the tube, and the resulting suspension was then cooled to 0°C with an ice bath. A solution of the indicated cyclobutenyl phosphate **3** (0.100 mmol, 1.00 equiv.) in Et_2O was added to the mixture, and Grignard reagent R^3MgX (2.00 equiv.) was added dropwise by syringe under the a static pressure of nitrogen gas. The mixture was stirred under room temperature overnight. After the indicated reaction time, the reaction mixture was quenched with a saturated aqueous solution of NH_4Cl and extracted with Et_2O. The combined organic phases were washed with brine, dried over anhydrous Na_2SO_4, and concentrated under reduced pressure. And the residue was purified by flash column chromatography on silica gel using the indicated mixture of cyclohexane and methyl *tert*-butyl ether.

7. Experimental Details for Conjugate Silylation of Cyclobutenones

7.1 3-(Dimethyl(phenyl)silyl)-3-phenylcyclobutan-1-one (2a)

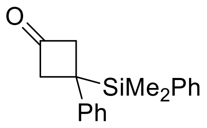

2a
$C_{18}H_{20}OSi$
M = 280.44 g/mol

Prepared from 3-phenylcyclobut-2-en-1-one (**1a**, 28.8 mg, 0.200 mmol,1.00 equiv.) and $Me_2PhSiZnCl{\cdot}2LiCl$ (0.482 mL, 0.400 mmol, 0.83 M in THF, 2.00 equiv.) according to **GP1**. The residue was purified by flash column chromatography on silica gel using cyclohexane:methyl *tert*-butyl ether = 10:1 as the eluent to afford **2a** as a white solid (53.3 mg, 95% yield).

M.p. = 86–87°C (cyclohexane). $\mathbf{R_f}$ = 0.32 (cyclohexane:methyl *tert*-butyl ether = 10:1). **IR** (ATR): $\tilde{\nu}$ = 700, 738, 769, 809, 1111, 1248, 1381, 1425, 1778, 2955 cm^{-1}. $\mathbf{^1H}$ **NMR** (500 MHz, $CDCl_3$) δ = 0.33 (s, 6H), 3.41–3.35 (m, 2H), 3.51–3.45 (m, 2H), 6.90 (d, *J* = 7.6 Hz, 2H), 7.21–7.15 (m, 1H), 7.35–7.27 (m, 6H), 7.43–7.39 (m, 1H) ppm. $\mathbf{^{13}C}$ **NMR** (126 MHz, $CDCl_3$) δ = –6.0, 27.0, 56.1, 124.9, 126.9, 127.7, 127.9, 129.7, 134.5, 134.7, 146.6, 205.3 ppm. $\mathbf{^{29}Si\{^1H\}}$ **DEPT NMR** δ = 0.90 ppm. **HRMS** (APCI) exact mass for $[M+H]^+$ $C_{18}H_{21}OSi^+$: calculated 281.1356, found 281.1358.

7.2 3-(Dimethyl(phenyl)silyl)-3-(p-tolyl)cyclobutan-1-one (2b)

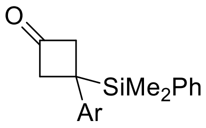

2b (Ar = 4-MeC_6H_4)
$C_{19}H_{22}OSi$
M = 294.47 g/mol

Prepared from 3-(p-tolyl)cyclobut-2-en-1-one (**1b**, 31.6 mg, 0.200 mmol, 1.00 equiv.) and $Me_2PhSiZnCl{\cdot}2LiCl$ (0.482 mL, 0.400 mmol, 0.83 M in THF, 2.00 equiv.) according to **GP1**. The residue was purified by flash column chromatography on silica gel using cyclohexane:methyl *tert*-butyl ether = 10:1 as the eluent to afford **2b** as a white solid (58.9 mg, quant yield).

M.p. = 87–88°C (cyclohexane). $\mathbf{R_f}$ = 0.31 (cyclohexane:methyl *tert*-butyl ether = 10:1). **IR** (ATR): $\tilde{\nu}$ = 698, 734, 777, 805, 1108, 1246, 1381, 1780, 2903, 2951 cm^{-1}. $\mathbf{^1H}$ **NMR** (500 MHz, $CDCl_3$): δ = 0.30 (s, 6H), 2.33 (s, 3H), 3.35–3.28 (m, 2H), 3.47–3.39 (m, 2H), 6.77 (d, *J* = 8.2 Hz, 2H), 7.06 (d, *J* = 8.2 Hz, 2H), 7.33–7.28 (m, 4H), 7.40–7.36 (m, 1H) ppm. $\mathbf{^{13}C}$ **NMR** (126 MHz, $CDCl_3$) δ = –5.9, 20.9, 26.4, 56.1, 126.9, 127.7, 128.5, 129.7, 134.4,

134.5, 134.9, 143.5, 205.5 ppm. **^{29}Si{^{1}H} DEPT NMR** δ = 0.60 ppm. **HRMS** (APCI) exact mass for $[M+H]^+$ $C_{19}H_{23}OSi^+$: calculated 295.1513, found 295.1505.

7.3 3-(Dimethyl(phenyl)silyl)-3-(m-tolyl)cyclobutan-1-one (2c)

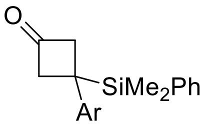

2c (Ar = 3-MeC_6H_4)
$C_{19}H_{22}OSi$
M = 294.47 g/mol

Prepared from 3-(m-tolyl)cyclobut-2-en-1-one (**1c**, 31.6 mg, 0.200 mmol, 1.00 equiv.) and $Me_2PhSiZnCl\cdot 2LiCl$ (0.482 mL, 0.400 mmol, 0.83 M in THF, 2.00 equiv.) according to **GP1**. The residue was purified by flash column chromatography on silica gel using using cyclohexane:methyl *tert*-butyl ether = 10:1 as the eluent to afford **2c** as a white solid (58.3 mg, 99% yield).

M.p. = 65–67°C (cyclohexane). **R_f** = 0.33 (cyclohexane:methyl *tert*-butyl ether = 10:1). **IR** (ATR): $\tilde{\nu}$ = 694, 734, 773, 811, 829, 1115, 1247, 1424, 1769, 2898, 2954 cm^{-1}. **^{1}H NMR** (500 MHz, $CDCl_3$) δ = 0.32 (s, 6H), 2.28 (s, 3H), 3.39–3.33 (m, 2H), 3.48–3.43 (m, 2H), 6.62 (s, 1H), 6.72 (d, *J* = 7.8 Hz, 1H), 6.99 (d, *J* = 7.5 Hz, 1H), 7.18–7.14 (m, 1H), 7.34–7.24 (m, 4H), 7.42–7.38 (m, 1H) ppm. **^{13}C NMR** (126 MHz, $CDCl_3$) δ = –6.0, 21.5, 26.8, 56.0, 124.0, 125.7, 127.65, 127.73, 127.9, 129.7, 134.6, 134.8, 137.4, 146.4, 205.5 ppm. **^{29}Si{^{1}H} DEPT NMR** δ = 0.78 ppm. **HRMS** (APCI) exact mass for $[M+H]^+$ $C_{19}H_{23}OSi^+$: calculated 295.1513, found 295.1506.

7.4 3-(Dimethyl(phenyl)silyl)-3-(4-methoxyphenyl)cyclobutan-1-one (2d)

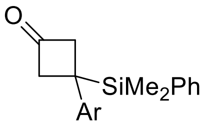

2d (Ar = 4-$MeOC_6H_4$)
$C_{19}H_{22}O_2Si$
M = 310.47 g/mol

Prepared from 3-(4-methoxyphenyl)cyclobut-2-en-1-one (**1d**, 34.8 mg, 0.200 mmol, 1.00 equiv.) and $Me_2PhSiZnCl\cdot 2LiCl$ (0.482 mL, 0.400 mmol, 0.83 M in THF, 2.00 equiv.) according to **GP1**. The residue was purified by flash column chromatography on silica gel using cyclohexane:methyl *tert*-butyl ether = 10:1 as the eluent to afford **2d** as a white solid (58.4 mg, 94% yield).

M.p. = 85–86°C (cyclohexane). **R_f** = 0.21 (cyclohexane:methyl *tert*-butyl ether = 10:1). **IR** (ATR): $\tilde{\nu}$ = 703, 739, 775, 805, 1026, 1106, 1243, 1506, 1778, 2905, 2950 cm^{-1}. **^{1}H NMR** (500 MHz, $CDCl_3$) δ = 0.30 (s, 6H), 3.33–3.28 (m, 2H), 3.45–3.40 (m, 2H), 3.80 (s, 3H), 6.82–6.77 (m, 4H), 7.33–7.28 (m, 4H), 7.41–7.36 (m, 1H) ppm. **^{13}C NMR** (126 MHz, $CDCl_3$) δ = –5.9, 26.0, 55.3, 56.1, 113.4, 127.7, 128.0, 129.7, 134.5, 134.9, 138.5, 157.2,

205.4 ppm. **^{29}Si{^{1}H} DEPT NMR** δ = 0.60 ppm. **HRMS** (APCI) exact mass for $[M+H]^+$ $C_{19}H_{23}O_2Si^+$: calculated 311.1462, found 311.1458.

7.5 3-(4-Chlorophenyl)-3-(dimethyl(phenyl)silyl)cyclobutan-1-one (2e)

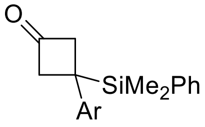

2e (Ar = 4-ClC_6H_4)
$C_{18}H_{19}ClOSi$
M = 314.88 g/mol

Prepared from 3-(4-chlorophenyl)cyclobut-2-en-1-one (**1e**, 35.7 mg, 0.200 mmol, 1.00 equiv.) and $Me_2PhSiZnCl·2LiCl$ (0.482 mL, 0.400 mmol, 0.83 M in THF, 2.00 equiv.) according to **GP1**. The residue was purified by flash column chromatography on silica gel using cyclohexane:methyl *tert*-butyl ether = 10:1 as the eluent to afford **2e** as a white solid (52.9 mg, 84% yield).

M.p. = 73–74°C (cyclohexane). **R_f** = 0.28 (cyclohexane:methyl *tert*-butyl ether = 10:1). **IR** (ATR): $\tilde{\nu}$ = 676, 701, 772, 806, 1107, 1249, 1487, 1778, 2903, 2950 cm^{-1}. **^{1}H NMR** (500 MHz, $CDCl_3$) δ = 0.29 (s, 6H), 3.32–3.23 (m, 2H), 3.48–3.39 (m, 2H), 6.76 (d, *J* = 8.4 Hz, 2H), 7.19 (d, *J* = 8.3 Hz, 2H), 7.27–7.23 (m, 2H), 7.32–7.28 (m, 2H), 7.41–7.35 (m, 1H) ppm. **^{13}C NMR** (126 MHz, $CDCl_3$) δ = –6.1, 26.8, 56.1, 127.8, 128.0, 128.3, 129.9, 130.9, 134.3, 134.4, 145.2, 204.5 ppm. **^{29}Si{^{1}H} DEPT NMR** δ = 1.00 ppm. **HRMS** (APCI) exact mass for $[M+H]^+$ $C_{18}H_{20}ClOSi^+$: calculated 315.0966, found 315.0963.

7.6 3-(Dimethyl(phenyl)silyl)-3-(4-fluorophenyl)cyclobutan-1-one (2f)

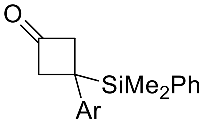

2f (Ar = 4-FC_6H_4)
$C_{18}H_{19}FOSi$
M = 298.43 g/mol

Prepared from 3-(4-fluorophenyl)cyclobut-2-en-1-one (**1f**, 32.4 mg, 0.200 mmol, 1.00 equiv.) and $Me_2PhSiZnCl·2LiCl$ (0.482 mL, 0.400 mmol, 0.83 M in THF, 2.00 equiv.) according to **GP1**. The residue was purified by flash column chromatography on silica gel using cyclohexane:methyl *tert*-butyl ether = 10:1 as the eluent to afford **2f** as a white solid (47.7 mg, 80% yield).

M.p. = 94–95°C (cyclohexane). **R_f** = 0.30 (cyclohexane:methyl *tert*-butyl ether = 10:1). **IR** (ATR): $\tilde{\nu}$ = 698, 733, 774, 810, 1114, 1210, 1249, 1504, 1777, 2915, 2955 cm^{-1}. **^{1}H NMR** (400 MHz, $CDCl_3$) δ = 0.31 (s, 6H), 3.34–3.27 (m, 2H), 3.49–3.41 (m, 2H), 6.82–6.75 (m, 2H), 6.97–6.90 (m, 2H), 7.28–7.24 (m, 2H), 7.34–7.29 (m, 2H), 7.42–7.37 (m, 1H) ppm. **^{13}C NMR** (101 MHz, $CDCl_3$) δ = –6.1, 26.5, 56.2, 114.6, 114.8, 127.8, 128.3 (d, *J* = 8.5 Hz), 129.9, 134.4, 142.2 (d, *J* = 2.9 Hz), 160.7 (d, *J* = 244.8 Hz), 204.8 ppm. **^{29}Si{^{1}H}**

DEPT NMR δ = 0.95 ppm. **^{19}F NMR** (470 MHz, $CDCl_3$) δ = –118.5 ppm. **HRMS** (APCI) exact mass for $[M+H]^+$ $C_{18}H_{20}FOSi^+$: calculated 299.1262, found 299.1259.

7.7 3-Butyl-3-(dimethyl(phenyl)silyl)cyclobutan-1-one (2g)

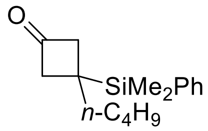

2g

$C_{16}H_{24}OSi$

M = 260.45 g/mol

Prepared from 3-butylcyclobut-2-en-1-one (**1g**, 24.8 mg, 0.200 mmol, 1.00 equiv.) and $Me_2PhSiZnCl·2LiCl$ (0.482 mL, 0.400 mmol, 0.83 M in THF, 2.00 equiv.) according to **GP1**. The residue was purified by flash column chromatography on silica gel using cyclohexane:methyl *tert*-butyl ether = 10:1 as the eluent to afford **2g** as a colorless oil (52.1 mg, quantitative yield).

R_f = 0.34 (cyclohexane:methyl *tert*-butyl ether = 10:1). **IR** (ATR): $\tilde{\nu}$ = 698, 732, 770, 810, 1049, 1110, 1251, 1426, 1776, 2927, 2954 cm^{-1}. **^{1}H NMR** (500 MHz, $CDCl_3$) δ = 0.38 (s, 6H), 0.89–0.82 (t, *J* = 6.7 Hz, 3H), 1.30–1.23 (m, 4H), 1.66–1.59 (m, 2H), 2.78–2.69 (m, 2H), 3.07–3.00 (m, 2H), 7.41–7.33 (m, 3H), 7.55–7.48 (m, 2H) ppm. **^{13}C NMR** (126 MHz, $CDCl_3$) δ = –4.6, 13.9, 18.7, 23.4, 28.7, 38.3, 54.0, 128.0, 129.4, 134.1, 136.7, 207.5 ppm. **^{29}Si{^{1}H} DEPT NMR** δ = 1.12. **HRMS** (APCI) exact mass for $[M+H]^+$ $C_{16}H_{25}OSi^+$: calculated 261.1669, found 261.1666.

7.8 3-(Dimethyl(phenyl)silyl)-3-hexylcyclobutan-1-one (2h)

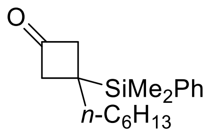

2h

$C_{18}H_{28}OSi$

M = 288.51 g/mol

Prepared from 3-hexylcyclobut-2-en-1-one (**1h**, 30.4 mg, 0.200 mmol, 1.00 equiv.) and $Me_2PhSiZnCl·2LiCl$ (0.482 mL, 0.400 mmol, 0.83 M in THF, 2.00 equiv.) according to **GP1**. The residue was purified by flash column chromatography on silica gel using cyclohexane:methyl *tert*-butyl ether = 10:1 as the eluent to afford **2h** as a yellow oil (53.1 mg, 92% yield).

R_f = 0.38 (cyclohexane:methyl *tert*-butyl ether = 10:1). **IR** (ATR): $\tilde{\nu}$ = 698, 731, 771, 810, 1050, 1108, 1250, 1426, 1777, 2925, 2953 cm^{-1}. **^{1}H NMR** (400 MHz, $CDCl_3$) δ = 0.38 (s, 6H), 0.86 (t, *J* = 6.7 Hz, 3H), 1.28–1.19 (m, 8H), 1.64–1.57 (m, 2H), 2.78–2.69 (m, 2H), 3.07–2.99 (m, 2H), 7.40–7.35 (m, 3H), 7.53–7.49 (m, 2H) ppm. **^{13}C NMR** (101 MHz,

$CDCl_3$) δ = –4.6, 14.1, 18.8, 22.6, 26.5, 30.0, 31.7, 38.7, 54.1, 128.0, 129.5, 134.1, 136.7, 207.6 ppm. **^{29}Si{^{1}H} DEPT NMR** δ = 1.24 ppm. **HRMS** (APCI) exact mass for $[M+H]^+$ $C_{18}H_{29}OSi^+$: calculated 289.1982, found 289.1981.

7.9 3-(Dimethyl(phenyl)silyl)-3-phenethylcyclobutan-1-one (2i)

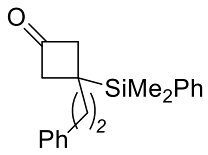

2i
$C_{20}H_{24}OSi$
M = 308.50 g/mol

Prepared from 3-phenethylcyclobut-2-en-1-one (**1i**, 34.4 mg, 0.200 mmol, 1.00 equiv.) and $Me_2PhSiZnCl·2LiCl$ (0.482 mL, 0.400 mmol, 0.83 M in THF, 2.00 equiv.) according to **GP1**. The residue was purified by flash column chromatography on silica gel using cyclohexane:methyl *tert*-butyl ether = 10:1 as the eluent to afford **2i** as a colorless oil (56.1 mg, 91% yield).

R_f = 0.22 (cyclohexane:methyl *tert*-butyl ether = 10:1). **IR** (ATR): $\tilde{\nu}$ = 697, 734, 771, 810, 1109, 1252, 1426, 1774, 2907, 2950 cm^{-1}. **^{1}H NMR** (500 MHz, $CDCl_3$) δ = 0.32 (s, 6H), 1.85–1.78 (m, 2H), 2.47–2.41 (m, 2H), 2.75–2.69 (m, 2H), 3.04–2.98 (m, 2H), 6.96–6.92 (m, 2H), 7.07–7.03 (m, 1H), 7.15–7.11 (m, 2H), 7.32–7.26 (m, 3H), 7.46–7.42 (m, 2H) ppm. **^{13}C NMR** (126 MHz, $CDCl_3$) δ = –4.6, 19.1, 33.1, 41.1, 54.2, 126.0, 128.11, 128.13, 128.5, 129.6, 134.1, 136.5, 142.0, 206.9 ppm. **^{29}Si{^{1}H} DEPT NMR** δ = 1.15 ppm. **HRMS** (APCI) exact mass for $[M+H]^+$ $C_{20}H_{25}OSi^+$: calculated 309.1669, found 309.1668.

7.10 3-(Dimethyl(phenyl)silyl)-3-(3-phenylpropyl)cyclobutan-1-one (2j)

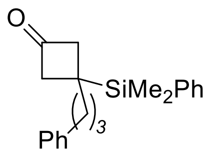

2j
$C_{21}H_{26}OSi$
M = 322.52 g/mol

Prepared from 3-(3-phenylpropyl)cyclobut-2-en-1-one (**1j**, 37.3 mg, 0.200 mmol, 1.00 equiv.) and $Me_2PhSiZnCl·2LiCl$ (0.482 mL, 0.400 mmol, 0.83 M in THF, 2.00 equiv.) according to **GP1**. The residue was purified by flash column chromatography on silica gel using cyclohexane:methyl *tert*-butyl ether = 10:1 as the eluent to afford **2**j as a colorless oil (51.6 mg, 80% yield).

R_f = 0.27 (cyclohexane:methyl *tert*-butyl ether = 10:1). **IR** (ATR): $\tilde{\nu}$ = 697, 733, 771, 810, 1108, 1251, 1774, 2853, 2933 cm^{-1}. **^{1}H NMR** (500 MHz, $CDCl_3$) δ = 0.36 (s, 6H), 1.67–1.56 (m, 4H), 2.57–2.53 (m, 2H), 2.75–2.69 (m, 2H), 3.07–3.00 (m, 2H), 7.11–7.06 (m,

2H), 7.20–7.16 (m, 1H), 7.29–7.23 (m, 2H), 7.40–7.34 (m, 3H), 7.50–7.45 (m, 2H) ppm. **^{13}C NMR** (126 MHz, $CDCl_3$) δ = –4.6, 18.8, 28.3, 36.4, 38.2, 54.2, 125.9, 128.0, 128.29, 128.34, 129.5, 134.0, 136.6, 141.9, 207.2 ppm. **^{29}Si{^{1}H} DEPT NMR** δ = 1.08 ppm. **HRMS** (APCI) exact mass for $[M+H]^+$ $C_{21}H_{27}OSi^+$: calculated 323.1826, found 323.1825.

7.11 3-(4-Chlorobutyl)-3-(dimethyl(phenyl)silyl)cyclobutan-1-one (2k)

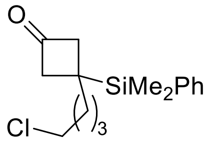

2k
$C_{16}H_{23}ClOSi$
M = 294.89 g/mol

Prepared from 3-(4-chlorobutyl)cyclobut-2-en-1-one (**1k**, 31.7 mg, 0.200 mmol, 1.00 equiv.) and $Me_2PhSiZnCl·2LiCl$ (0.482 mL, 0.400 mmol, 0.83 M in THF, 2.00 equiv.) according to **GP1**. The residue was purified by flash column chromatography on silica gel using cyclohexane:methyl *tert*-butyl ether = 10:1 as the eluent to afford **2k** as a colorless oil (45.4 mg, 77% yield).

R_f = 0.18 (cyclohexane:methyl *tert*-butyl ether = 10:1). **IR** (ATR): $\tilde{\nu}$ = 700, 734, 771, 811, 1109, 1252, 1384, 1426, 1774, 2909, 2951 cm^{-1}. **^{1}H NMR** (400 MHz, $CDCl_3$) δ = 0.39 (s, 6H), 1.48–1.36 (m, 2H), 1.75–1.60 (m, 4H), 2.79–2.70 (m, 2H), 3.11–3.02 (m, 2H), 3.47 (t, *J* = 6.6 Hz, 2H), 7.42–7.33 (m, 3H), 7.54–7.48 (m, 2H) ppm. **^{13}C NMR** (101 MHz, $CDCl_3$) δ = –4.6, 18.8, 23.9, 33.0, 37.9, 44.7, 54.2, 128.1, 129.6, 134.1, 136.5, 207.0 ppm. **^{29}Si{^{1}H} DEPT NMR** δ = 1.13 ppm. **HRMS** (APCI) exact mass for $[M+H]^+$ $C_{16}H_{24}ClOSi^+$: calculated 295.1279, found 295.1278.

7.12 3-Cyclopropyl-3-(dimethyl(phenyl)silyl)cyclobutan-1-one (2l)

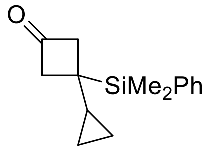

2l
$C_{15}H_{20}OSi$
M = 244.41 g/mol

Prepared from 3-cyclopropylcyclobut-2-en-1-one (**1l**, 21.6 mg, 0.200 mmol, 1.00 equiv.) and $Me_2PhSiZnCl·2LiCl$ (0.482 mL, 0.400 mmol, 0.83 M in THF, 2.00 equiv.) according to **GP1**. The residue was purified by flash column chromatography on silica gel using cyclohexane:methyl *tert*-butyl ether = 10:1 as the eluent to afford **2l** as a yellow oil (37.6 mg, 77% yield).

R_f = 0.51 (cyclohexane:methyl *tert*-butyl ether = 10:1). **IR** (ATR): $\tilde{\nu}$ = 697, 732, 772, 810, 1047, 1107, 1426, 1777, 2903, 2954 cm^{-1}. **^{1}H NMR** (400 MHz, $CDCl_3$) δ = 0.15–0.11 (m,

2H), 0.41 (s, 6H), 0.46–0.41 (m, 2H), 1.13–1.05 (m, 1H), 2.55–2.48 (m, 2H), 2.90–2.82 (m, 2H), 7.41–7.35 (m, 3H), 7.58–7.53 (m, 2H) ppm. **^{13}C NMR** (101 MHz, $CDCl_3$) δ = –6.3, 0.0, 14.4, 17.2, 50.0, 127.1, 128.7, 133.5, 135.5, 206.3 ppm. **^{29}Si{^{1}H} DEPT NMR** δ = 1.77 ppm. **HRMS** (APCI) exact mass for $[M+H]^+$ $C_{15}H_{21}OSi^+$: calculated 245.1356, found 245.1355.

7.13 3-Cyclohexyl-3-(dimethyl(phenyl)silyl)cyclobutan-1-one (2m)

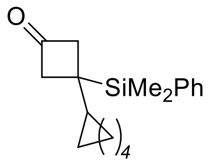

2m
$C_{18}H_{26}OSi$
M = 286.49 g/mol

Prepared from 3-cyclohexylcyclobut-2-en-1-one (**1m**, 30.0 mg, 0.200 mmol, 1.00 equiv.) and $Me_2PhSiZnCl·2LiCl$ (0.482 mL, 0.400 mmol, 0.83 M in THF, 2.00 equiv.) according to **GP1**. The residue was purified by flash column chromatography on silica gel using cyclohexane:methyl *tert*-butyl ether = 10:1 as the eluent to afford **2m** as a yellow oil (49.8 mg, 87% yield).

R_f = 0.35 (cyclohexane:methyl *tert*-butyl ether = 10:1). **IR** (ATR): $\tilde{\nu}$ = 703, 736, 769, 816, 905, 1110, 1245, 1379, 1426, 2852, 2924 cm^{-1}. **^{1}H NMR** (500 MHz, $CDCl_3$) δ = 0.41 (s, 6H), 1.17–0.95 (m, 5H), 1.47–1.36 (m, 1H), 1.76–1.60 (m, 5H), 2.88–2.80 (m, 2H), 3.10–3.02 (m, 2H), 7.39–7.33 (m, 3H), 7.54–7.49 (m, 2H) ppm. **^{13}C NMR** (126 MHz, $CDCl_3$) δ = –2.7, 24.0, 26.3, 27.1, 30.5, 49.0, 53.8, 127.9, 129.3, 134.2, 137.8, 207.6 ppm. **^{29}Si{^{1}H} DEPT NMR** δ = 0.05 ppm. **HRMS** (APCI) exact mass for $[M+H]^+$ $C_{18}H_{27}OSi^+$: calculated 287.1826, found 287.1825.

7.14 3-(Dimethyl(phenyl)silyl)-3-(trimethylsilyl)cyclobutan-1-one (2n)

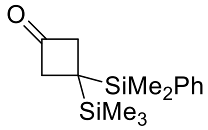

2n
$C_{15}H_{24}OSi_2$
M = 276.53 g/mol

Prepared from 1,4-dihydro-1,4-epoxynaphthalene (**1n**, 28.1 mg, 0.200 mmol, 1.00 equiv.) and $Me_2PhSiZnCl·2LiCl$ (0.482 mL, 0.400 mmol, 0.83 M in THF, 2.00 equiv.) according to **GP1**. The residue was purified by flash column chromatography on silica gel using cyclohexane:methyl *tert*-butyl ether = 10:1 as the eluent to afford **2n** as a yellow oil (47.0 mg, 85% yield).

R$_f$ = 0.39 (cyclohexane:methyl *tert*-butyl ether = 10:1). **IR** (ATR): $\tilde{\nu}$ = 700, 772, 809, 832, 976, 1107, 1426, 1778, 2901, 2953 cm^{-1}. **^{1}H NMR** (500 MHz, $CDCl_3$) δ = –0.05 (s, 9H), 0.39 (s, 6H), 3.04–2.98 (m, 2H), 3.17–3.10 (m, 2H), 7.39–7.35 (m, 3H), 7.52–7.49 (m, 2H) ppm. **^{13}C NMR** (101 MHz, $CDCl_3$) δ = –1.4, –0.0, 6.1, 53.7, 130.2, 131.8, 136.5, 139.4, 209.1 ppm. **^{29}Si{^{1}H} DEPT NMR** δ = 8.2 ppm. **HRMS** (APCI) exact mass for $[M+H]^+$ $C_{15}H_{25}OSi_2^+$: calculated 277.1438, found 277.1440.

7.15 3-(Dimethyl(phenyl)silyl)-2-methyl-3-phenylcyclobutan-1-one (2o)

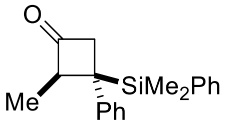

2o
$C_{19}H_{22}OSi$
M = 294.47 g/mol

Prepared from 2-methyl-3-phenylcyclobut-2-en-1-one (**1o**, 31.6 mg, 0.200 mmol, 1.00 equiv.) and $Me_2PhSiZnCl\cdot 2LiCl$ (0.482 mL, 0.400 mmol, 0.83 M in THF, 2.00 equiv.) according to **GP1**. The diastereomeric ratio was determined by relative ^{1}HNMR peak height of the Me groups after quenched with a saturated aqueous solution of NH_4Cl. The residue was purified by flash column chromatography on silica gel using cyclo-hexane:methyl *tert*-butyl ether = 10:1 as the eluent to afford **2o** as a white solid (55.9 mg, d.r = 98:2, 95% yield).

M.p. = 93–95°C (cyclohexane). **R$_f$** = 0.26 (cyclohexane:methyl *tert*-butyl ether = 10:1). **IR** (ATR): $\tilde{\nu}$ = 694, 735, 770, 807, 830, 1108, 1240, 1413, 1767, 2948, 2916, 2957 cm^{-1}. **^{1}H NMR** (500 MHz, $CDCl_3$) δ = 0.41 (s, 3H), 0.43 (s, 3H), 1.54 (d, *J* = 7.4 Hz, 3H), 3.36–3.30 (m, 1H), 3.46–3.40 (m, 1H), 3.69 (q, *J* = 7.7 Hz, 1H), 6.93–6.87 (m, 2H), 7.21–7.17 (m, 2H), 7.25–7.21 (m, 1H), 7.33–7.25 (m, 4H), 7.42–7.37 (m, 1H) ppm. **^{13}C NMR** (126 MHz, $CDCl_3$) δ = –4.0, –3.9, 13.3, 33.7, 53.8, 65.0, 125.2, 126.9, 127.4, 127.7, 129.4, 134.8, 135.6, 148.5, 207.8 ppm. **^{29}Si{^{1}H} DEPT NMR** δ = –1.37 ppm. **HRMS** (APCI) exact mass for $[M+H]^+$ $C_{19}H_{23}OSi^+$: calculated 295.1513, found 295.1512.

7.16 3-(Dimethyl(phenyl)silyl)-2,3-dipropylcyclobutan-1-one (2p)

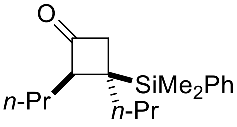

2p
$C_{18}H_{28}OSi$
M = 288.51 g/mol

Prepared from 2,3-dipropylcyclobut-2-en-1-one (**1p**, 30.4 mg, 0.200 mmol, 1.00 equiv.) and $Me_2PhSiZnCl\cdot 2LiCl$ (0.482 mL, 0.400 mmol, 0.83 M in THF, 2.00 equiv.) according

to **GP1**. The diastereomeric ratio was determined by relative ^{1}HNMR peak height of the CH_2 groups after quenched with a saturated aqueous solution of NH_4Cl. The residue was purified by flash column chromatography on silica gel using cyclohexane:methyl *tert*-butyl ether = 10:1 as the eluent to afford **2p** as a yellow oil (54.8 mg, d.r = 69:31, 95% yield; in our hands, the two diastereoisomers were not separable by conventional flash chromatography on silica gel).

R$_f$ = 0.51 (cyclohexane:methyl *tert*-butyl ether = 10:1). **IR** (ATR): $\tilde{\nu}$ = 702, 736, 772, 830, 1109, 1255, 1427, 1770, 2929, 2957 cm^{-1}. **^{1}H NMR** (500 MHz, $CDCl_3$) δ = 0.37 (s, 3.2H), 0.41 (s, 2.5H), 0.43 (s, 2.8H), 0.93–0.85 (m, 8.5 H), 1.32–1.24 (m, 2.6H), 1.50–1.34 (m, 4.6H), 1.64–1.53 (m, 2.3H), 1.84–1.74 (m, 2.0H), 2.55–2.49 (m, 0.4H), 2.75–2.69 (m, 1.0H), 2.97–2.88 (m, 1.4H), 3.06–3.00 (m, 1.4H), 7.40–7.35 (m, 4.5H), 7.55–7.50 (m, 3.1H) ppm. **^{13}C NMR** (126 MHz, $CDCl_3$) δ = –4.2, –3.8, –2.9, –2.7, 14.0, 14.2, 14.7, 15.1, 15.3, 19.3, 20.5, 21.8, 22.1, 25.8, 26.8, 30.6, 35.2, 42.3, 51.1, 52.1, 65.3, 67.3, 127.8, 127.9, 129.2, 129.4, 134.2, 134.4, 137.1, 137.4, 208.5, 209.2 ppm. **^{29}Si{^{1}H} DEPT NMR** δ = –0.47, 2.08 ppm. **HRMS** (APCI) exact mass for $[M+H]^+$ $C_{18}H_{29}OSi^+$: calculated 289.1982, found 289.1983.

8. Experimental Details for Conjugate Silylation/Trapping Reactions

8.1 3-(Dimethyl(phenyl)silyl)-3-phenylcyclobut-1-en-1-yl diphenyl phosphate (3a)

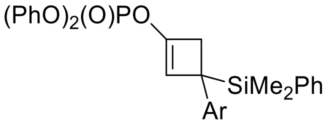

3a (Ar = Ph)
$C_{30}H_{29}O_4PSi$
M = 512.62 g/mol

Prepared from 3-phenylcyclobut-2-en-1-one (**1a**, 28.8 mg, 0.200 mmol,1.00 equiv.) and $(Me_2PhSi)_2Zn{\cdot}x$LiCl (x ⩽ 4; 0.71 mL, 0.240 mmol, 0.34 M in Et_2O, 1.20 equiv.) according to **GP2**. The residue was purified by automatic column chromatography using cyclohexane/ethyl acetate (50:1 → 10:1) to afford **3a** as a yellow oil (69.7 mg, 68%).

R$_f$ = 0.32 (cyclohexane: ethyl acetate = 10:1). **IR** (ATR): $\tilde{\nu}$ = 701, 775, 810, 961, 1011, 1184, 1215, 1302, 1488, 1591, 1624, 2958, 3068 cm^{-1}. **^{1}H NMR** (500 MHz, $CDCl_3$) δ = 0.29 (s, 3H), 0.30 (s, 3H), 2.93–2.88 (d, *J* = 14.1 Hz, 1H), 3.32–3.26 (d, *J* = 13.5 Hz, 1H), 5.70 (s, 1H), 6.90–6.83 (m, 2H), 7.15–7.09 (m, 1H), 7.24–7.17 (m, 8H), 7.30–7.27 (m, 4H), 7.37–7.32 (m, 5H) ppm. **^{13}C NMR** (126 MHz, $CDCl_3$) δ = –5.6, –5.2, 38.4, 43.5 (d, *J* = 5.9 Hz), 115.1 (d, *J* = 8.2 Hz), 120.0 (d, *J* = 4.7 Hz), 124.7, 125.6, 127.3, 127.4, 127.5, 129.2, 129.8, 134.3, 136.1, 140.3, 140.4, 143.5, 150.27 (d, *J* = 2.4 Hz), 150.33 (d, *J* = 2.3 Hz) ppm. **^{29}Si{^{1}H} DEPT NMR** δ = –0.62 ppm. **^{31}P NMR** δ = –18.27 ppm. **HRMS** (APCI) exact mass for $[M+H]^+$ $C_{30}H_{30}O_4PSi^+$: calculated 513.1645, found 513.1644.

8.2 3-(Dimethyl(phenyl)silyl)-3-(p-tolyl)cyclobut-1-en-1-yl diphenyl phosphate (3b)

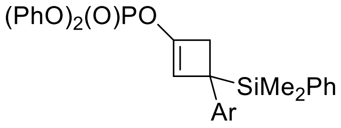

3b (Ar = 4-MeC_6H_4)
$C_{31}H_{31}O_4PSi$
M = 526.64 g/mol

Prepared from 3-(p-tolyl)cyclobut-2-en-1-one (**1b**, 31.6 mg, 0.200 mmol, 1.00 equiv.) and $(Me_2PhSi)_2Zn\cdot x LiCl$ (x ⩽ 4; 0.71 mL, 0.240 mmol, 0.34 M in Et_2O, 1.20 equiv.) according to **GP2**. The residue was purified by automatic column chromatography using cyclohexane/ethyl acetate (50:1 → 10:1) to afford **3b** as a yellow oil (87.4 mg, 83%).
R_f = 0.36 (cyclohexane: ethyl acetate = 10:1). **IR** (ATR): $\tilde{\nu}$ = 700, 772, 808, 946, 1010, 1182, 1253, 1301, 1426, 1487, 1589, 1623, 2956 cm^{-1}. **^{1}H NMR** (400 MHz, $CDCl_3$) δ = 0.279 (s, 3H), 0.284 (s, 3H), 2.31 (s, 3H), 2.90–2.83 (m, 1H), 3.29–3.24 (m, 1H), 5.68 (s, 1H), 6.79–6.74 (m, 2H), 7.03–6.99 (m, 2H), 7.23–7.17 (m, 6H), 7.28–7.26 (m, 1H), 7.30–7.28 (m, 1H), 7.36–7.30 (m, 7H) ppm. **^{13}C NMR** (101 MHz, $CDCl_3$) δ = –5.5, –5.1, 21.0, 38.0, 43.6 (d, *J* = 5.6 Hz), 115.3 (d, *J* = 7.7 Hz), 120.1 (d, *J* = 5.6 Hz), 125.6, 127.3, 127.5, 128.3, 129.2, 129.9, 134.1, 134.4, 136.3, 140.2, 140.3, 140.5, 150.3 (d, *J* = 1.9 Hz), 150.4 (d, *J* = 1.9 Hz) ppm. **^{29}Si{^{1}H} DEPT NMR** δ = –0.87 ppm. **^{31}P NMR** δ = –18.29 ppm. **HRMS** (APCI) exact mass for $[M+H]^+$ $C_{31}H_{32}O_4PSi^+$: calculated 527.1802, found 527.1801.

8.3 3-(Dimethyl(phenyl)silyl)-3-(m-tolyl)cyclobut-1-en-1-yl diphenyl phosphate (3c)

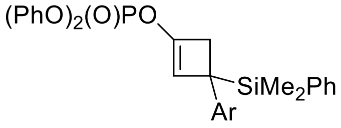

3c (Ar = 3-MeC_6H_4)
$C_{31}H_{31}O_4PSi$
M = 526.64 g/mol

Prepared from 3-(m-tolyl)cyclobut-2-en-1-one (**1c**, 31.6 mg, 0.200 mmol, 1.00 equiv.) and $(Me_2PhSi)_2Zn\cdot x LiCl$ (x ⩽ 4; 0.71 mL, 0.240 mmol, 0.34 M in Et_2O, 1.20 equiv.) according to **GP2**. The residue was purified by automatic column chromatography using cyclohexane/ethyl acetate (50:1 → 10:1) to afford **3c** as a yellow oil (86.4 mg, 82%).
R_f = 0.41 (cyclohexane: ethyl acetate = 10:1). **IR** (ATR): $\tilde{\nu}$ = 701, 732, 772, 808, 944, 1009, 1185, 1300, 1486, 1589, 1623, 2956, 3067 cm^{-1}. **^{1}H NMR** (400 MHz, $CDCl_3$) δ = 0.26 (s, 3H), 0.27 (s, 3H), 2.22 (s, 3H), 2.91–2.83 (m, 1H), 3.29–3.21 (m, 1H), 5.66 (s, 1H), 6.60–6.57 (m, 1H), 6.69–6.64 (m, 1H), 6.93–6.88 (m, 1H), 7.10–7.04 (m, 1H), 7.22–7.15 (m, 6H), 7.26–7.24 (m, 1H), 7.29–7.26 (m, 3H), 7.35–7.29 (m, 5H) ppm. **^{13}C NMR** (101 MHz, $CDCl_3$) δ = –5.6, –5.2, 21.4, 38.4, 43.5 (d, *J* = 5.6 Hz), 115.2 (d, *J* = 7.7 Hz),

120.1 (d, *J* = 4.7 Hz), 124.4, 125.4, 125.6, 127.4, 128.3, 129.2, 134.4, 136.2, 136.9, 140.3, 140.4, 143.3, 150.31 (d, *J* = 1.8 Hz), 150.38 (d, *J* = 1.8 Hz) ppm. **^{29}Si{^{1}H} DEPT NMR** δ = –0.72 ppm. **^{1}H/^{29}Si HMQC NMR** (500/99 MHz, $CDCl_3$, optimized for *J* = 7.0 Hz**)** δ = 0.22/–0.95, 0.24/–0.95, 3.22/–0.95, 7.25/–0.95 ppm. **HRMS** (APCI) exact mass for $[M+H]^+$ $C_{31}H_{31}O_4PSi^+$: calculated 527.1802, found 527.1801.

8.4 3-(Dimethyl(phenyl)silyl)-3-(4-methoxyphenyl)cyclobut-1-en-1-yl diphenyl phosphate (3d)

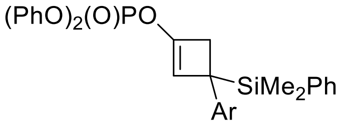

3d (Ar = 4-MeOC_6H_4)
$C_{31}H_{31}O_5PSi$
M = 542.64 g/mol

Prepared from 3-(4-methoxyphenyl)cyclobut-2-en-1-one (**1d**, 34.8 mg, 0.200 mmol, 1.00 equiv.) and $(Me_2PhSi)_2Zn \cdot x LiCl$ (x ⩽ 4; 0.77 mL, 0.240 mmol, 0.31 M in Et_2O, 1.20 equiv.) according to **GP2**. The residue was purified by automatic column chromatography using cyclohexane/ethyl acetate (50:1 → 5:1) to afford **3d** as a yellow oil (66.2 mg, 61%).

R_f = 0.32 (cyclohexane: ethyl acetate = 5:1). **IR** (ATR): $\tilde{\nu}$ = 688, 774, 810, 959, 1010, 1181, 1246, 1298, 1488, 1589, 2833, 2954, 3067 cm^{-1}. **^{1}H NMR** (500 MHz, $CDCl_3$) δ = 0.28 (s, 3H), 0.29 (s, 3H), 2.84 (d, *J* = 13.6 Hz, 1H), 3.26 (d, *J* = 13.4 Hz, 1H), 3.79 (s, 3H), 5.66 (s, 1H), 6.78–6.74 (m, 3H), 7.23–7.17 (m, 6H), 7.32–7.27 (m, 4H), 7.37–7.32 (m, 6H) ppm. **^{13}C NMR** (126 MHz, $CDCl_3$) δ = –5.5, –5.0, 37.7, 43.7 (d, *J* = 5.8 Hz), 55.4, 113.2, 115.5 (d, *J* = 8.3 Hz). 120.2 (d, *J* = 4.7 Hz), 125.7, 127.6, 128.4, 129.4, 129.9, 130.0, 134.5, 135.8, 136.4, 139.3, 140.36, 140.43, 150.4 (d, *J* = 2.3 Hz), 150.5 (d, *J* = 2.3 Hz), 157.1 ppm. **^{1}H/^{29}Si HMQC NMR** (500/99 MHz, $CDCl_3$, optimized for *J* = 7.0 Hz**)** δ = 0.28/–0.94, 0.29/–0.94, 3.26/–0.94, 7.31/–0.94 ppm. **^{31}P NMR** δ = –18.26 ppm. **HRMS** (APCI) exact mass for $[M+H]^+$ $C_{31}H_{32}O_5PSi^+$: calculated 543.1751, found 543.1754.

8.5 3-(4-Chlorophenyl)-3-(dimethyl(phenyl)silyl)cyclobut-1-en-1-yl diphenyl phosphate (3e)

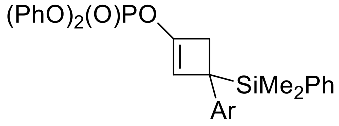

3e (Ar = 4-ClC_6H_4)
$C_{30}H_{28}ClO_4PSi$
M = 547.06 g/mol

Prepared from 3-(4-chlorophenyl)cyclobut-2-en-1-one (**1e**, 35.7 mg, 0.200 mmol, 1.00 equiv.) and $(Me_2PhSi)_2Zn{\cdot}x$LiCl (x ⩽ 4; 0.71 mL, 0.240 mmol, 0.34 M in Et_2O, 1.20 equiv.) according to **GP2**. The residue was purified by automatic column chromatography using cyclohexane/ethyl acetate (50:1 → 10:1) to afford **3e** as a yellow oil (89.7 mg, 82%).

R_f = 0.29 (cyclohexane: ethyl acetate = 5:1). **IR** (ATR): $\tilde{\nu}$ = 702, 727, 808, 905, 960, 1181, 1253, 1297, 1426, 1482, 1589, 1624, 2957, 3068 cm^{-1}. **^{1}H NMR** (500 MHz, $CDCl_3$) δ = 0.27 (s, 3H), 0.28 (s, 3H), 2.85–2.79 (d, *J* = 13.6 Hz, 1H), 3.27 (d, *J* = 13.5 Hz, 1H), 5.63 (s, 1H), 6.14 (d, *J* = 8.3 Hz, 2H), 7.14 (d, *J* = 8.5 Hz, 2H), 7.23–7.17 (m, 6H), 7.29–7.26 (m, 4H), 7.36–7.31 (m, 5H) ppm. **^{13}C NMR** (126 MHz, $CDCl_3$) δ = –5.5, –5.0, 38.5, 43.8 (d, *J* = 5.9 Hz), 115.1 (d, *J* = 8.2 Hz), 120.3 (d, *J* = 4.7 Hz), 126.0, 127.90, 127.92, 128.9, 129.7, 130.2, 130.8, 134.6, 136.0, 140.8, 140.9, 142.5, 150.56 (d, *J* = 2.4 Hz), 150.62 (d, *J* = 3.5 Hz) ppm. **^{29}Si{^{1}H} DEPT NMR** δ = –0.52 ppm. **^{31}P NMR** δ = –18.25 ppm. **HRMS** (APCI) exact mass for $[M+H]^+$ $C_{31}H_{29}ClO_4PSi^+$: calculated 547.1256, found 547.1257.

8.6 3-(Dimethyl(phenyl)silyl)-3-(4-fluorophenyl)cyclobut-1-en-1-yl diphenyl phosphate (3f)

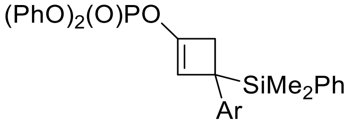

3f (Ar = 4-FC_6H_4)
$C_{30}H_{28}FO_4PSi$
M = 530.61 g/mol

Prepared from 3-(4-fluorophenyl)cyclobut-2-en-1-one (**1f**, 32.4 mg, 0.200 mmol, 1.00 equiv.) and $(Me_2PhSi)_2Zn{\cdot}x$LiCl (x ⩽ 4; 0.71 mL, 0.240 mmol, 0.34 M in Et_2O, 1.20 equiv.) according to **GP2**. The residue was purified by automatic column chromatography using cyclohexane/ethyl acetate (50:1 → 10:1) to afford **3f** as a yellow oil (84.9 mg, 80%).

R_f = 0.31 (cyclohexane: ethyl acetate = 10:1). **IR** (ATR): $\tilde{\nu}$ = 727, 809, 905, 962, 1011, 1180, 1215, 1297, 1488, 1590, 2958, 3068 cm^{-1}. **^{1}H NMR** (500 MHz, $CDCl_3$) δ = 0.24 (s, 3H), 0.25 (s, 3H), 2.80 (d, *J* = 13.7 Hz, 1H), 3.24 (d, *J* = 13.4 Hz, 1H), 5.61 (s, 1H), 6.77–6.71 (m, 2H), 6.87–6.80 (m, 2H), 7.20–7.14 (m, 6H), 7.25–7.22 (m, 4H), 7.33–7.29 (m, 5H) ppm. **^{13}C NMR** (126 MHz, $CDCl_3$) δ = –5.4, –5.0, 38.2, 43.9 (d, *J* = 5.9 Hz), 114.6 (d, *J* = 21.1 Hz), 115.3 (d, *J* = 8.2 Hz), 120.4 (d, *J* = 4.7 Hz), 126.0, 127.9, 128.9, 129.0, 129.7, 130.2, 134.6, 136.22, 139.6 (d, *J* = 2.4 Hz), 140.8 (d, *J* = 10.6 Hz), 150.59 (d, *J* = 2.3 Hz), 150.64 (d, *J* = 2.1 Hz), 160.8 (d, *J* = 243.3 Hz) ppm. **^{29}Si{^{1}H} DEPT NMR** δ = –0.60 ppm. **^{31}P NMR** δ = –18.25 ppm. **^{19}F NMR** δ = –118.7 ppm. **HRMS** (APCI) exact mass for $[M+H]^+$ $C_{31}H_{29}FO_4PSi^+$: calculated 531.1551, found 531.1550.

8.7 3-Butyl-3-(dimethyl(phenyl)silyl)cyclobut-1-en-1-yl diphenyl phosphate (3g)

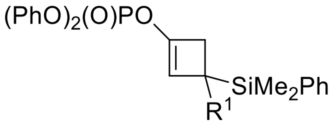

3g (R^1 = *n*-C_4H_9)
$C_{28}H_{33}O_4PSi$
M = 492.63 g/mol

Prepared from 3-butylcyclobut-2-en-1-one (**1g**, 24.8 mg, 0.200 mmol, 1.00 equiv.) and $(Me_2PhSi)_2Zn \cdot x LiCl$ (x ⩽ 4; 0.77 mL, 0.240 mmol, 0.31 M in Et_2O, 1.20 equiv.) according to **GP2**. The residue was purified by automatic column chromatography using cyclohexane/ethyl acetate (50:1 → 5:1) to afford **3g** as a yellow oil (47.3 mg, 48%).
R_f = 0.42 (cyclohexane: ethyl acetate = 5:1). **IR** (ATR): $\tilde{\nu}$ = 700, 769, 809, 944, 1185, 1251, 1301, 1487, 1589, 2926, 2954 cm^{-1}. **^{1}H NMR** (500 MHz, $CDCl_3$) δ = 0.291 (s, 3H), 0.296 (s, 3H), 0.81 (t, *J* = 6.7 Hz, 3H), 1.22–1.14 (m, 4H), 1.47–1.38 (m, 1H), 1.59–1.50 (m, 1H), 2.42 (d, *J* = 13.8 Hz, 1H), 2.76 (d, *J* = 13.9 Hz, 1H), 5.29 (s, 1H), 7.25–7.15 (m, 6H), 7.37–7.31 (m, 7H), 7.51–7.47 (m, 2H) ppm. **^{13}C NMR** (126 MHz, $CDCl_3$) δ = –5.0, –4.7, 14.0, 23.4, 27.9, 31.5, 34.2, 40.2 (d, *J* = 5.9 Hz), 117.0 (d, *J* = 8.3 Hz), 120.0 (d, *J* = 5.7 Hz), 125.5, 127.7, 129.0, 129.8, 134.0, 137.6, 138.4 (d, *J* = 10.6 Hz), 150.4 (d, *J* = 8.2 Hz) ppm. **^{29}Si{^{1}H} DEPT NMR** δ = –0.60 ppm. **^{31}P NMR** δ = –18.23 ppm. **HRMS** (APCI) exact mass for $[M+H]^+$ $C_{28}H_{34}O_4PSi^+$: calculated 493.1958, found 493.1961.

8.8 3-(Dimethyl(phenyl)silyl)-3-hexylcyclobut-1-en-1-yl diphenyl phosphate (3h)

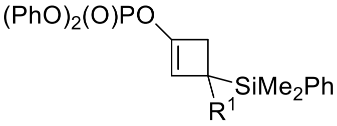

3h (R^1 = *n*-C_6H_{13})
$C_{30}H_{37}O_4PSi$
M = 520.68 g/mol

Prepared from 3-hexylcyclobut-2-en-1-one (**1h**, 30.4 mg, 0.200 mmol, 1.00 equiv.) and $(Me_2PhSi)_2Zn \cdot x LiCl$ (x ⩽ 4; 0.77 mL, 0.240 mmol, 0.31 M in Et_2O, 1.20 equiv.) according to **GP2**. The residue was purified by automatic column chromatography using cyclohexane/ethyl acetate (50:1 → 5:1) to afford **3h** as a yellow oil (51.0 mg, 49%).
R_f = 0.39 (cyclohexane: ethyl acetate = 5:1). **IR** (ATR): $\tilde{\nu}$ = 701, 771, 810, 947, 1186, 1252, 1488, 1590, 2852, 2924, 2953 cm^{-1}. **^{1}H NMR** (500 MHz, $CDCl_3$) δ = 0.29 (s, 3H), 0.30 (s, 3H), 0.84 (t, *J* = 6.9 Hz, 3H), 1.23–1.13 (m, 8H), 1.45–1.38 (m, 1H), 1.57–1.49 (m, 1H), 2.42 (d, *J* = 13.8 Hz, 1H), 2.77 (d, *J* = 13.7 Hz, 1H), 5.29 (s, 1H), 7.25–7.18 (m, 6H), 7.38–7.31 (m, 7H), 7.51–7.46 (m, 2H) ppm. **^{13}C NMR** (126 MHz, $CDCl_3$) δ = –4.9, –4.7, 14.1, 22.6, 25.2, 30.0, 31.6, 31.8, 34.6, 40.2 (d, *J* = 4.7 Hz), 117.0 (d, *J* = 7.1 Hz), 120.0 (d, *J* = 5.9 Hz), 125.5, 127.7, 129.0, 129.8, 134.0, 137.7, 138.4 (d, *J* = 10.6 Hz),

150.4 (d, J = 7.0 Hz) ppm. **^{29}Si{^{1}H} DEPT NMR** δ = –0.62 ppm. **^{31}P NMR** δ = –18.22 ppm. **HRMS** (APCI) exact mass for $[M+H]^+$ $C_{30}H_{38}O_4PSi^+$: calculated 521.2271, found 521.2269.

8.9 3-(Dimethyl(phenyl)silyl)-3-phenethylcyclobut-1-en-1-yl diphenyl phosphate (3i)

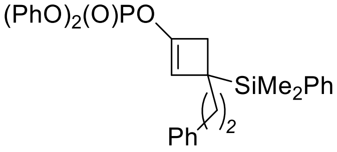

3i
$C_{32}H_{33}O_4PSi$
M = 540.67 g/mol

Prepared from 3-phenethylcyclobut-2-en-1-one (**1i**, 34.4 mg, 0.200 mmol, 1.00 equiv.) and $(Me_2PhSi)_2Zn\cdot x LiCl$ (x ⩽ 4; 0.77 mL, 0.240 mmol, 0.31 M in Et_2O, 1.20 equiv.) according to **GP2**. The residue was purified by automatic column chromatography using cyclohexane/ethyl acetate (50:1 → 5:1) to afford **3i** as a yellow oil (58.4 mg, 54%).
R_f = 0.35 (cyclohexane: ethyl acetate = 10:1). **IR** (ATR): $\tilde{\nu}$ = 700, 726, 772, 810, 905, 963, 1185, 1296, 1488, 1590, 3066 cm^{-1}. **^{1}H NMR** (500 MHz, $CDCl_3$) δ = 0.27 (s, 6H), 1.73–1.63 (m, 1H), 1.86–1.77 (m, 1H), 2.42 (t, J = 8.6 Hz, 2H), 2.47 (d, J = 14.1 Hz, 1H), 2.78 (d, J = 13.8 Hz, 1H), 5.28 (s, 1H), 7.00–6.95 (m, 2H), 7.11–7.06 (m, 1H), 7.20–7.14 (m, 8H), 7.33–7.26 (m, 7H), 7.47–7.43 (m, 2H) ppm. **^{13}C NMR** (126 MHz, $CDCl_3$) δ = –5.0, –4.8, 31.7, 32.1, 36.5, 40.1 (d, J = 4.7 Hz), 116.6 (d, J = 8.2 Hz), 120.0 (d, J = 4.7 Hz), 125.60, 125.67, 127.8, 128.2, 128.3, 129.2, 129.9, 134.0, 137.3, 138.7 (d, J = 11.1 Hz), 142.8, 150.4 (d, J = 7.1 Hz) ppm. **^{29}Si{^{1}H} DEPT NMR** δ = –0.52 ppm. **^{31}P NMR** δ = –18.20 ppm. **HRMS** (APCI) exact mass for $[M+H]^+$ $C_{32}H_{34}O_4PSi^+$: calculated 541.1958, found 541.1960.

8.10 3-(Dimethyl(phenyl)silyl)-3-(3-phenylpropyl)cyclobut-1-en-1-yl diphenyl phosphate (3j)

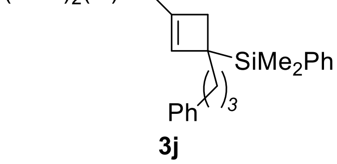

3j
$C_{33}H_{35}O_4PSi$
M = 554.70 g/mol

Prepared from 3-(3-phenylpropyl)cyclobut-2-en-1-one (**1j**, 37.3 mg, 0.200 mmol, 1.00 equiv.) and $(Me_2PhSi)_2Zn\cdot x LiCl$ (x ⩽ 4; 0.77 mL, 0.240 mmol, 0.31 M in Et_2O, 1.20 equiv.) according to **GP2**. The residue was purified by automatic column chromato-

graphy using cyclohexane/ethyl acetate (50:1 → 5:1) to afford **3j** as a yellow oil (51.0 mg, 46%).

R$_f$ = 0.38 (cyclohexane: ethyl acetate = 10:1). **IR** (ATR): $\tilde{\nu}$ = 700, 772, 811, 961, 1188, 1489, 1590, 2854, 2933, 3024 cm^{-1}. **^{1}H NMR** (500 MHz, $CDCl_3$) δ = 0.19 (s, 6H), 1.53–1.40 (m, 4H), 2.32 (d, *J* = 13.8 Hz, 1H), 2.39 (t, *J* = 7.2 Hz, 2H), 2.67 (d, *J* = 13.8 Hz, 1H), 5.18 (s, 1H), 6.97 (d, *J* = 7.5 Hz, 2H), 7.12–7.08 (m, 6H), 7.18–7.12 (m, 3H), 7.25–7.20 (m, 7H), 7.37 (d, *J* = 5.9 Hz, 2H) ppm. **^{13}C NMR** (126 MHz, $CDCl_3$) δ = –5.0, –4.8, 27.6, 31.5, 34.0, 36.5, 40.2 (d, *J* = 5.9 Hz), 116.7 (d, *J* = 8.3 Hz), 120.0 (d, *J* = 5.1 Hz), 125.5, 125.7, 127.7, 128.2, 128.3, 129.1, 129.8, 134.0, 137.5, 138.5 (d, *J* = 10.6 Hz), 142.4, 150.4 (d, *J* = 7.0 Hz) ppm. **^{29}Si{^{1}H} DEPT NMR** δ = –0.59 ppm. **^{31}P NMR** δ = –18.24 ppm. **HRMS** (APCI) exact mass for $[M+H]^+$ $C_{33}H_{36}O_4PSi^+$: calculated 555.2115, found 555.2105.

8.11 3-(4-Chlorobutyl)-3-(dimethyl(phenyl)silyl)cyclobut-1-en-1-yl diphenyl phosphate (3k)

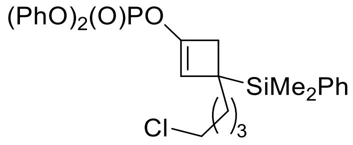

3k

$C_{28}H_{32}ClO_4PSi$

M = 527.07 g/mol

Prepared from 3-(4-chlorobutyl)cyclobut-2-en-1-one (**1k**, 31.7 mg, 0.200 mmol, 1.00 equiv.) and $(Me_2PhSi)_2Zn\cdot x LiCl$ (x ⩽ 4; 0.77 mL, 0.240 mmol, 0.31 M in Et_2O, 1.20 equiv.) according to **GP2**. The residue was purified by automatic column chromatography using cyclohexane/ethyl acetate (50:1 → 5:1) to afford **3k** as a yellow oil (53.8 mg, 51%).

R$_f$ = 0.34 (cyclohexane: ethyl acetate = 5:1). **IR** (ATR): $\tilde{\nu}$ = 688, 734, 772, 960, 1187, 1301, 1488, 1590, 1621, 2953, 3068 cm^{-1}. **^{1}H NMR** (500 MHz, $CDCl_3$) δ = 0.30 (s, 6H), 1.37–1.29 (m, 2H), 1.48–1.40 (m, 1H), 1.69–1.55 (m, 3H), 2.43 (d, *J* = 14.0 Hz, 1H), 2.79 (d, *J* = 13.9 Hz, 1H), 3.41 (t, *J* = 6.6 Hz, 2H), 5.28 (s, 1H), 7.25–7.19 (m, 6H), 7.38–7.32 (m, 7H), 7.51–7.46 (m, 2H) ppm. **^{13}C NMR** (126 MHz, $CDCl_3$) δ = –5.1, –4.8, 23.0, 31.4, 33.2, 33.6, 40.1 (d, *J* = 5.9 Hz), 44.8, 116.6 (d, *J* = 8.3 Hz), 120.0 (d, *J* = 5.3 Hz), 125.6, 127.8, 129.1, 129.9, 134.0, 137.4, 138.6 (d, *J* = 9.5 Hz), 150.4 (d, *J* = 7.1 Hz) ppm. **^{29}Si{^{1}H} DEPT NMR** δ = –0.54 ppm. **^{31}P NMR** δ = –18.23 ppm. **HRMS** (APCI) exact mass for $[M+H]^+$ $C_{28}H_{33}ClO_4PSi^+$: calculated 527.1569, found 527.1567.

8.12 3-Cyclopropyl-3-(dimethyl(phenyl)silyl)cyclobut-1-en-1-yl diphenyl phos phate (3l)

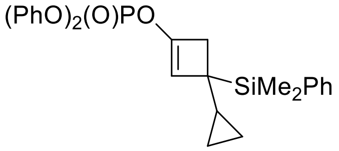

3l
$C_{27}H_{29}O_4PSi$
M = 476.58 g/mol

Prepared from 3-cyclopropylcyclobut-2-en-1-one (**1l**, 21.6 mg, 0.200 mmol, 1.00 equiv.) and $(Me_2PhSi)_2Zn{\cdot}x$LiCl (x ⩽ 4; 0.77 mL, 0.240 mmol, 0.31 M in Et_2O, 1.20 equiv.) according to **GP2**. The residue was purified by automatic column chromatography using cyclohexane/ethyl acetate (50:1 → 5:1) to afford **3l** as a yellow oil (49.6 mg, 52%).

$\mathbf{R_f}$ = 0.39 (cyclohexane: ethyl acetate = 5:1). **IR** (ATR): $\tilde{\nu}$ = 688, 773, 811, 962, 1009, 1188, 1311, 1489, 1590, 2956, 2997, 3069 cm^{-1}. ^{1}**H NMR** (500 MHz, $CDCl_3$) δ = 0.00—0.08 (m, 1H), 0.15–0.07 (m, 1H), 0.24–0.18 (m, 1H), 0.32 (s, 3H), 0.35 (s, 3H), 0.41–0.36 (m, 1H), 0.96–0.88 (m, 1H), 2.32 (d, *J* = 13.5 Hz, 1H), 2.79 (d, *J* = 13.6 Hz, 1H), 4.97 (s, 1H), 7.24–7.17 (m, 6H), 7.39–7.31 (m, 7H), 7.57–7.50 (m, 2H) ppm. ^{13}**C NMR** (126 MHz, $CDCl_3$) δ = –3.8, –3.6, 0.0, 4.2, 15.2, 33.0, 42.0 (d, *J* = 4.7 Hz), 113.7 (d, *J* = 8.2 Hz), 121.4 (d, *J* = 4.6 Hz), 127.0, 129.1, 130.5, 131.3, 135.6, 138.9, 141.4 (d, *J* = 9.5 Hz), 151.8 (d, *J* = 7.0 Hz) ppm. ^{29}**Si{^{1}H} DEPT NMR** δ = –0.15 ppm. ^{31}**P NMR** δ = –18.31 ppm. **HRMS** (APCI) exact mass for $[M+H]^+$ $C_{27}H_{30}O_4PSi^+$: calculated 477.1645, found 477.1643.

8.13 3-Cyclohexyl-3-(dimethyl(phenyl)silyl)cyclobut-1-en-1-yl diphenyl phos-phate (3m)

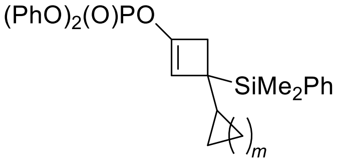

3m (*m* = 4)
$C_{30}H_{35}O_4PSi$
M = 518.66 g/mol

Prepared from 3-cyclohexylcyclobut-2-en-1-one (**1m**, 30.0 mg, 0.200 mmol, 1.00 equiv.) and $(Me_2PhSi)_2Zn{\cdot}x$LiCl (x ⩽ 4; 0.77 mL, 0.240 mmol, 0.31 M in Et_2O, 1.20 equiv.) according to **GP2**. The residue was purified by automatic column chromatography using cyclohexane/ethyl acetate (50:1 → 5:1) to afford **3m** as a yellow oil (49.8 mg, 48%).

$\mathbf{R_f}$ = 0.39 (cyclohexane: ethyl acetate = 5:1). **IR** (ATR): $\tilde{\nu}$ = 687, 770, 810, 960, 1186, 1254, 1301, 1488, 1590, 1622, 2849, 2923, 3067 cm^{-1}. ^{1}**H NMR** (500 MHz, $CDCl_3$) δ = 0.32 (s, 3H), 0.33 (s, 3H), 1.12–0.85 (m, 5H), 1.42–1.33 (m, 1H), 1.65–1.55 (m, 5H), 2.53 (d, *J* = 14.0 Hz, 1H), 2.72 (d, *J* = 13.9 Hz, 1H), 5.35 (s, 1H), 7.24–7.17 (m, 6H),

7.38–7.30 (m, 7H), 7.52–7.47 (m, 2H) ppm. **^{13}C NMR** (126 MHz, $CDCl_3$) δ = –3.1, –2.6, 26.4, 26.85, 26.89, 30.3, 30.6, 36.4, 39.0 (d, *J* = 5.9 Hz), 44.7, 117.1 (d, *J* = 8.3 Hz), 120.0 (d, *J* = 4.7 Hz), 125.5, 127.6, 128.9, 129.8, 134.0, 138.7, 139.2 (d, *J* = 9.4 Hz), 150.4 (d, *J* = 7.0 Hz) ppm. **^{29}Si{^{1}H} DEPT NMR** δ = –1.41 ppm. **^{31}P NMR** δ = –18.23 ppm. **HRMS** (APCI) exact mass for $[M+H]^+$ $C_{30}H_{36}O_4PSi^+$: calculated 519.2115, found 519.2114.

8.14 3-(Dimethyl(phenyl)silyl)-3-(trimethylsilyl)cyclobut-1-en-1-yl diphenyl phosphate (3n)

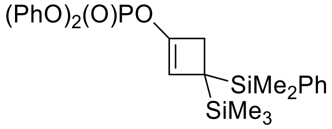

3n
$C_{27}H_{33}O_4PSi_2$
M = 508.70 g/mol

Prepared from 3-(trimethylsilyl)cyclobut-2-en-1-one (**1n**, 28.1 mg, 0.200 mmol, 1.00 equiv.) and $(Me_2PhSi)_2Zn\cdot x LiCl$ (x ⩽ 4; 0.77 mL, 0.240 mmol, 0.31 M in Et_2O, 1.20 equiv.) according to **GP2**. The residue was purified by automatic column chromatography using cyclohexane/ethyl acetate (50:1 → 5:1) to afford **3n** as a yellow oil (49.8 mg, 48%).

R$_f$ = 0.46 (cyclohexane: ethyl acetate = 5:1). **IR** (ATR): $\tilde{\nu}$ = 725, 904, 945, 1180, 1252, 1295, 1488, 1590, 2955, 3069 cm^{-1}. **^{1}H NMR** (500 MHz, $CDCl_3$) δ = –0.14 (s, 9H), 0.31 (s, 3H), 0.33 (s, 3H), 2.79–2.67 (m, 2H), 5.36 (s, 1H), 7.23–7.19 (m, 5H), 7.36–7.30 (m, 7H), 7.45–7.40 (m, 1H), 7.52–7.46 (m, 2H) ppm. **^{13}C NMR** (126 MHz, $CDCl_3$) δ = –1.8, –1.6, 0.0, 23.2, 39.5 (d, *J* = 5.9 Hz), 118.7 (d, *J* = 8.2 Hz), 122.5 (d, *J* = 4.7 Hz), 127.5, 128.5, 129.6, 131.1, 131.9, 132.1, 136.1, 138.6 (d, *J* = 10.6 Hz), 140.2, 152.4 (d, *J* = 7.1 Hz) ppm. **^{29}Si{^{1}H} DEPT NMR** δ = –1.39, 4.54 ppm. **^{31}P NMR** δ = –18.29 ppm. **HRMS** (APCI) exact mass for $[M+H]^+$ $C_{27}H_{34}O_4PSi_2^+$: calculated 509.1728, found 509.1729.

8.15 3-(Dimethyl(phenyl)silyl)-2-methyl-3-phenylcyclobut-1-en-1-yl diphenyl phosphate (3o)

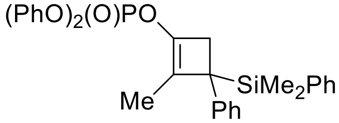

3o
$C_{31}H_{31}O_4PSi$
M = 526.64 g/mol

Prepared from 2-methyl-3-phenylcyclobut-2-en-1-one (**1o**, 31.6 mg, 0.200 mmol, 1.00 equiv.) and $(Me_2PhSi)_2Zn\cdot x LiCl$ (x ⩽ 4; 0.77 mL, 0.240 mmol, 0.31 M in Et_2O, 1.20 equiv.) according to **GP2**. The residue was purified by automatic column chromato-

graphy using cyclohexane/ethyl acetate (50:1 → 5:1) to afford **3o** as a yellow oil (62.1 mg, 59%).

R$_f$ = 0.13 (cyclohexane: ethyl acetate = 20:1). **IR** (ATR): $\tilde{\nu}$ = 701, 774, 817, 951, 1009, 1088, 1186, 1487, 1590, 2918, 2956, 3066 cm^{-1}. **^{1}H NMR** (500 MHz, $CDCl_3$) δ = 0.29 (s, 6H), 1.89 (s, 3H), 2.88 (d, *J* = 13.2 Hz, 1H), 3.23 (d, *J* = 13.2 Hz, 1H), 6.93 (d, *J* = 7.6 Hz, 2H), 7.13–7.08 (m, 1H), 7.21–7.14 (m, 8H), 7.25–7.23 (m, 4H), 7.33–7.27 (m, 5H) ppm. **^{13}C NMR** (126 MHz, $CDCl_3$) δ = –4.8, –4.4, 12.6, 41.3, 42.5 (d, *J* = 2.3 Hz), 120.1 (d, *J* = 5.8 Hz), 124.9, 125.5, 126.2 (d, *J* = 10.6 Hz), 127.5 (d, *J* = 2.3 Hz), 127.6, 129.0, 129.2, 129.8, 134.1 (d, *J* = 9.4 Hz), 134.4, 136.6, 143.7, 150.4 (d, *J* = 7.0 Hz) ppm. **^{29}Si{^{1}H} DEPT NMR** δ = –0.71 ppm. **^{31}P NMR** δ = –17.90 ppm. **HRMS** (APCI) exact mass for [M+H]$^+$ $C_{31}H_{32}O_4PSi^+$: calculated 527.1802, found 527.1793.

8.16 3-(Dimethyl(phenyl)silyl)-2,3-dipropylcyclobut-1-en-1-yl diphenyl phos-phate (3p)

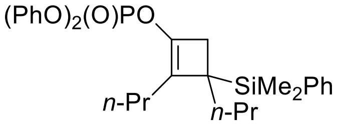

2p
$C_{30}H_{37}O_4PSi$
M = 520.68 g/mol

Prepared from 2,3-dipropylcyclobut-2-en-1-one (**1p**, 30.4 mg, 0.200 mmol, 1.00 equiv.) and $(Me_2PhSi)_2Zn\cdot x LiCl$ (x ⩽ 4; 0.77 mL, 0.240 mmol, 0.31 M in Et_2O, 1.20 equiv.) according to **GP2**. The residue was purified by automatic column chromatography using cyclohexane/ethyl acetate (50:1 → 5:1) to afford **3p** as a yellow oil (39.6 mg, 38%).

R$_f$ = 0.20 (cyclohexane: ethyl acetate = 20:1). **IR** (ATR): $\tilde{\nu}$ = 701, 770, 813, 943, 1008, 1186, 1488, 1590, 2870, 2955 cm^{-1}. **^{1}H NMR** (500 MHz, $CDCl_3$) δ = 0.27 (s, 3H), 0.33 (s, 3H), 0.87–0.81 (m, 6H), 1.29–1.14 (m, 3H), 1.50–1.43 (m, 3H), 1.86–1.77 (m, 1H), 1.99–1.89 (m, 1H), 2.47 (d, *J* = 13.5 Hz, 1H), 2.75 (d, *J* = 13.2 Hz, 1H), 7.25–7.20 (m, 6H), 7.37–7.31 (m, 7H), 7.54–7.48 (m, 2H) ppm. **^{13}C NMR** (126 MHz, $CDCl_3$) δ = –4.6, –4.4, 14.5, 14.8, 17.9, 20.1, 29.3, 34.0, 34.6, 37.7, 120.0 (d, *J* = 2.3 Hz), 120.1 (d, *J* = 2.4 Hz), 125.4, 127.7, 129.0, 129.8, 130.8 (d, *J* = 9.6 Hz), 131.2 (d, *J* = 9.4 Hz), 133.9, 134.1, 137.9, 150.5 (d, *J* = 7.1 Hz) ppm. **^{29}Si{^{1}H} DEPT NMR** δ = –0.87 ppm. **^{31}P NMR** δ = –17.93 ppm. **HRMS** (APCI) exact mass for [M+H]$^+$ $C_{30}H_{38}O_4PSi^+$: calculated 521.2271, found 521.2262.

9. Experimental Details for Kumada Coupling of Cyclobutenyl Phosphates

9.1 (1,3-Diphenylcyclobut-2-en-1-yl)dimethyl(phenyl)silane (4aa)

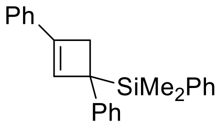

4aa
$C_{24}H_{24}Si$
M = 340.54 g/mol

Prepared from 3-(dimethyl(phenyl)silyl)-3-phenylcyclobut-1-en-1-yl diphenyl phosphate (**3a**, 51.3 mg, 0.100 mmol, 1.00 equiv.) and PhMgBr (0.067 mL, 0.200 mmol, 3.0 M in Et_2O, 2.00 equiv.) according to **GP3**. The residue was purified by flash column chromatography on silica gel using cyclohexane:methyl *tert*-butyl ether = 50:1 as the eluent to afford **4aa** as a yellow oil (24.5 mg, 72% yield).
R_f = 0.41 (cyclohexane:methyl *tert*-butyl ether = 50:1). **IR** (ATR): $\tilde{\nu}$ = 699, 751, 775, 811, 1112, 1249, 1487, 1595 cm^{-1}. **^1H NMR** (500 MHz, $CDCl_3$) δ = 0.29 (s, 3H), 0.31 (s, 3H), 2.94 (d, *J* = 13.2 Hz, 1H), 3.31 (d, *J* = 13.2 Hz, 1H), 6.76 (s, 1H), 6.92 (d, *J* = 7.2 Hz, 2H), 7.06 (t, *J* = 7.3 Hz, 1H), 7.20–7.16 (m, 2H), 7.25–7.21 (m, 1H), 7.37–7.27 (m, 9H) ppm. **^{13}C NMR** (126 MHz, $CDCl_3$) δ = –5.6, –5.1, 39.4, 44.1, 124.09, 124.12, 126.9, 127.3, 127.41, 127.44, 128.3, 129.0, 131.0, 134.4, 134.6, 136.8, 143.8, 144.6 ppm. **$^{29}Si\{^1H\}$ DEPT NMR** δ = –1.24 ppm. **HRMS** (APCI) exact mass for $[M+H]^+$ $C_{24}H_{25}Si^+$: calculated 341.1720, found 341.1722.

9.2 (1-Butyl-3-phenylcyclobut-2-en-1-yl)dimethyl(phenyl)silane (4ga)

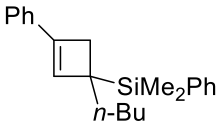

4ga
$C_{22}H_{28}Si$
M = 320.55 g/mol

Prepared from 3-butyl-3-(dimethyl(phenyl)silyl)cyclobut-1-en-1-yl diphenyl phosphate (**3g**, 49.3 mg, 0.100 mmol, 1.00 equiv.) and PhMgBr (0.067 mL, 0.200 mmol, 3.0 M in Et_2O, 2.00 equiv.) according to **GP3**. The residue was purified by flash column chromatography on silica gel using cyclohexane:methyl *tert*-butyl ether = 50:1 as the eluent to afford **4ga** as a yellow oil (19.2 mg, 60% yield).
R_f = 0.28 (cyclohexane). **IR** (ATR): $\tilde{\nu}$ = 697, 750, 771, 812, 1110, 1249, 1426, 1487, 2925, 2954 cm^{-1}. **^1H NMR** (500 MHz, $CDCl_3$) δ = 0.30 (s, 6H), 0.81 (t, *J* = 7.1 Hz, 3H), 1.29–1.16 (m, 5H), 1.66–1.58 (m, 1H), 2.48 (d, *J* = 13.4 Hz, 1H), 2.78 (d, *J* = 13.4 Hz, 1H), 6.44 (s, 1H), 7.23–7.19 (m, 1H), 7.36–7.30 (m, 7H), 7.57–7.53 (m, 2H) ppm. **^{13}C NMR** (126 MHz, $CDCl_3$) δ = –4.9, –4.7, 14.0, 23.5, 28.1, 34.1, 35.1, 37.5, 123.9, 127.0, 127.6, 128.3, 128.8, 133.7, 134.1, 135.0, 138.5, 142.6 ppm. **$^{29}Si\{^1H\}$ DEPT NMR** δ = –

1.34 ppm. **HRMS** (APCI) exact mass for $[M+H]^+$ $C_{22}H_{29}Si^+$: calculated 321.2033, found 321.2036.

9.3 Dimethyl(phenyl)(3-phenyl-1-(3-phenylpropyl)cyclobut-2-en-1-yl)silane (4ja)

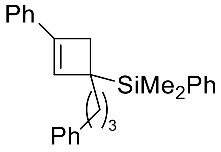

4ja
$C_{27}H_{30}Si$
M = 382.62 g/mol

Prepared from 3-(dimethyl(phenyl)silyl)-3-(3-phenylpropyl)cyclobut-1-en-1-yl diphenyl phosphate (**3j**, 36.1 mg, 0.0650 mmol, 1.00 equiv.) and PhMgBr (0.043 mL, 0.0975 mmol, 3.0 M in Et_2O, 2.00 equiv.) according to **GP3**. The residue was purified by flash column chromatography on silica gel using cyclohexane:methyl *tert*-butyl ether = 50:1 as the eluent to afford **4ja** as a yellow oil (12.9 mg, 52% yield).

R$_f$ = 0.34 (cyclohexane:methyl *tert*-butyl ether = 50:1). **IR** (ATR): $\tilde{\nu}$ = 698, 750, 813, 1110, 1249, 1426, 1490, 2852, 2930, 3024 cm^{-1}. **^{1}H NMR** (500 MHz, $CDCl_3$) δ = 0.29 (s, 6H), 1.63–1.56 (m, 3H), 1.73–1.65 (m, 1H), 2.53–2.45 (m, 3H), 2.78 (d, *J* = 13.2 Hz, 1H), 6.42 (s, 1H), 7.07 (d, *J* = 7.7 Hz, 2H), 7.14 (t, *J* = 7.2 Hz, 1H), 7.24–7.20 (m, 3H), 7.32–7.29 (m, 4H), 7.37–7.34 (m, 3H), 7.55–7.51 (m, 2H) ppm. **^{13}C NMR** (126 MHz, $CDCl_3$) δ = –5.0, –4.8, 27.8, 34.1, 35.1, 36.7, 37.4, 124.0, 125.6, 127.1, 127.7, 128.19, 128.25, 128.34, 128.9, 133.4, 134.1, 134.8, 138.3, 142.7, 142.8 ppm. **^{29}Si{^{1}H} DEPT NMR** δ = –1.29 ppm. **HRMS** (APCI) exact mass for $[M+H]^+$ $C_{27}H_{31}Si^+$: calculated 383.2190, found 383.2184.

9.4 (1-(4-Chlorobutyl)-3-phenylcyclobut-2-en-1-yl)dimethyl(phenyl)silane (4ka)

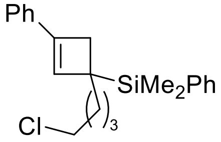

4ka
$C_{22}H_{27}ClSi$
M = 354.99 g/mol

Prepared from 3-(4-chlorobutyl)-3-(dimethyl(phenyl)silyl)cyclobut-1-en-1-yl diphenyl phosphate (**3k**, 52.7 mg, 0.100 mmol, 1.00 equiv.) and PhMgBr (0.067 mL, 0.200 mmol, 3.0 M in Et_2O, 2.00 equiv.) according to **GP3**. The residue was purified by flash column chromatography on silica gel using cyclohexane:methyl *tert*-butyl ether = 50:1 as the eluent to afford **4ka** as a yellow oil (19.9 mg, 56% yield).

R$_f$ = 0.22 (cyclohexane). **IR** (ATR): $\tilde{\nu}$ = 697, 751, 812, 1110, 1249, 1426, 1445, 1487, 2837, 2933 cm^{-1}. **^{1}H NMR** (500 MHz, $CDCl_3$) δ = 0.30 (s, 6H), 1.45–1.36 (m, 2H), 1.58–

1.50 (m, 1H), 1.71–1.60 (m, 3H), 2.48 (d, *J* = 13.4 Hz, 1H), 2.80 (d, *J* = 13.4 Hz, 1H), 3.43 (t, *J* = 6.8 Hz, 2H), 6.43 (s, 1H), 7.25–7.20 (m, 1H), 7.34–7.28 (m, 4H), 7.37–7.34 (m, 3H), 7.57–7.51 (m, 2H) ppm. **^{13}C NMR** (126 MHz, $CDCl_3$) δ = –5.0, –4.9, 23.2, 33.4, 33.6, 35.0, 37.4, 44.9, 124.0, 127.2, 127.7, 128.3, 128.9, 133.2, 134.0, 134.8, 138.2, 142.9 ppm. **^{29}Si{^{1}H} DEPT NMR** δ = –1.24 ppm. **HRMS** (APCI) exact mass for $[M+H]^+$ $C_{22}H_{28}ClSi^+$: calculated 355.1643, found 355.1638.

9.5 (3-Hexyl-1-phenylcyclobut-2-en-1-yl)dimethyl(phenyl)silane (4ab)

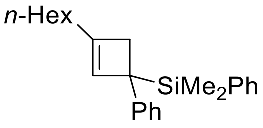

4ab
$C_{24}H_{32}Si$
M = 348.61 g/mol

Prepared from 3-(dimethyl(phenyl)silyl)-3-phenylcyclobut-1-en-1-yl diphenyl phosphate (**3a**, 51.3 mg, 0.100 mmol, 1.00 equiv.) and PhMgBr (0.067 mL, 0.200 mmol, 3.0 M in Et_2O, 2.00 equiv.) according to **GP3**. The residue was purified by flash column chromatography on silica gel using cyclohexane as the eluent to afford **4ab** as a yellow oil (8.37 mg, 24% yield).

R_f = 0.28 (cyclohexane). **IR** (ATR): $\tilde{\nu}$ = 699, 734, 772, 810, 830, 1112, 1249, 1427, 1487, 1596, 2854, 2925, 2955 cm^{-1}. **^{1}H NMR** (500 MHz, $CDCl_3$) δ = 0.24 (s, 6H), 0.88 (t, *J* = 6.9 Hz, 3H), 1.28–1.20 (m, 8H), 1.96–1.85 (m, 2H), 2.52 (d, *J* = 13.4 Hz, 1H), 2.86 (d, *J* = 13.3 Hz, 1H), 6.14 (s, 1H), 6.87 (d, *J* = 7.6 Hz, 2H), 7.03 (t, *J* = 7.4 Hz, 1H), 7.16 (t, *J* = 7.7 Hz, 2H), 7.29–7.22 (m, 2H), 7.34–7.29 (m, 3H) ppm. **^{13}C NMR** (126 MHz, $CDCl_3$) δ = –5.6, –5.2, 14.1, 22.6, 26.9, 29.2, 30.8, 31.7, 41.1, 43.8, 123.8, 126.8, 127.2, 127.3, 128.8, 130.3, 134.4, 137.2, 145.3, 148.3 ppm. **^{29}Si{^{1}H} DEPT NMR** δ = –1.62 ppm. **HRMS** (APCI) exact mass for $[M+H]^+$ $C_{24}H_{33}Si^+$: calculated 349.2346, found 349.2347.

9.6 (3-Cyclohexyl-1-phenylcyclobut-2-en-1-yl)dimethyl(phenyl)silane (4ac)

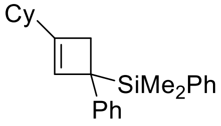

4ac
$C_{24}H_{30}Si$
M = 346.59 g/mol

Prepared from 3-(dimethyl(phenyl)silyl)-3-phenylcyclobut-1-en-1-yl diphenyl phosphate (**3a**, 51.3 mg, 0.100 mmol, 1.00 equiv.) and PhMgBr (0.067 mL, 0.200 mmol, 3.0 M in Et_2O, 2.00 equiv.) according to **GP3**. The residue was purified by flash column chromatography on silica gel using cyclohexane as the eluent to afford **4ac** as a yellow oil (17.0 mg, 49% yield).

R_f = 0.26 (*n*-pentane). **IR** (ATR): $\tilde{\nu}$ = 698, 772, 810, 1110, 1248, 1445, 1488, 1595, 2849, 2921 cm^{-1}. **^{1}H NMR** (500 MHz, $CDCl_3$) δ = 0.24 (s, 6H), 0.92–0.87 (m, 1H), 1.24–1.03 (m, 4H), 1.71–1.58 (m, 5H), 1.91–1.79 (m, 1H), 2.50 (d, *J* = 13.2 Hz, 1H), 2.88 (d, *J* = 13.2 Hz, 1H), 6.10 (s, 1H), 6.89 (d, *J* = 7.7 Hz, 2H), 7.04 (t, *J* = 7.3 Hz, 1H), 7.17 (t, *J* = 7.5 Hz, 2H), 7.30–7.26 (m, 2H), 7.35–7.30 (m, 3H) ppm. **^{13}C NMR** (126 MHz, $CDCl_3$) δ = –5.5, –5.2, 25.77, 25.84, 26.3, 30.3, 30.5, 38.8, 39.8, 43.3, 123.8, 126.8, 127.2, 127.3, 128.3, 128.8, 134.5, 137.2, 145.4, 152.4 ppm. **^{29}Si{^{1}H} DEPT NMR** δ = –1.57 ppm. **HRMS** (APCI) exact mass for $[M+H]^+$ $C_{24}H_{31}Si^+$: calculated 347.2190, found 347.2183.

10. 1.0 mmol Scale Reactions

10.1 Synthesis of 2g on 1.0 mmol scale

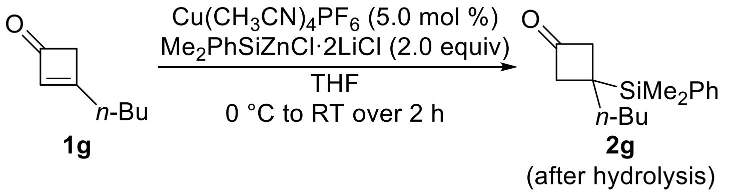

An oven-dried 50-mL Schlenk tube equipped with a magnetic stir bar was charged with $Cu(CH_3CN)_4PF_6$ (18.7 mg, 5.00 mol%) and 3-butylcyclobut-2-en-1-one (**1g**, 124 mg, 1.00 mmol, 1.00 equiv.). The tube was evacuated under high vacuum and backfilled with nitrogen (3 times). THF (15 mL) was added to the tube, and the resulting suspension was stirred under room temperature for 5 min. The mixture was then cooled to 0 °C, and a solution of $Me_2PhSiZnCl{\cdot}2LiCl$ (2.41 mL, 2.00 mmol, 0.83 M in THF, 2.00 equiv.) was added dropwise under a static pressure of nitrogen gas, and then the mixture was stirred under room temperature for 2 h. After the indicated reaction time, the reaction mixture was quenched with a saturated aqueous solution of NH_4Cl, and extracted with Et_2O. The combined organic phase was washed with brine, dried over anhydrous Na_2SO_4, and concentrated under reduced pressure. The residue was purified by flash column chromatography on silica gel using cyclohexane:methyl *tert*-butyl ether = 10:1 as the eluent to afford **2g** as a colorless oil (224 mg, 86%).

10.2 Synthesis of 3a on 1.5 mmol scale

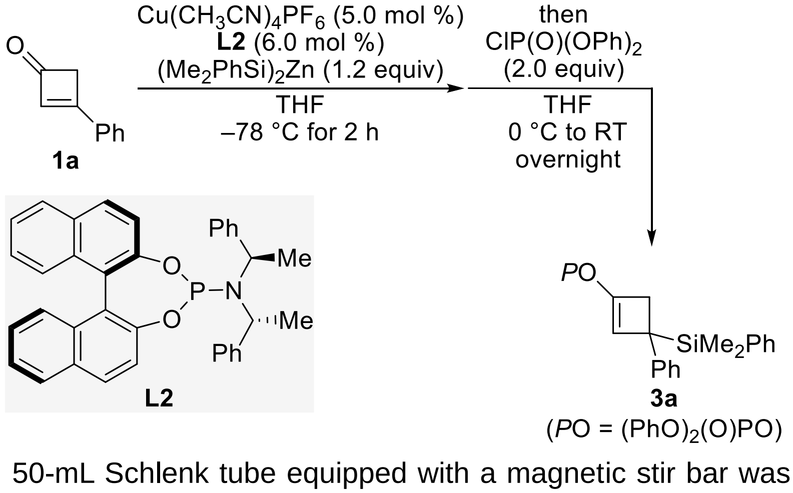

An oven-dried 50-mL Schlenk tube equipped with a magnetic stir bar was charged with $Cu(CH_3CN)_4PF_6$ (28.0 mg, 5.00 mol%), **L2** (48.6 mg, 6.00 mol%). The tube was evacuated under high vacuum and backfilled with nitrogen (3 times). THF (15 mL) was added to the tube, and the resulting suspension was stirred under room temperature for 30 min before 3-phenylcyclobut-2-en-1-one (**1a**, 216 mg, 1.50 mmol, 1.00 equiv.) were added. The mixture was then cooled to –78 °C in a dry ice/acetone bath, and a solution of $(Me_2PhSi)_2Zn{\cdot}x LiCl$ ($x \leqslant 4$; 5.80 mL, 1.80 mmol, 1.20 equiv., ~0.31 M in Et_2O) was

added dropwise under a static pressure of nitrogen gas and the mixture was stirred at –78 °C for 2 h. After the indicated reaction time, $ClPO(OPh)_2$ (0.620 mL, 806 mg, 3.00 mmol, 2.00 equiv.) and THF (5 mL) were added at –78 °C. The reaction mixture was then warmed to 0 °C and stirred at room temperature overnight. The reaction mixture was quenched with a saturated aqueous solution of NH_4Cl, and extracted with Et_2O. The combined organic phase was washed with brine, dried over anhydrous Na_2SO_4, and concentrated under reduced pressure, and the residue was purified by automatic column chromatography using cyclohexane/ethyl acetate (50:1 → 10:1) to afford **3a** as a yellow oil (454 mg, 59%).

10.3 Synthesis of 4aa on 1.0 mmol scale

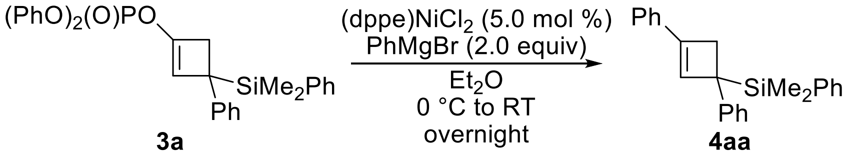

3a **4aa**

An oven-dried 50-mL Schlenk tube equipped with a magnetic stir bar was charged with $NiCl_2$(dppe) (26.4 mg, 5.00 mol%). The tube was evacuated under high vacuum and backfilled with nitrogen (3 times). Et_2O (15 mL) was added to the tube, and the resulting suspension was then cooled to 0°C with an ice bath. A solution of cyclobutenyl phosphates **3a** (512 mg, 1.00 mmol, 1.00 equiv.) in Et_2O was added to the mixture and PhMgX (0.667 mL, 2.00 equiv., 3.0 M in Et_2O, 2.00 equiv.) was added dropwise by syringe under a static pressure of nitrogen gas. The mixture was stirred under room temperature overnight. After the indicated reaction time, the reaction mixture was quenched with a saturated aqueous solution of NH_4Cl, and extracted with Et_2O. The combined organic phase was washed with brine, dried over anhydrous Na_2SO_4, and concentrated under reduced pressure. And the residue was purified by flash column chromatography on silica gel using cyclohexane:methyl *tert*-butyl ether = 50:1 as the eluent to afford **4aa** as a yellow oil (160 mg, 47% yield).

11. NMR Spectra

Figure S1. ^{1}H NMR spectrum (500 MHz, $CDCl_3$, 298 K) of 3-(Dimethyl(phenyl)silyl)-3-phenylcyclobutan-1-one (**2a**)

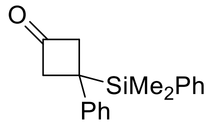

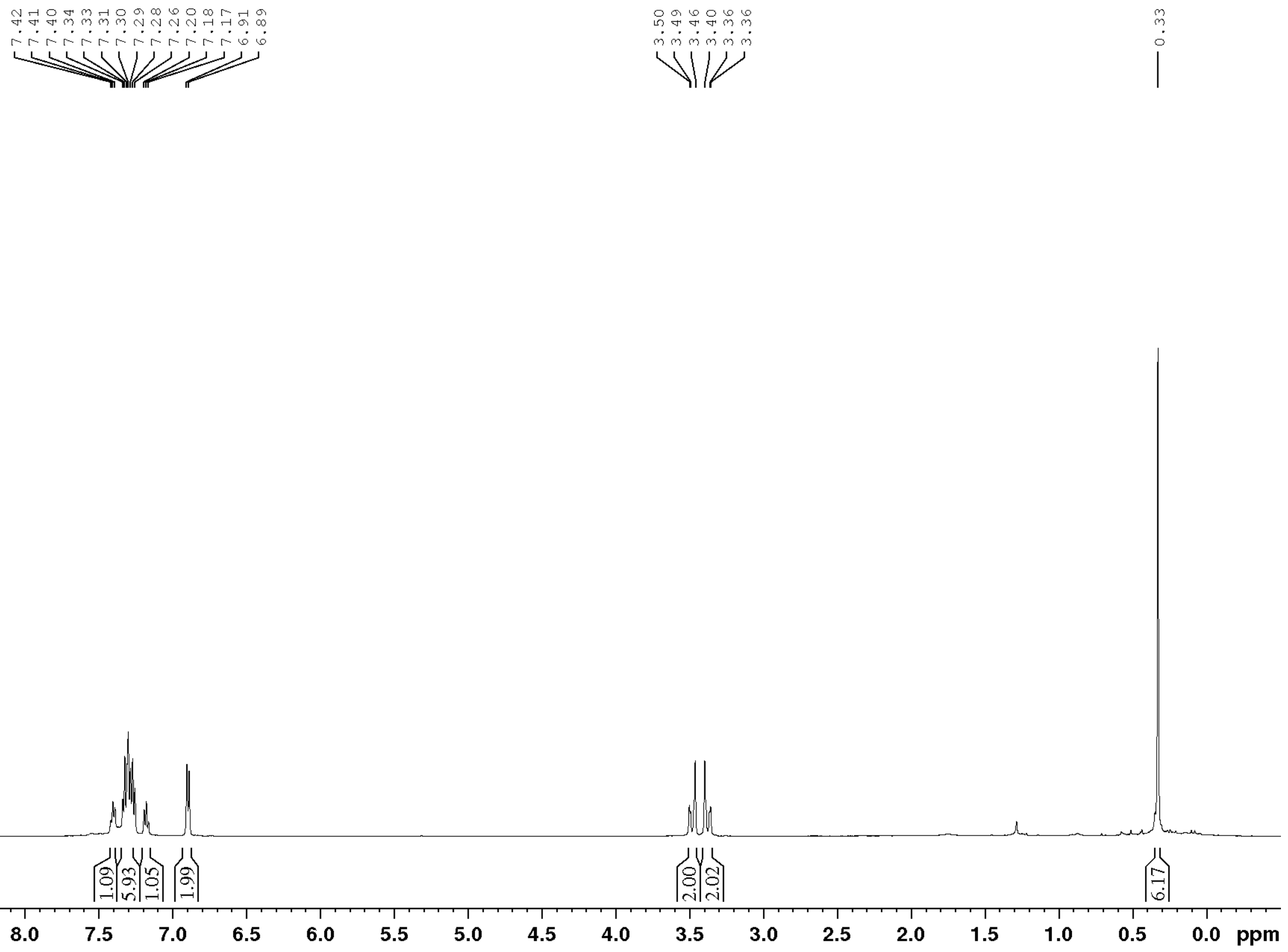

Figure S2. $^{13}C\{^1H\}$ NMR spectrum (126 MHz, $CDCl_3$, 298 K) of 3-(Dimethyl(phenyl)silyl)-3-phenylcyclobutan-1-one (**2a**)

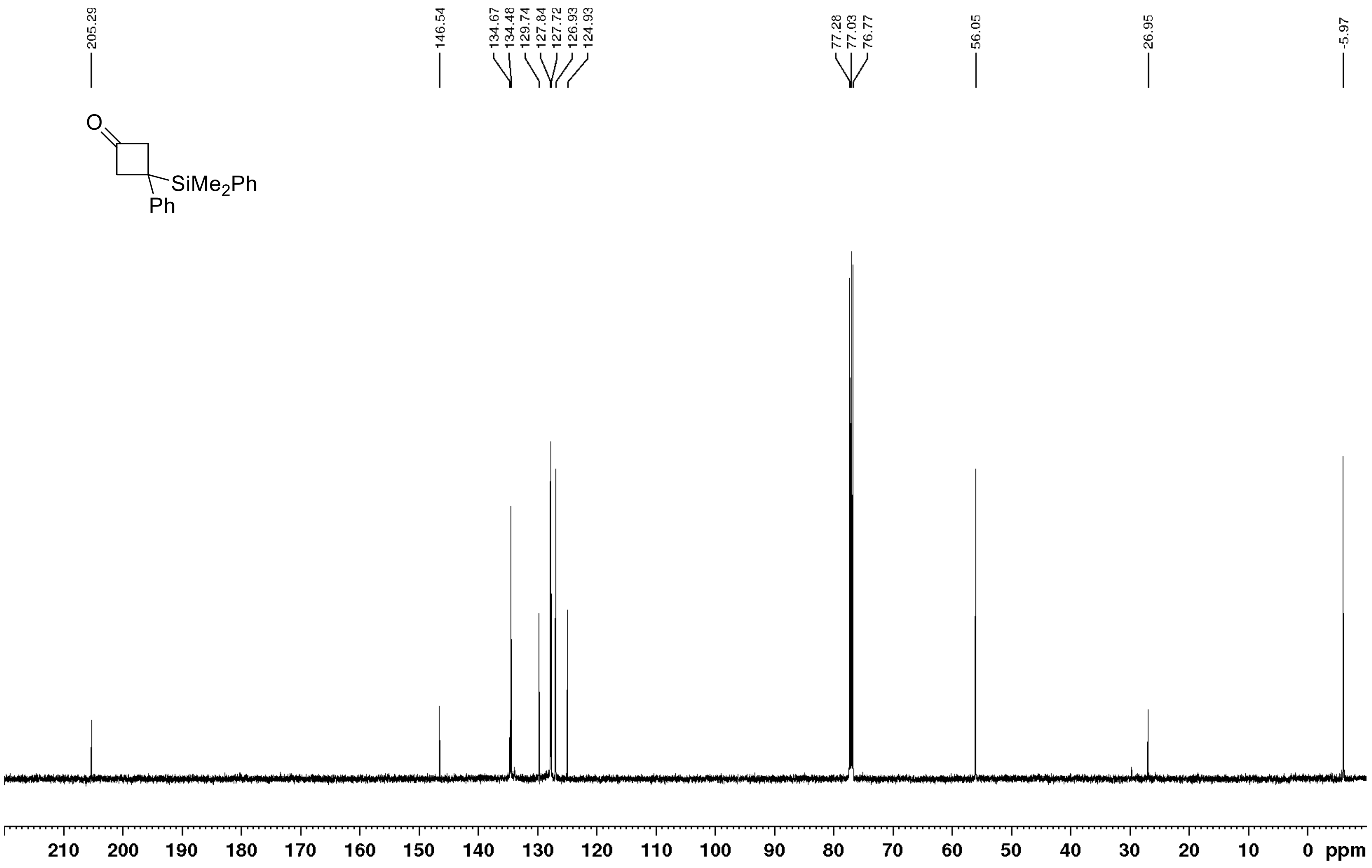

Figure S3. ^{29}Si {^{1}H} DEPT NMR spectrum (99 MHz, $CDCl_3$) of 3-(Dimethyl(phenyl)silyl)-3-phenylcyclobutan-1-one (**2a**)

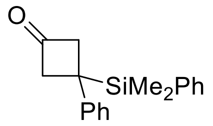

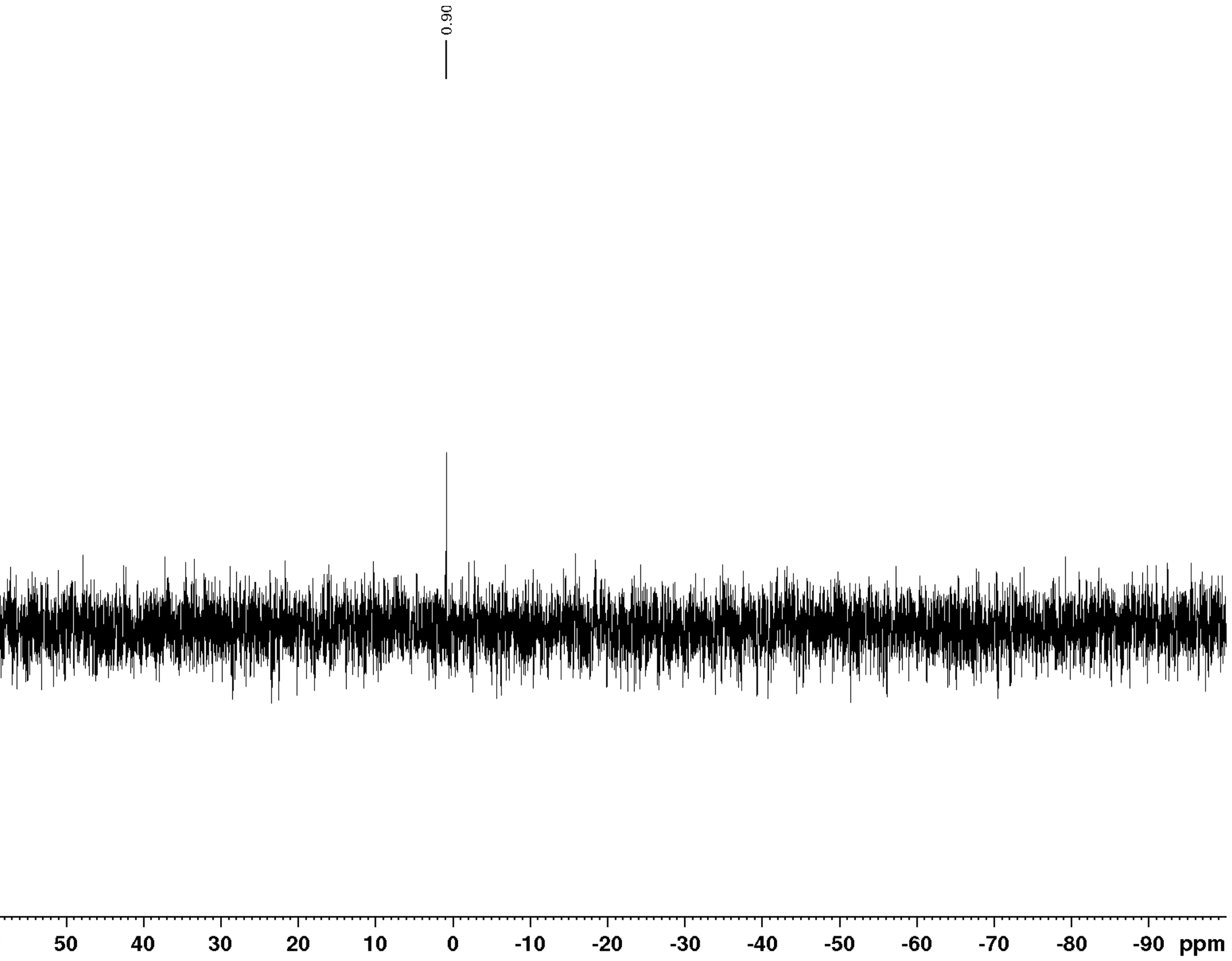

Figure S4. ^{1}H NMR spectrum (500 MHz, $CDCl_3$, 298 K) of 3-(Dimethyl(phenyl)silyl)-3-(p-tolyl)cyclobutan-1-one (**2b**) (# = Cyclohexane)

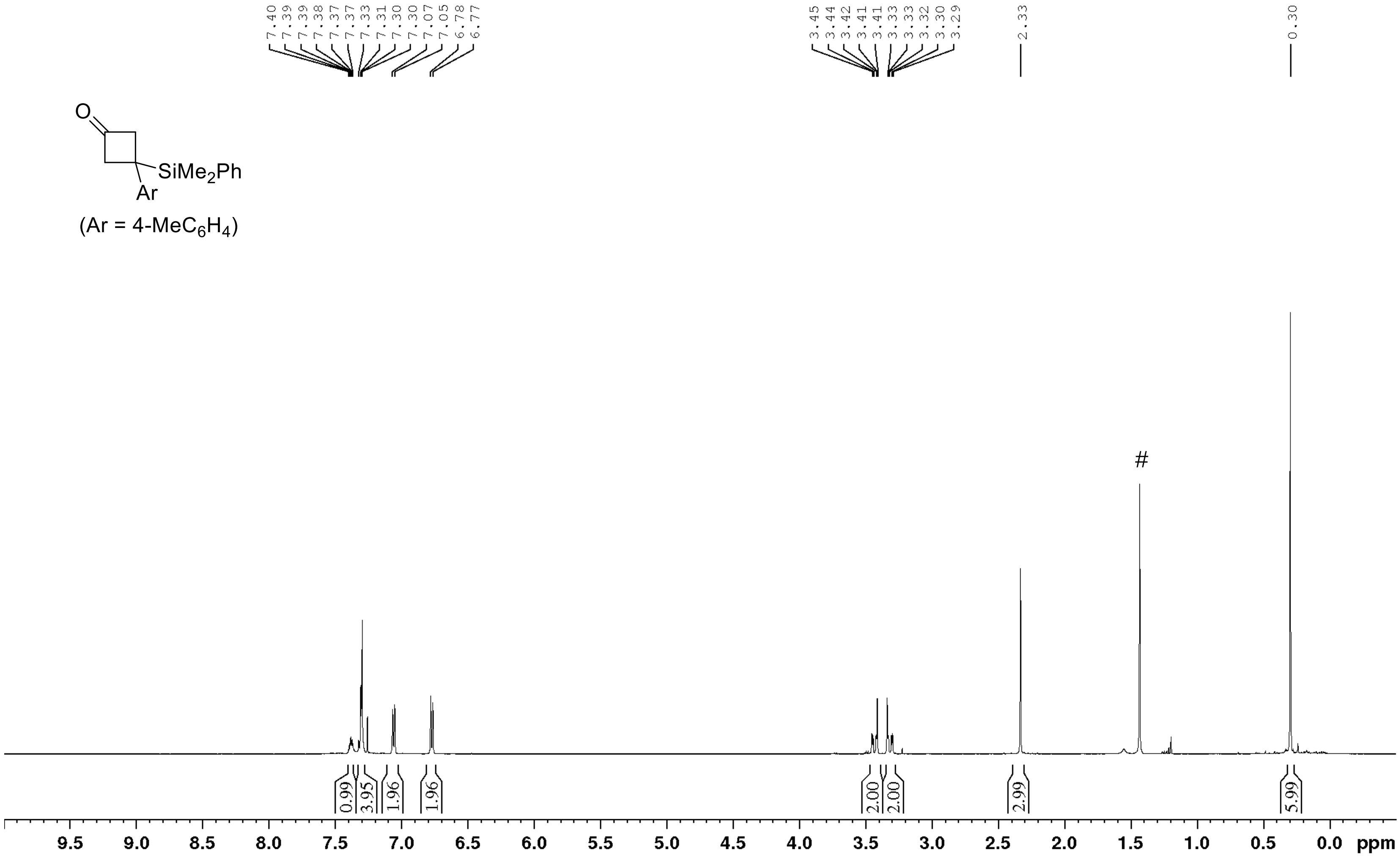

Figure S5. $^{13}C\{^{1}H\}$ NMR (126 MHz, $CDCl_3$, 298 K) of 3-(Dimethyl(phenyl)silyl)-3-(p-tolyl)cyclobutan-1-one (**2b**) (# = Cyclohexane)

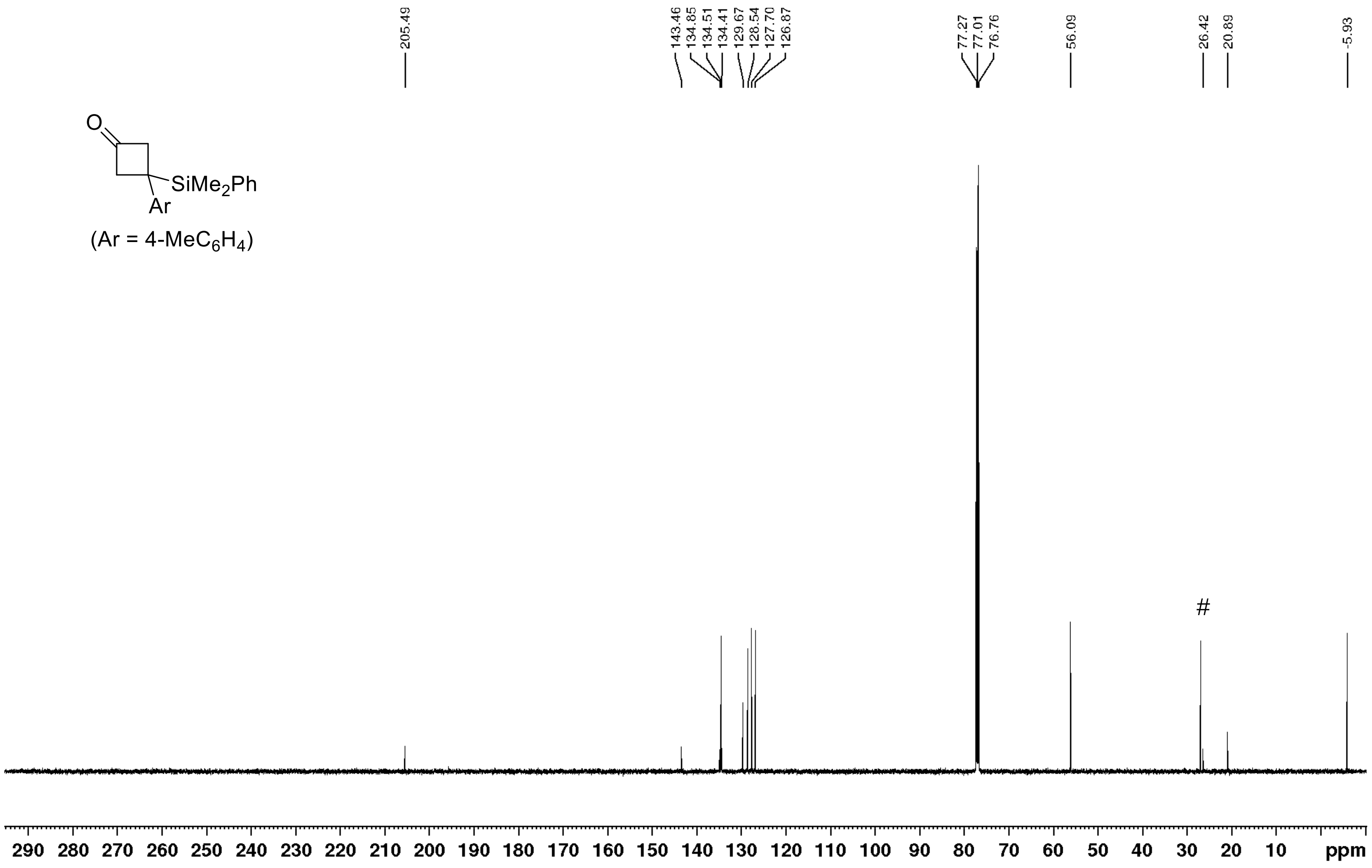

Figure S6. $^{29}Si\ \{^{1}H\}$ DEPT NMR spectrum (99 MHz, $CDCl_3$) of 3-(Dimethyl(phenyl)silyl)-3-(p-tolyl)cyclobutan-1-one (**2b**)

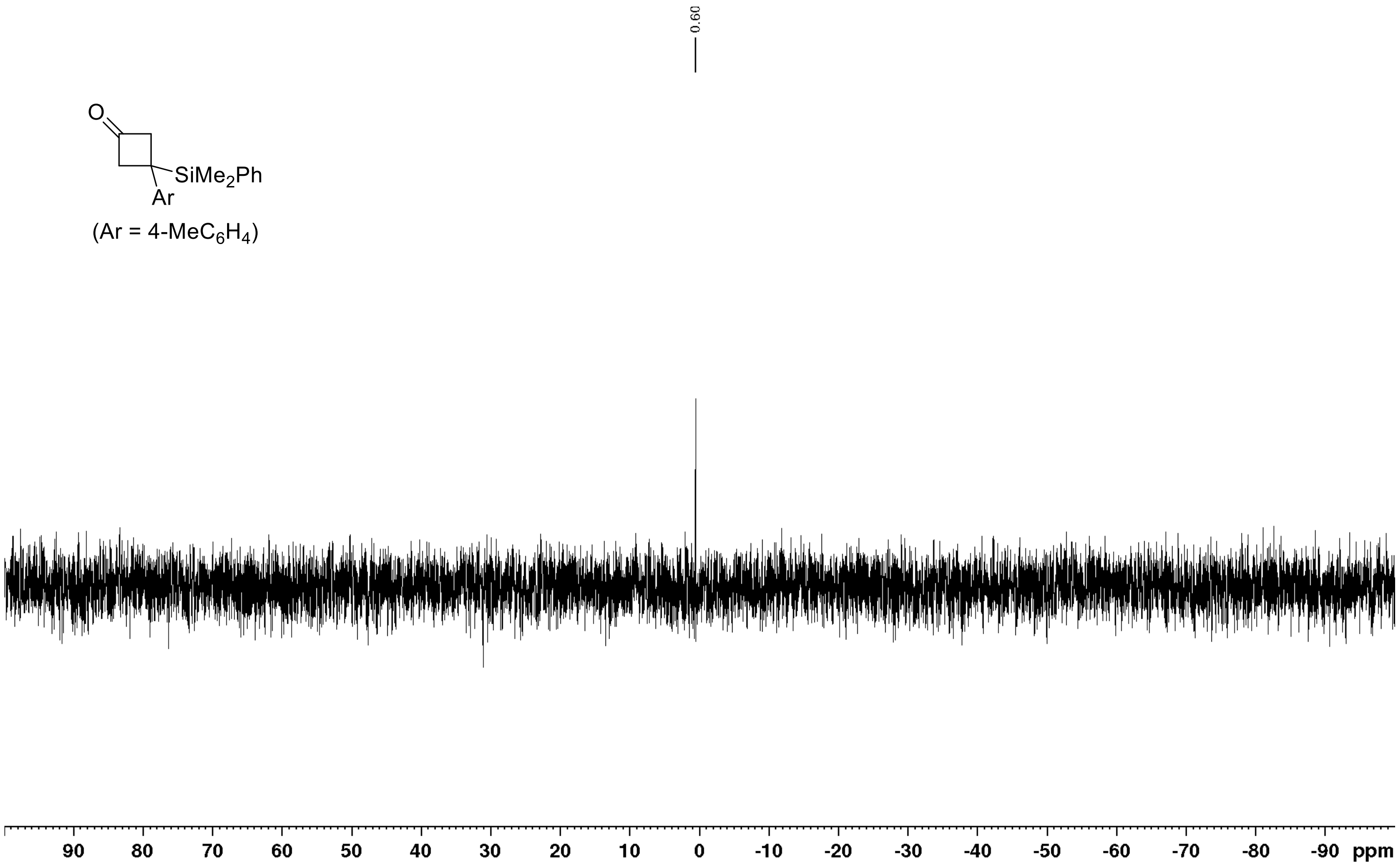

Figure S7. ^{1}H NMR (500 MHz, $CDCl_3$, 298 K) of 3-(Dimethyl(phenyl)silyl)-3-(m-tolyl)cyclobutan-1-one (**2c**)

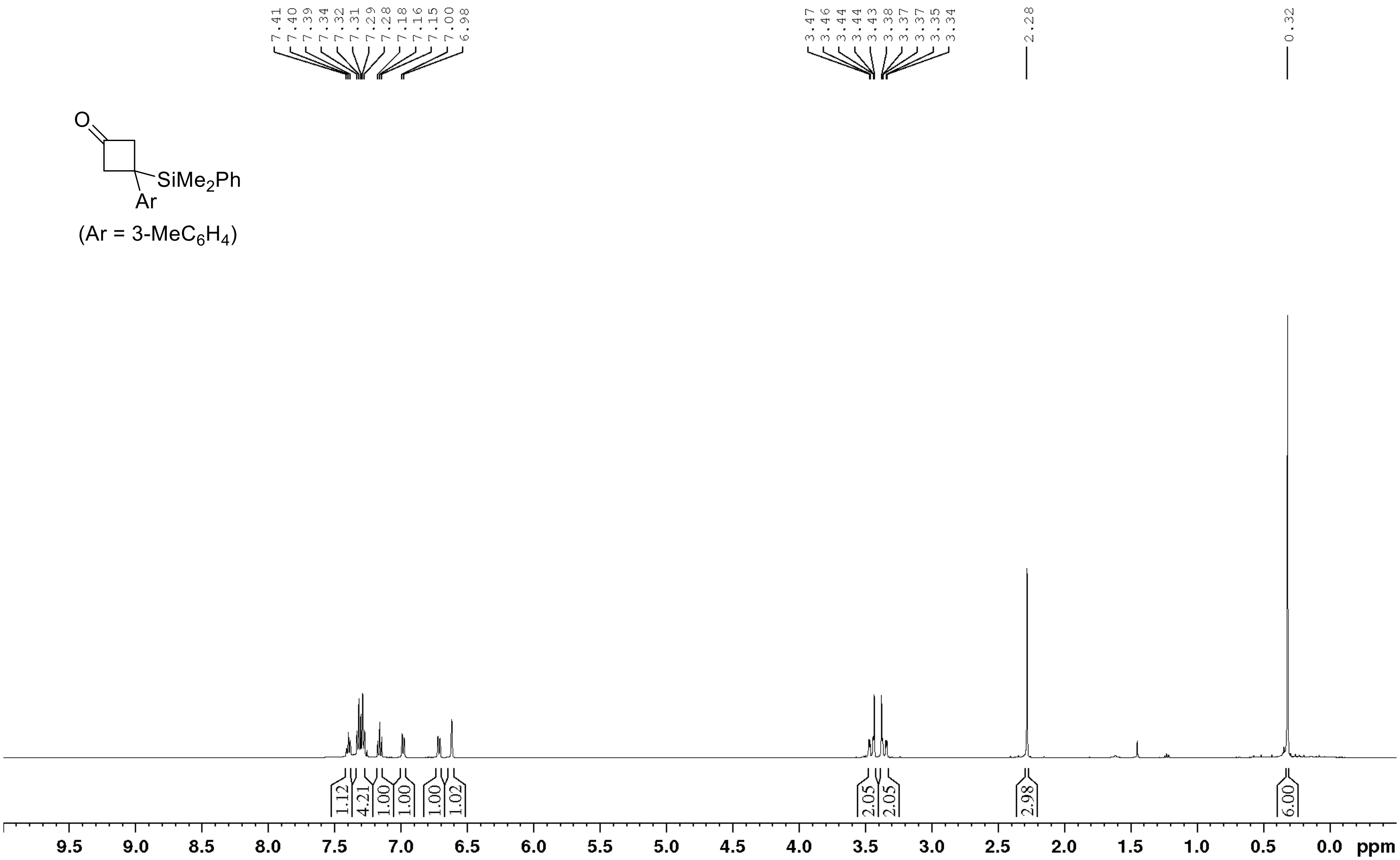

Figure S8. $^{13}C\{^1H\}$ NMR (126 MHz, $CDCl_3$, 298 K) of 3-(Dimethyl(phenyl)silyl)-3-(m-tolyl)cyclobutan-1-one (**2c**)

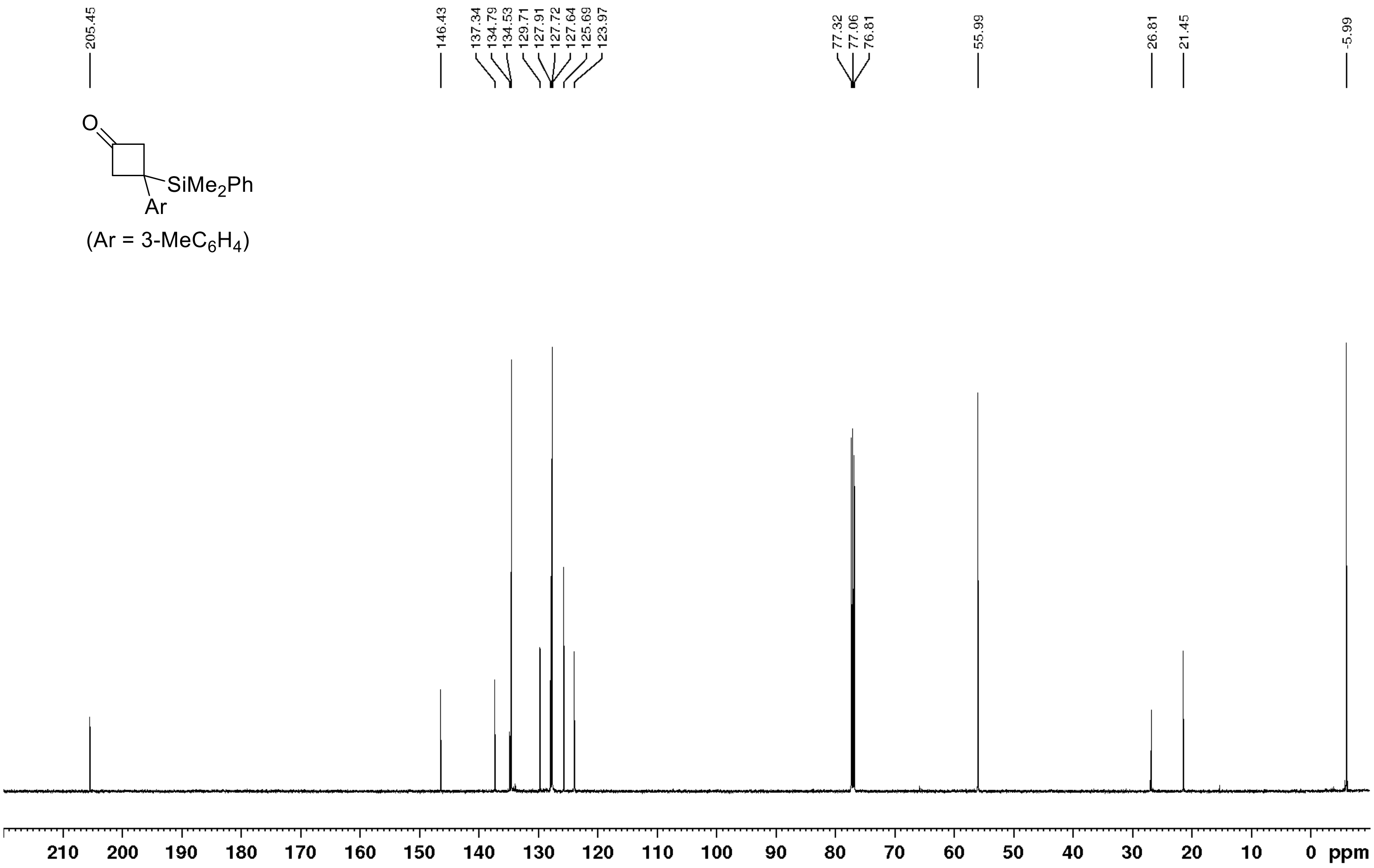

Figure S9. $^{29}Si\{^1H\}$ DEPT NMR (99 MHz, $CDCl_3$, 298 K) of 3-(Dimethyl(phenyl)silyl)-3-(m-tolyl)cyclobutan-1-one (**2c**)

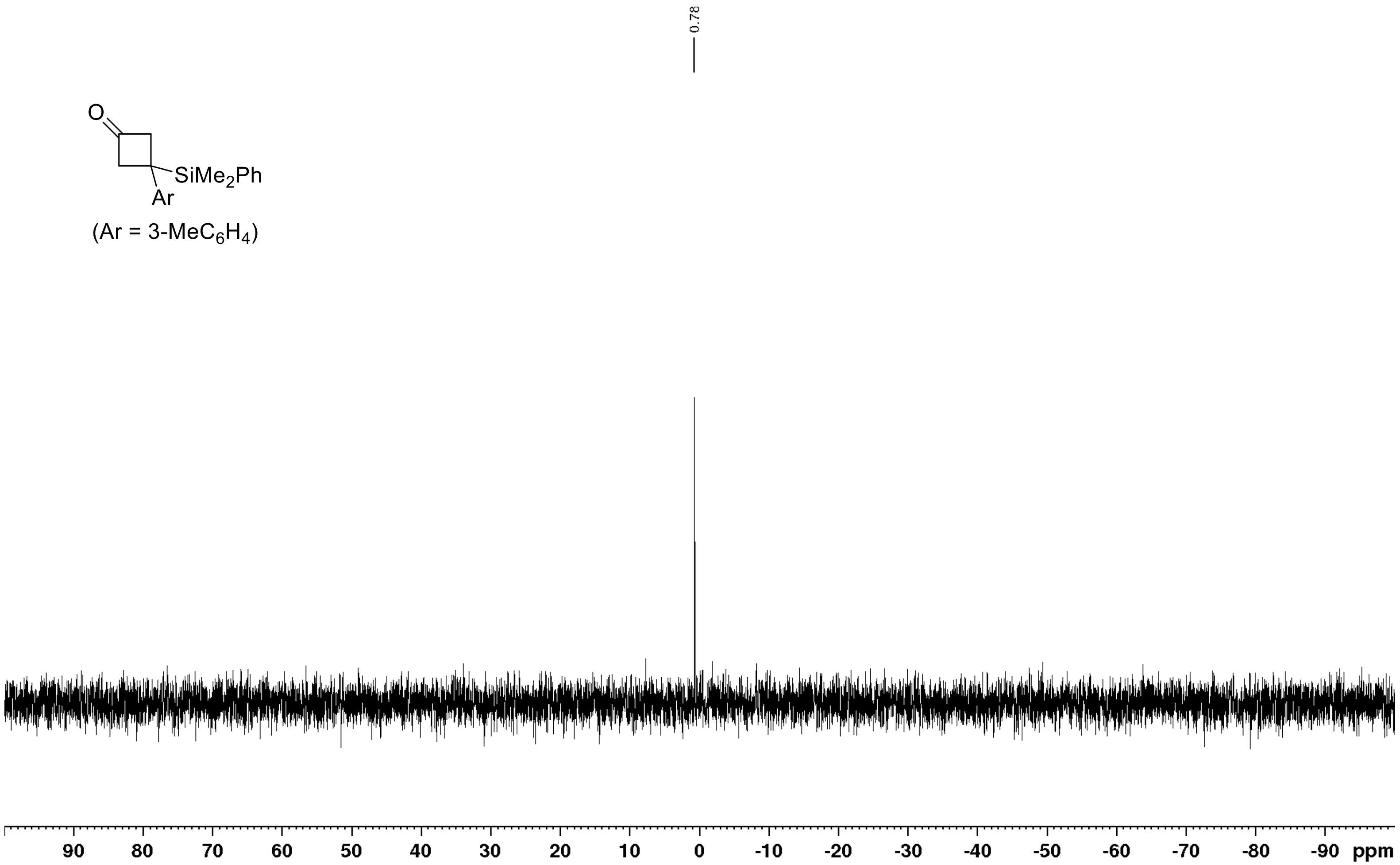

Figure S10. ^{1}H NMR (500 MHz, $CDCl_3$, 298 K) of 3-(Dimethyl(phenyl)silyl)-3-(4-methoxyphenyl)cyclobutan-1-one (**2d**)

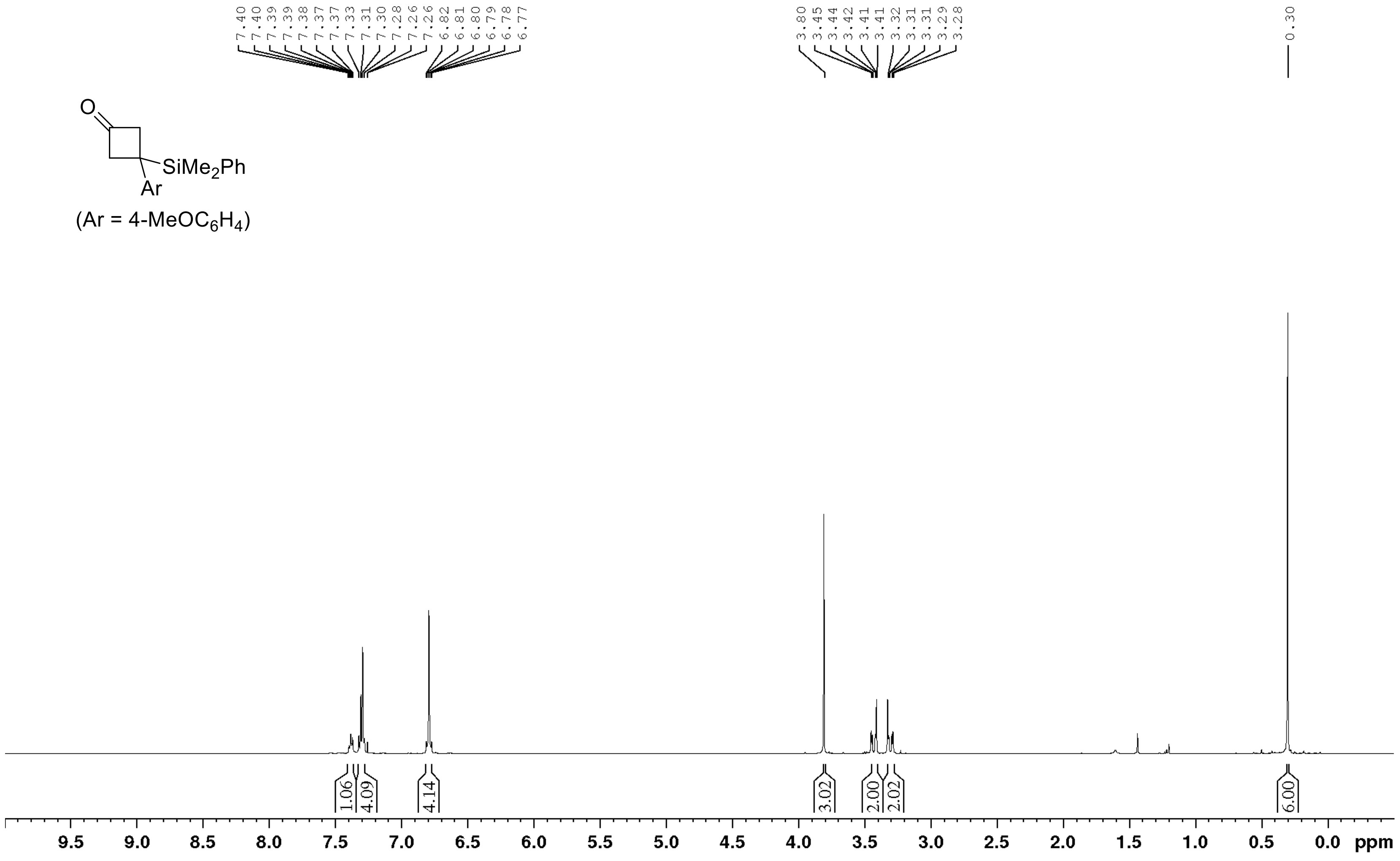

Figure S11. $^{13}C\{^1H\}$ NMR (126 MHz, $CDCl_3$, 298 K) of 3-(Dimethyl(phenyl)silyl)-3-(4-methoxyphenyl)cyclobutan-1-one (**2d**)

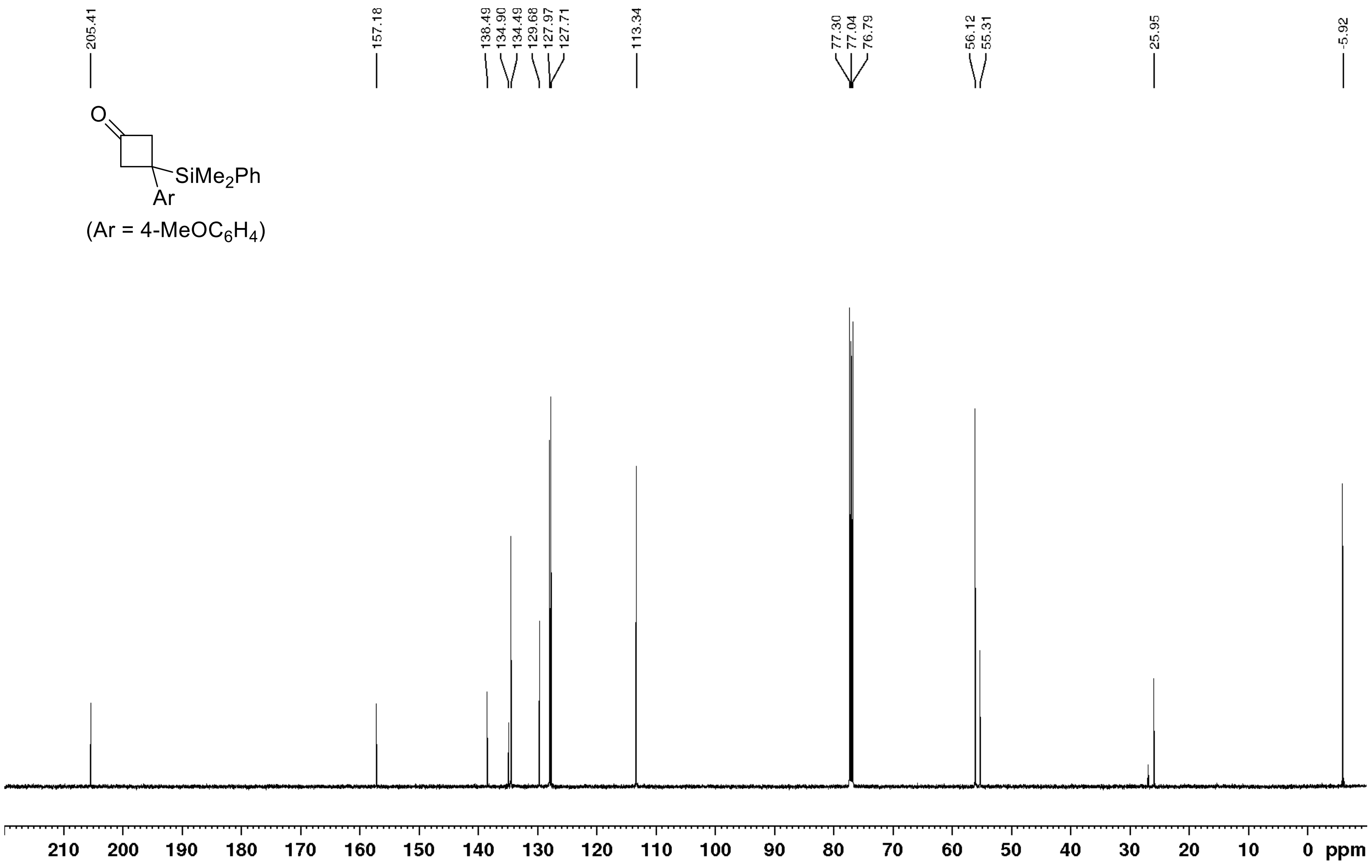

Figure S12. $^{29}Si\{^1H\}$ DEPT NMR (99 MHz, $CDCl_3$, 298 K) of 3-(Dimethyl(phenyl)silyl)-3-(4-methoxyphenyl)cyclobutan-1-one (**2d**)

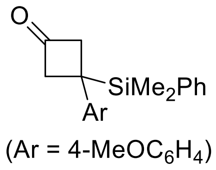

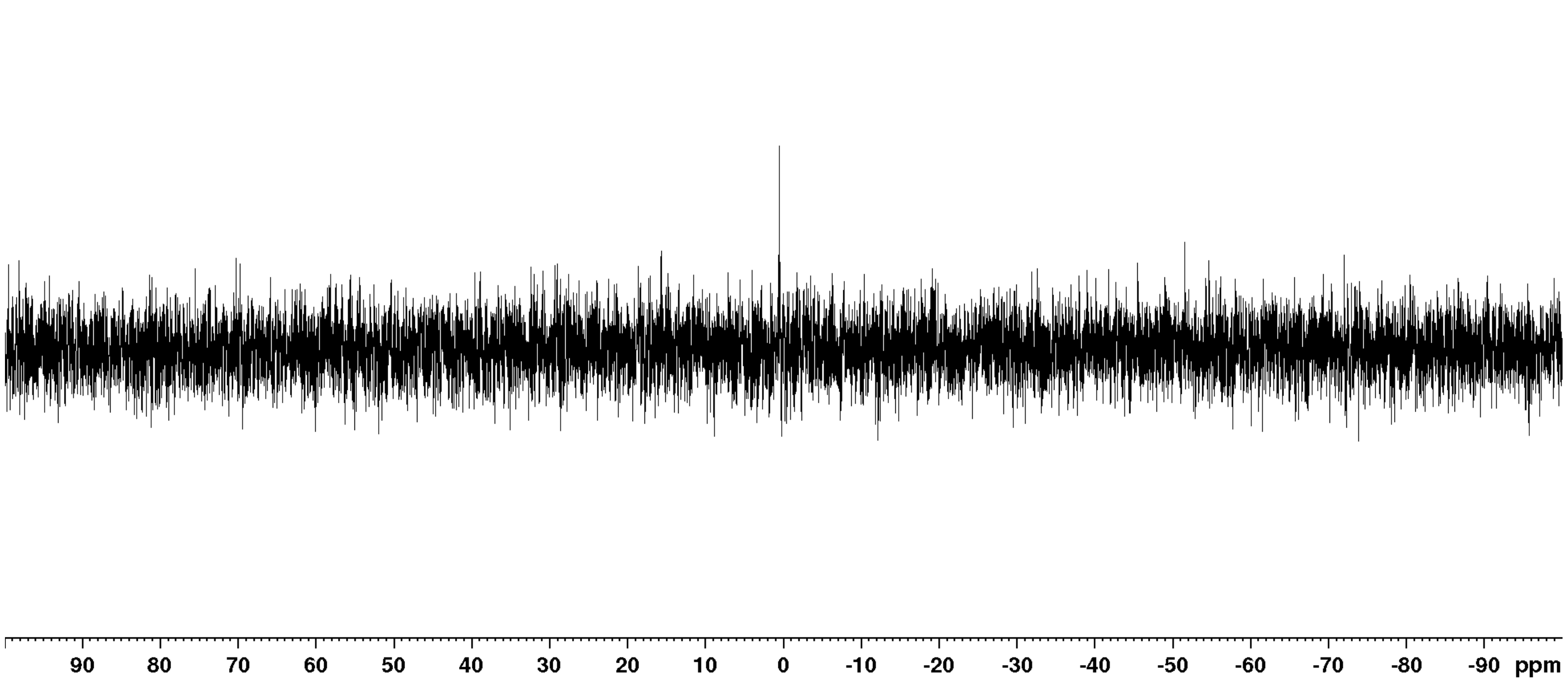

Figure S13. ^{1}H NMR spectrum (500 MHz, $CDCl_3$, 298 K) of 3-(4-Chlorophenyl)-3-(dimethyl(phenyl)silyl)cyclobutan-1-one (**2e**)

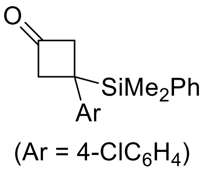

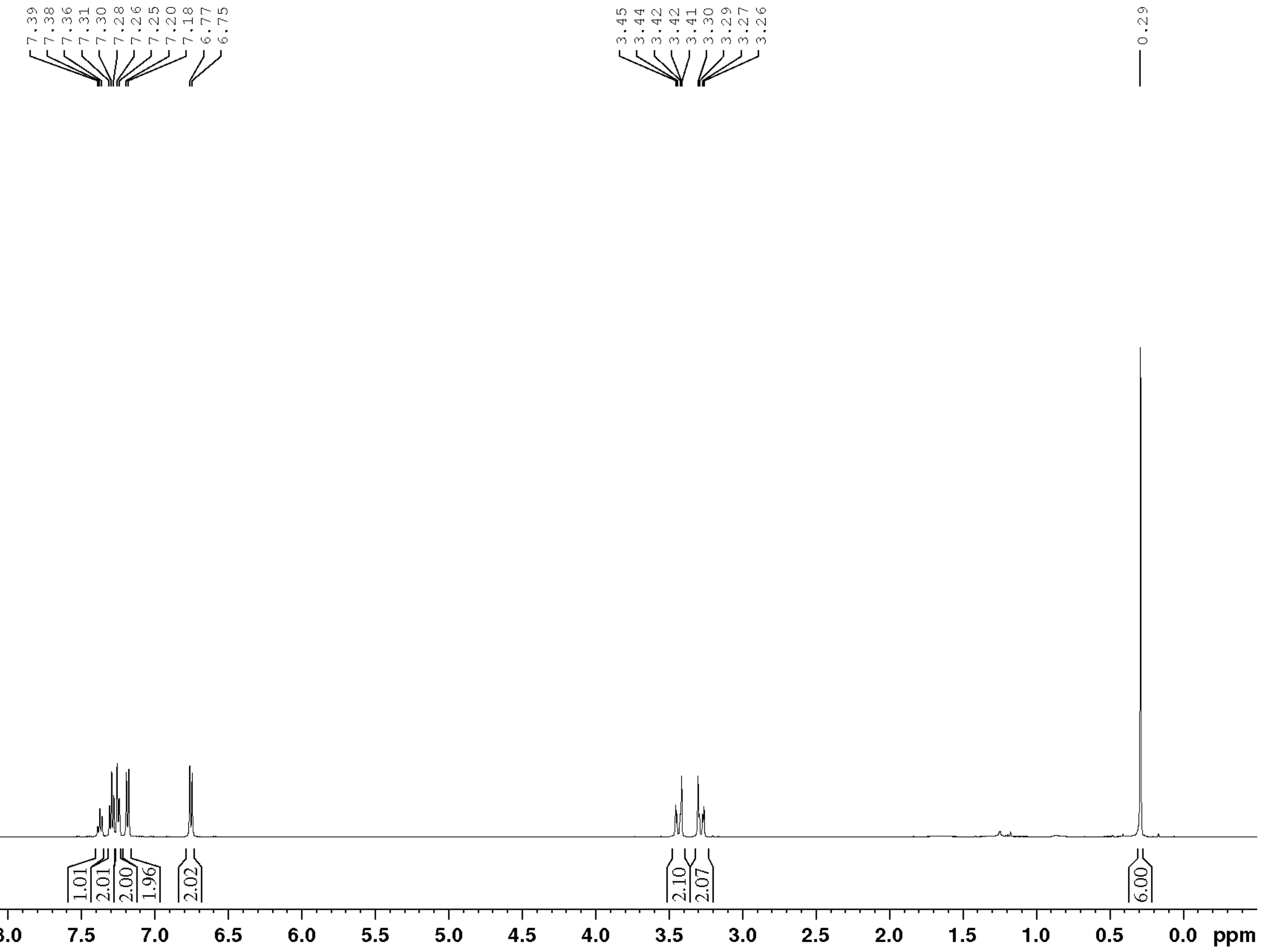

Figure S14. $^{13}C\{^1H\}$ NMR (126 MHz, $CDCl_3$, 298 K) of 3-(4-Chlorophenyl)-3-(dimethyl(phenyl)silyl)cyclobutan-1-one (**2e**)

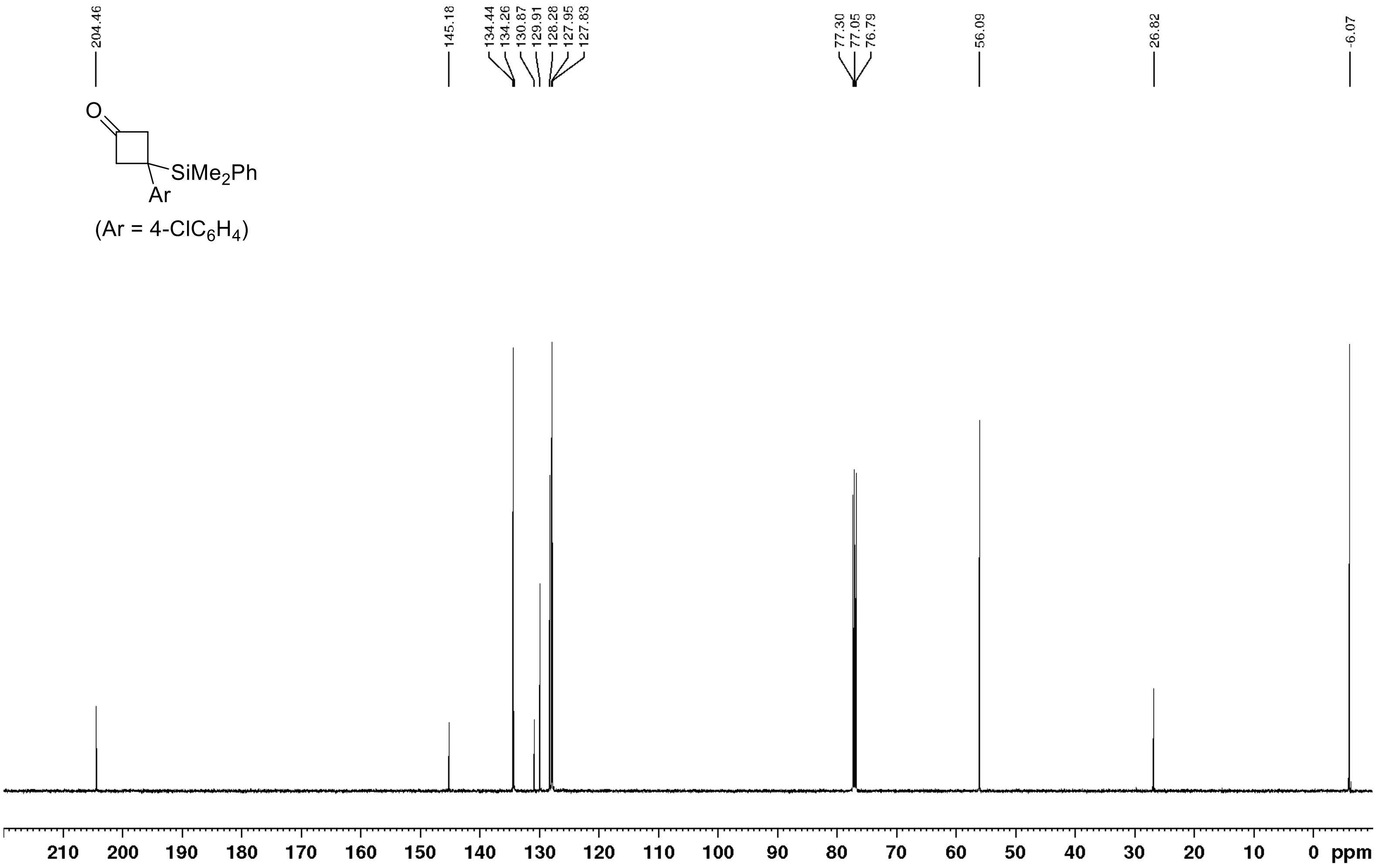

Figure S15. ^{29}Si {^1H} DEPT NMR spectrum (99 MHz, $CDCl_3$, 298 K) of 3-(4-Chlorophenyl)-3-(dimethyl(phenyl)silyl)cyclobutan-1-one (**2e**)

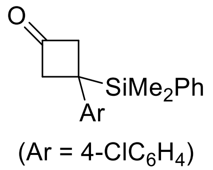

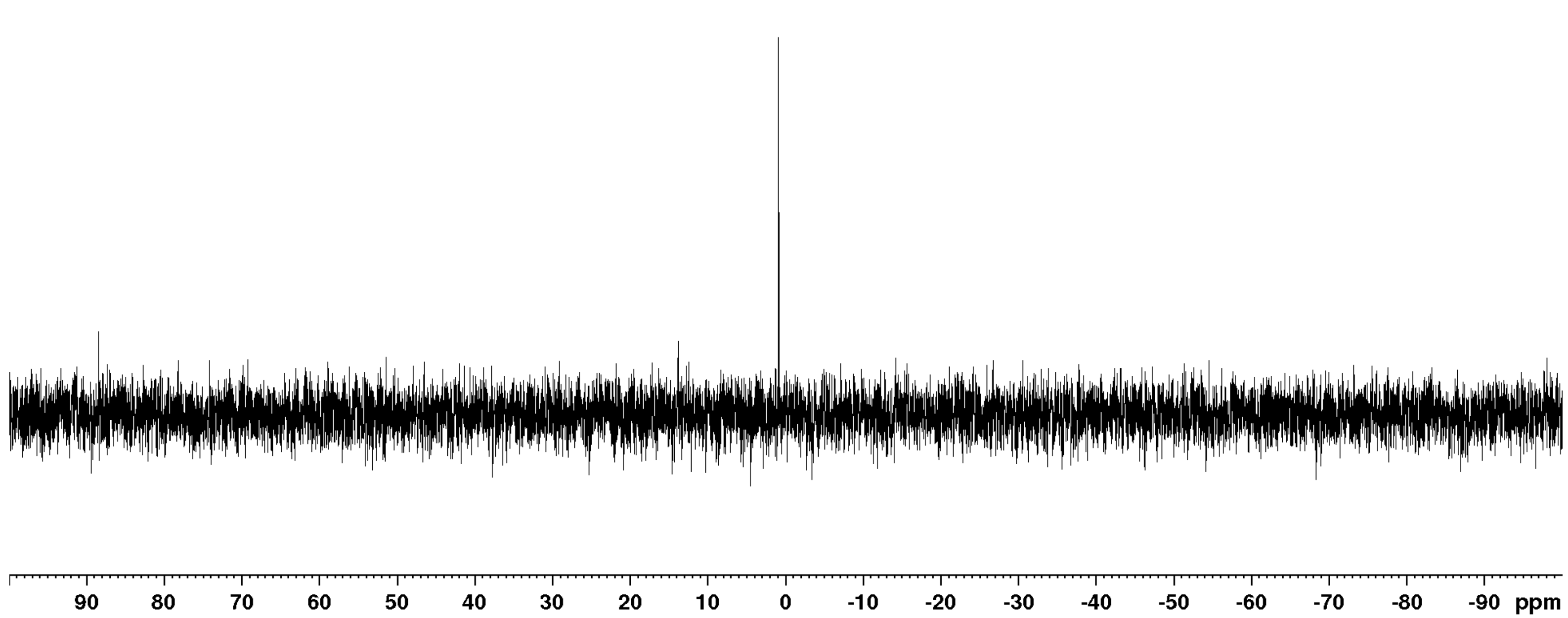

Figure S16. ^{1}H NMR spectrum (400 MHz, $CDCl_3$, 298 K) of 3-(Dimethyl(phenyl)silyl)-3-(4-fluorophenyl)cyclobutan-1-one (**2f**)

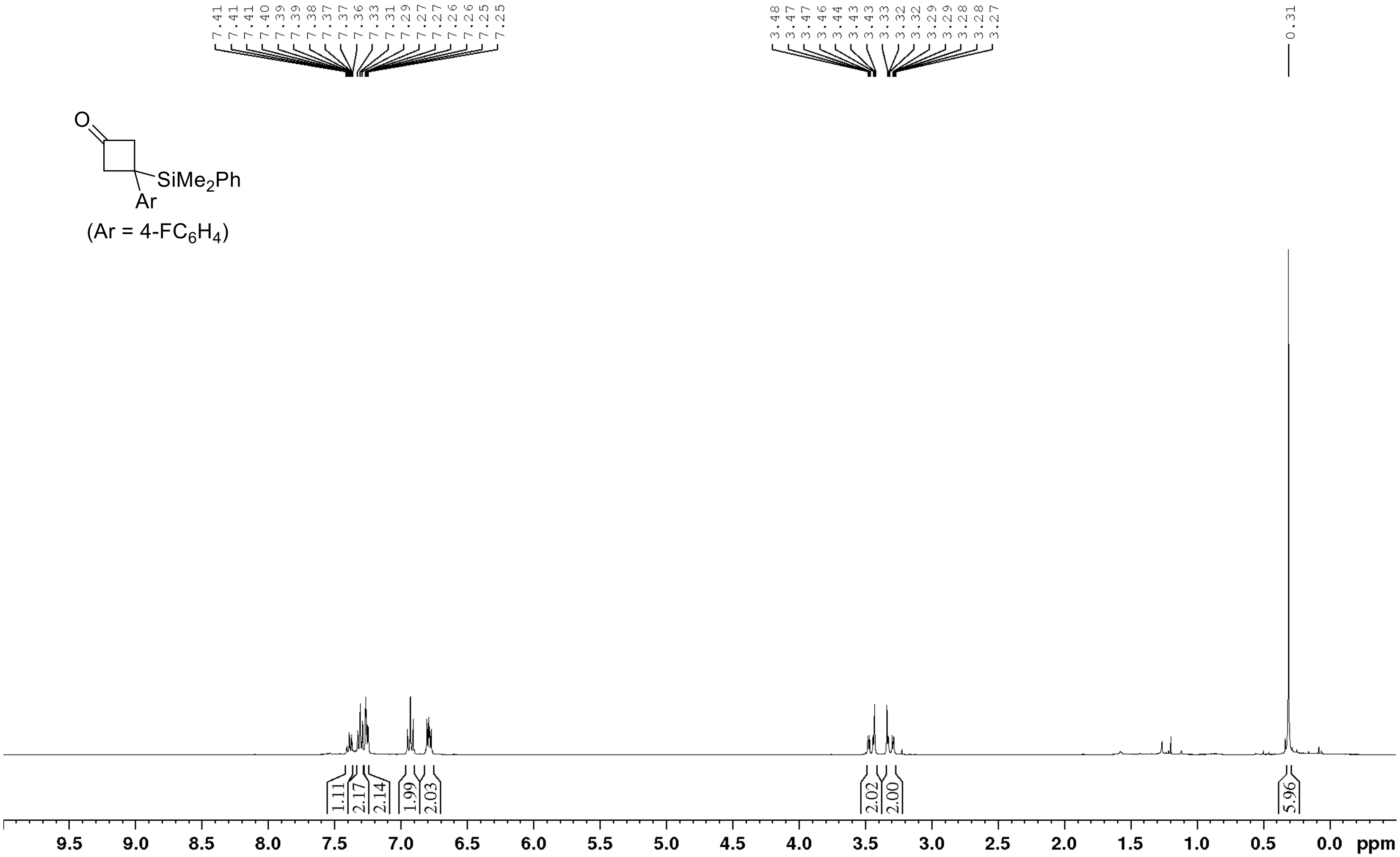

Figure S17. $^{13}C\{^{1}H\}$ NMR (101 MHz, $CDCl_3$, 298 K) of 3-(Dimethyl(phenyl)silyl)-3-(4-fluorophenyl)cyclobutan-1-one (**2f**)

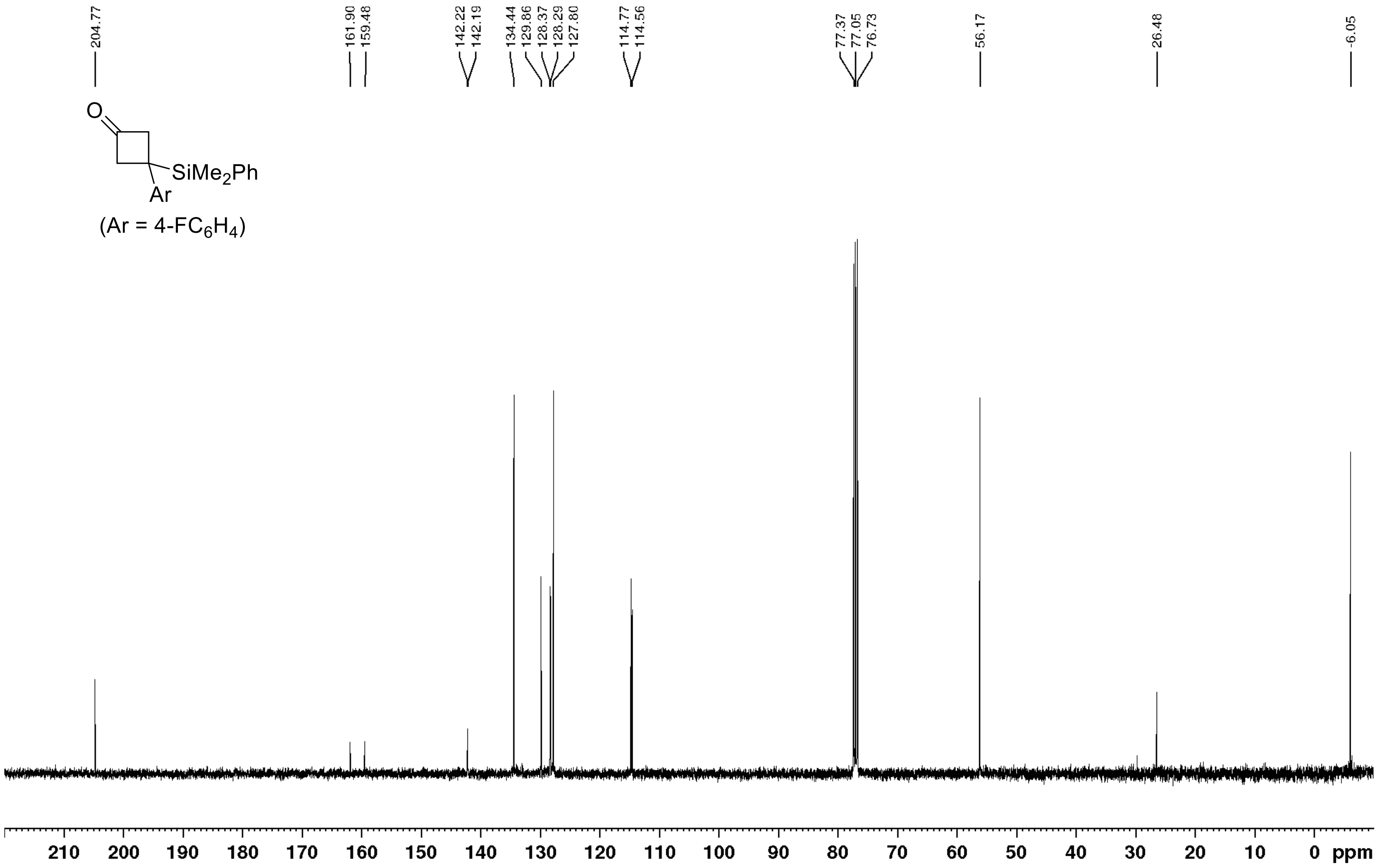

Figure S18. ^{19}F NMR (471 MHz, $CDCl_3$, 298 K) of 3-(Dimethyl(phenyl)silyl)-3-(4-fluorophenyl)cyclobutan-1-one (**2f**)

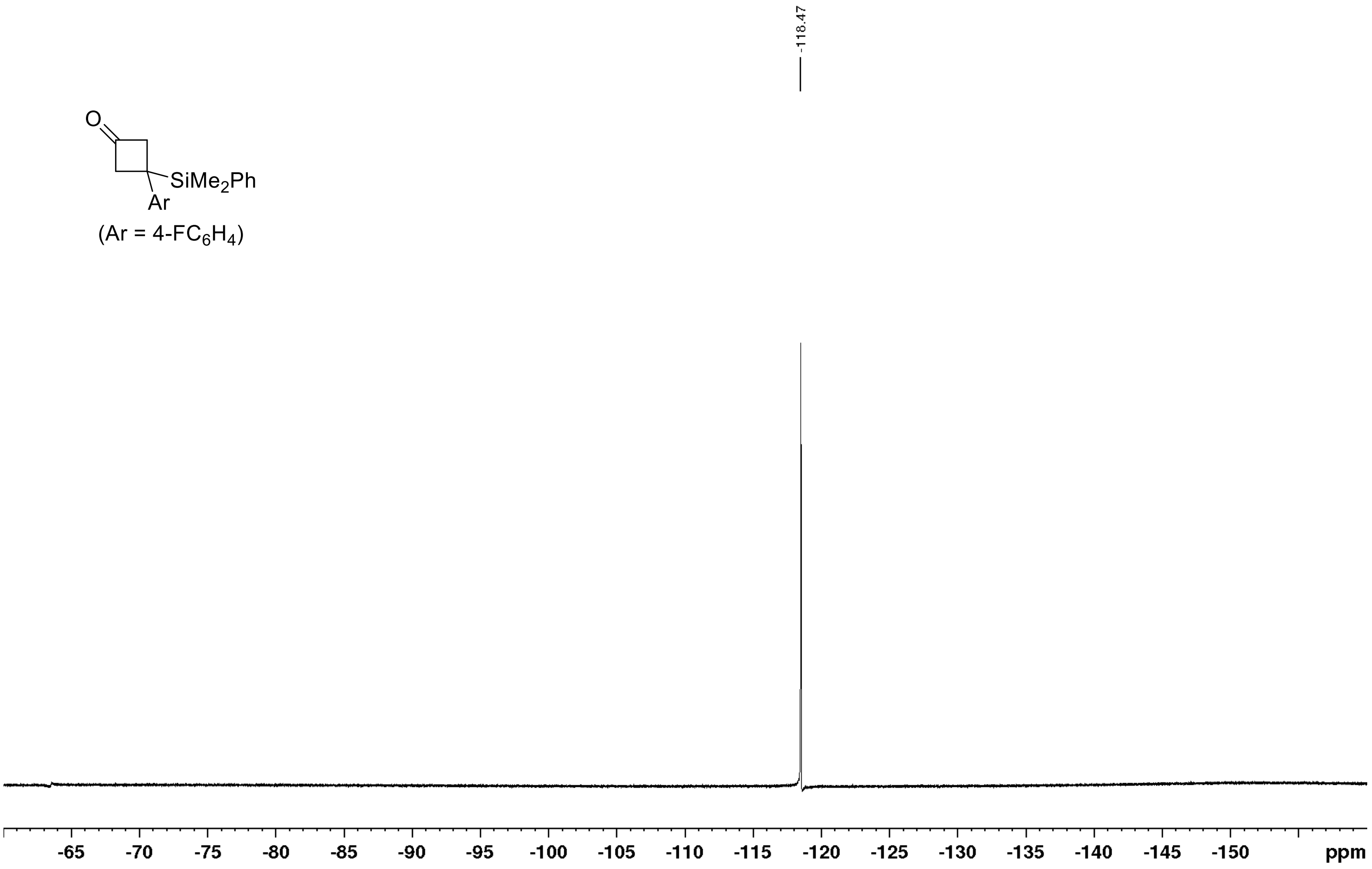

Figure S19. ^{29}Si {^{1}H} DEPT NMR spectrum (99 MHz, $CDCl_3$) of 3-(Dimethyl(phenyl)silyl)-3-(4-fluorophenyl)cyclobutan-1-one (**2f**)

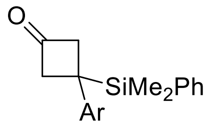

(Ar = 4-FC_6H_4)

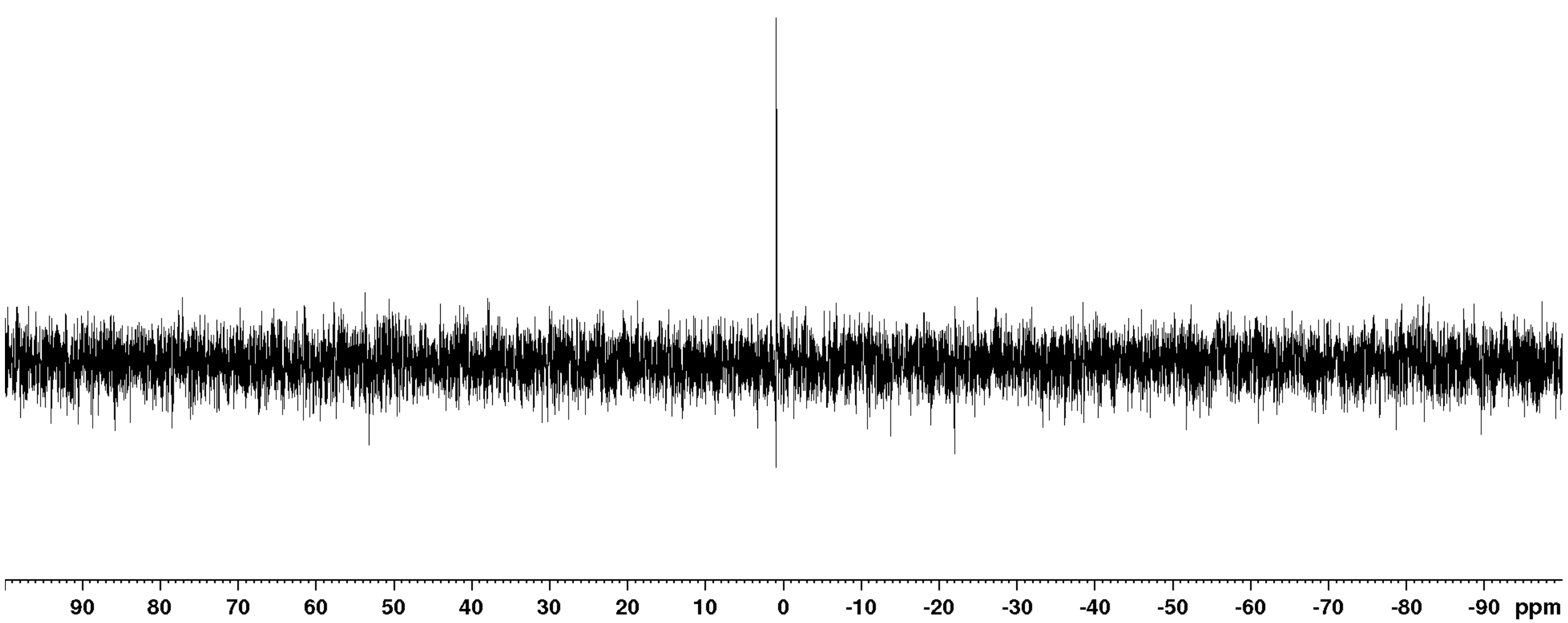

Figure S20. ^{1}H NMR spectrum (500 MHz, $CDCl_3$, 298 K) of 3-Butyl-3-(dimethyl(phenyl)silyl)cyclobutan-1-one (**2g**)

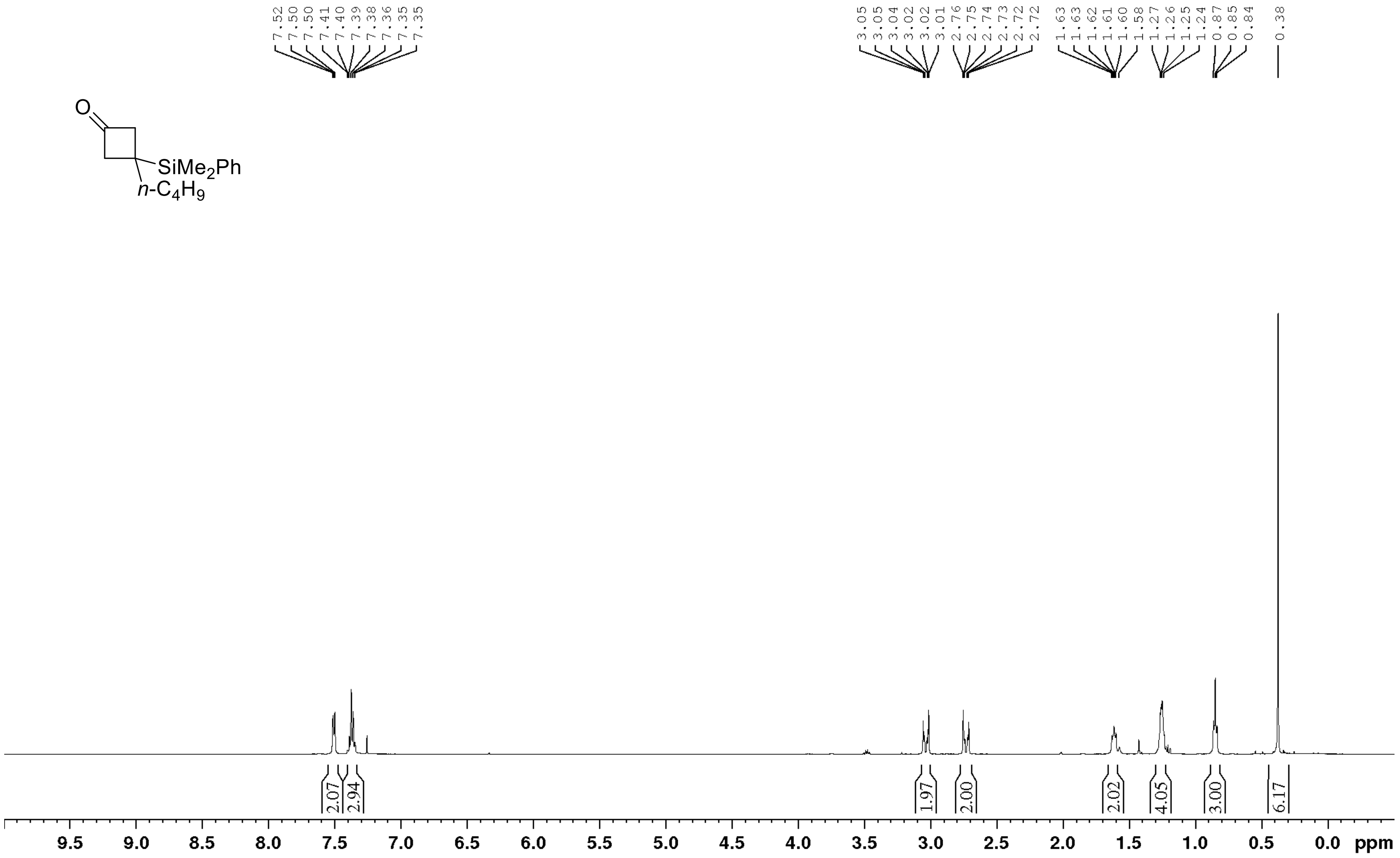

Figure S21. $^{13}C\{^{1}H\}$ NMR (126 MHz, $CDCl_3$, 298 K) of 3-Butyl-3-(dimethyl(phenyl)silyl)cyclobutan-1-one (**2g**)

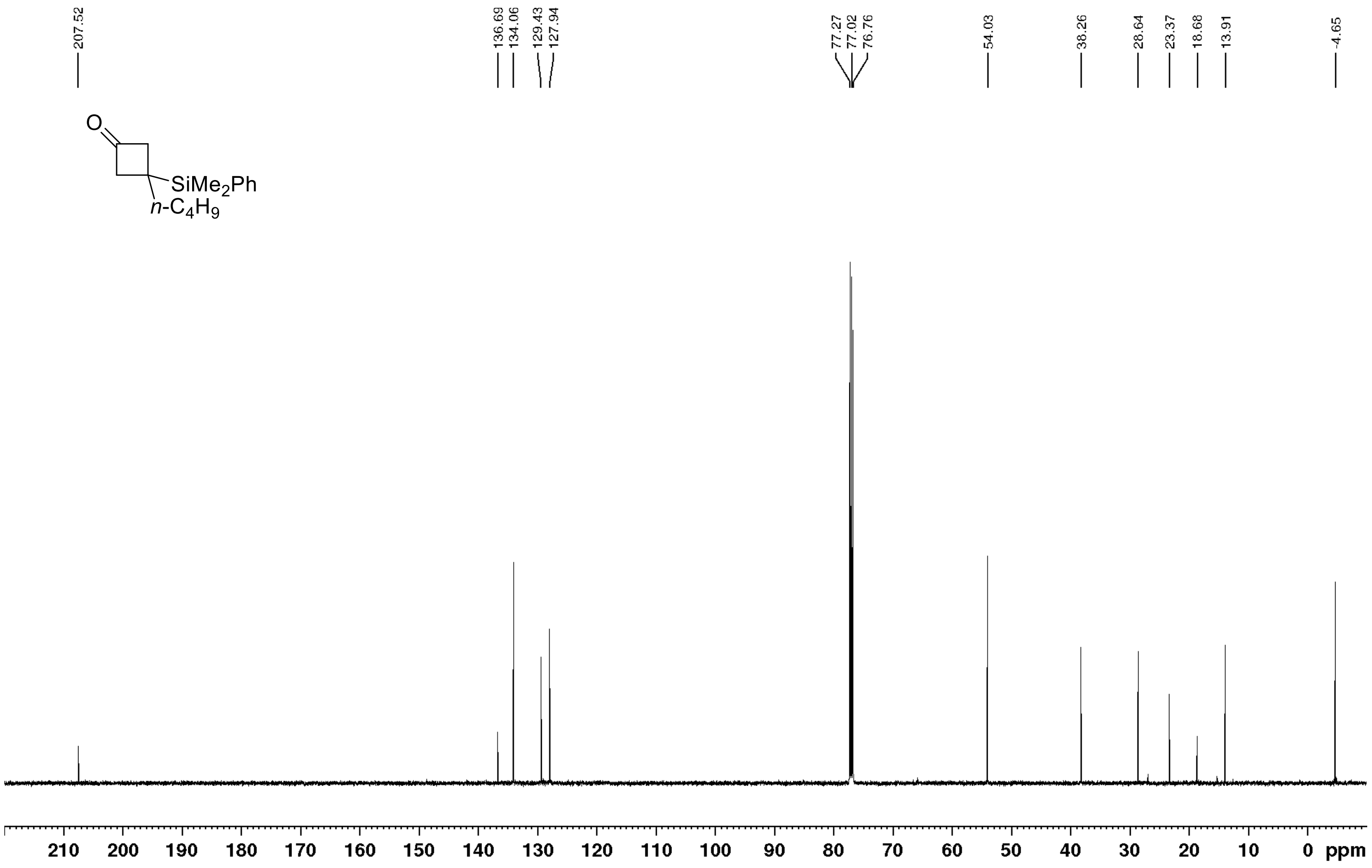

Figure S22. $^{29}Si\{^1H\}$ DEPT NMR (99 MHz, $CDCl_3$, 298 K) of 3-Butyl-3-(dimethyl(phenyl)silyl)cyclobutan-1-one (**2g**)

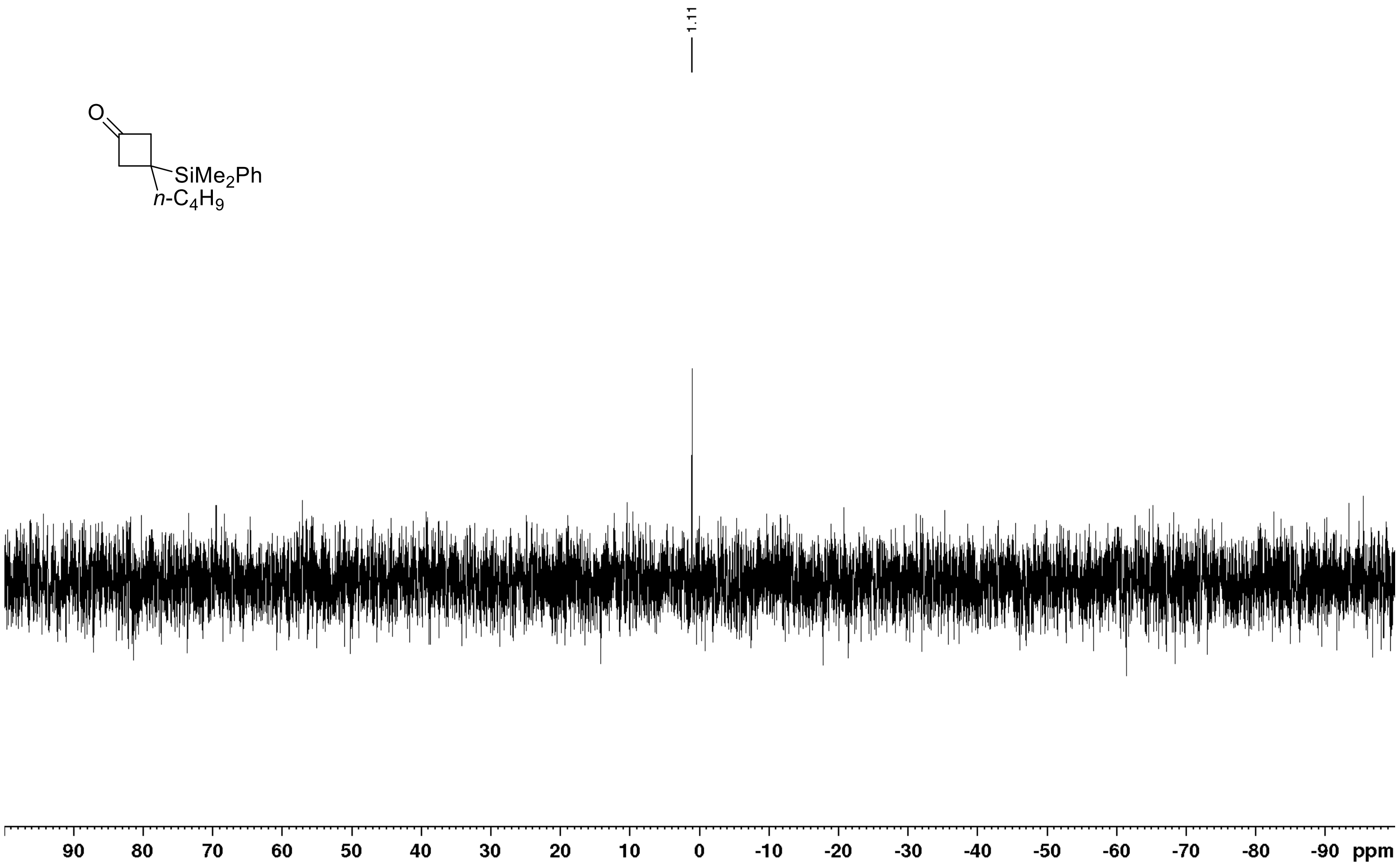

Figure S23. ^{1}H NMR spectrum (400 MHz, $CDCl_3$, 298 K) of 3-(Dimethyl(phenyl)silyl)-3-hexylcyclobutan-1-one (**2h**)

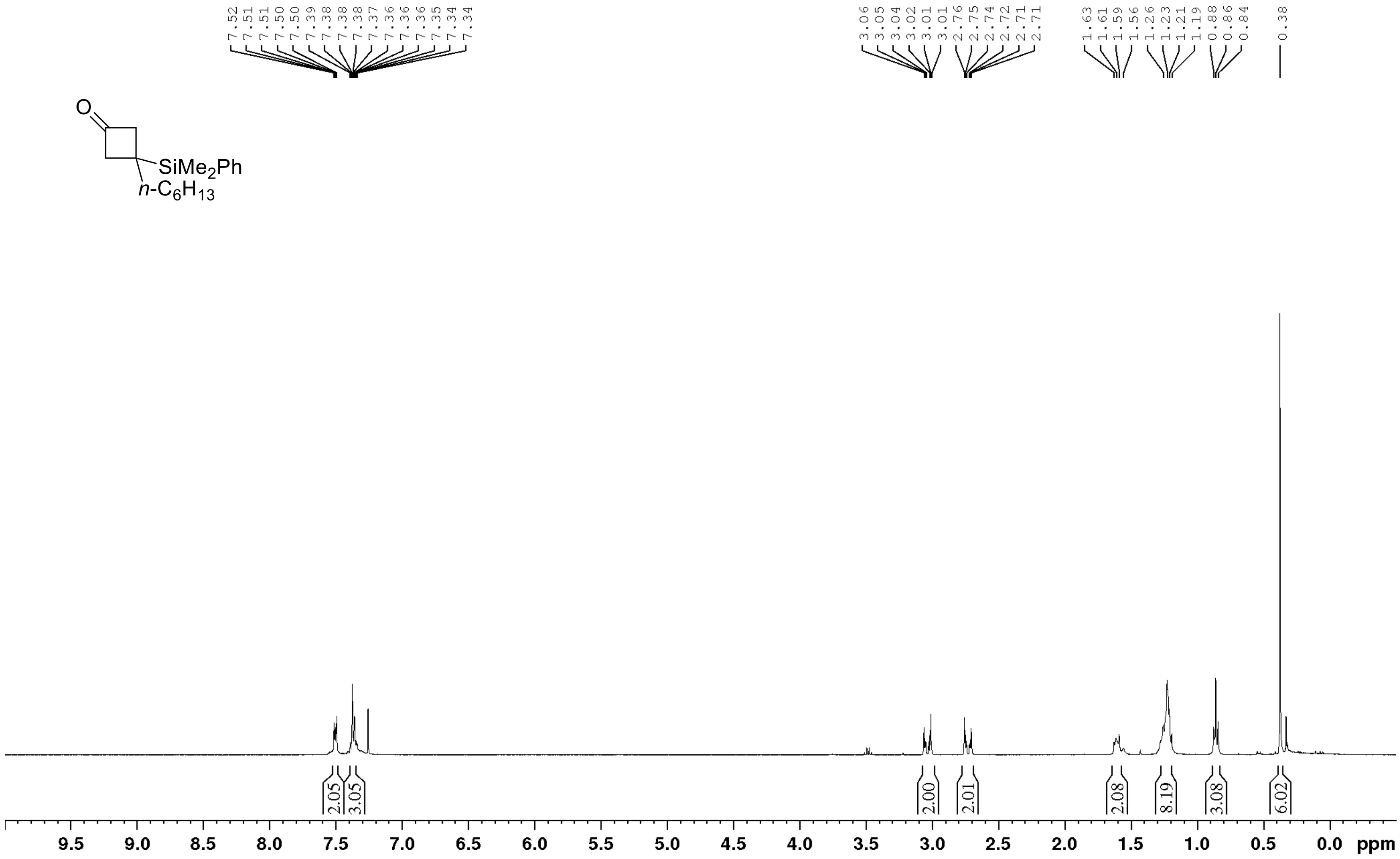

Figure S24. $^{13}C\{^1H\}$ NMR (101 MHz, $CDCl_3$, 298 K) of 3-(Dimethyl(phenyl)silyl)-3-hexylcyclobutan-1-one (**2h**)

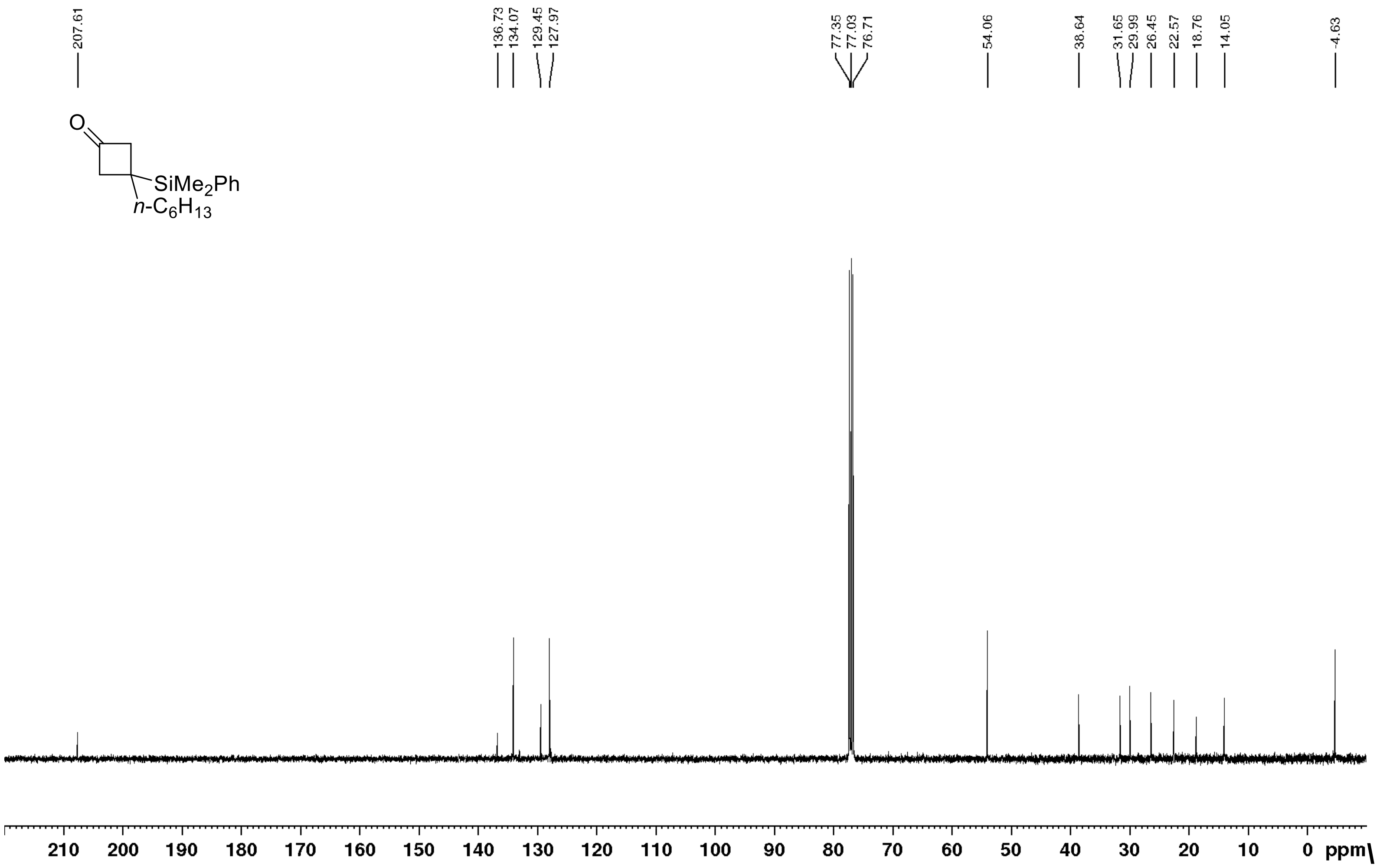

Figure S25. ^{29}Si{^{1}H} DEPT NMR (99 MHz, $CDCl_3$, 298 K) of 3-(Dimethyl(phenyl)silyl)-3-hexylcyclobutan-1-one (**2h**)

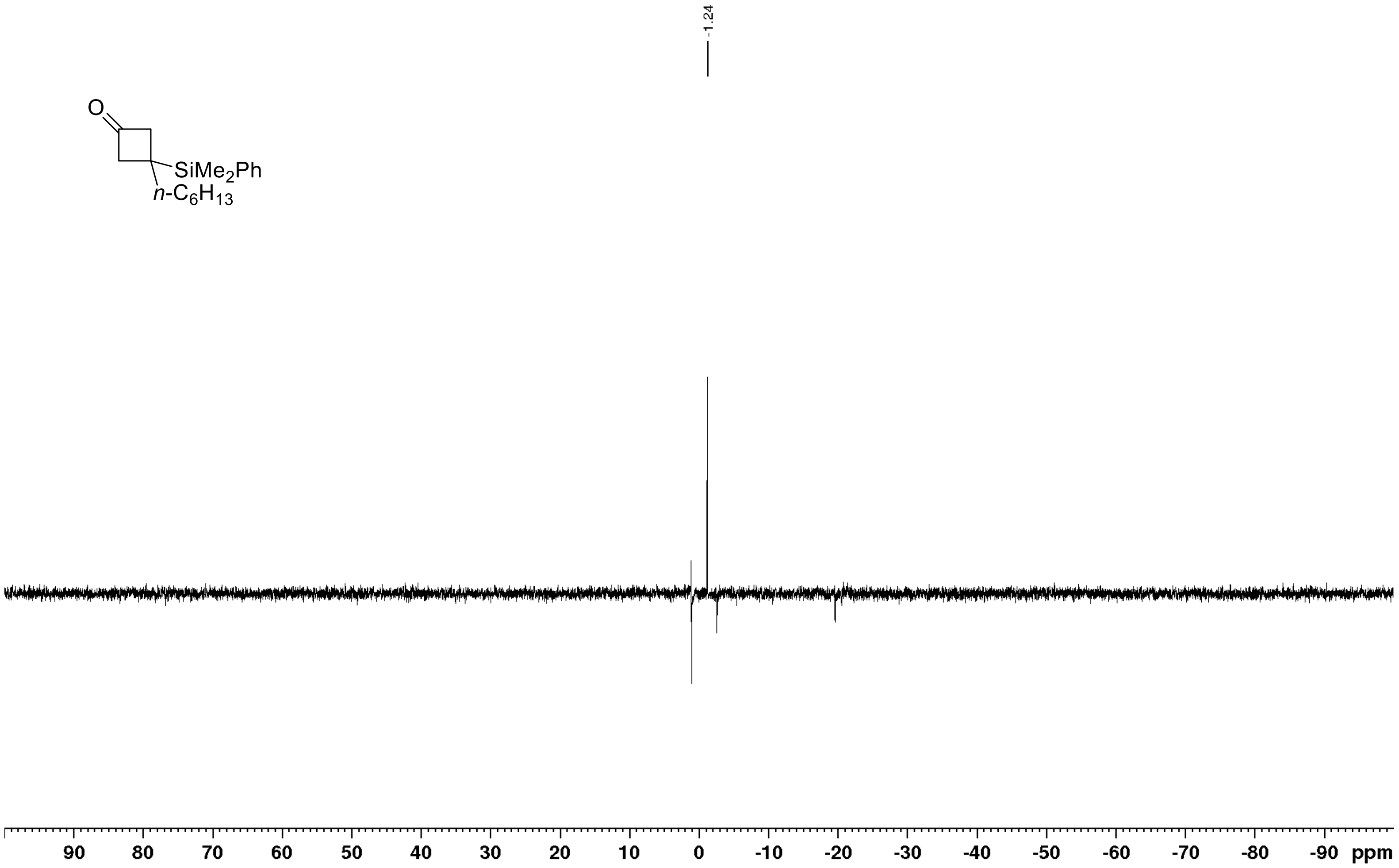

Figure S26. ^{1}H NMR spectrum (500 MHz, $CDCl_3$, 298 K) of 3-(Dimethyl(phenyl)silyl)-3-phenethylcyclobutan-1-one (**2i**)

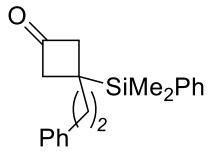

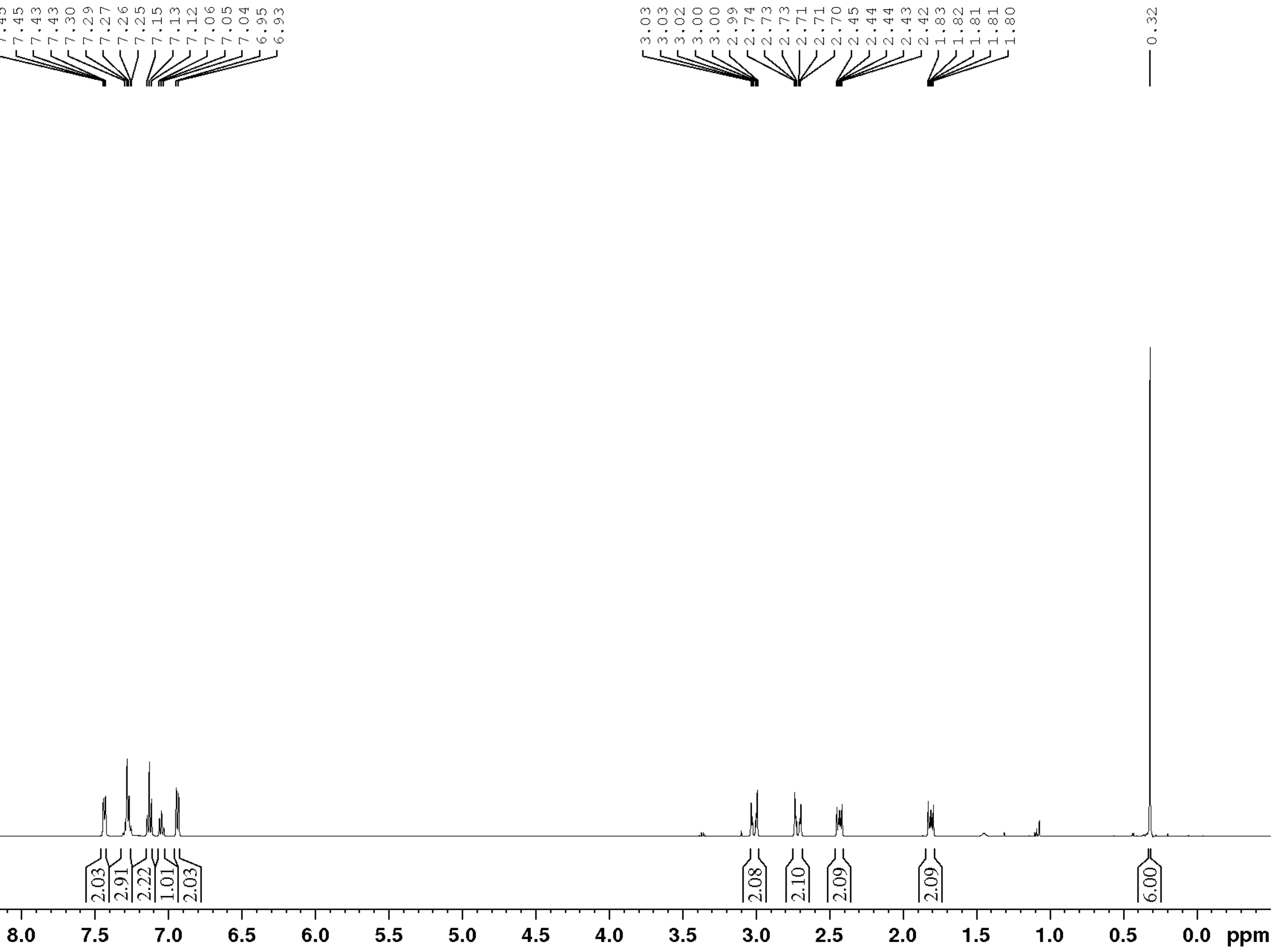

Figure S27. $^{13}C\{^{1}H\}$ NMR (126 MHz, $CDCl_3$, 298 K) of 3-(Dimethyl(phenyl)silyl)-3-phenethylcyclobutan-1-one (**2i**)

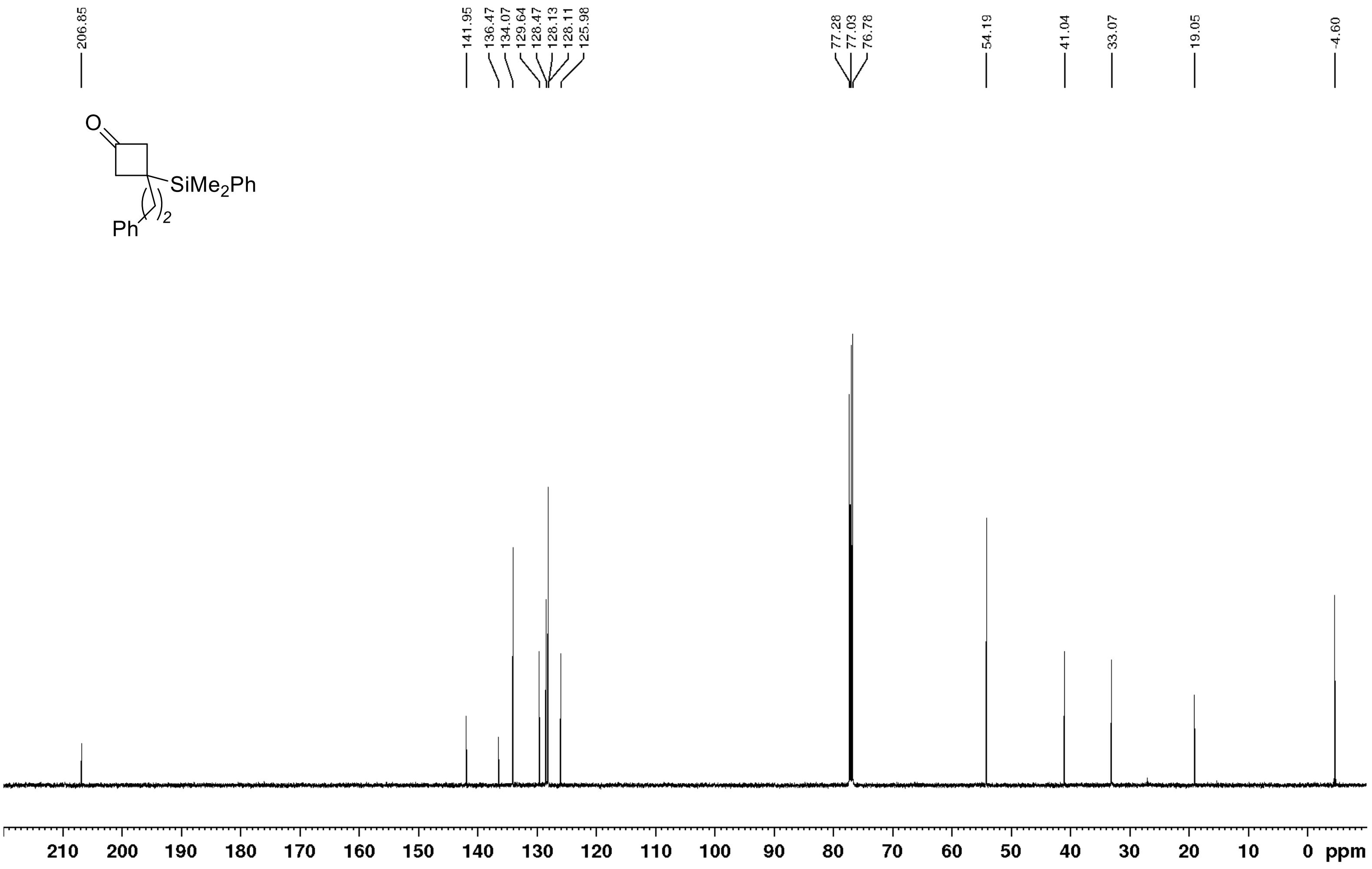

Figure S28. $^{29}Si\{^1H\}$ DEPT NMR (99 MHz, $CDCl_3$, 298 K) of 3-(Dimethyl(phenyl)silyl)-3-phenethylcyclobutan-1-one (**2i**)

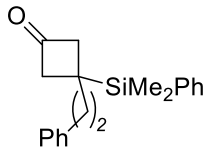

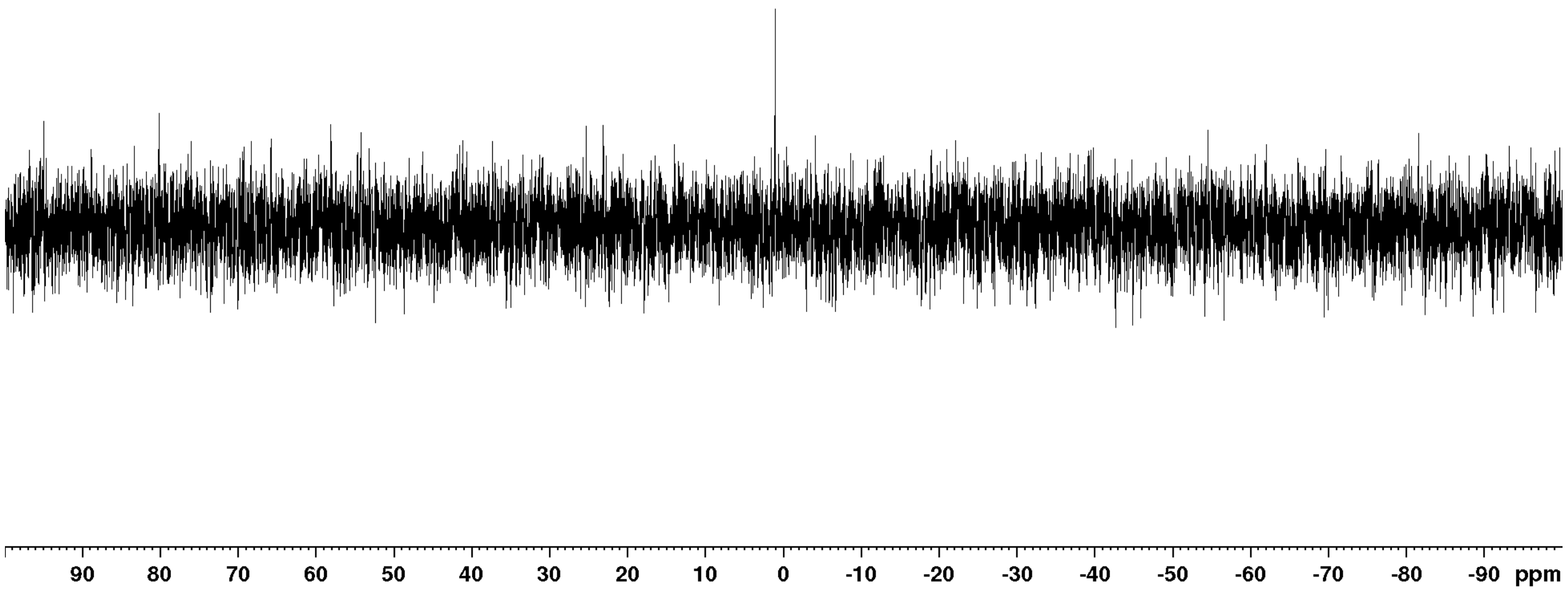

Figure S29. ^{1}H NMR spectrum (500 MHz, $CDCl_3$, 298 K) of 3-(Dimethyl(phenyl)silyl)-3-(3-phenylpropyl)cyclobutan-1-one (**2j**)

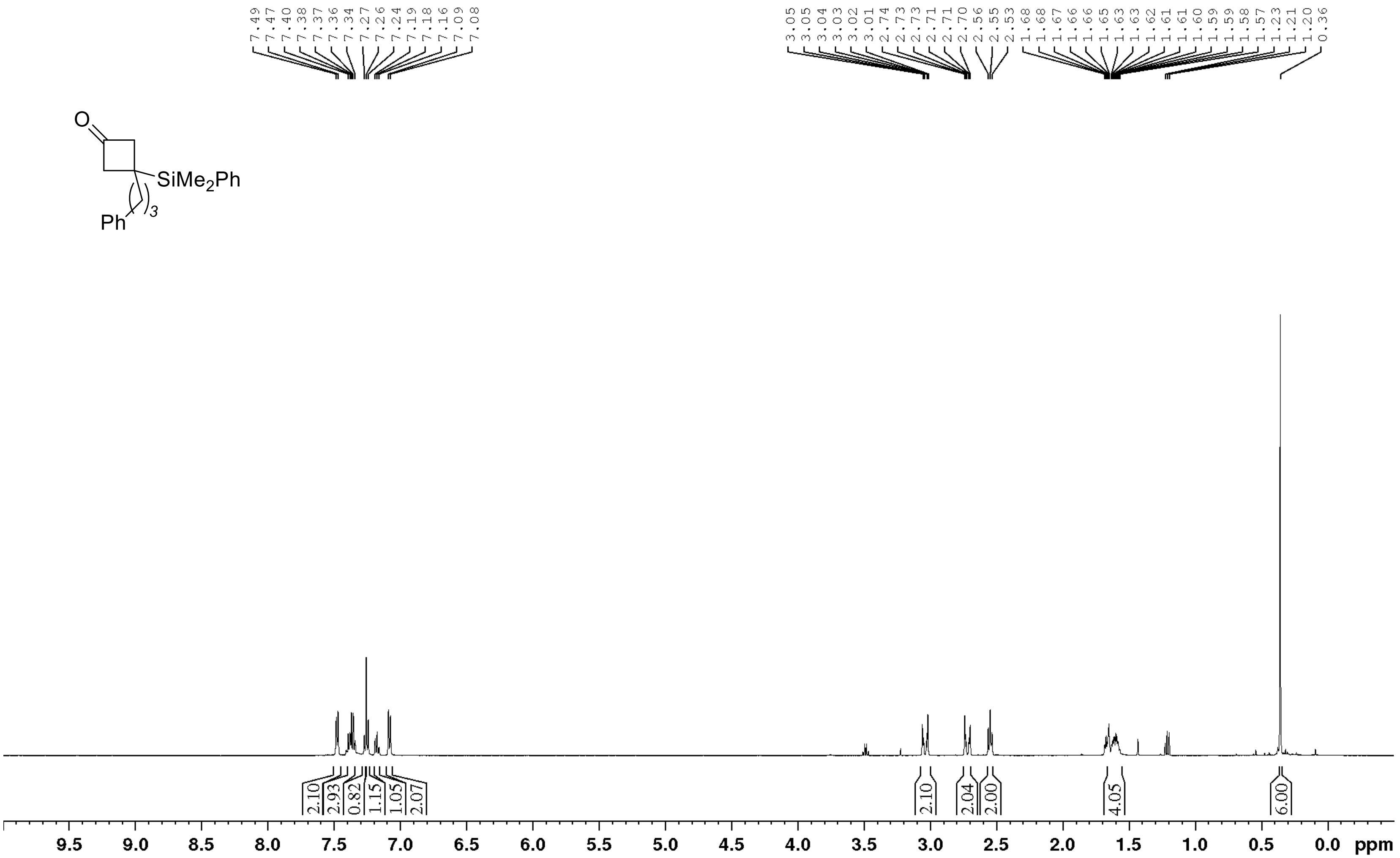

Figure S30. $^{13}C\{^{1}H\}$ NMR (126 MHz, $CDCl_3$, 298 K) of 3-(Dimethyl(phenyl)silyl)-3-(3-phenylpropyl)cyclobutan-1-one (**2j**)

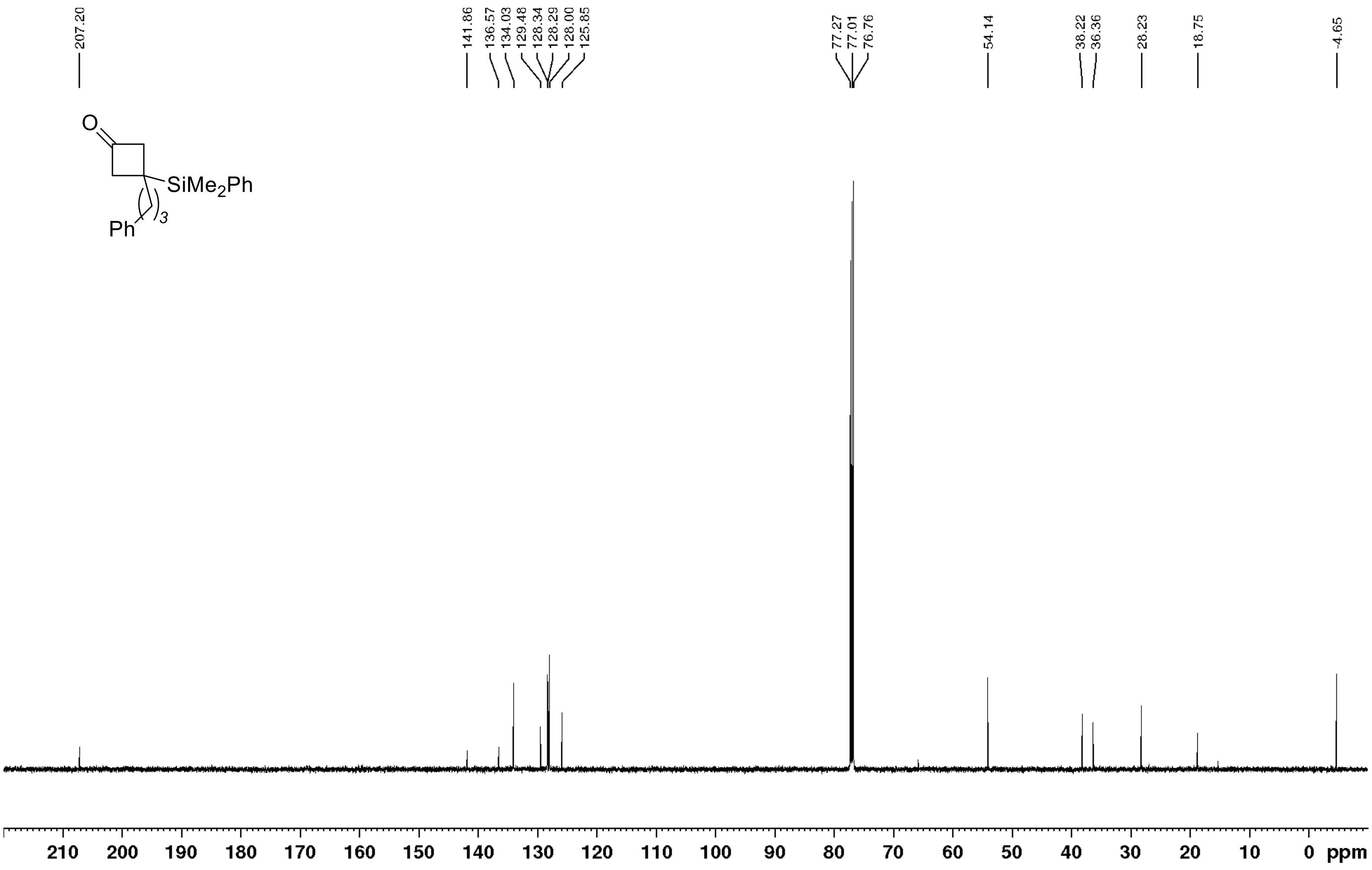

Figure S31. $^{29}Si\{^1H\}$ DEPT NMR (99 MHz, $CDCl_3$, 298 K) of 3-(Dimethyl(phenyl)silyl)-3-(3-phenylpropyl)cyclobutan-1-one (**2j**)

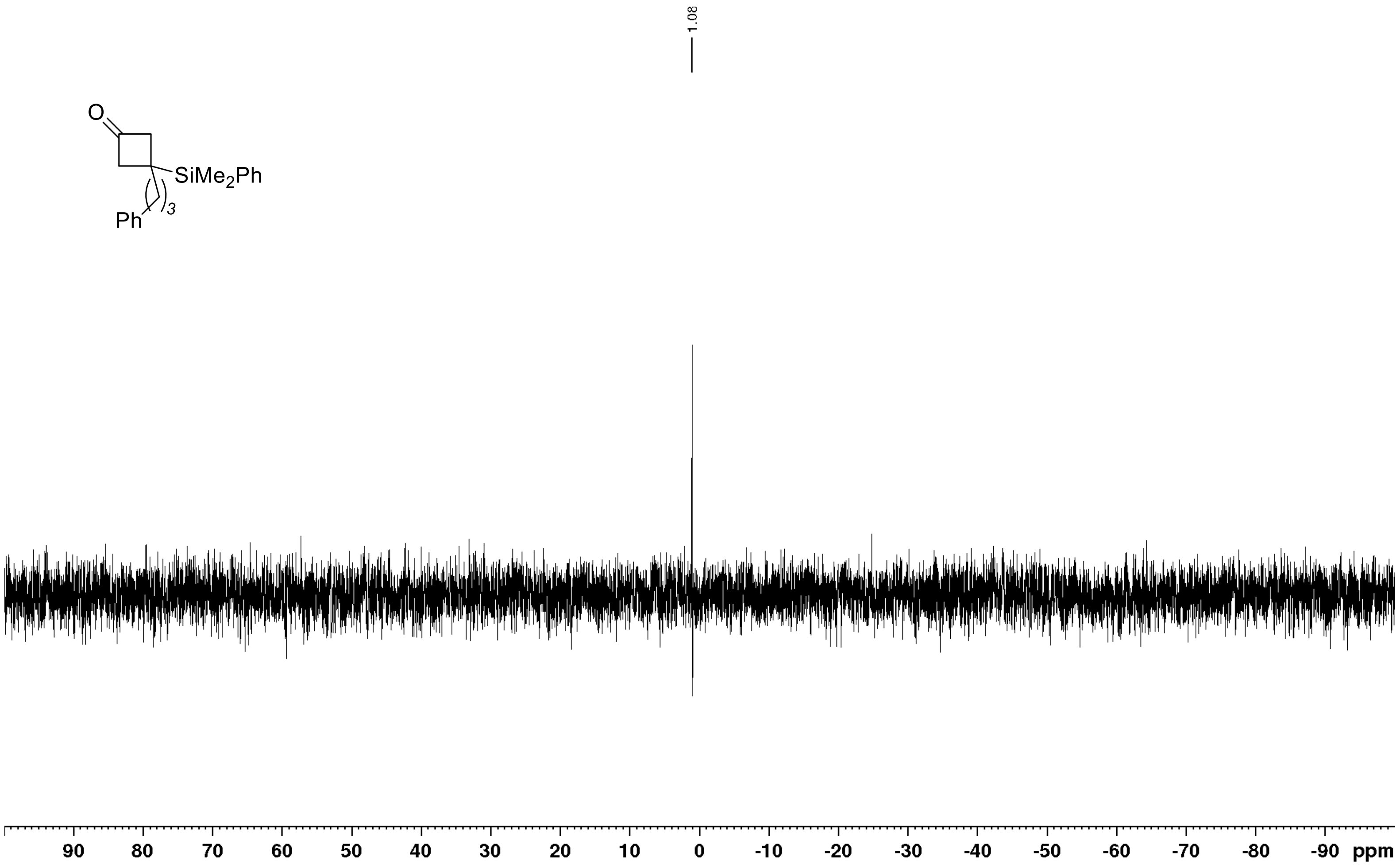

Figure S32. ^{1}H NMR spectrum (400 MHz, $CDCl_3$, 298 K) of 3-(4-Chlorobutyl)-3-(dimethyl(phenyl)silyl)cyclobutan-1-one (**2k**)

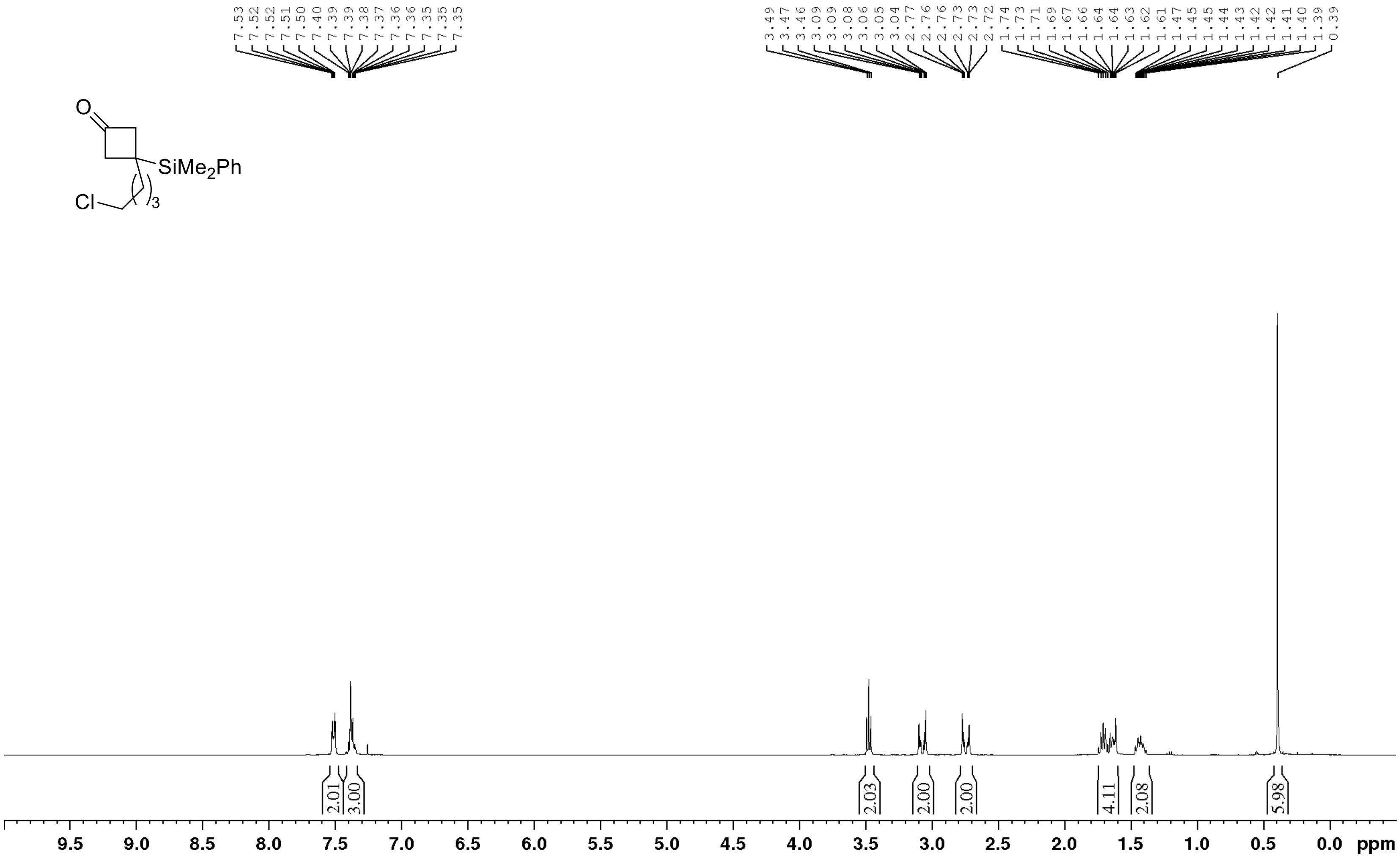

Figure S33. $^{13}C\{^{1}H\}$ NMR (101 MHz, $CDCl_3$, 298 K) of 3-(4-Chlorobutyl)-3-(dimethyl(phenyl)silyl)cyclobutan-1-one (**2k**)

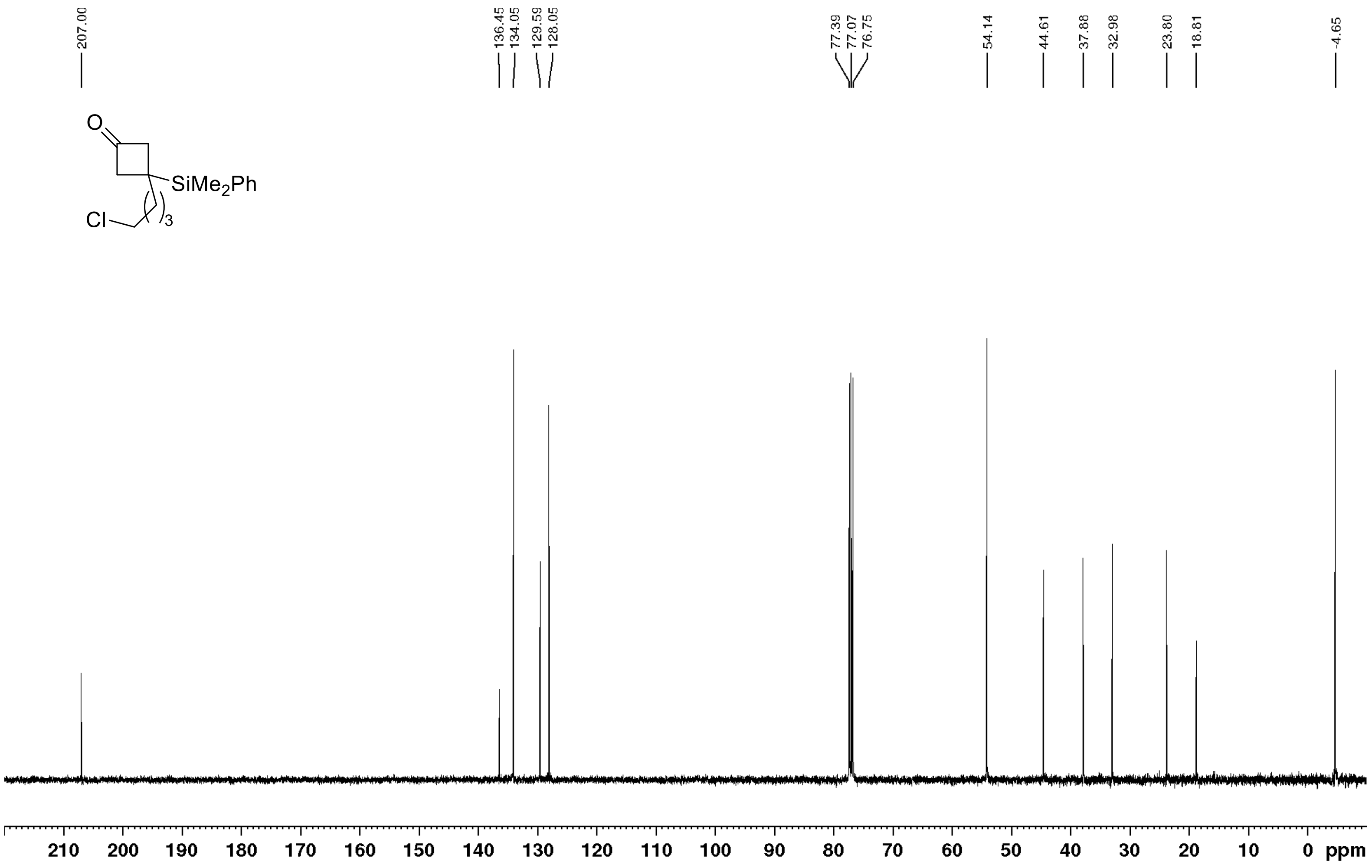

Figure S34. $^{29}Si\{^{1}H\}$ DEPT NMR (99 MHz, $CDCl_3$, 298 K) of 3-(4-Chlorobutyl)-3-(dimethyl(phenyl)silyl)cyclobutan-1-one (**2k**)

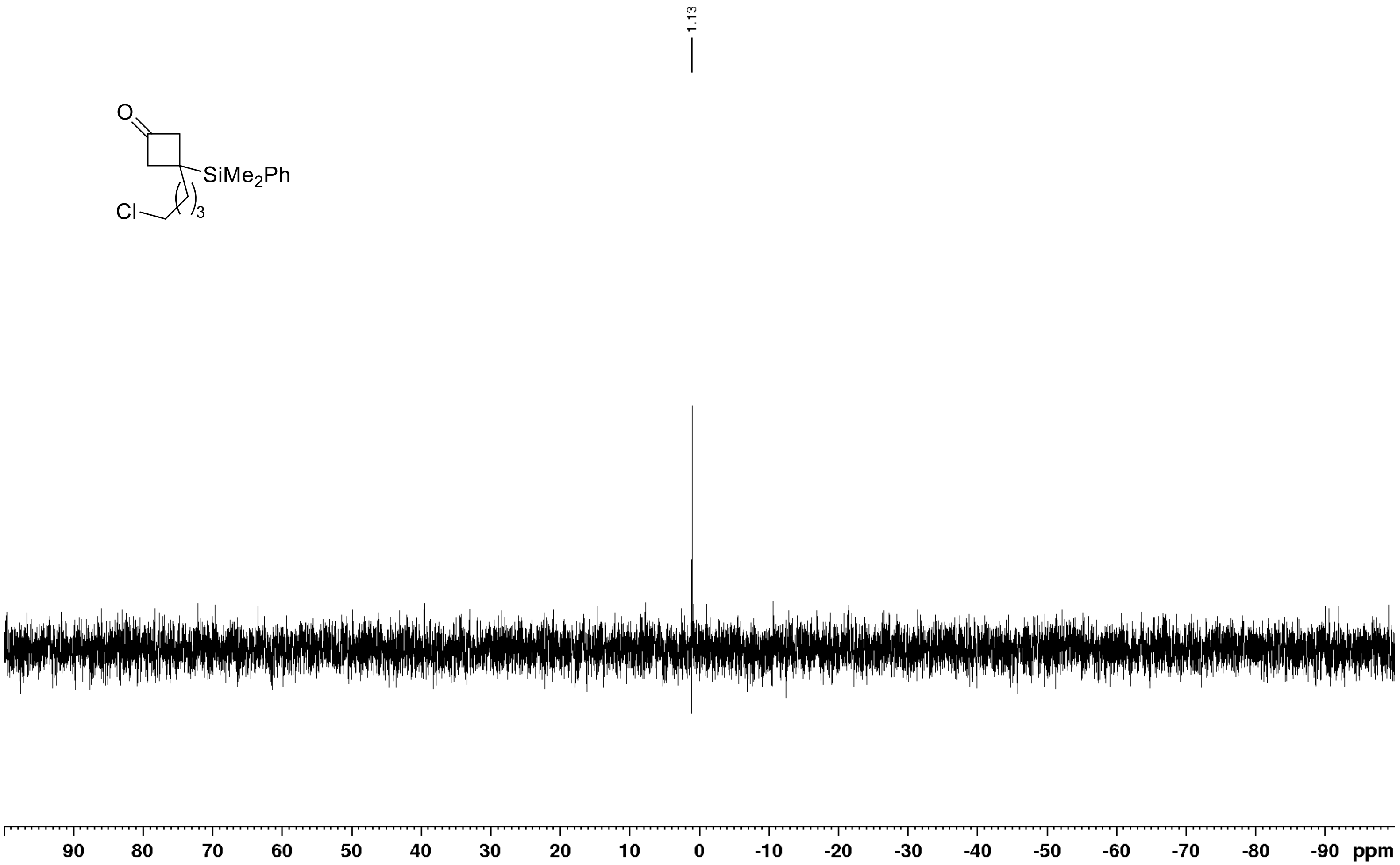

Figure S35. ^1H NMR spectrum (400 MHz, $CDCl_3$, 298 K) of 3-Cyclopropyl-3-(dimethyl(phenyl)silyl)cyclobutan-1-one (**2l**)

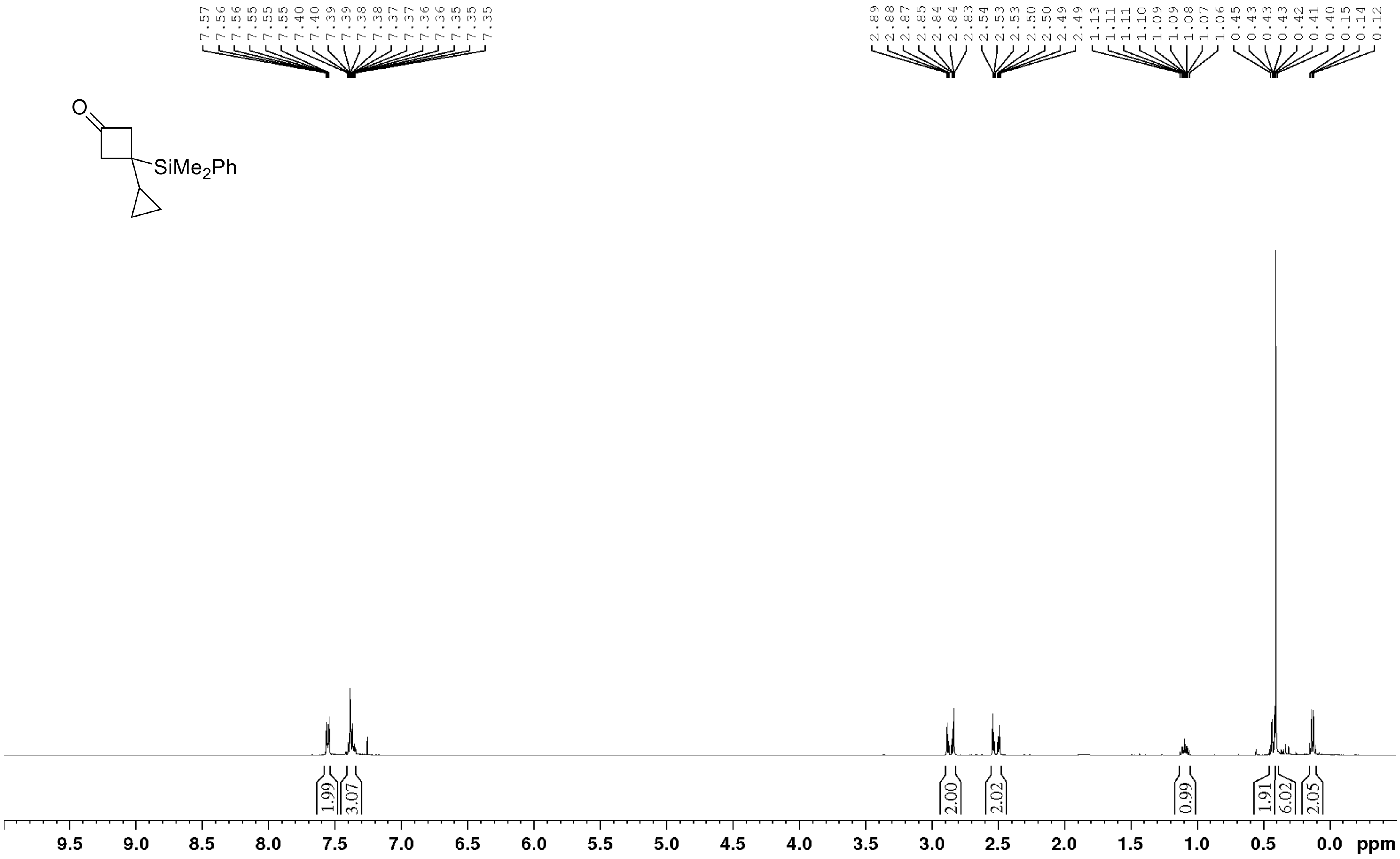

Figure S36. $^{13}C\{^{1}H\}$ NMR (101 MHz, $CDCl_3$, 298 K) of 3-Cyclopropyl-3-(dimethyl(phenyl)silyl)cyclobutan-1-one (**2l**)

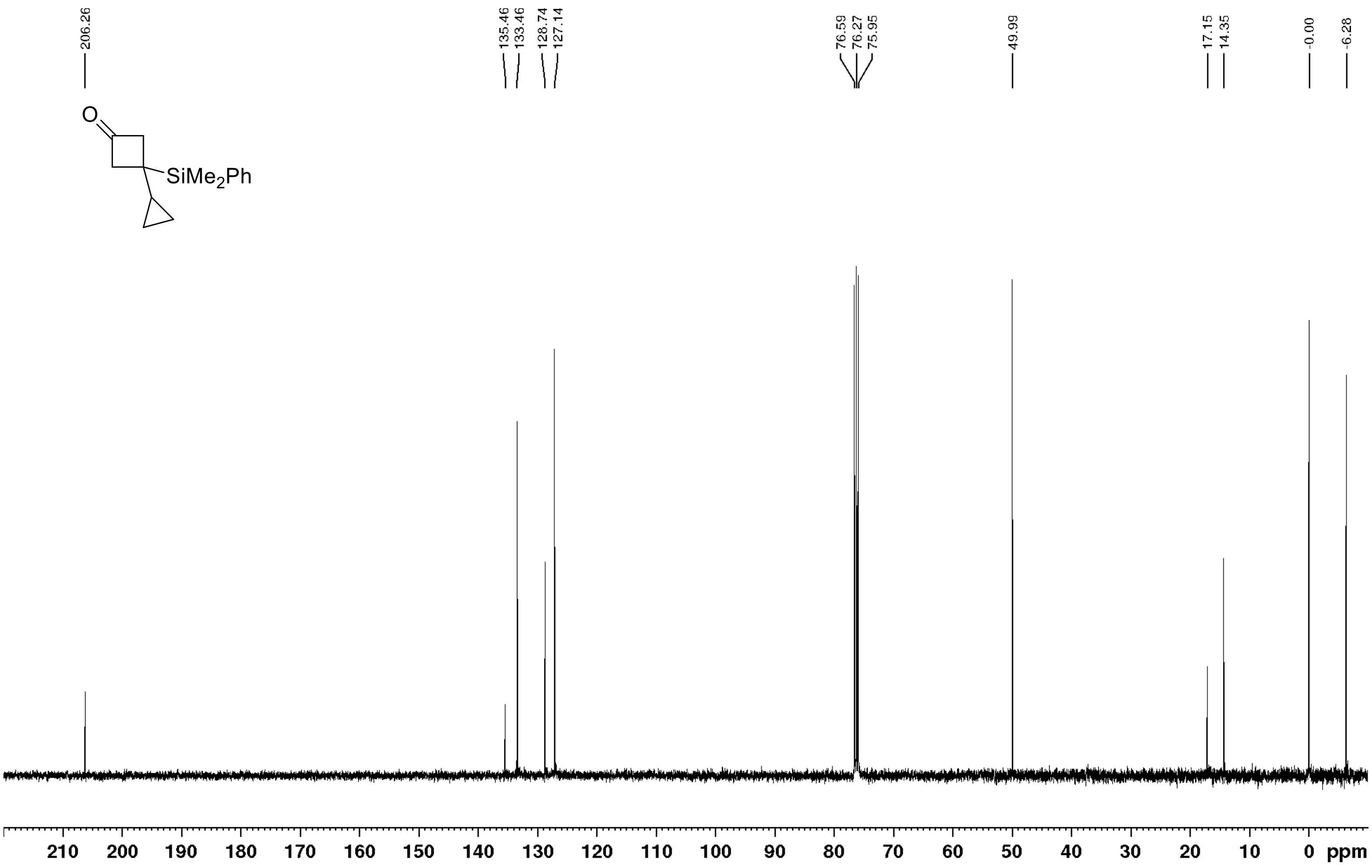

Figure S37. ^{29}Si{^{1}H} DEPT NMR (99 MHz, $CDCl_3$, 298 K) of 3-Cyclopropyl-3-(dimethyl(phenyl)silyl)cyclobutan-1-one (**2l**)

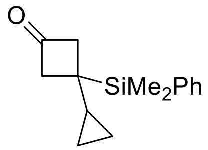

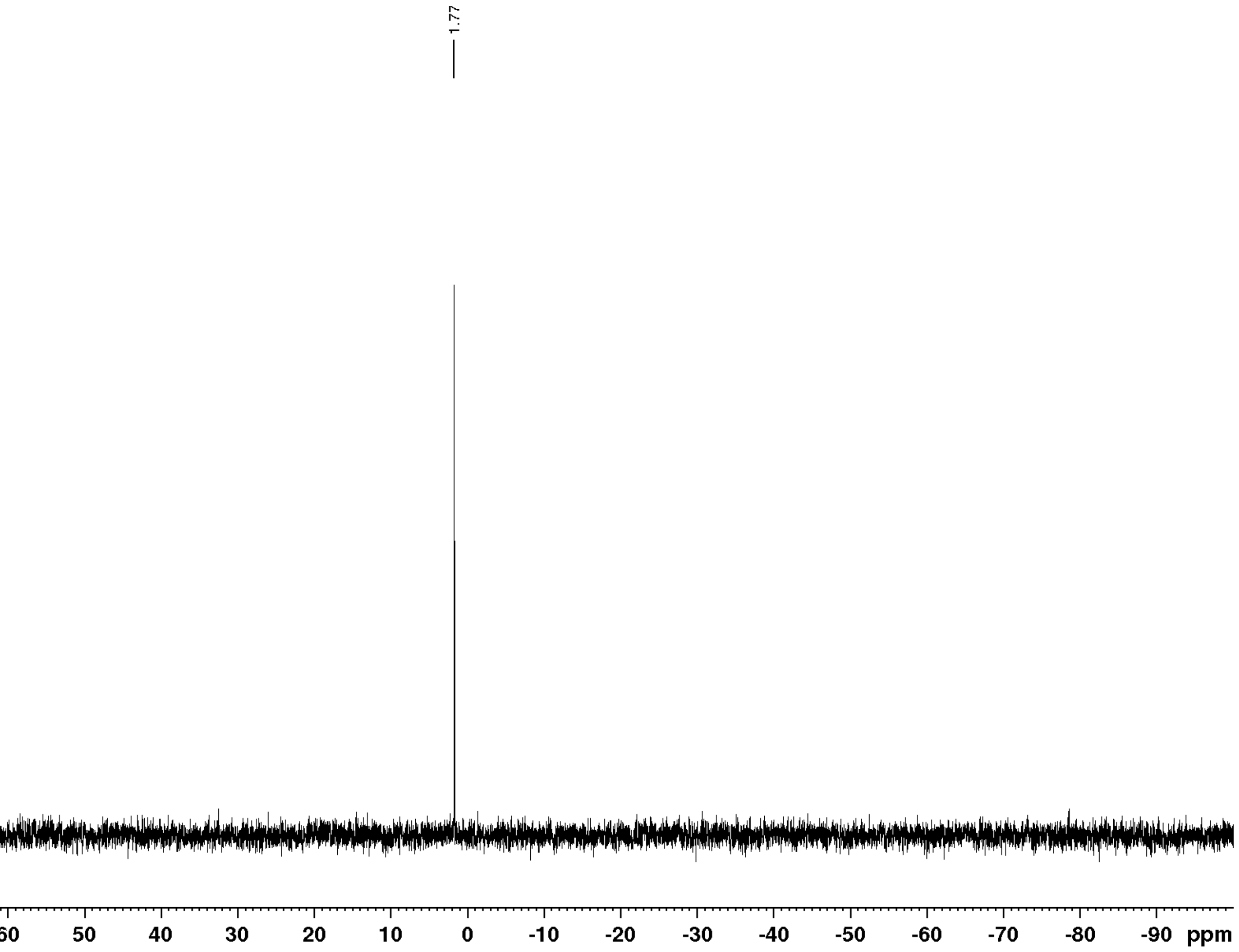

Figure S38. ^{1}H NMR spectrum (500 MHz, $CDCl_3$, 298 K) of 3-Cyclohexyl-3-(dimethyl(phenyl)silyl)cyclobutan-1-one (**2m**)

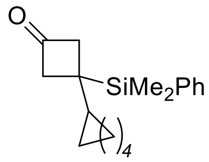

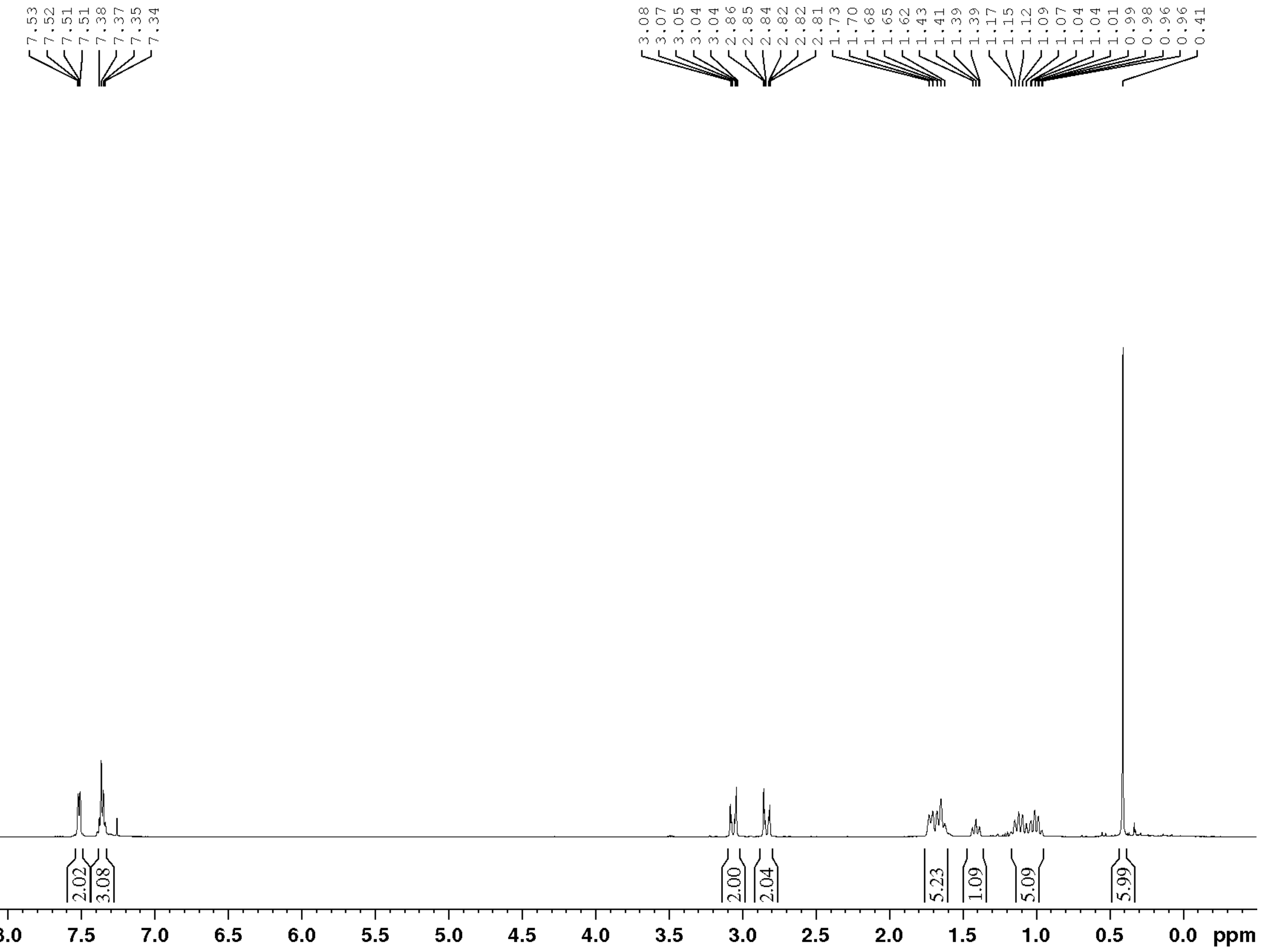

Figure S39. $^{13}C\{^1H\}$ NMR (126 MHz, $CDCl_3$, 298 K) of 3-Cyclohexyl-3-(dimethyl(phenyl)silyl)cyclobutan-1-one (**2m**)

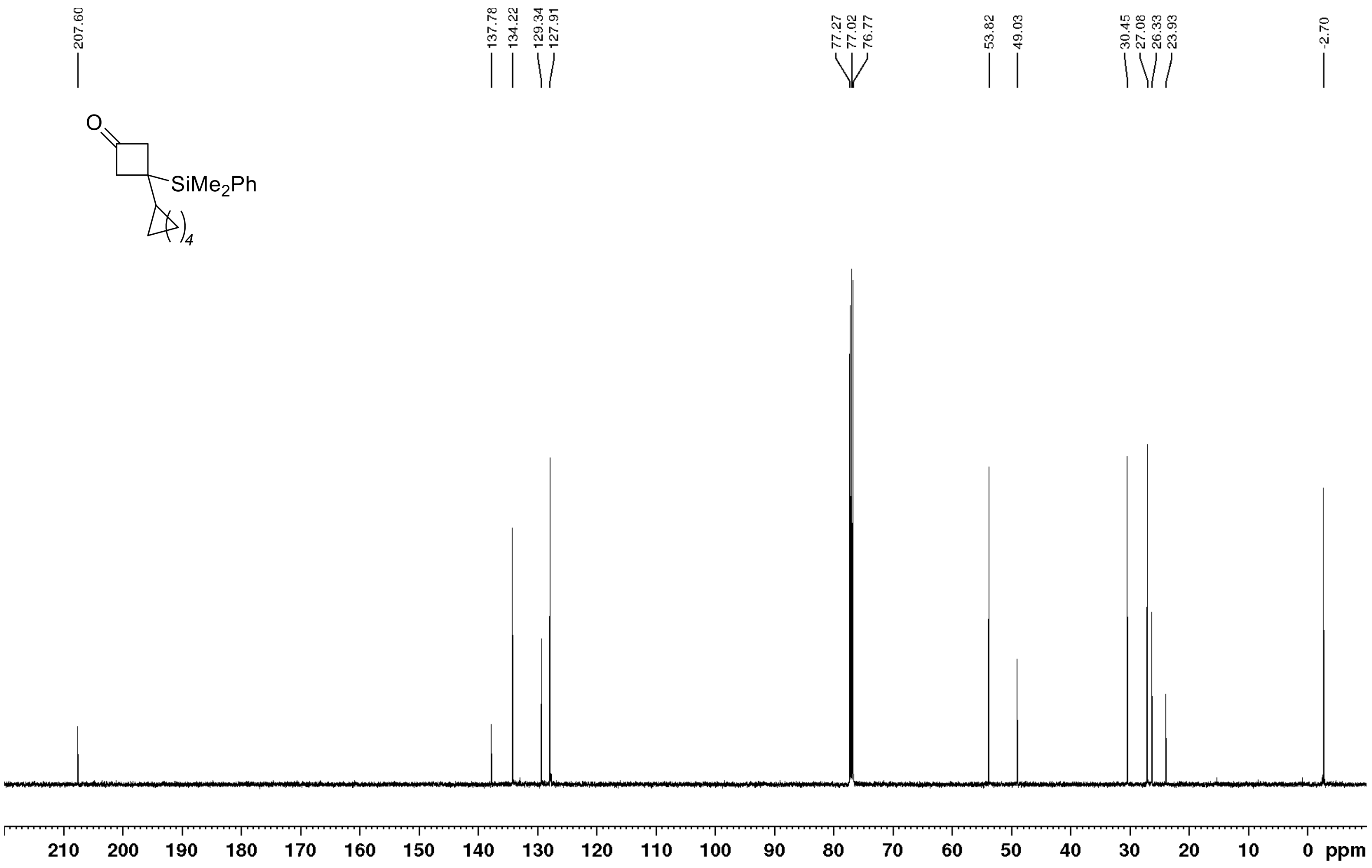

Figure S40. $^{29}Si\{^1H\}$ DEPT NMR (99 MHz, $CDCl_3$, 298 K) of 3-Cyclohexyl-3-(dimethyl(phenyl)silyl)cyclobutan-1-one (**2m**)

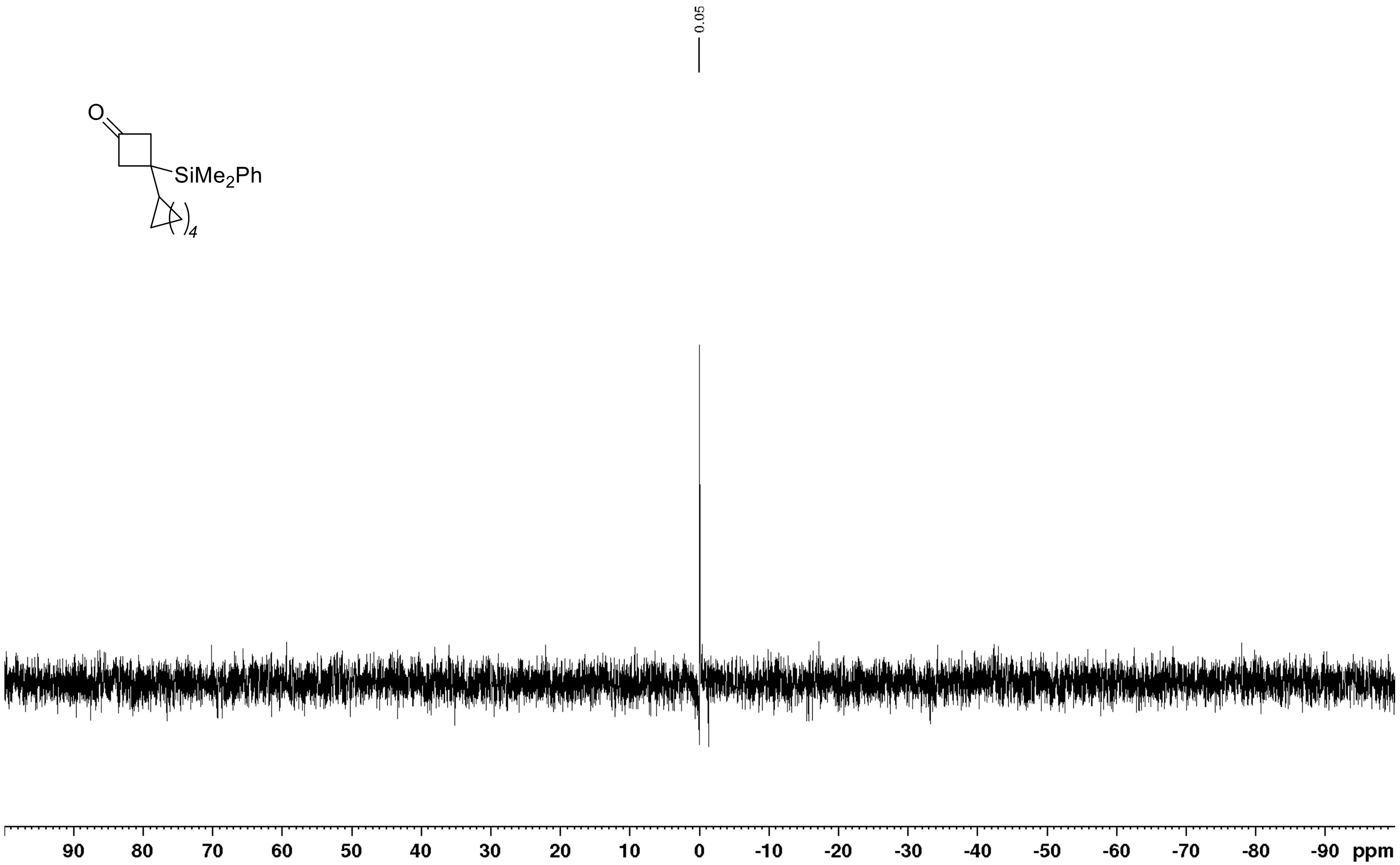

Figure S41. ^{1}H NMR spectrum (500 MHz, $CDCl_3$, 298 K) of 3-(Dimethyl(phenyl)silyl)-3-(trimethylsilyl)cyclobutan-1-one (**2n**)

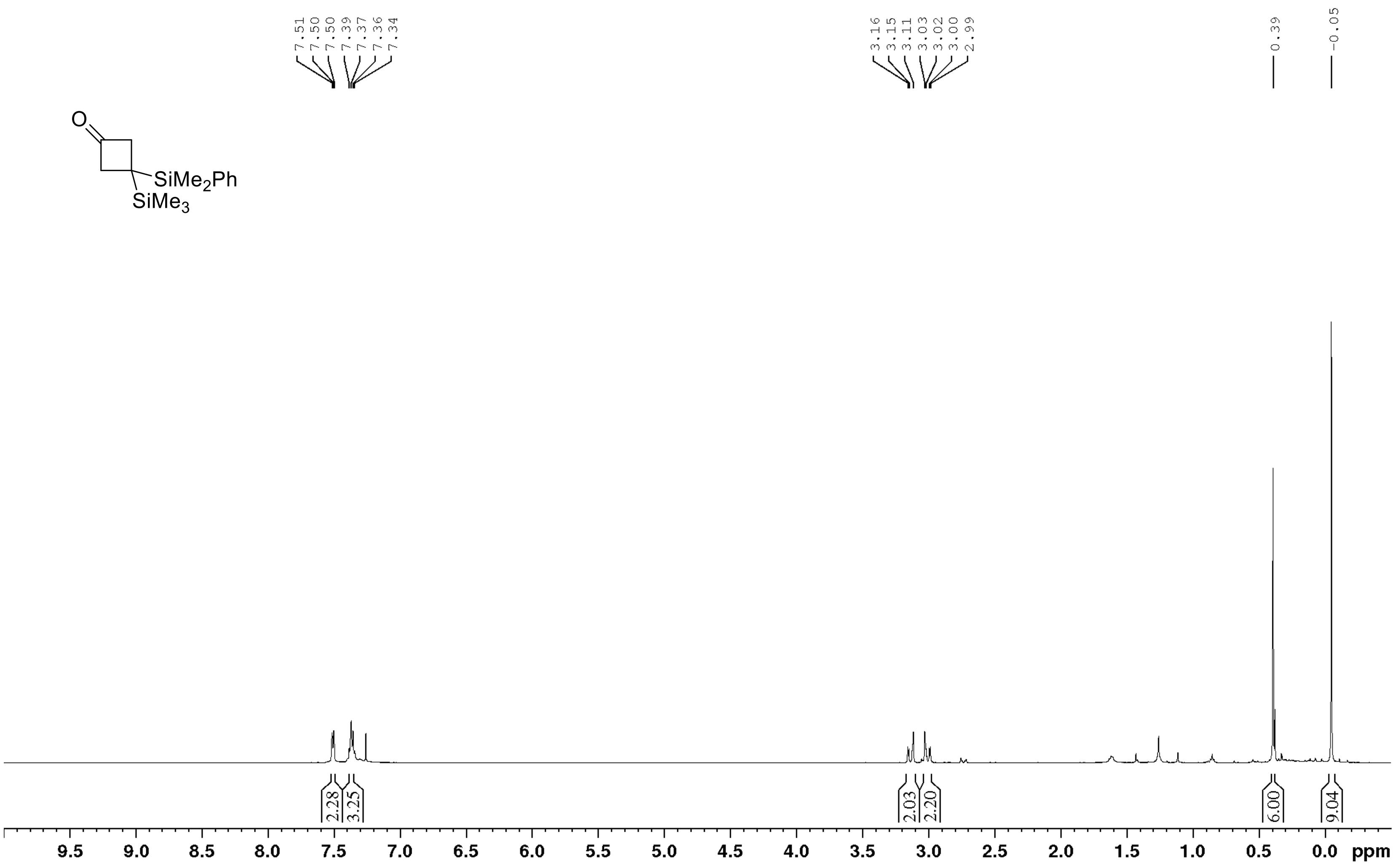

Figure S42. $^{13}C\{^{1}H\}$ NMR (126 MHz, $CDCl_3$, 298 K) of 3-(Dimethyl(phenyl)silyl)-3-(trimethylsilyl)cyclobutan-1-one (**2n**)

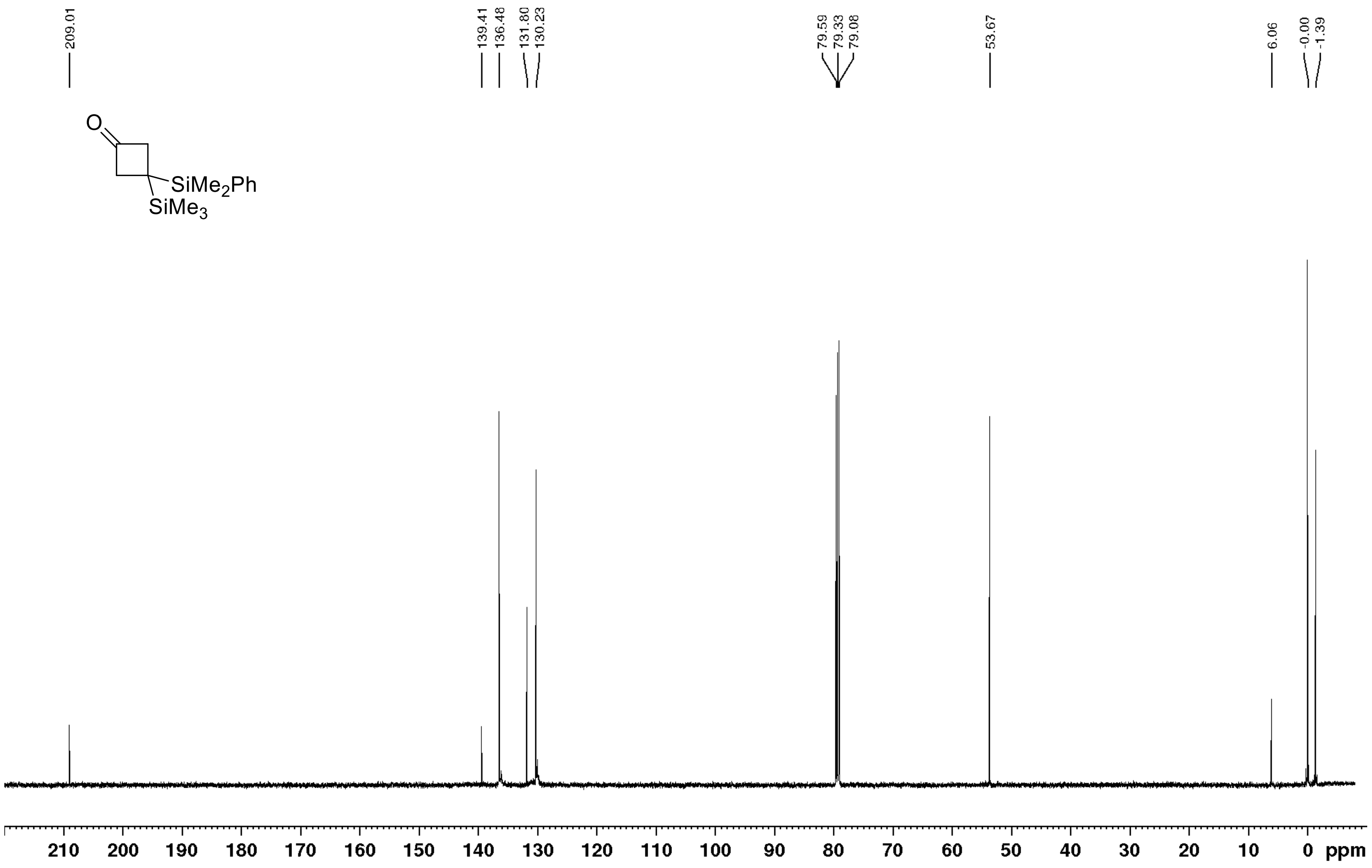

Figure S43. ^{1}H-^{29}Si HMQC NMR (99 MHz, $CDCl_3$, 298 K) of 3-(Dimethyl(phenyl)silyl)-3-(trimethylsilyl)cyclobutan-1-one (**2n**)

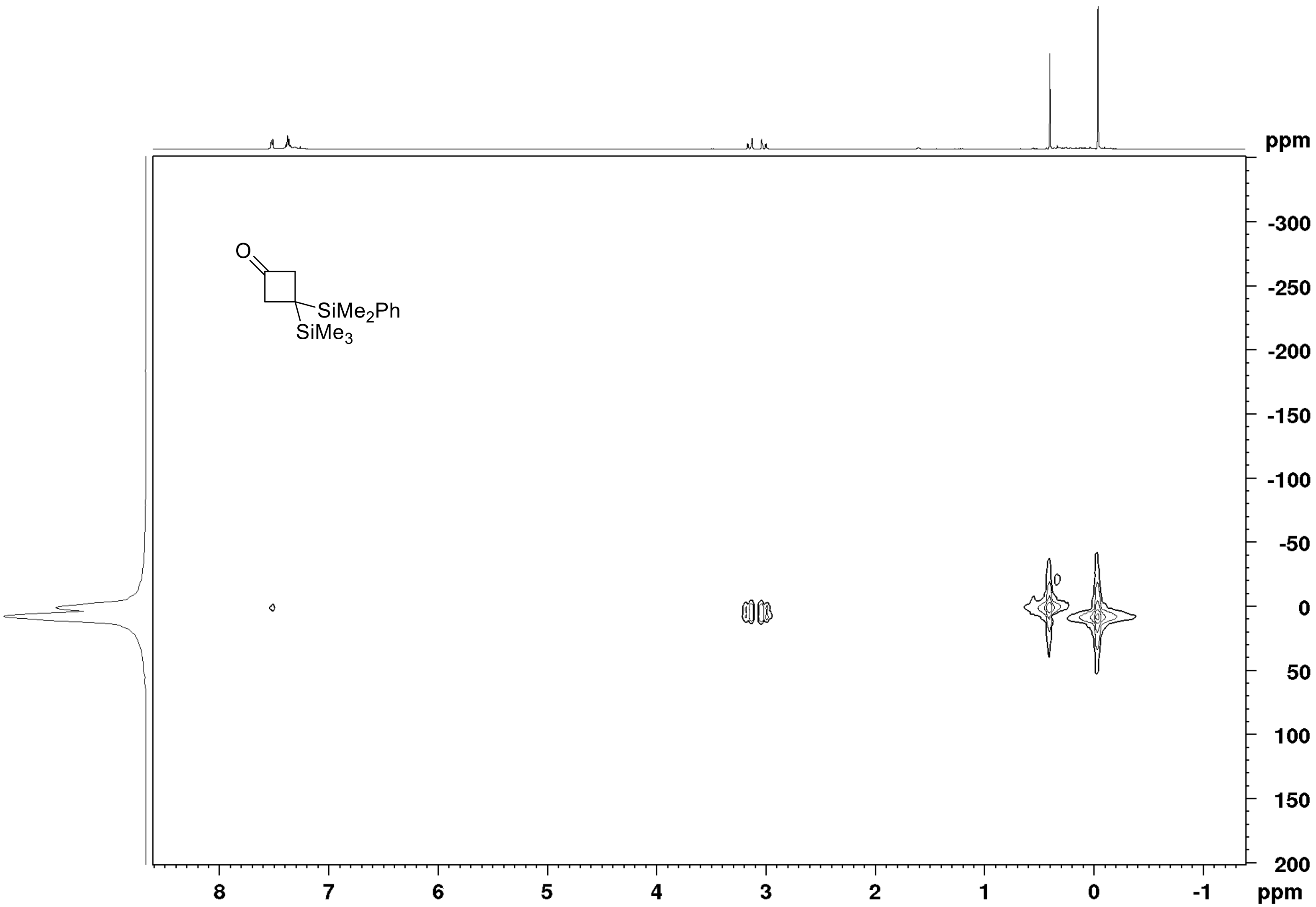

Figure S44. ^{1}H NMR spectrum (500 MHz, $CDCl_3$, 298 K) of 3-(Dimethyl(phenyl)silyl)-2-methyl-3-phenylcyclobutan-1-one (**2o**)

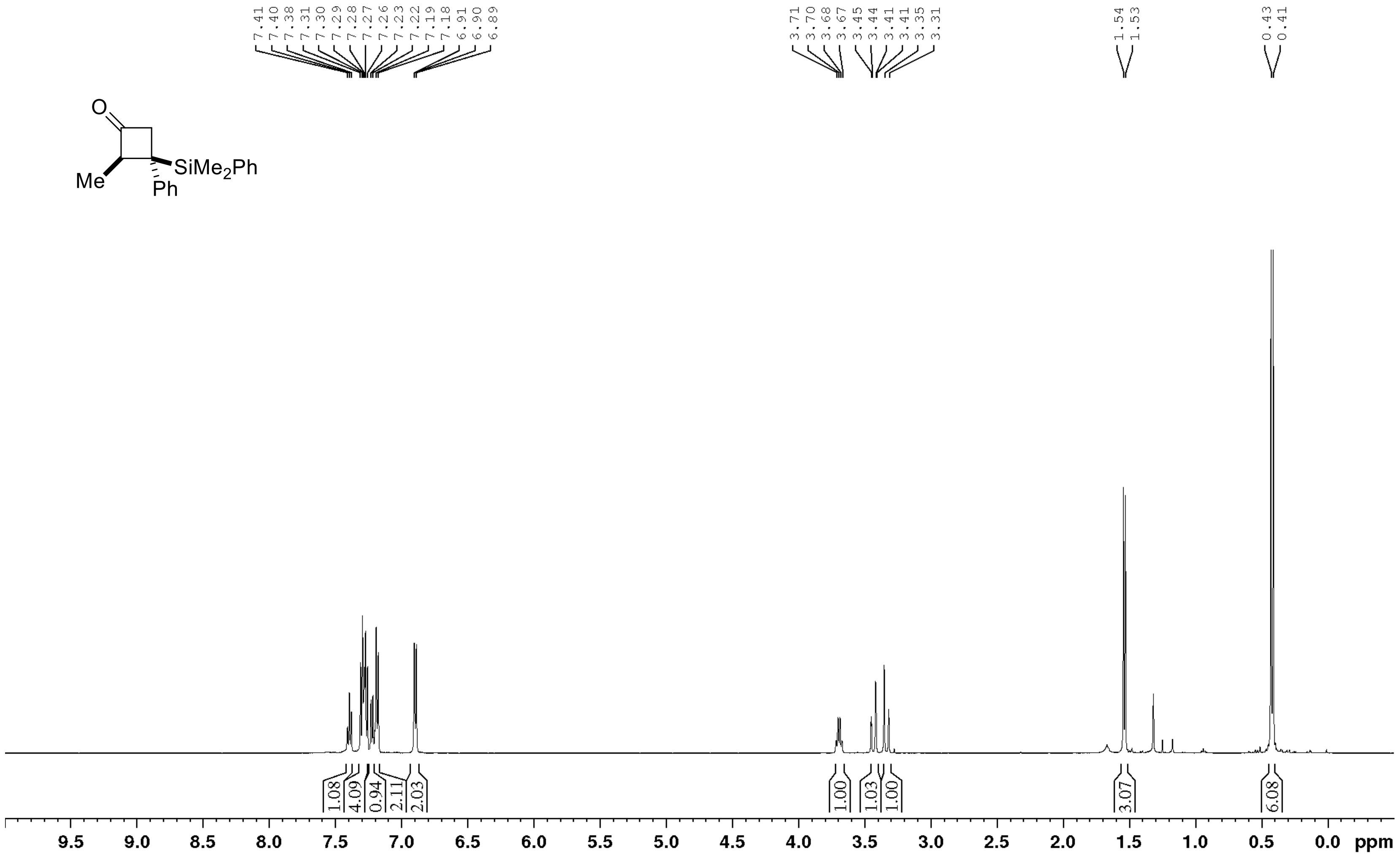

Figure S45. $^{13}C\{^{1}H\}$ NMR (126 MHz, $CDCl_3$, 298 K) of 3-(Dimethyl(phenyl)silyl)-2-methyl-3-phenylcyclobutan-1-one (**2o**)

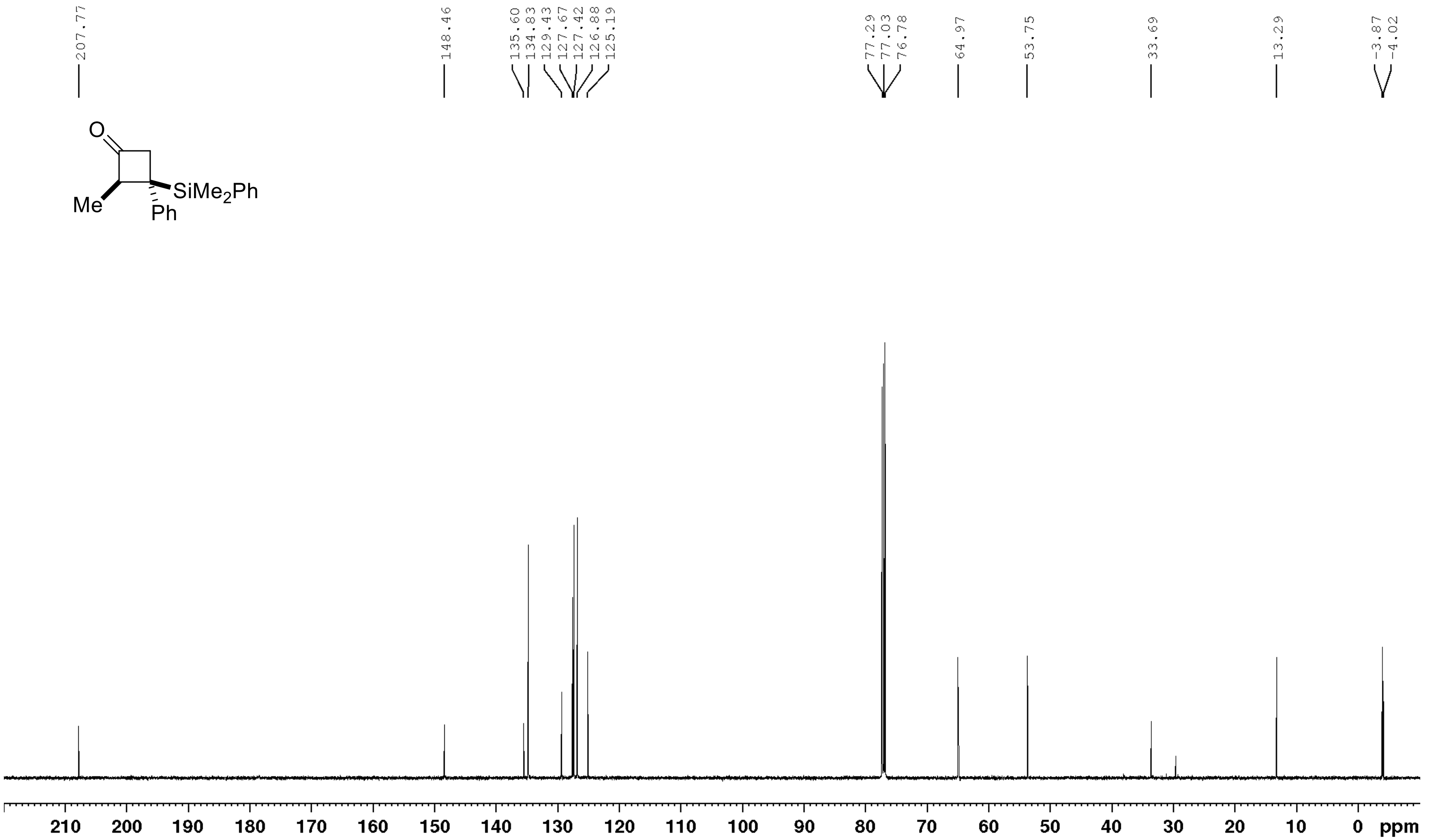

Figure S46. $^{29}Si\{^1H\}$ DEPT NMR (99 MHz, $CDCl_3$, 298 K) of 3-(Dimethyl(phenyl)silyl)-2-methyl-3-phenylcyclobutan-1-one (**2o**)

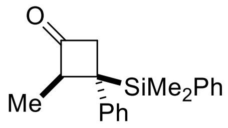

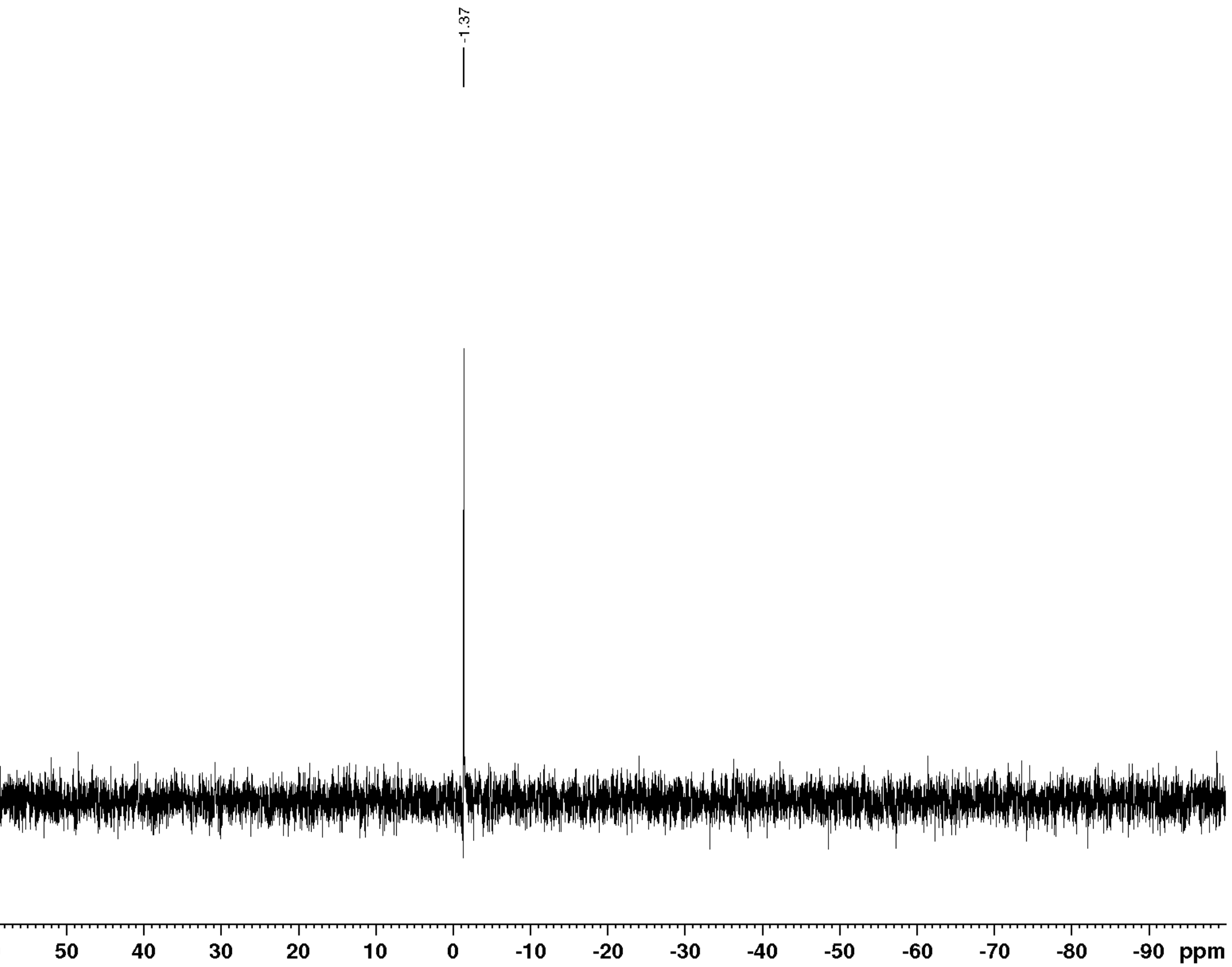

Figure S47. ^{1}H NMR spectrum (500 MHz, $CDCl_3$, 298 K) of 3-(Dimethyl(phenyl)silyl)-2,3-dipropylcyclobutan-1-one (**2p**)

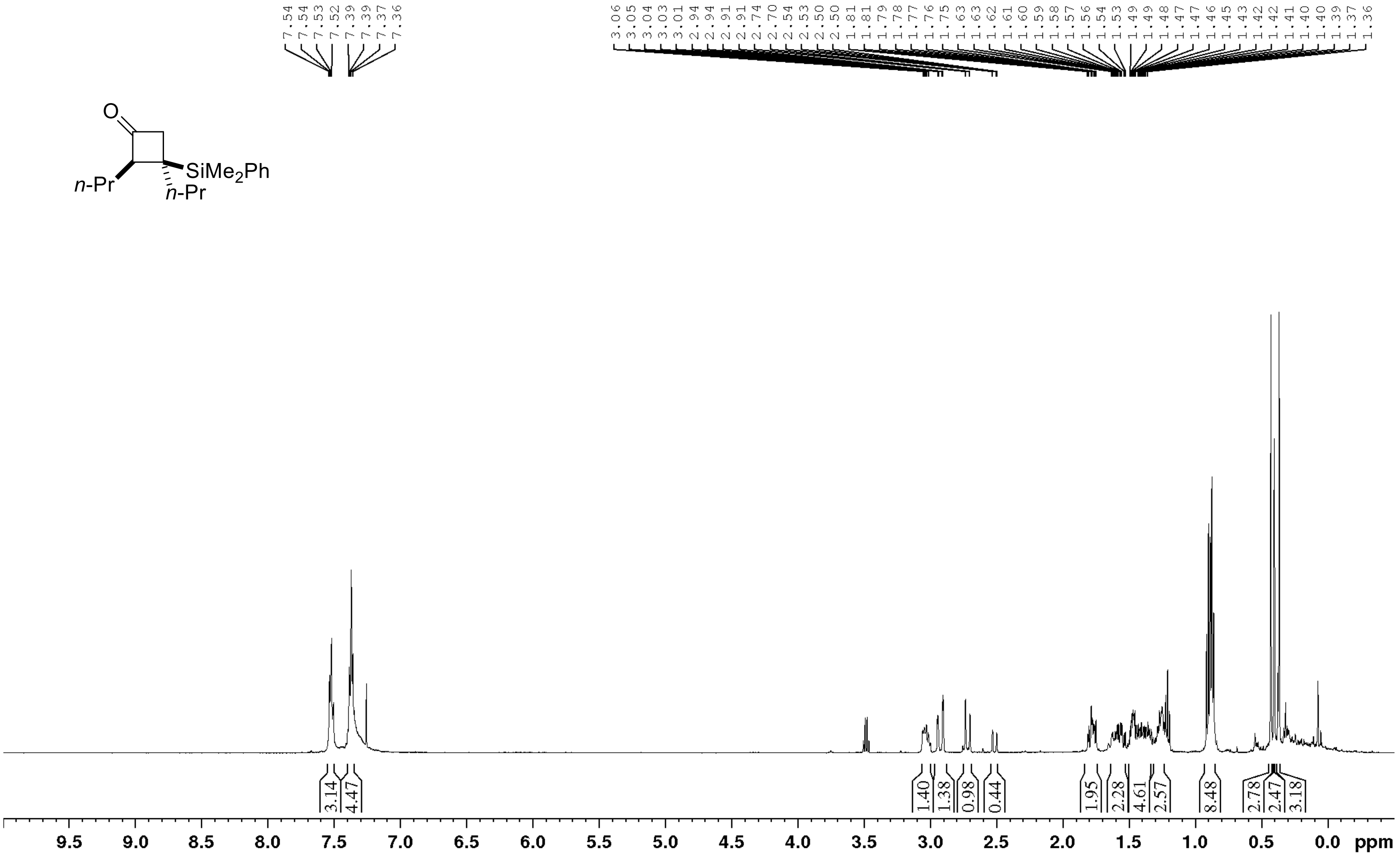

Figure S48. $^{13}C\{^1H\}$ NMR (126 MHz, $CDCl_3$, 298 K) of 3-(Dimethyl(phenyl)silyl)-2,3-dipropylcyclobutan-1-one (**2p**)

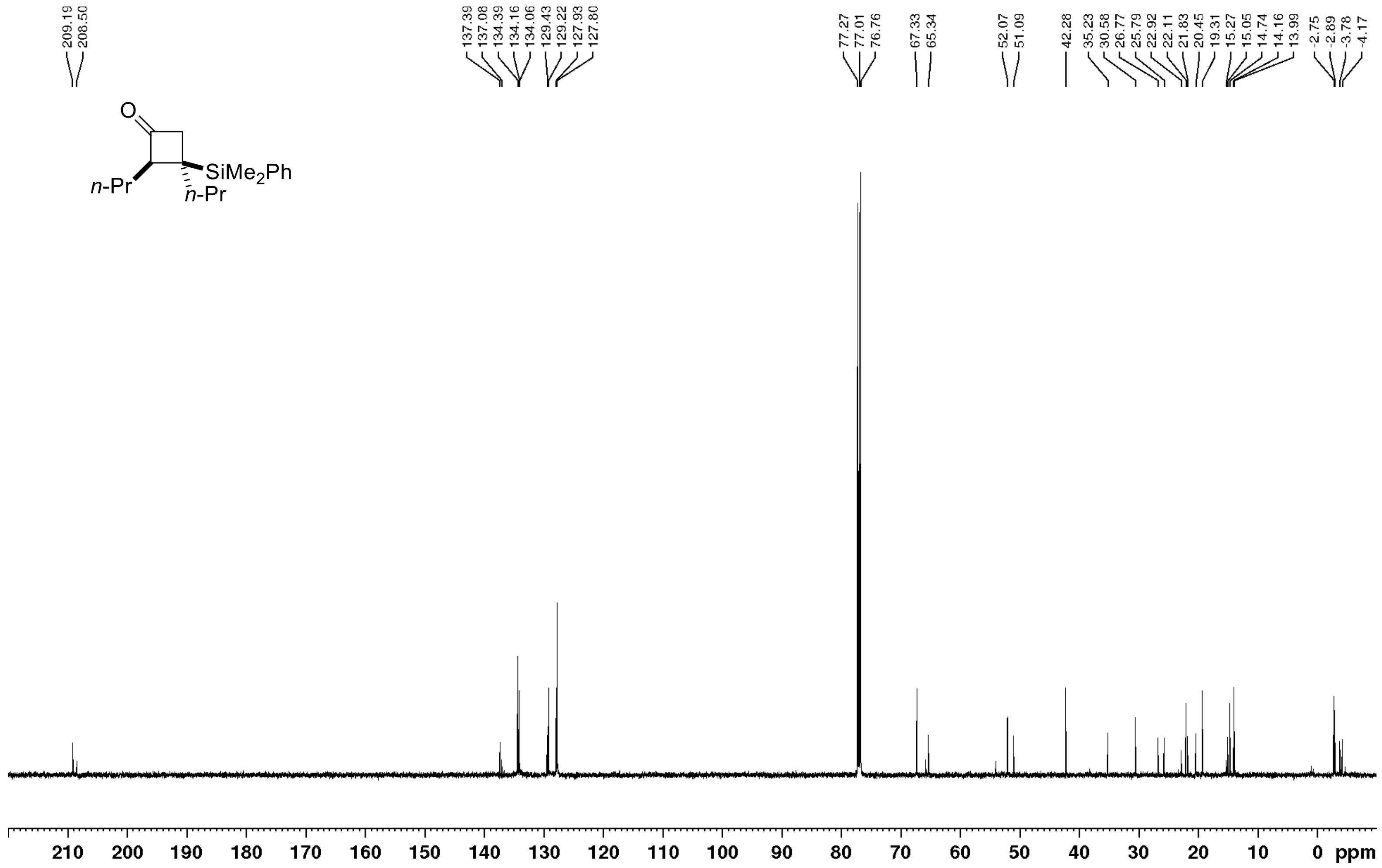

Figure S49. $^{29}Si\{^{1}H\}$ DEPT NMR (99 MHz, $CDCl_3$, 298 K) of 3-(Dimethyl(phenyl)silyl)-2,3-dipropylcyclobutan-1-one (**2p**)

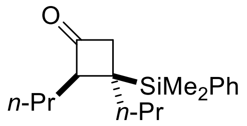

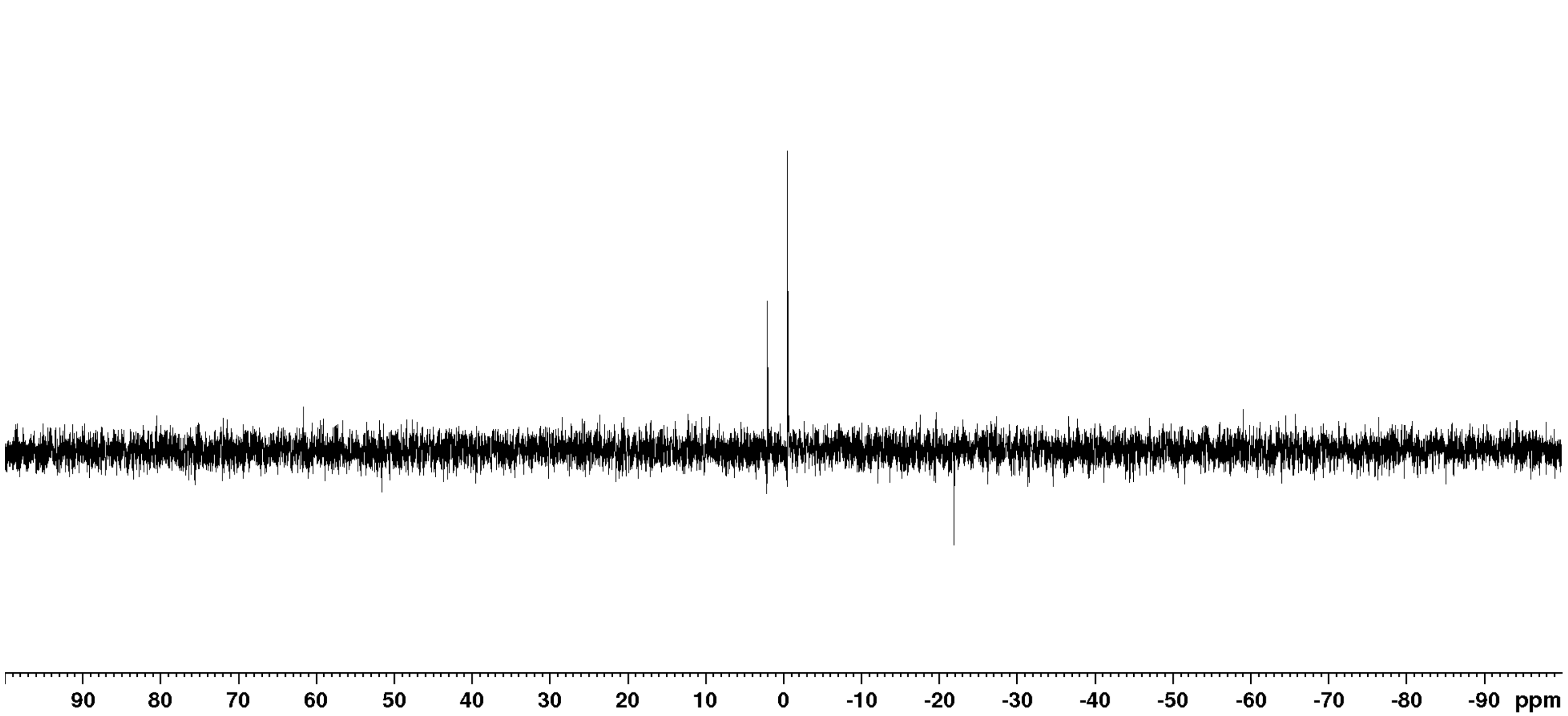

Figure S50. ^{1}H NMR spectrum (500 MHz, $CDCl_3$, 298 K) of 3-(Dimethyl(phenyl)silyl)-3-phenylcyclobut-1-en-1-yl diphenyl phosphate (**3a**)

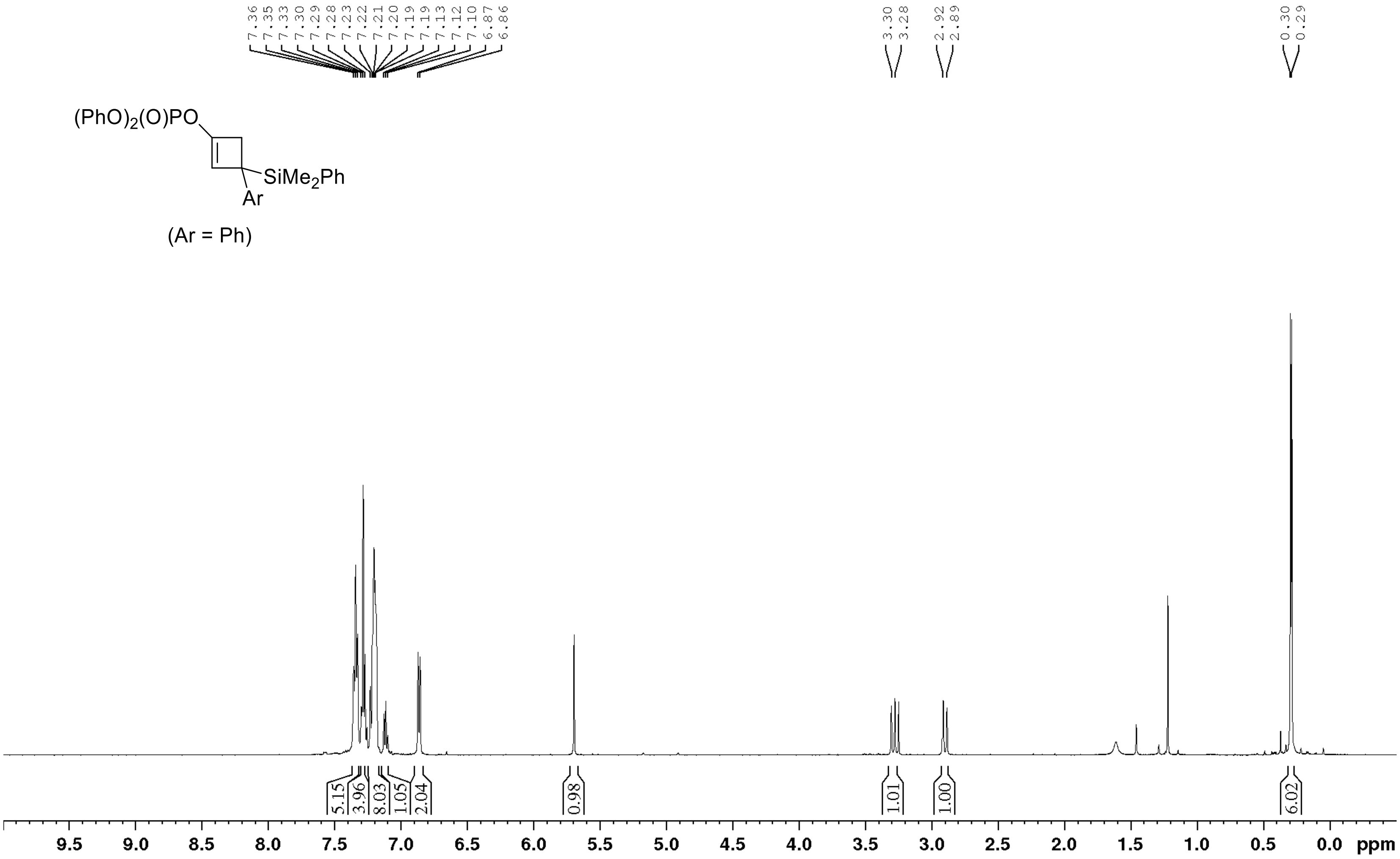

Figure S51. $^{13}C\{^{1}H\}$ NMR (126 MHz, $CDCl_3$, 298 K) of 3-(Dimethyl(phenyl)silyl)-3-phenylcyclobut-1-en-1-yl diphenyl phosphate (**3a**)

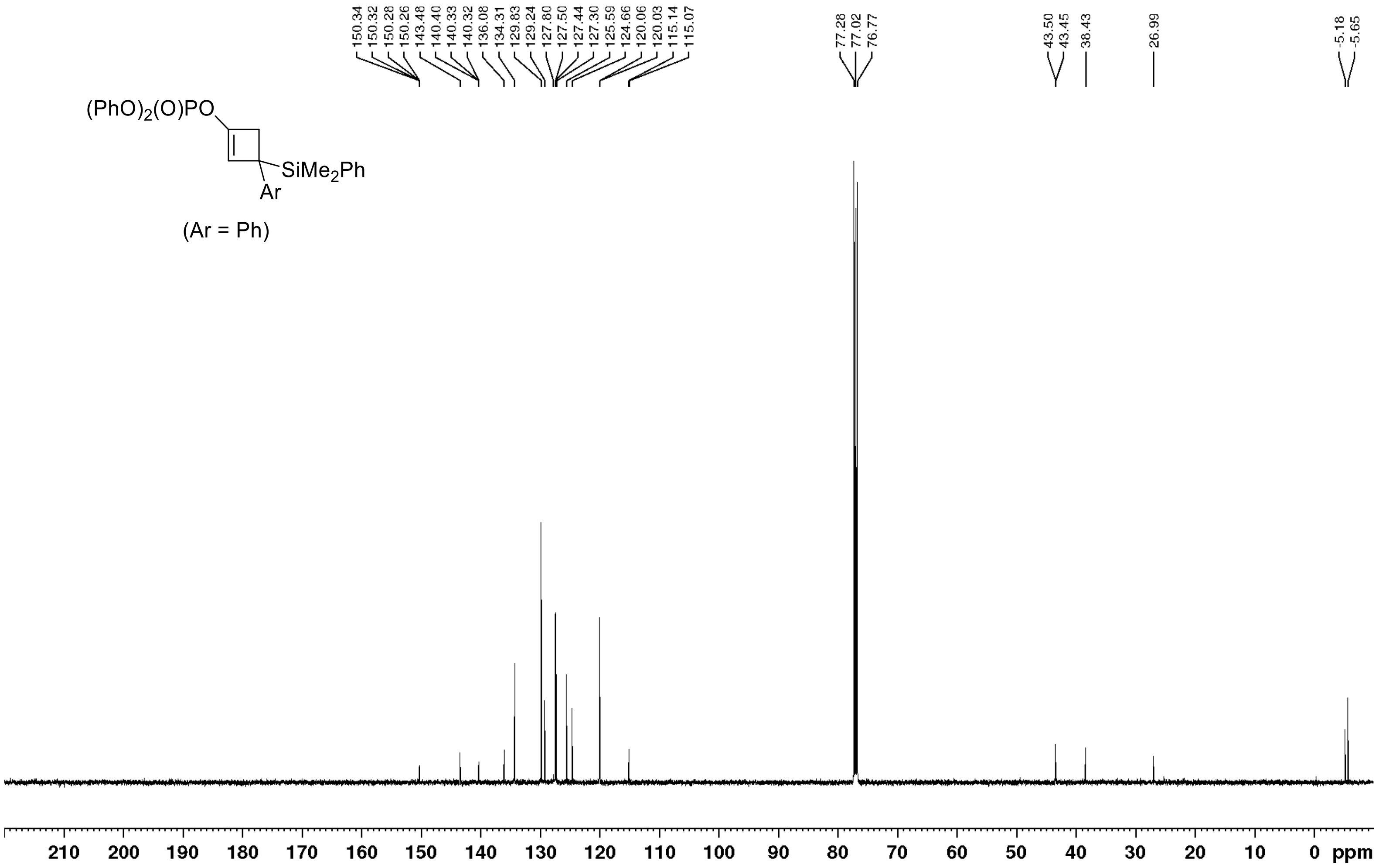

Figure S52. $^{29}Si\{^1H\}$ DEPT NMR (99 MHz, $CDCl_3$, 298 K) of 3-(Dimethyl(phenyl)silyl)-3-phenylcyclobut-1-en-1-yl diphenyl phosphate (**3a**)

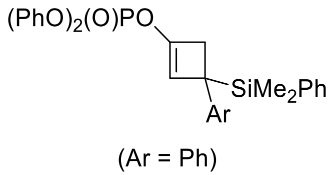

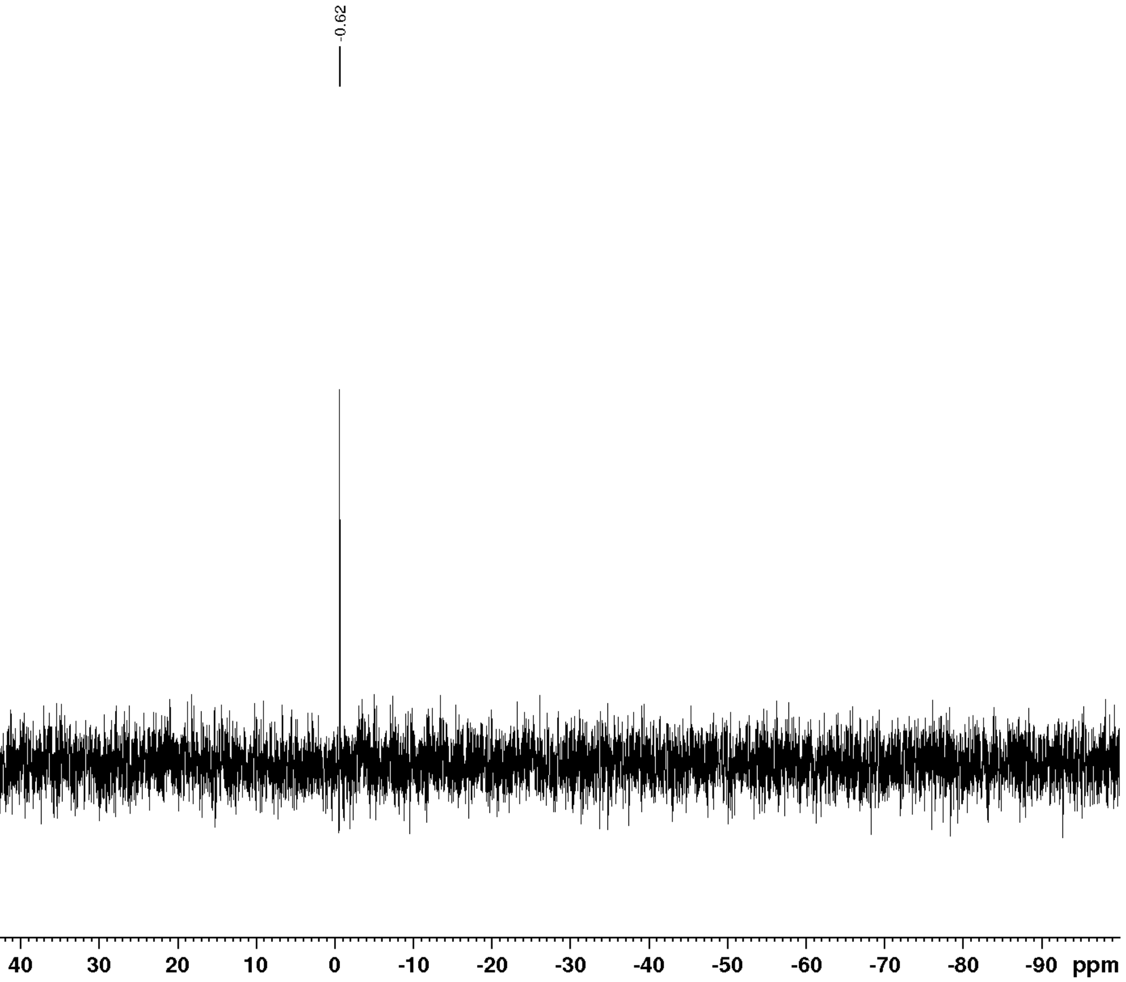

Figure S53. $^{31}P\{^1H\}$ NMR (202 MHz, $CDCl_3$, 298 K) of 3-(Dimethyl(phenyl)silyl)-3-phenylcyclobut-1-en-1-yl diphenyl phosphate (**3a**)

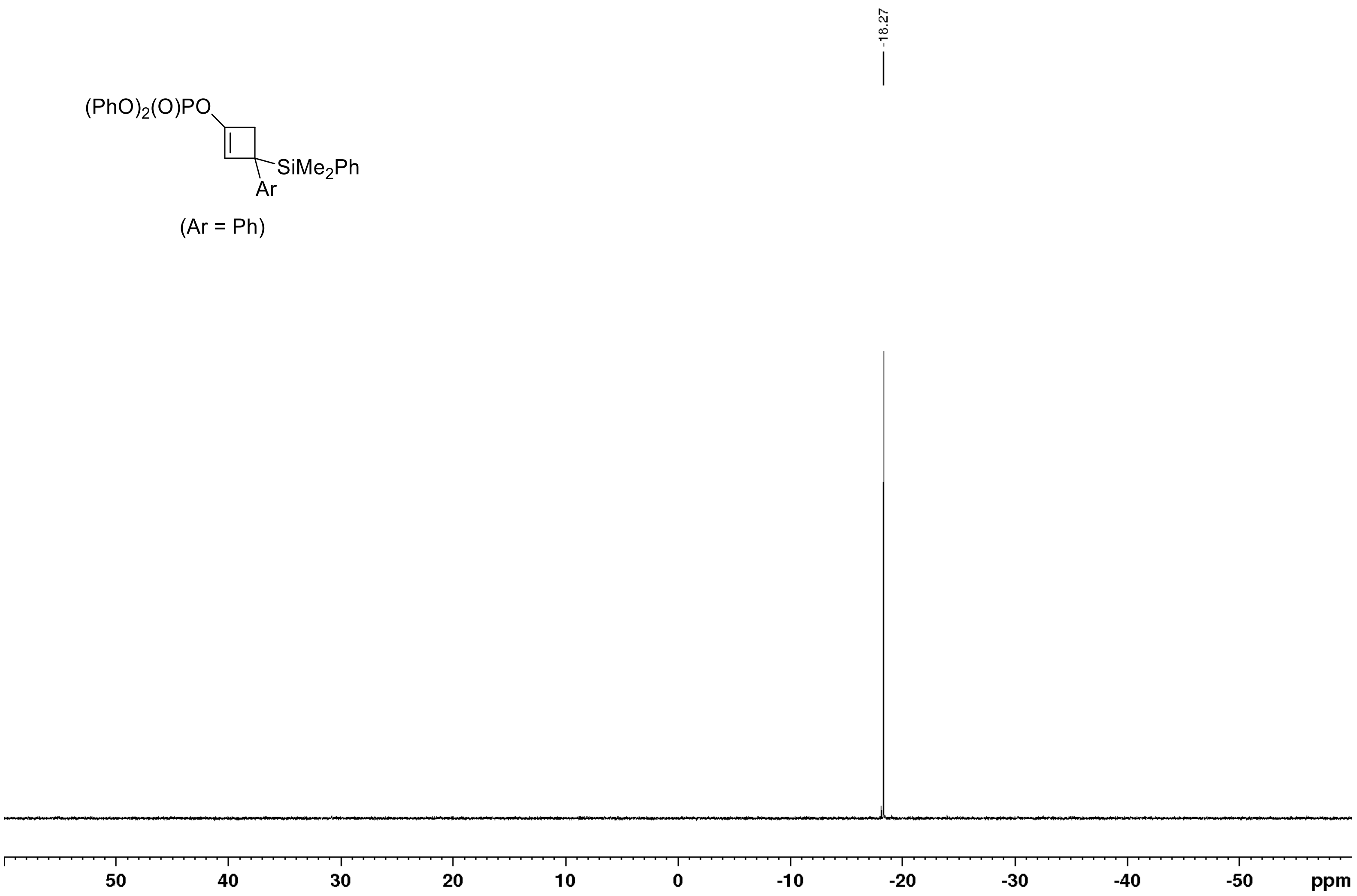

Figure S54. ^{1}H NMR spectrum (500 MHz, $CDCl_3$, 298 K) of 3-(Dimethyl(phenyl)silyl)-3-phenylcyclobut-1-en-1-yl diphenyl phosphate (**3b**) (# = Cyclohexane)

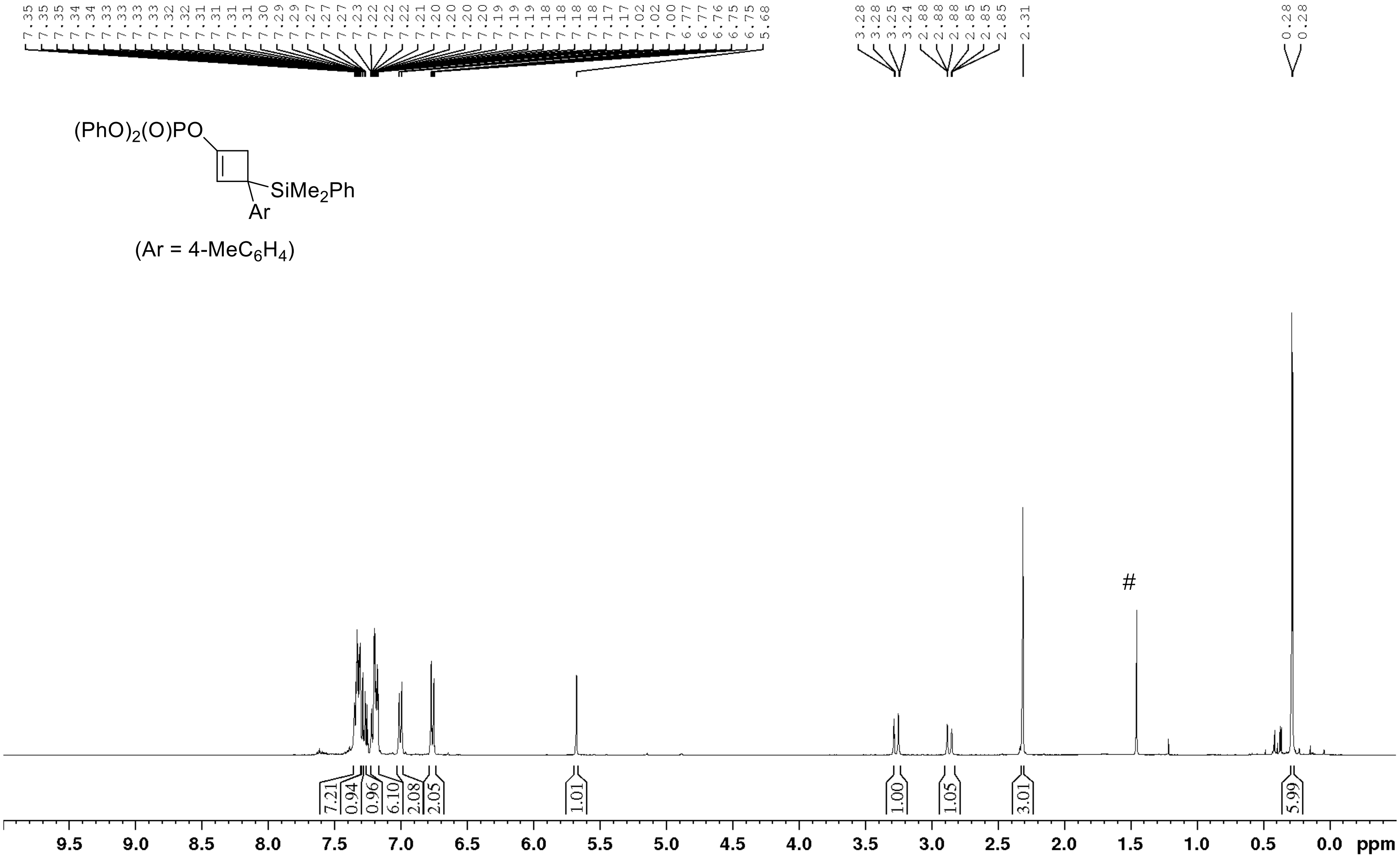

Figure S55. $^{13}C\{^{1}H\}$ NMR spectrum (126 MHz, $CDCl_3$, 298 K) of 3-(Dimethyl(phenyl)silyl)-3-phenylcyclobut-1-en-1-yl diphenyl phosphate (**3b**)

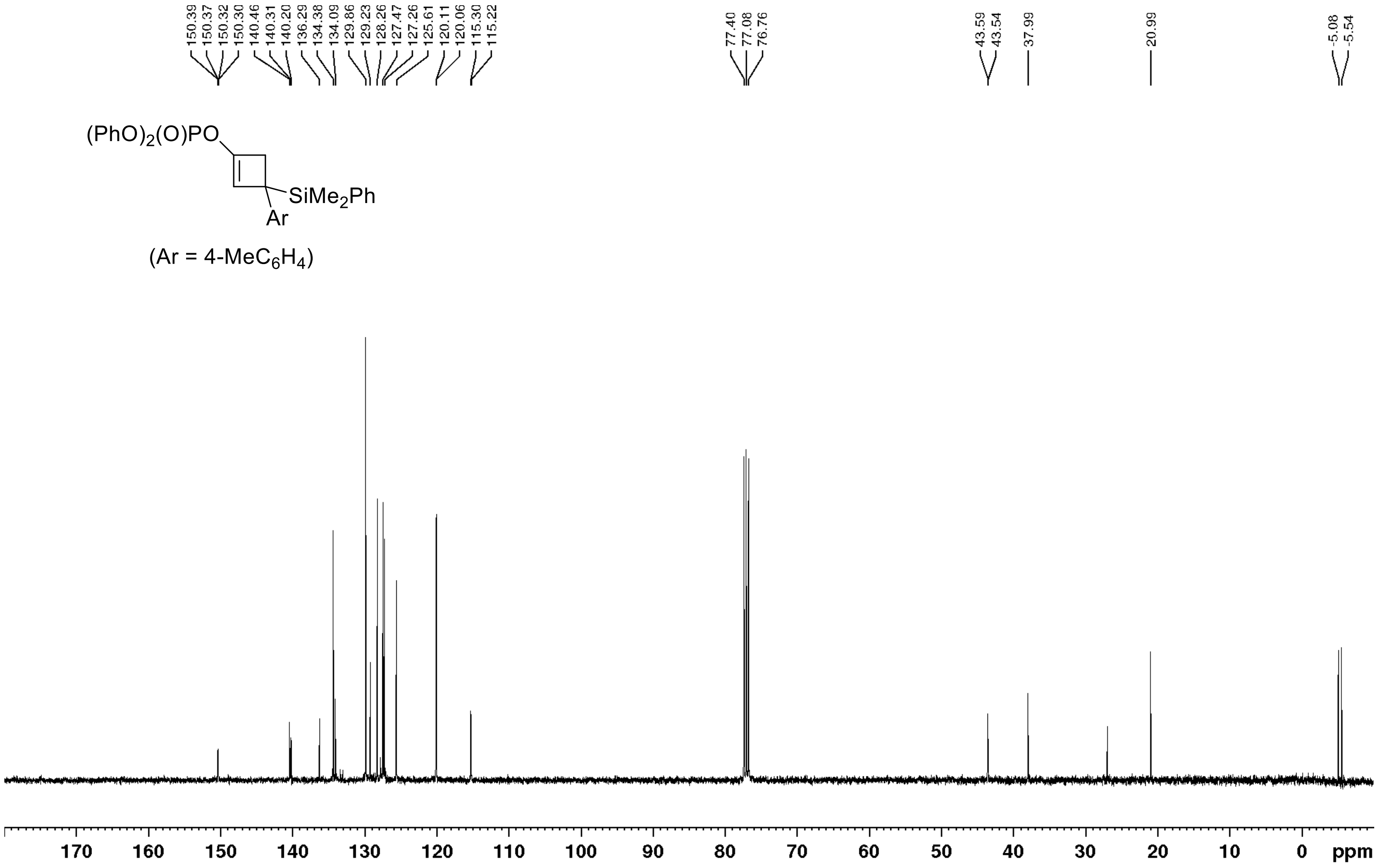

Figure S56. $^{29}Si\ \{^{1}H\}$ DEPT NMR spectrum (99 MHz, $CDCl_3$) of 3-(Dimethyl(phenyl)silyl)-3-phenylcyclobut-1-en-1-yl diphenyl phosphate (**3b**)

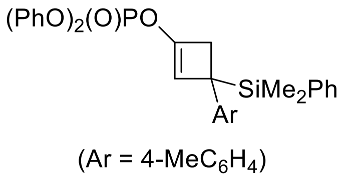

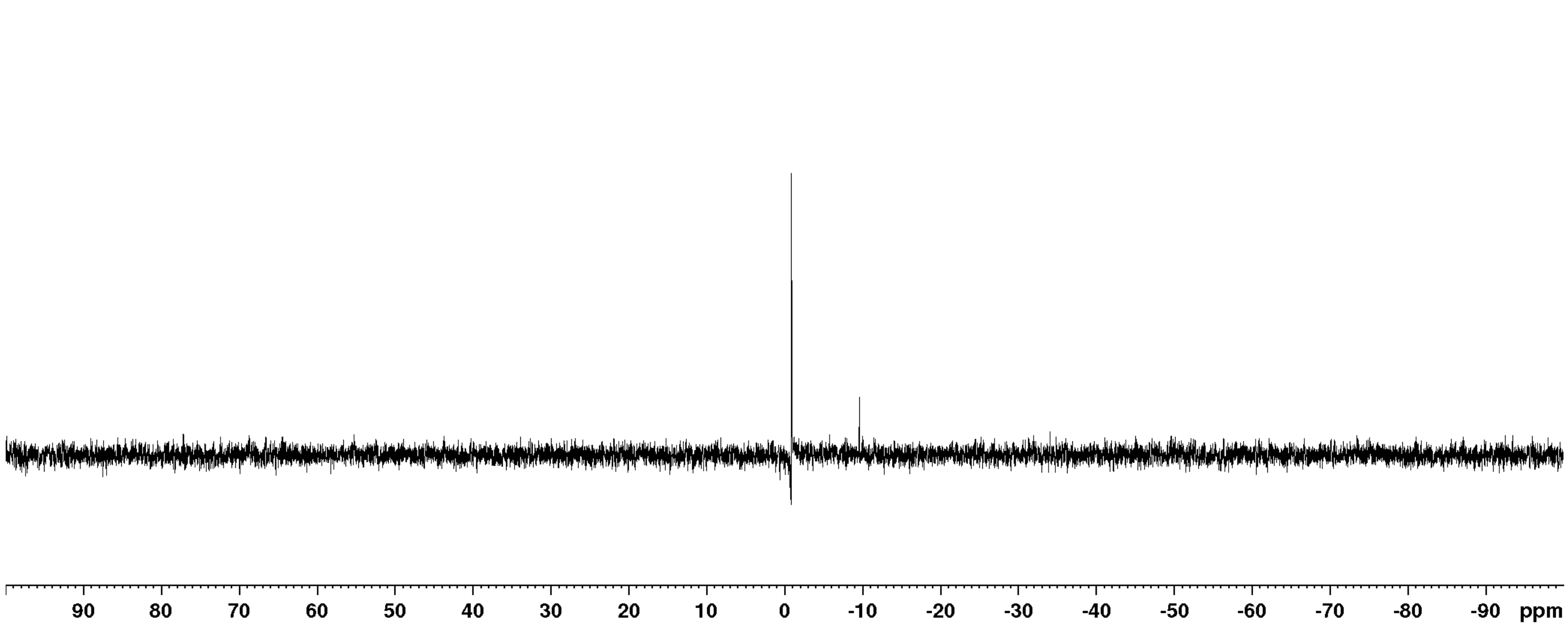

Figure S57. $^{31}P\{^{1}H\}$ NMR (202 MHz, $CDCl_3$, 298 K) of 3-(Dimethyl(phenyl)silyl)-3-phenylcyclobut-1-en-1-yl diphenyl phosphate (**3b**)

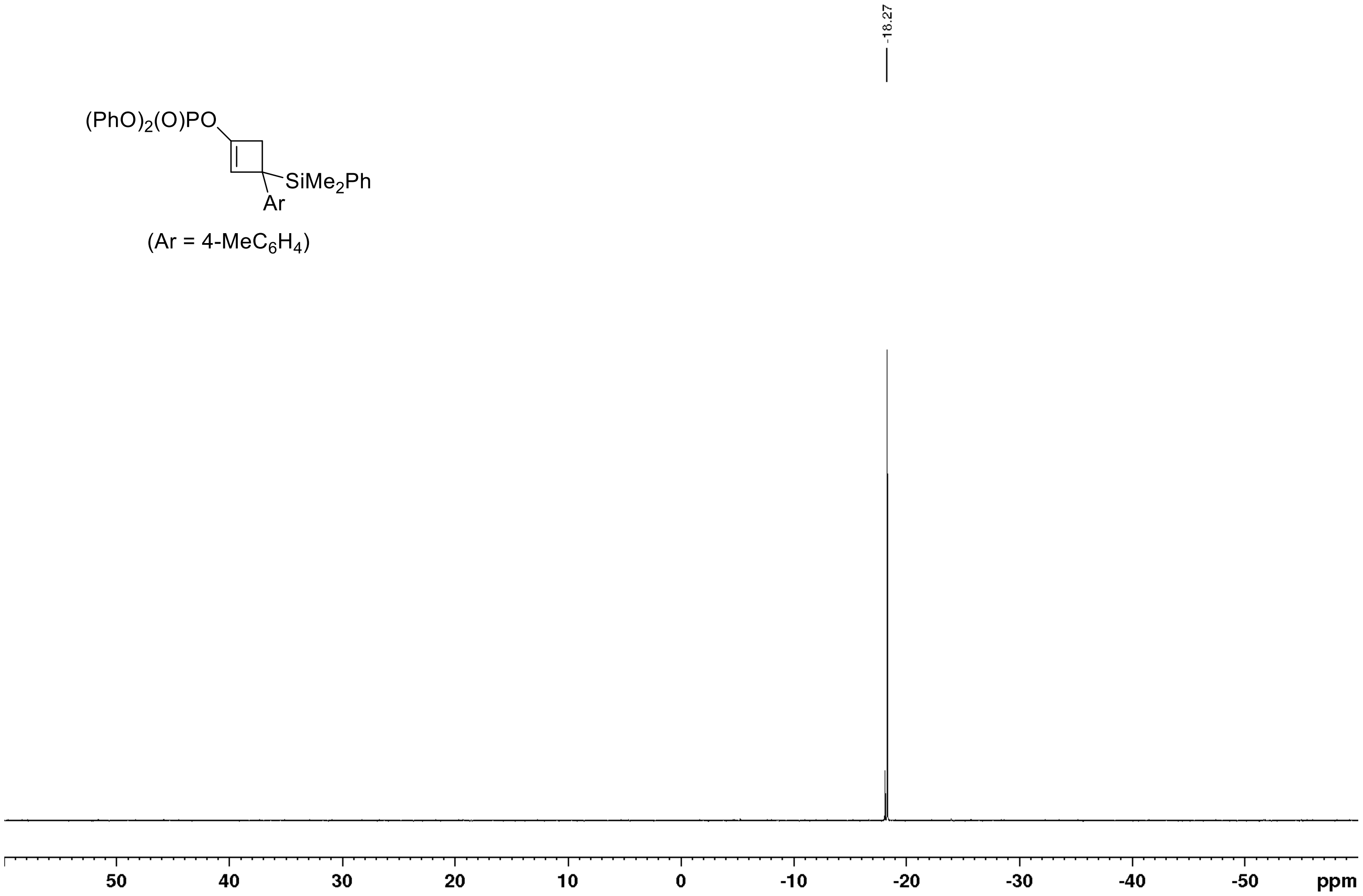

Figure S58. ^{1}H NMR spectrum (400 MHz, $CDCl_3$, 298 K) of 3-(Dimethyl(phenyl)silyl)-3-(m-tolyl)cyclobut-1-en-1-yl diphenyl phosphate (**3c**)

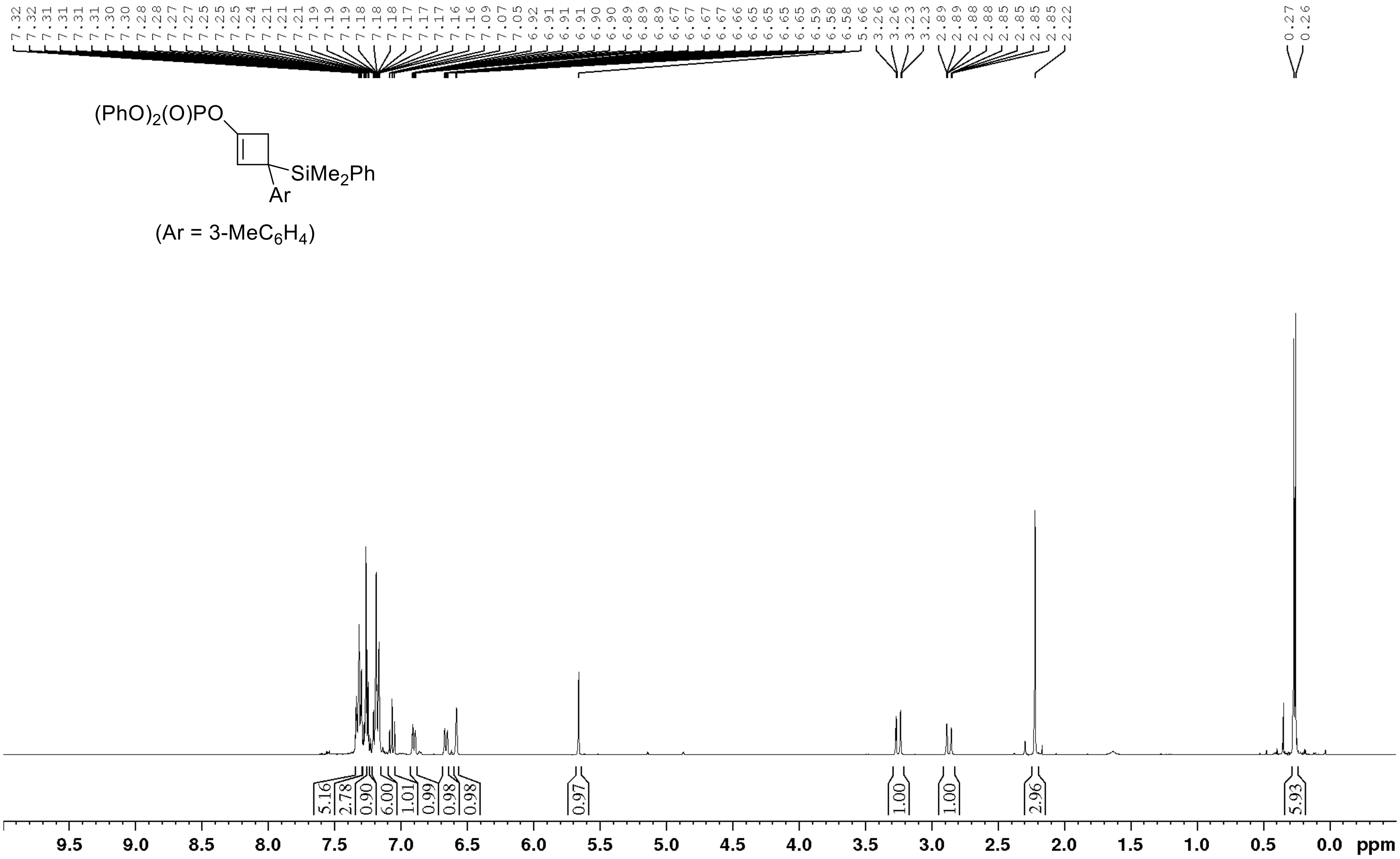

Figure S59. $^{13}C\{^{1}H\}$ NMR spectrum (101 MHz, $CDCl_3$, 298 K) of 3-(Dimethyl(phenyl)silyl)-3-(m-tolyl)cyclobut-1-en-1-yl diphenyl phosphate (**3c**)

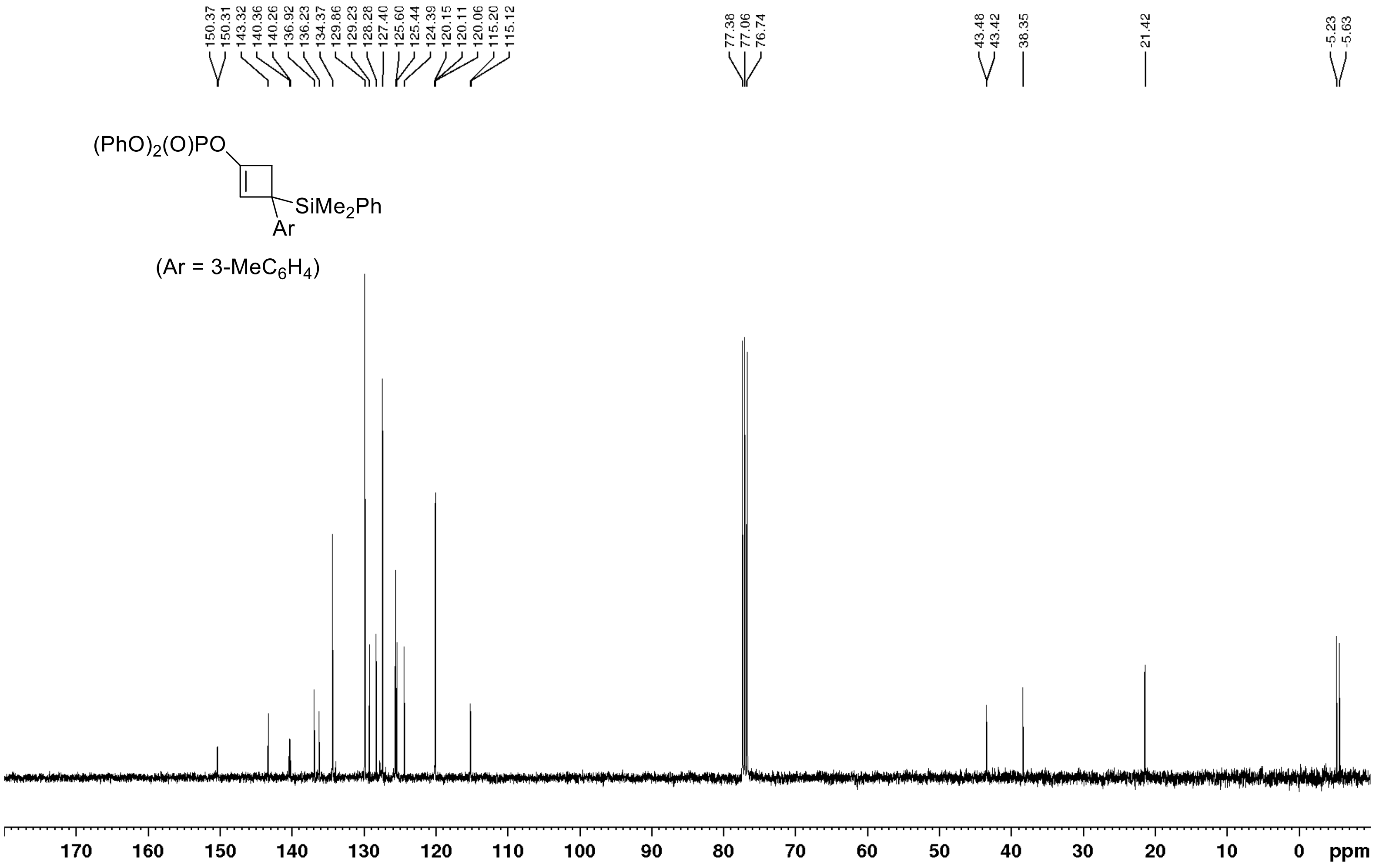

Figure S60. ^{1}H-^{29}Si HMQC NMR (99 MHz, $CDCl_3$, 298 K) of 3-(Dimethyl(phenyl)silyl)-3-(m-tolyl)cyclobut-1-en-1-yl diphenyl phosphate (**3c**)

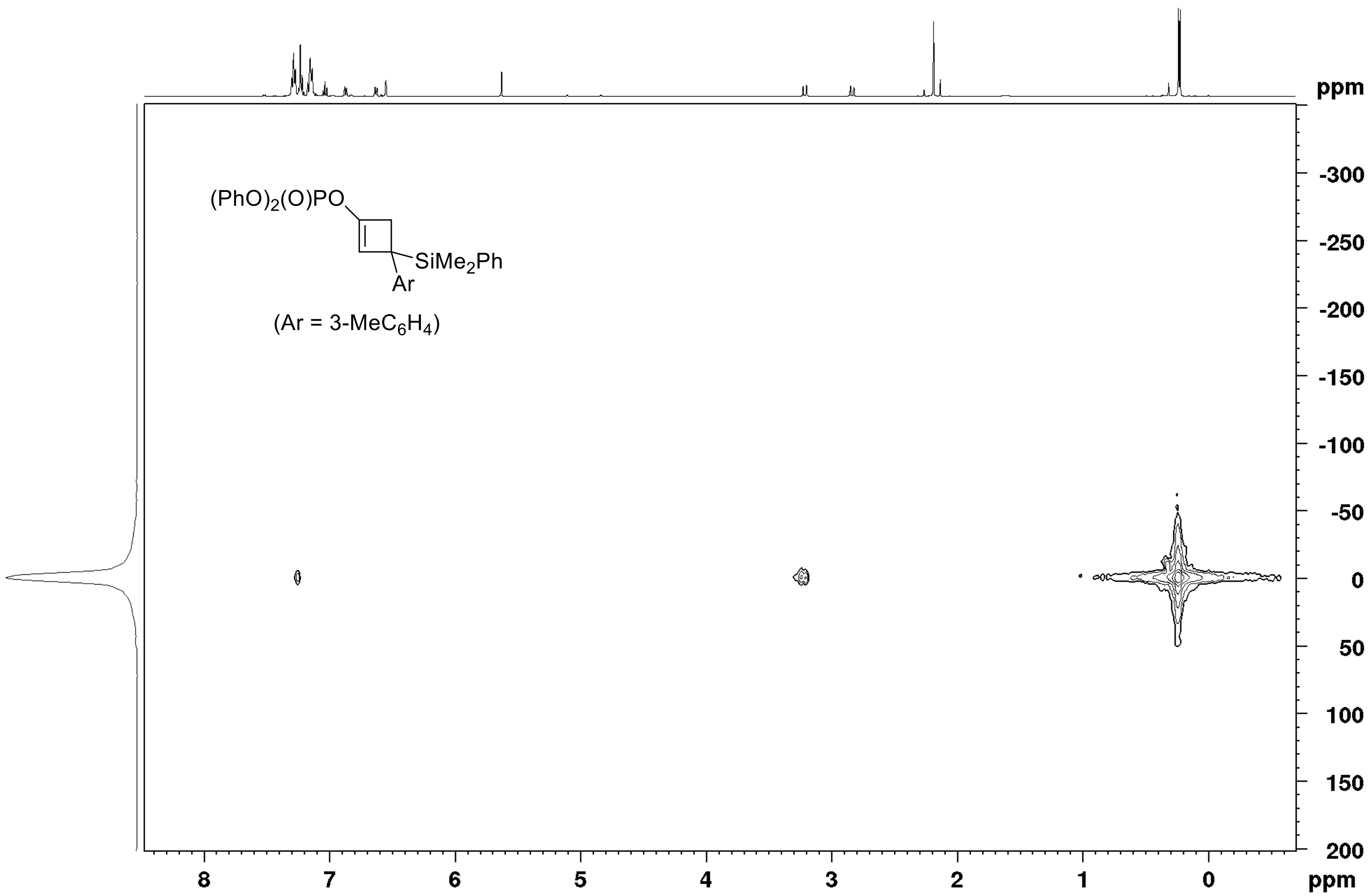

Figure S61. $^{31}P\{^{1}H\}$ NMR spectrum (202 MHz, $CDCl_3$, 298 K) of 3-(Dimethyl(phenyl)silyl)-3-(m-tolyl)cyclobut-1-en-1-yl diphenyl phosphate (**3c**)

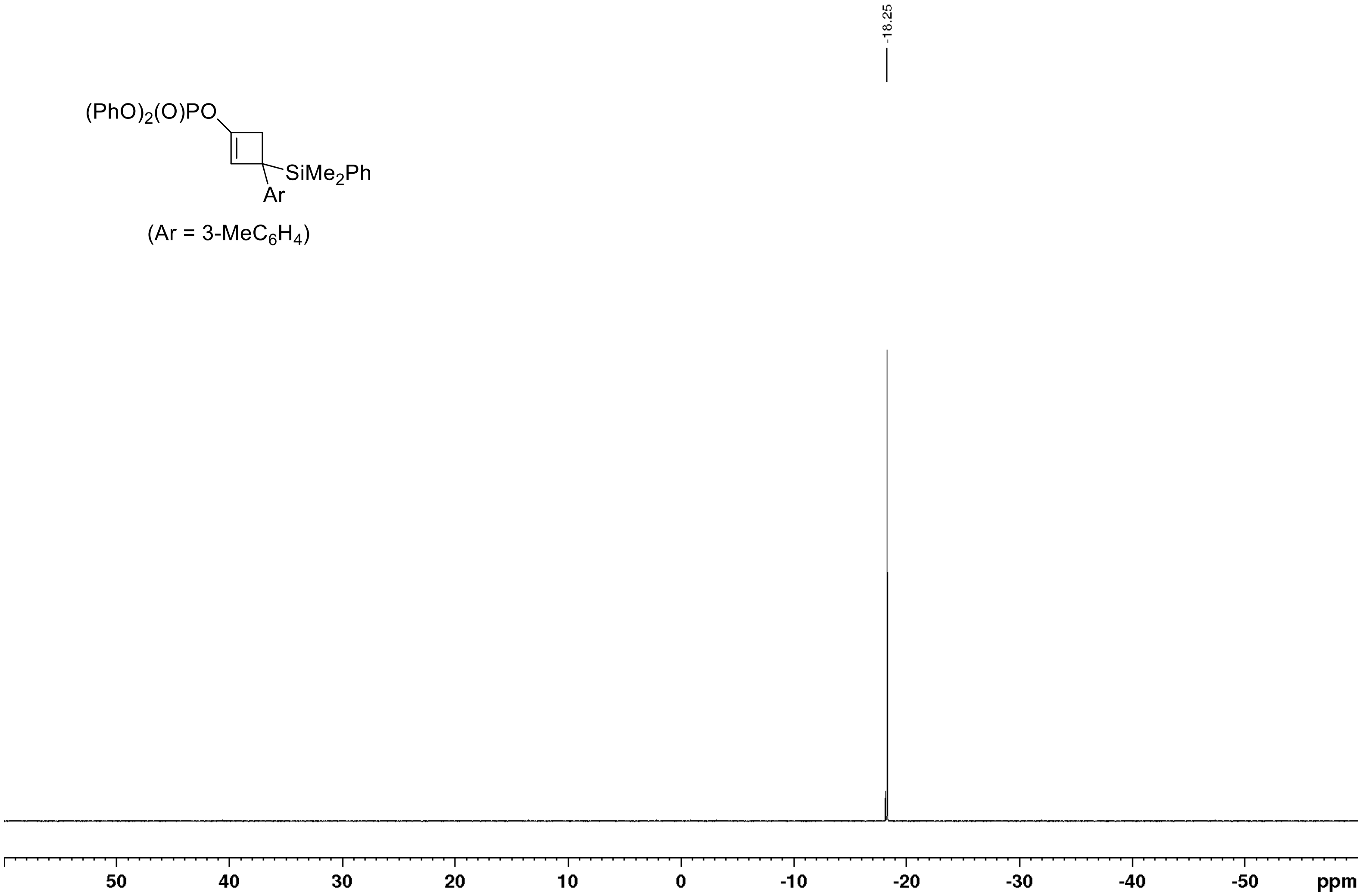

Figure S62. ^{1}H NMR spectrum (500 MHz, $CDCl_3$, 298 K) of 3-(Dimethyl(phenyl)silyl)-3-(4-methoxyphenyl)cyclobut-1-en-1-yl diphenyl phosphate (**3d**)

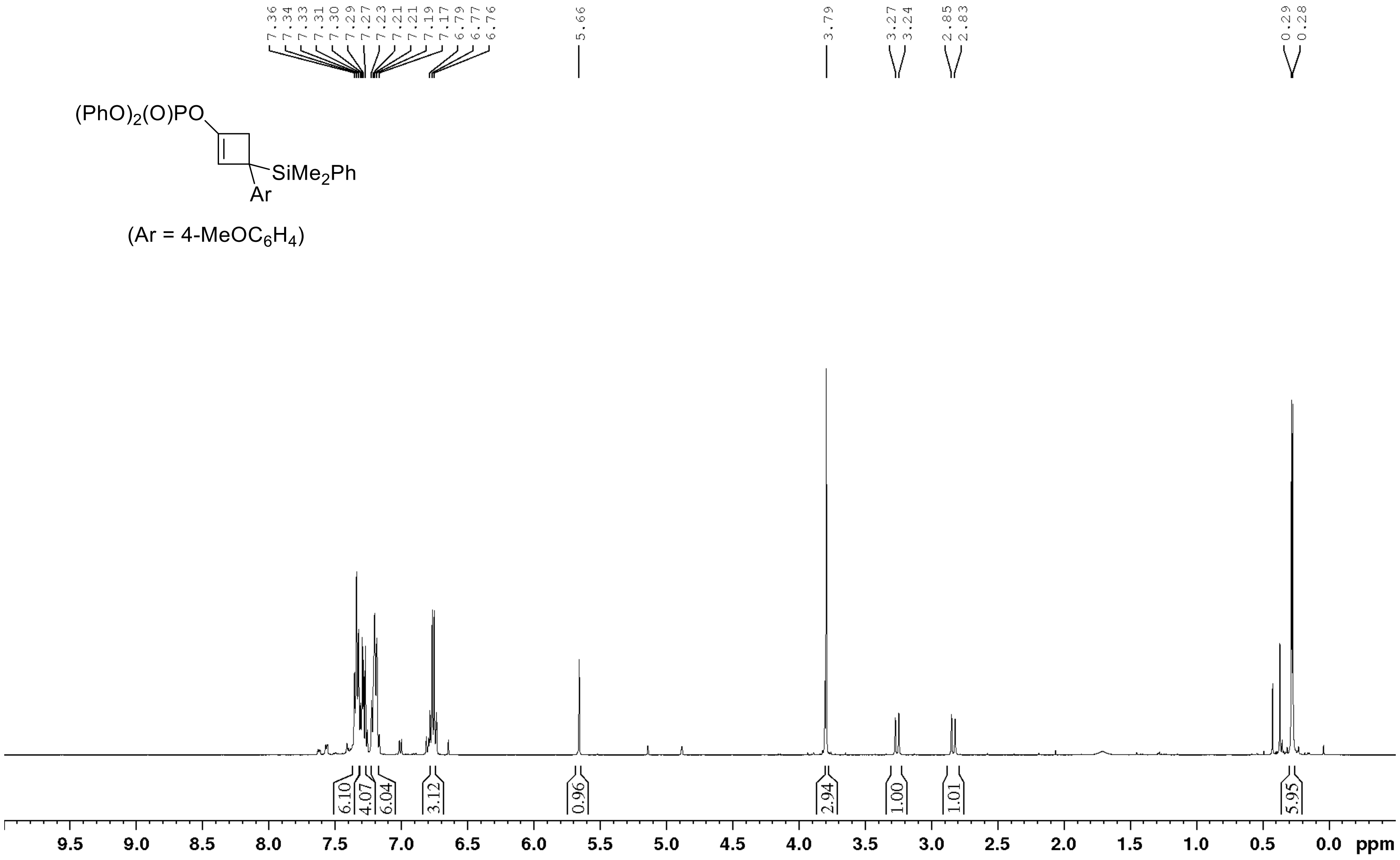

Figure S63. $^{13}C\{^{1}H\}$ NMR spectrum (126 MHz, $CDCl_3$, 298 K) of 3-(Dimethyl(phenyl)silyl)-3-(4-methoxyphenyl)cyclobut-1-en-1-yl diphenyl phosphate (**3d**)

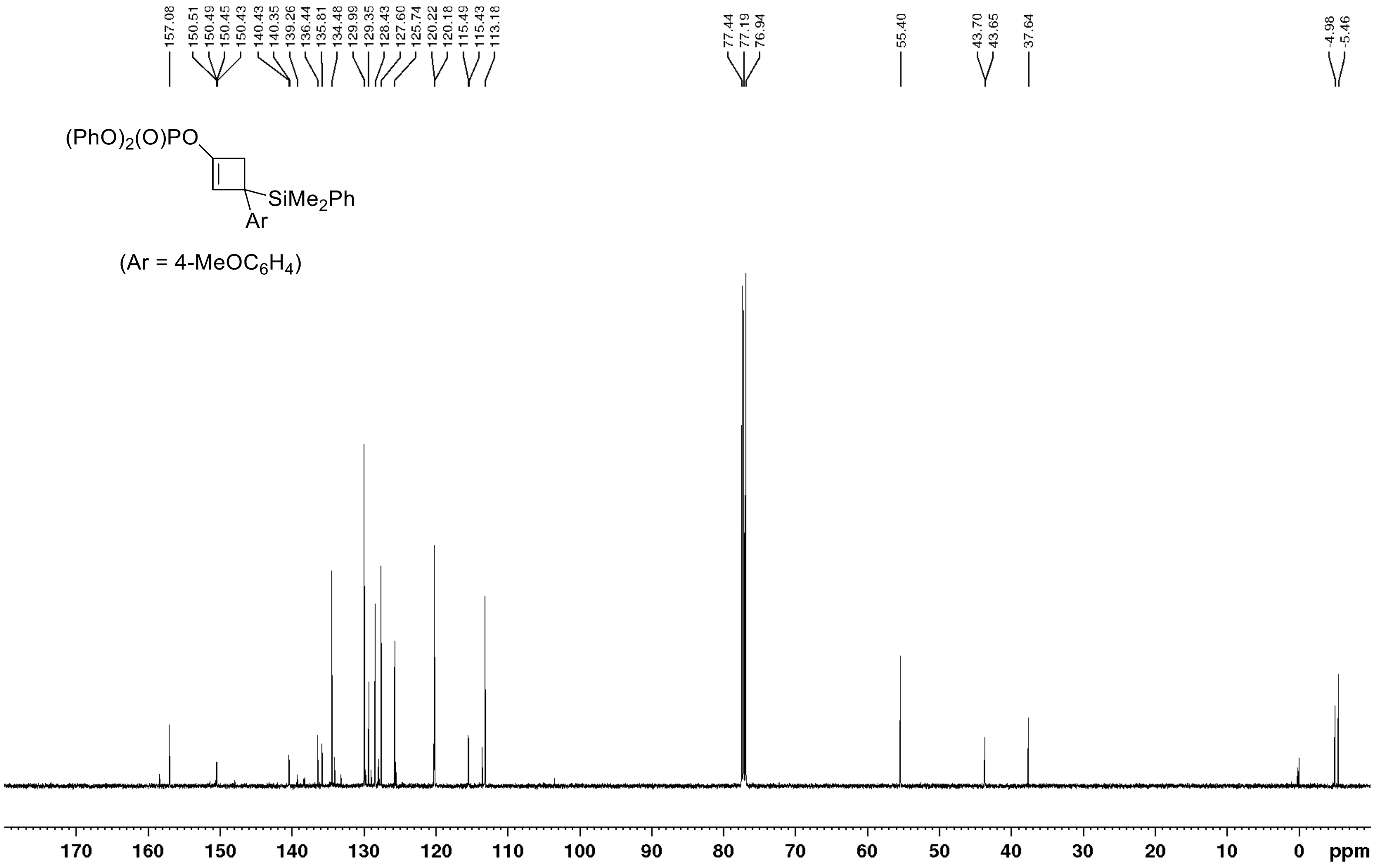

Figure S64. ^1H-^{29}Si HMQC NMR (99 MHz, $CDCl_3$, 298 K) of 3-(Dimethyl(phenyl)silyl)-3-(4-methoxyphenyl)cyclobut-1-en-1-yl diphenyl phosphate (**3d**)

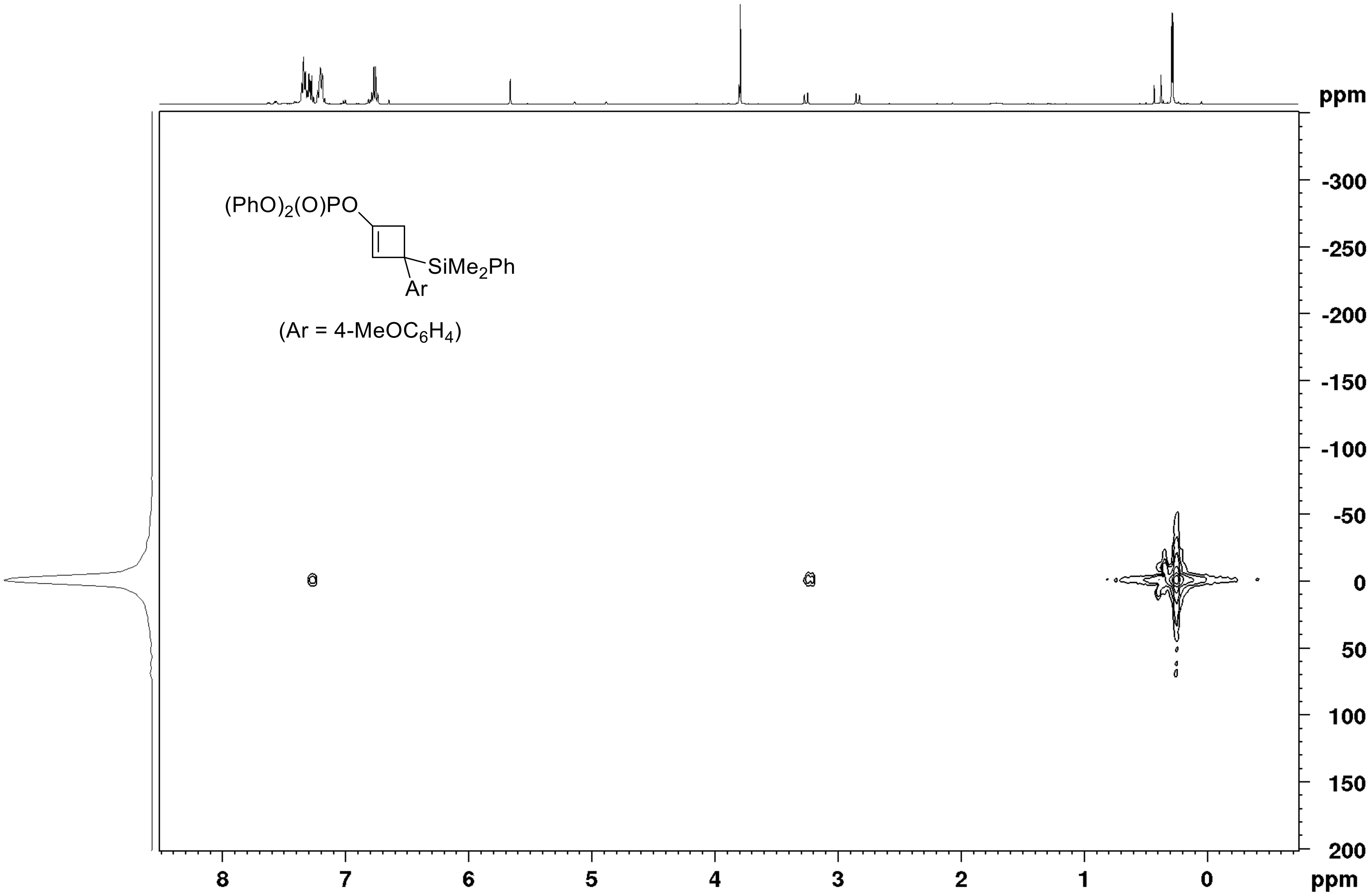

Figure S65. $^{31}P\{^{1}H\}$ NMR (202 MHz, $CDCl_3$, 298 K) of 3-(Dimethyl(phenyl)silyl)-3-(4-methoxyphenyl)cyclobut-1-en-1-yl diphenyl phosphate (**3d**)

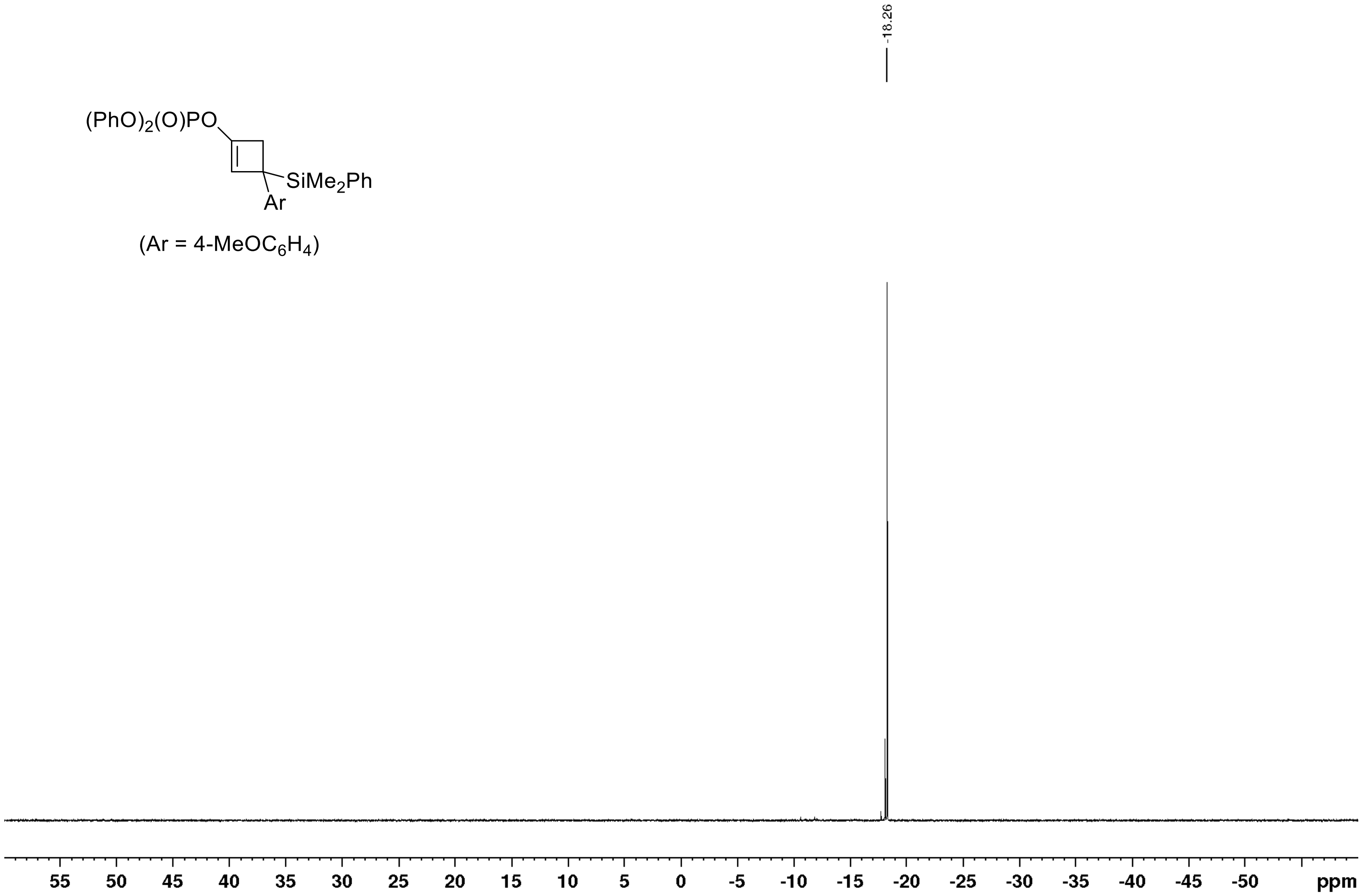

Figure S66. ^{1}H NMR spectrum (500 MHz, $CDCl_3$, 298 K) of 3-(4-Chlorophenyl)-3-(dimethyl(phenyl)silyl)cyclobut-1-en-1-yl diphenyl phosphate (**3e**) (# = Cyclohexane)

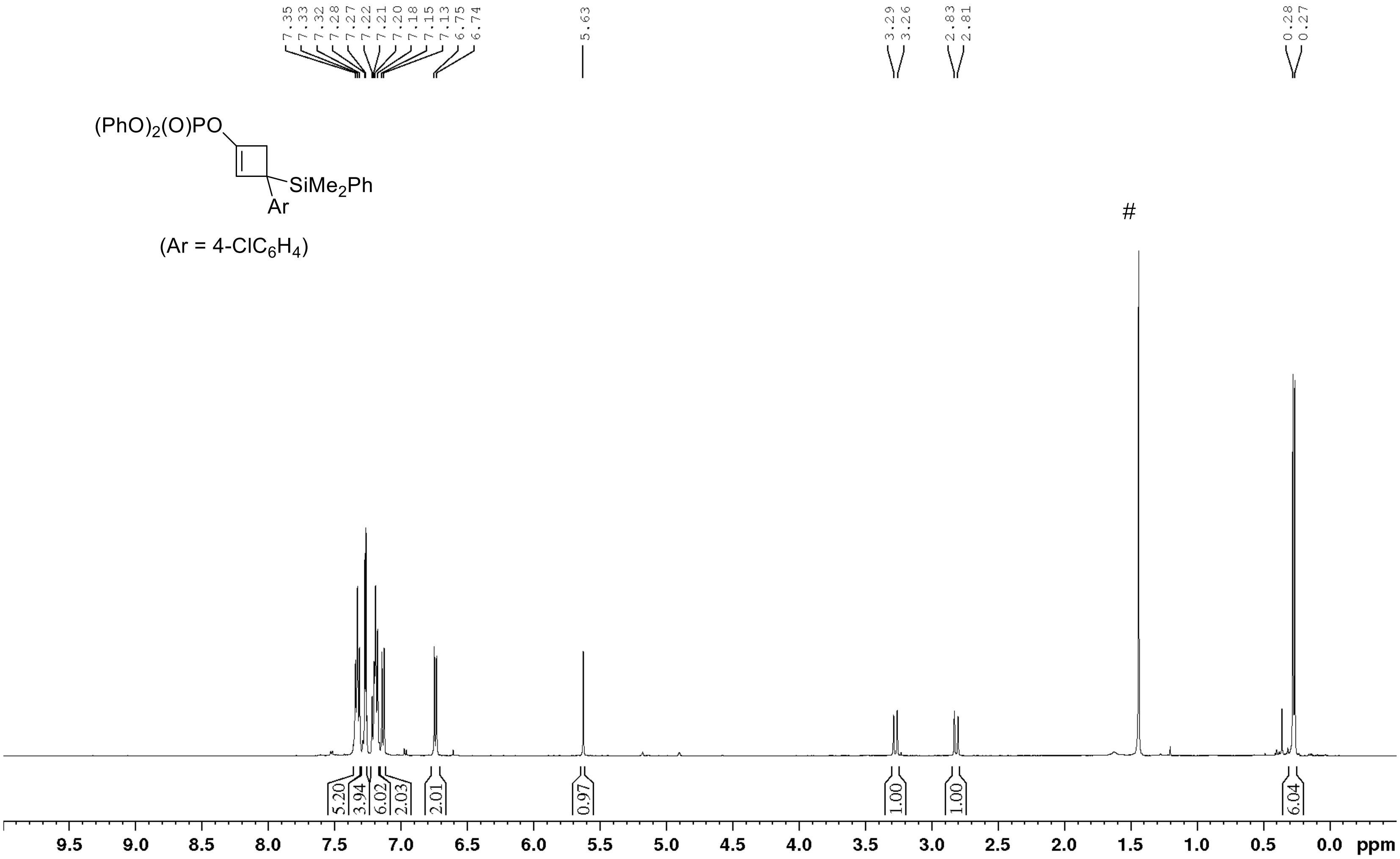

Figure S67. $^{13}C\{^1H\}$ NMR spectrum (126 MHz, $CDCl_3$, 298 K) of 3-(4-Chlorophenyl)-3-(dimethyl(phenyl)silyl)cyclobut-1-en-1-yl diphenyl phosphate (**3e**) (# = Cyclohexane)

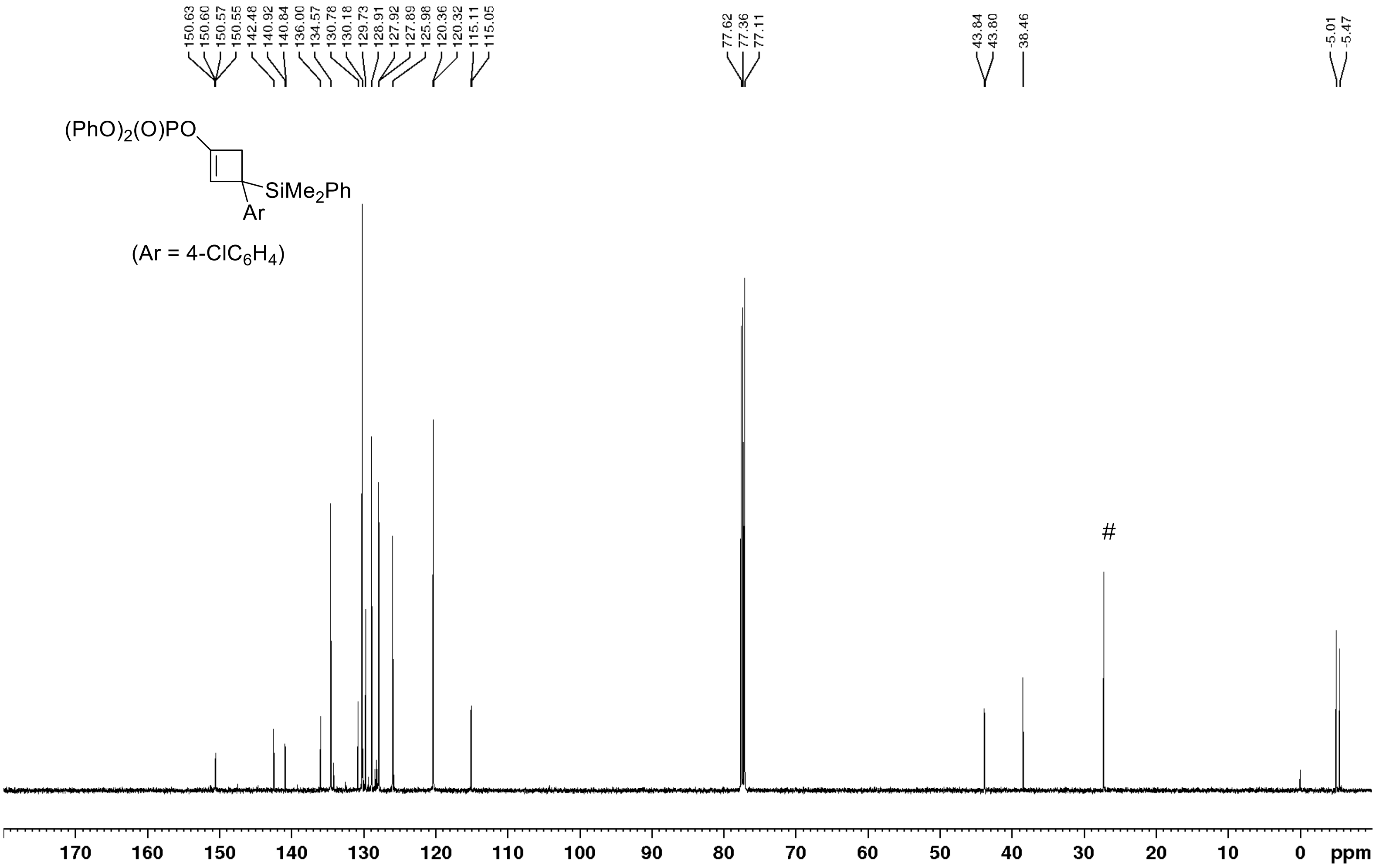

Figure S68. ^{29}Si {^{1}H} DEPT NMR spectrum (99 MHz, $CDCl_3$) of 3-(4-Chlorophenyl)-3-(dimethyl(phenyl)silyl)cyclobut-1-en-1-yl diphenyl phosphate (**3e**)

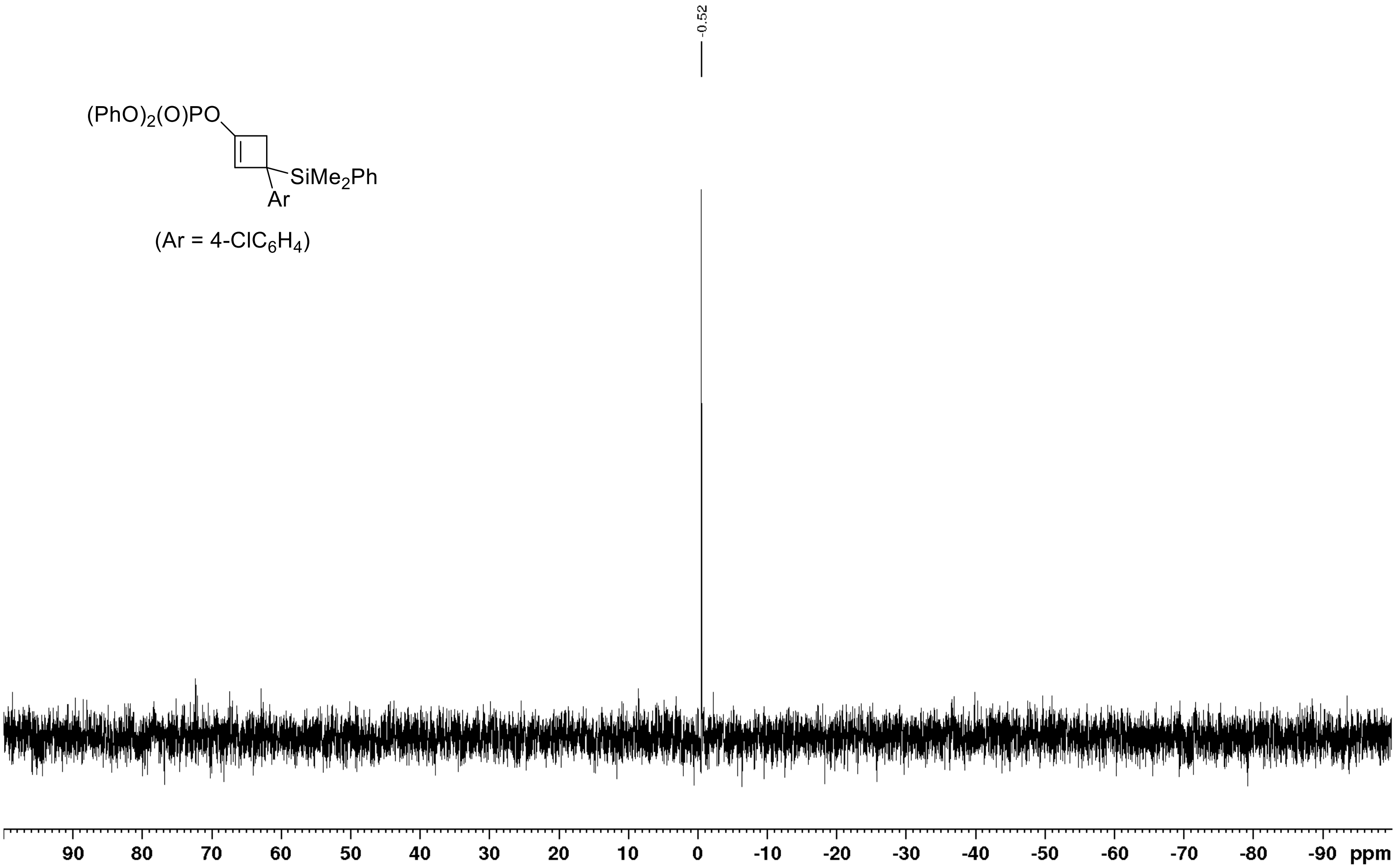

Figure S69. $^{31}P\{^1H\}$ NMR spectrum (202 MHz, $CDCl_3$, 298 K) of 3-(4-Chlorophenyl)-3-(dimethyl(phenyl)silyl)cyclobut-1-en-1-yl diphenyl phosphate (**3e**)

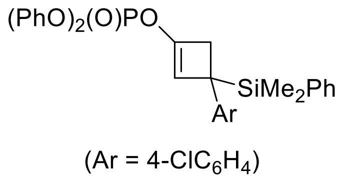

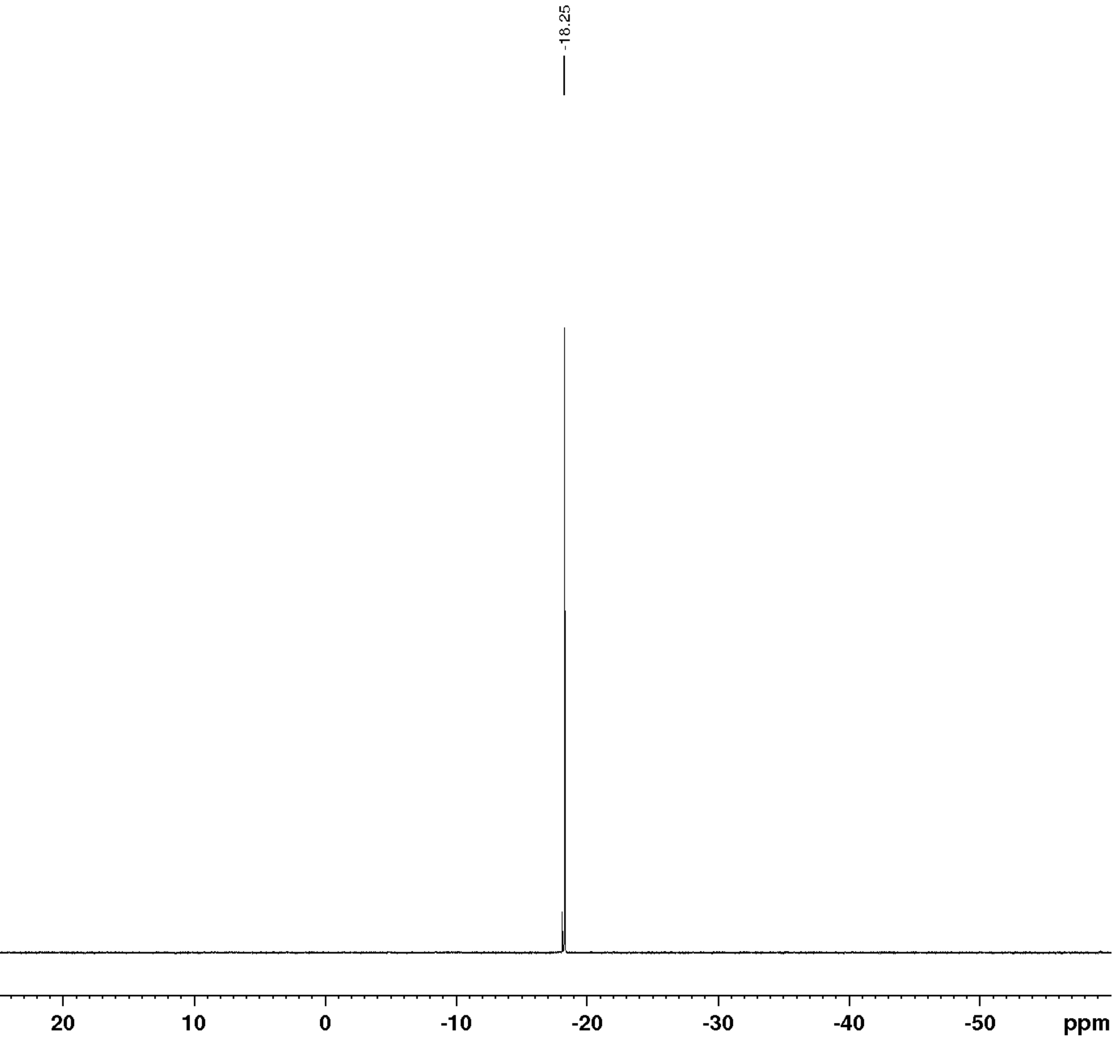

Figure S70. ^{1}H NMR spectrum (500 MHz, $CDCl_3$, 298 K) of 3-(Dimethyl(phenyl)silyl)-3-(4-fluorophenyl)cyclobut-1-en-1-yl diphenyl phosphate (**3f**)

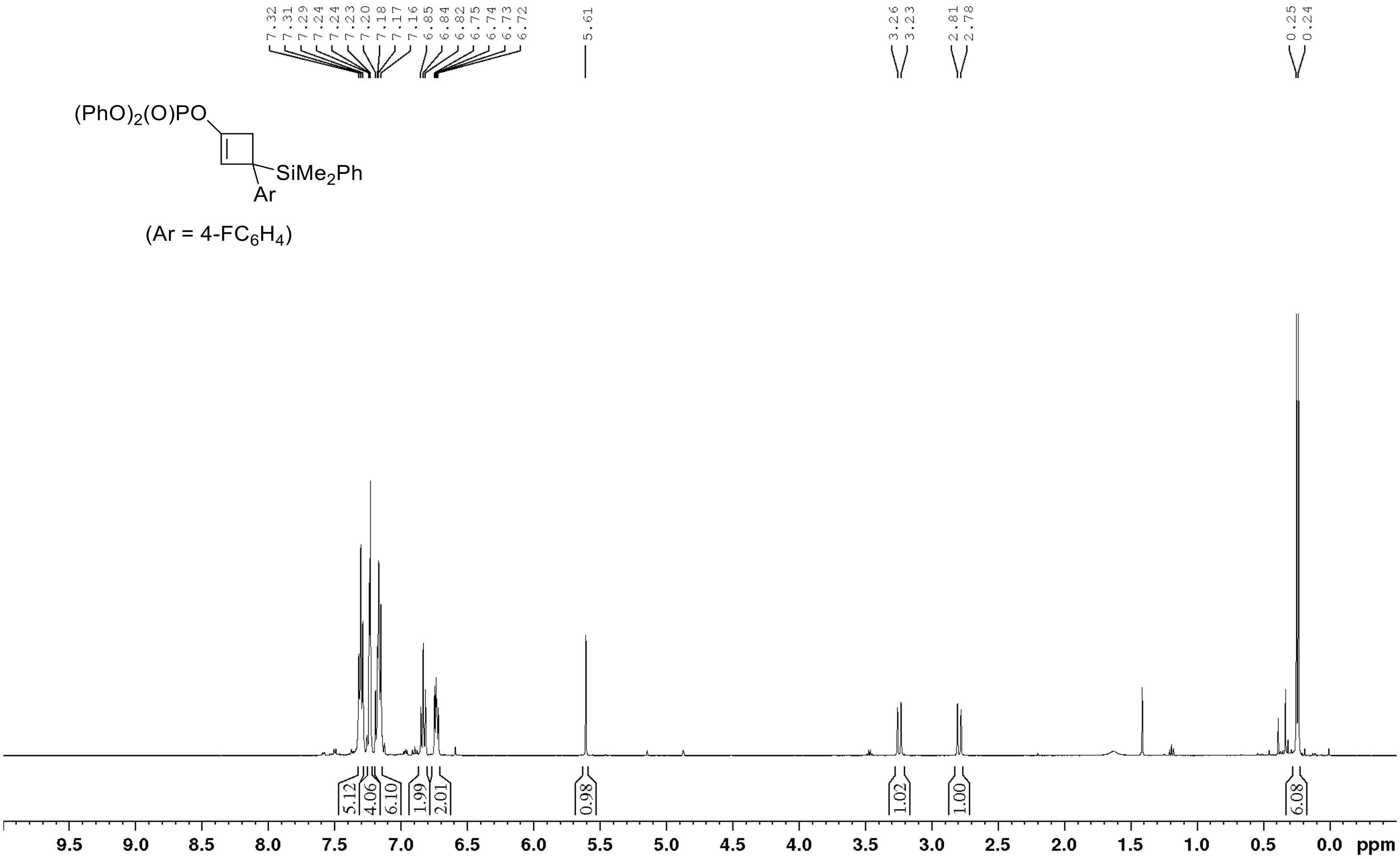

Figure S71. $^{13}C\{^{1}H\}$ NMR spectrum (101 MHz, $CDCl_3$, 298 K) of 3-(Dimethyl(phenyl)silyl)-3-(4-fluorophenyl)cyclobut-1-en-1-yl diphenyl phosphate (**3f**)

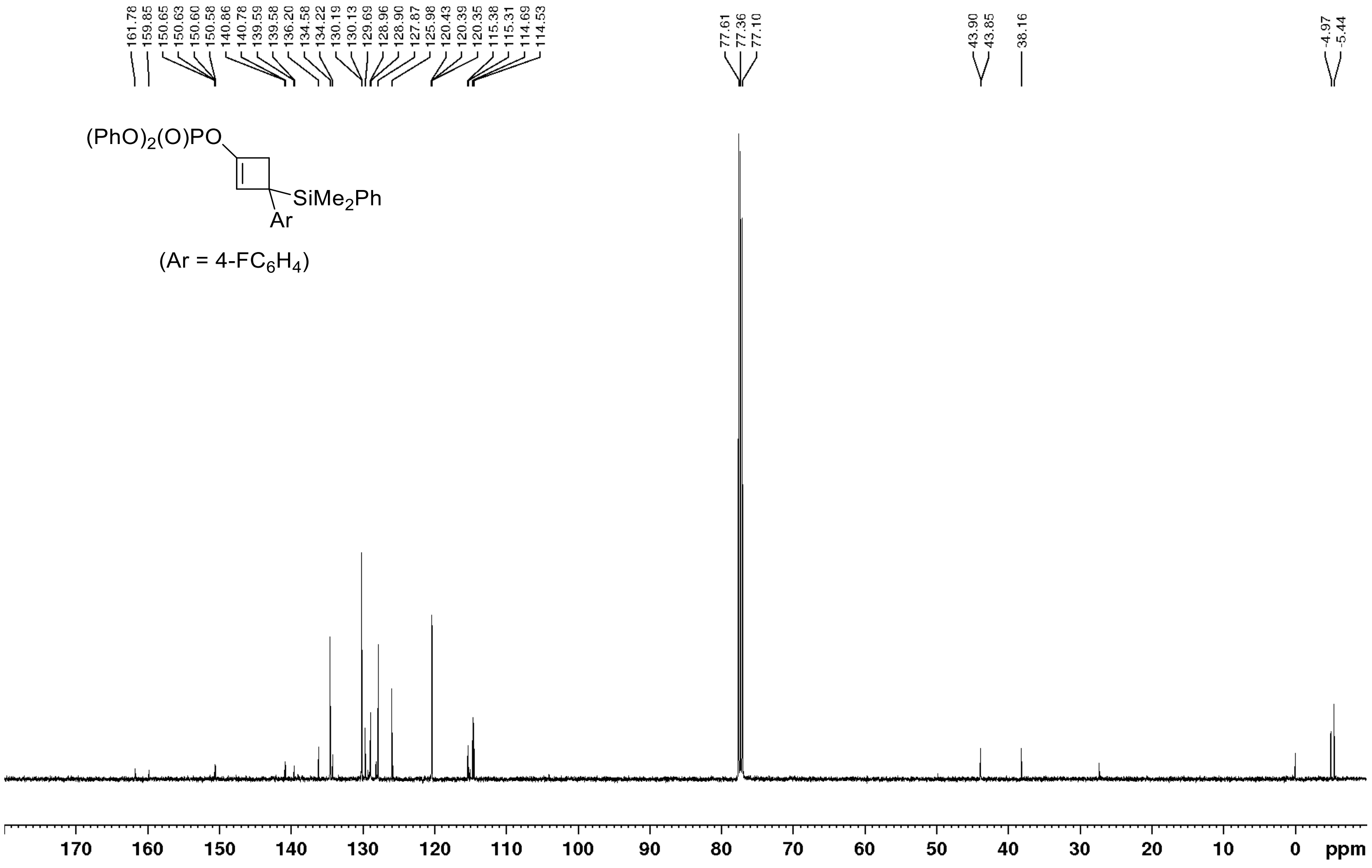

Figure S72. ^{29}Si {^{1}H} DEPT NMR spectrum (99 MHz, $CDCl_3$) of 3-(Dimethyl(phenyl)silyl)-3-(4-fluorophenyl)cyclobut-1-en-1-yl diphenyl phosphate (**3f**)

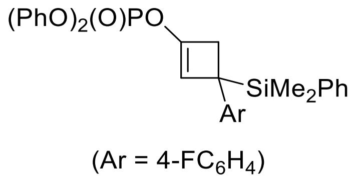

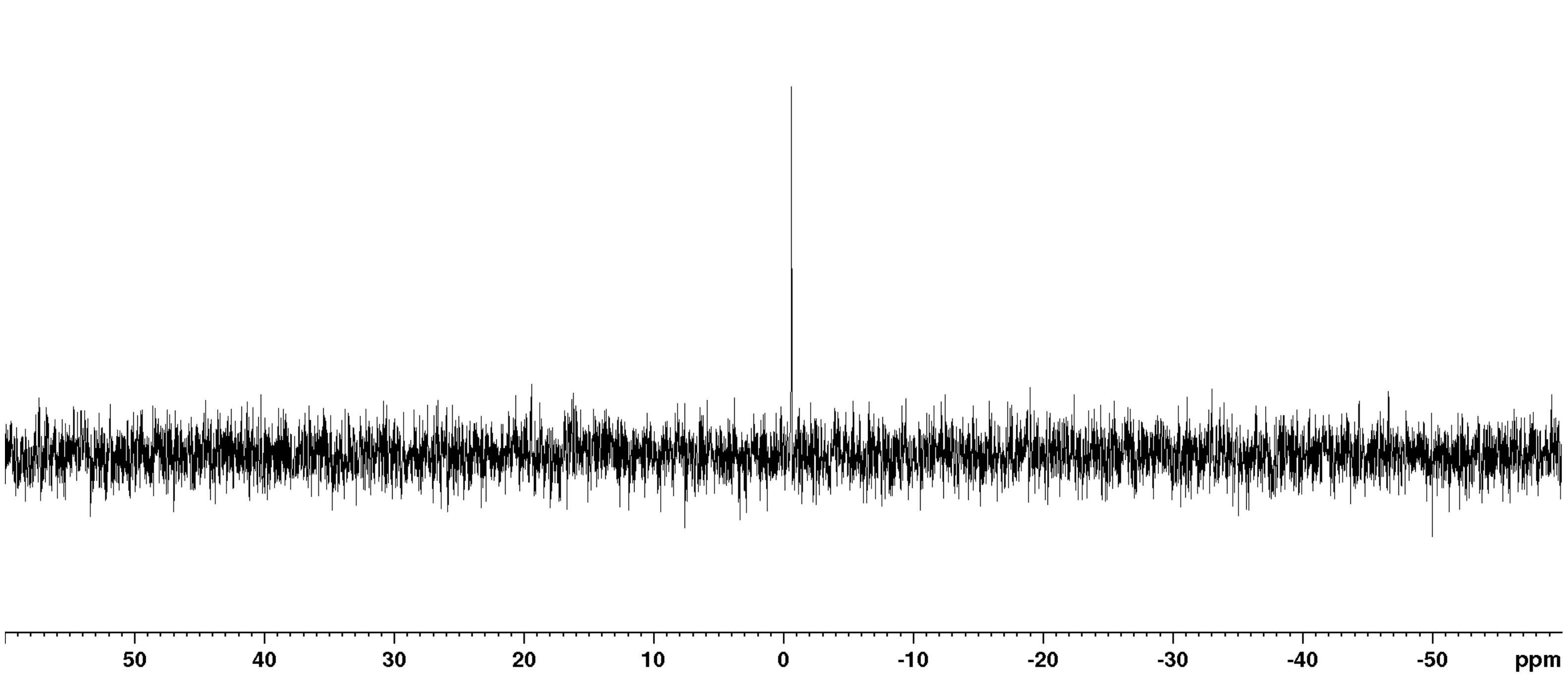

Figure S73. $^{31}P\{^1H\}$ NMR spectrum (202 MHz, $CDCl_3$, 298 K) of 3-(Dimethyl(phenyl)silyl)-3-(4-fluorophenyl)cyclobut-1-en-1-yl diphenyl phosphate (**3f**)

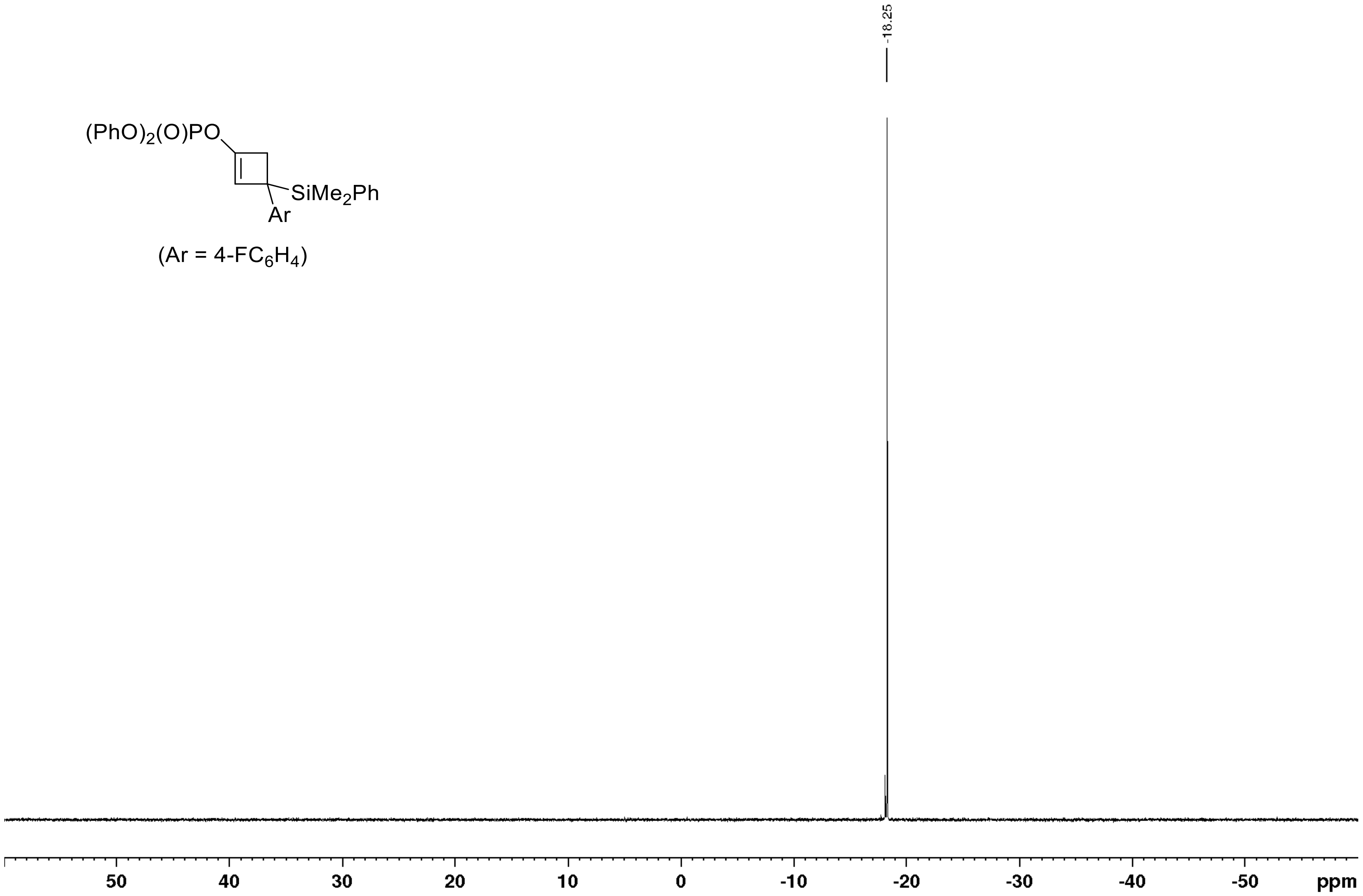

Figure S74. ^{19}F NMR (471 MHz, $CDCl_3$, 298 K) of 3-(Dimethyl(phenyl)silyl)-3-(4-fluorophenyl)cyclobut-1-en-1-yl diphenyl phosphate (**3f**)

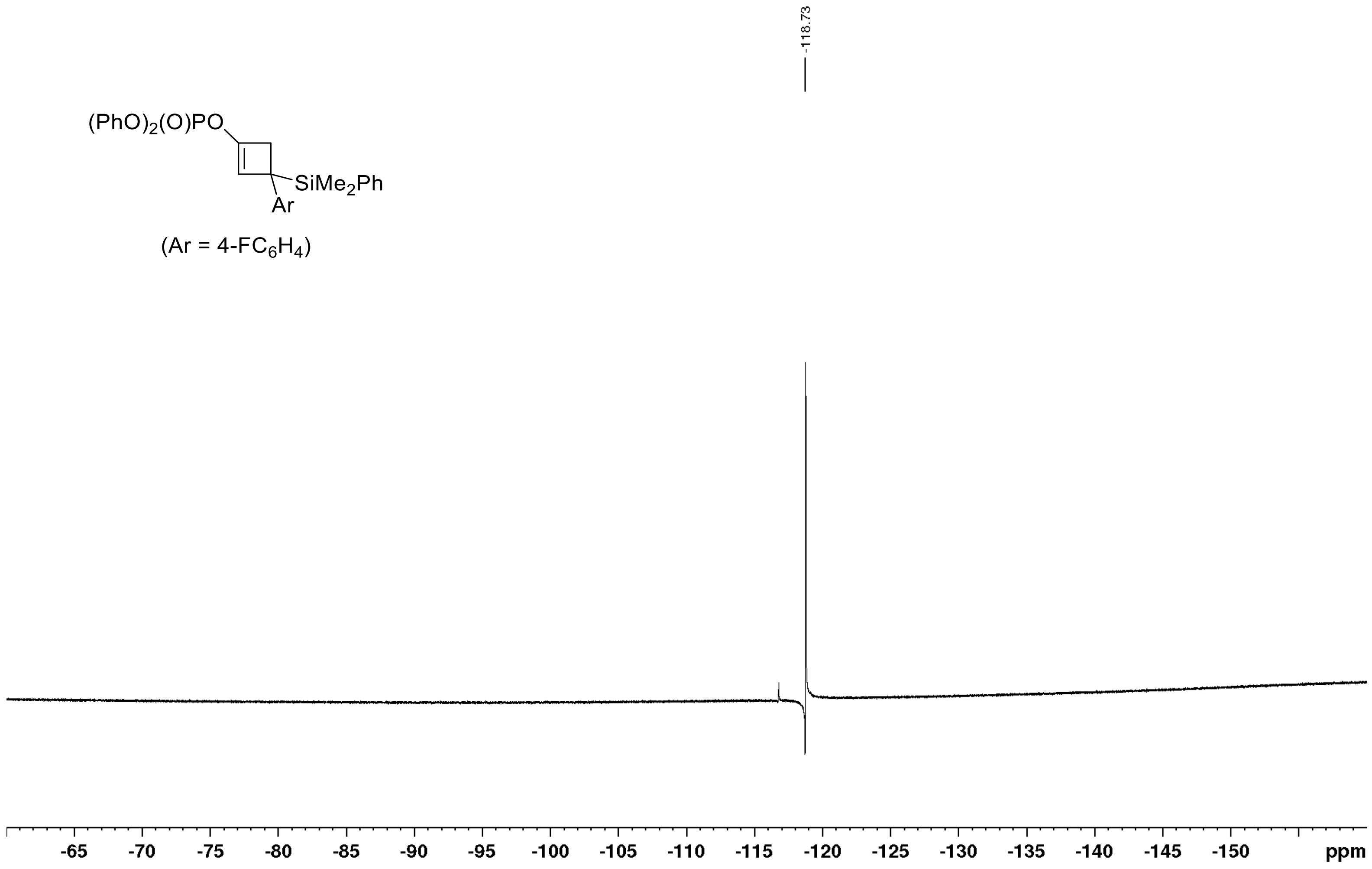

Figure S75. ^{1}H NMR (500 MHz, $CDCl_3$, 298 K) of 3-Butyl-3-(Dimethyl(phenyl)silyl)cyclobut-1-en-1-yl diphenyl phosphate (**3g**)

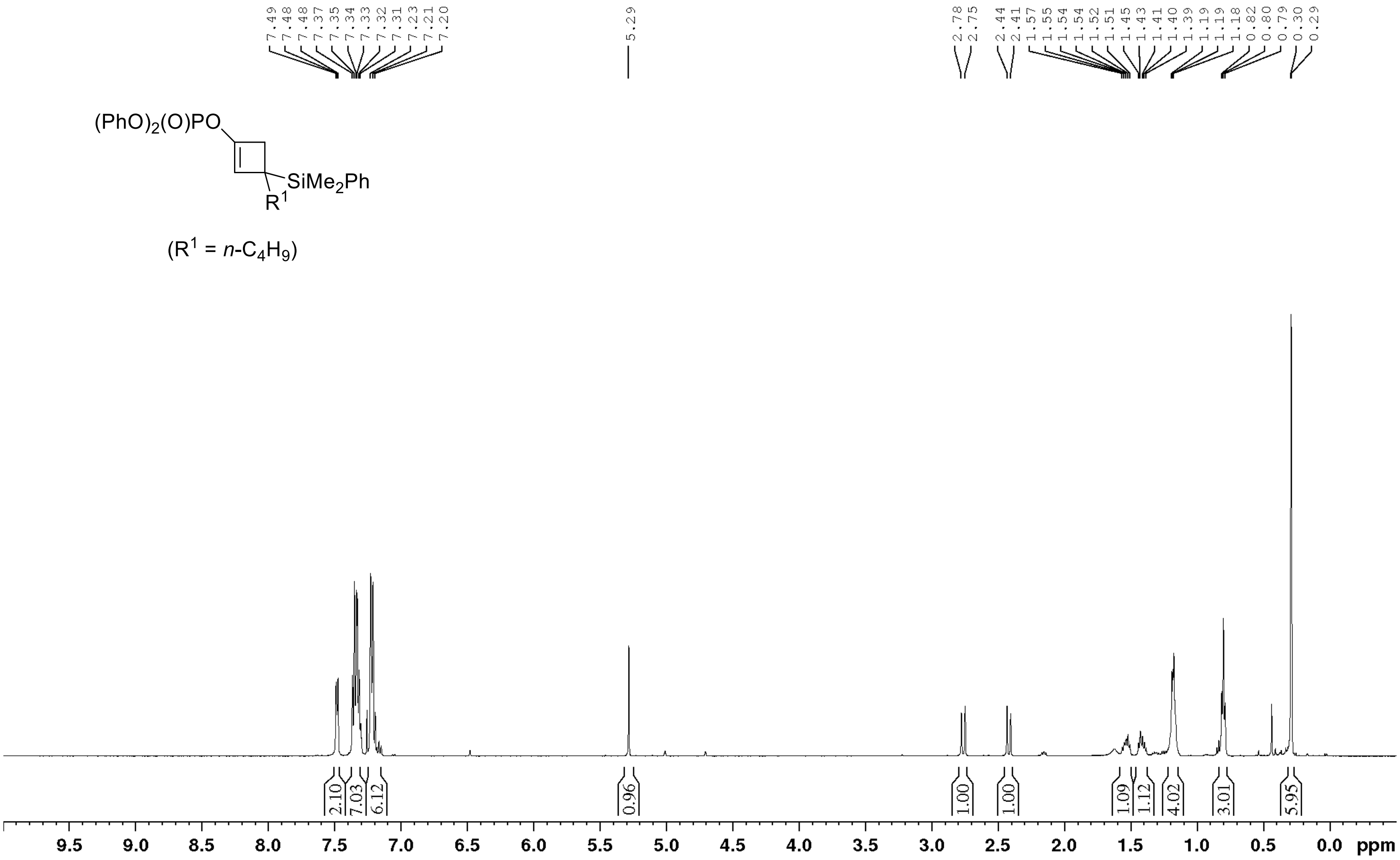

Figure S76. $^{13}C\{^{1}H\}$ NMR (126 MHz, $CDCl_3$, 298 K) of 3-Butyl-3-(dimethyl(phenyl)silyl)cyclobut-1-en-1-yl diphenyl phosphate (**3g**)

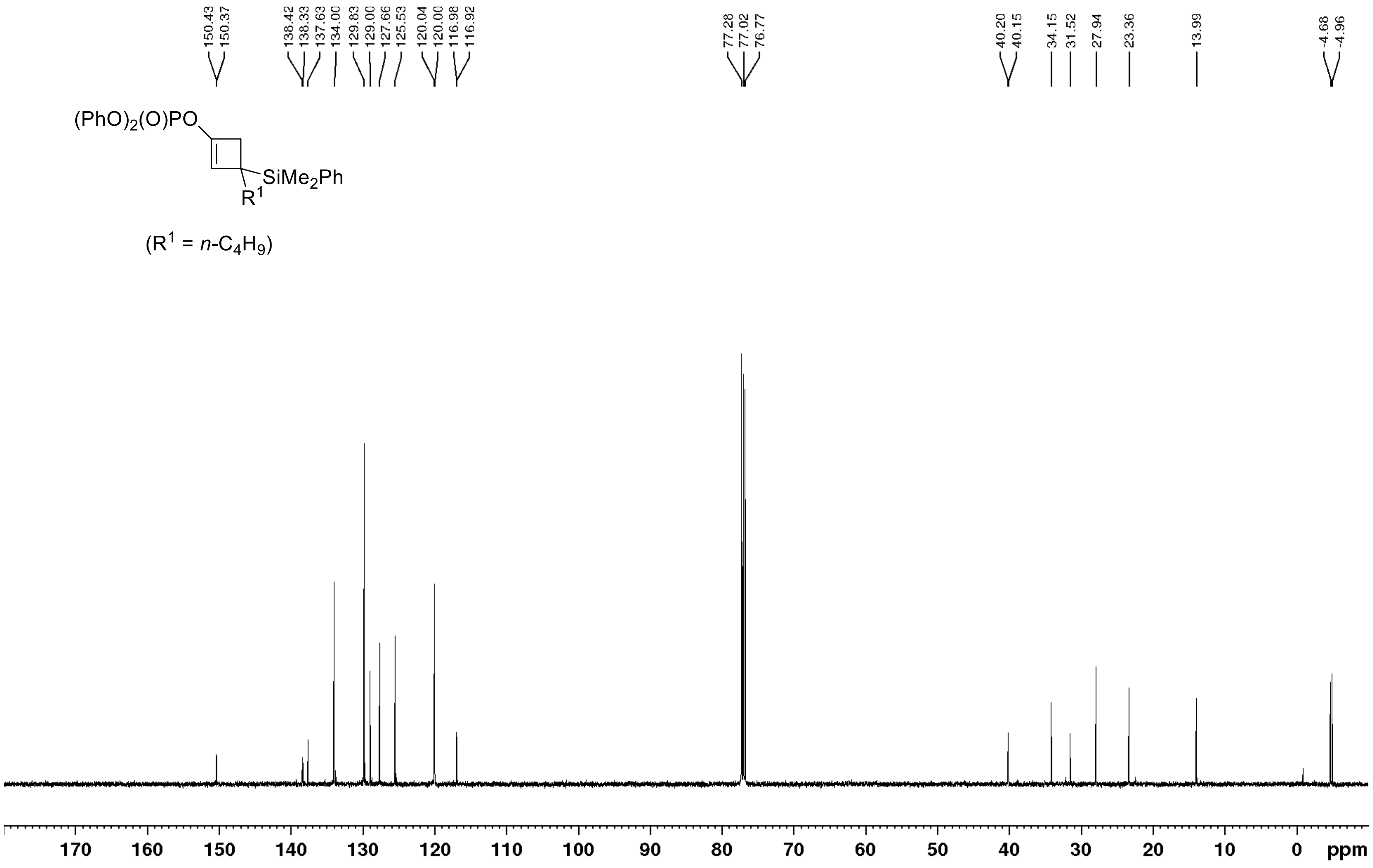

Figure S77. $^{29}Si\{^1H\}$ DEPT NMR (99 MHz, $CDCl_3$, 298 K) of 3-Butyl-3-(dimethyl(phenyl)silyl)cyclobut-1-en-1-yl diphenyl phosphate (**3g**)

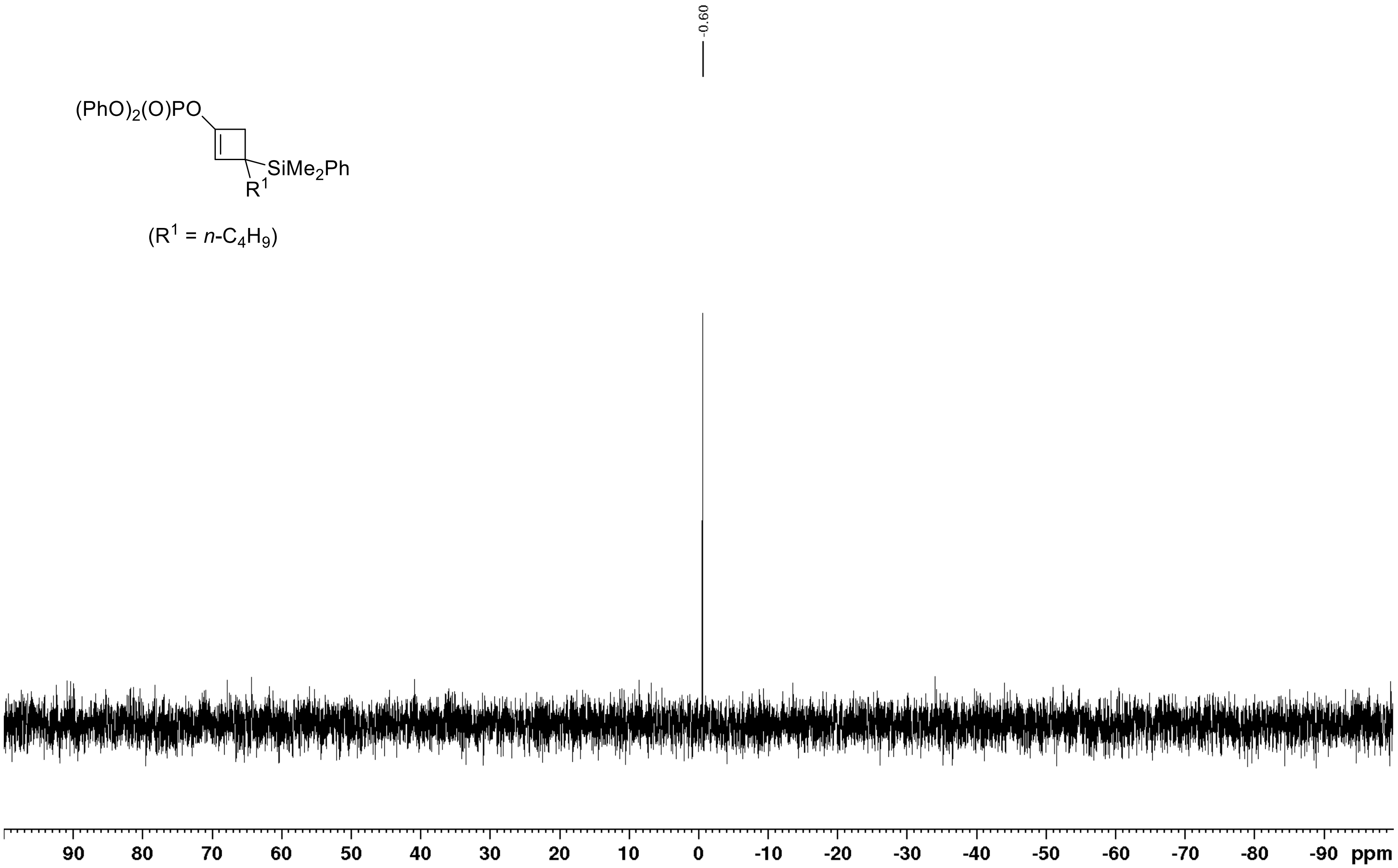

Figure S78. $^{31}P\{^{1}H\}$ NMR (99 MHz, $CDCl_3$, 298 K) of 3-Butyl-3-(dimethyl(phenyl)silyl)cyclobut-1-en-1-yl diphenyl phosphate (**3g**)

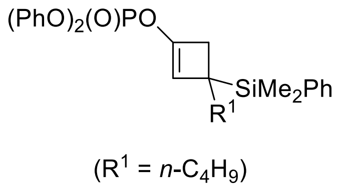

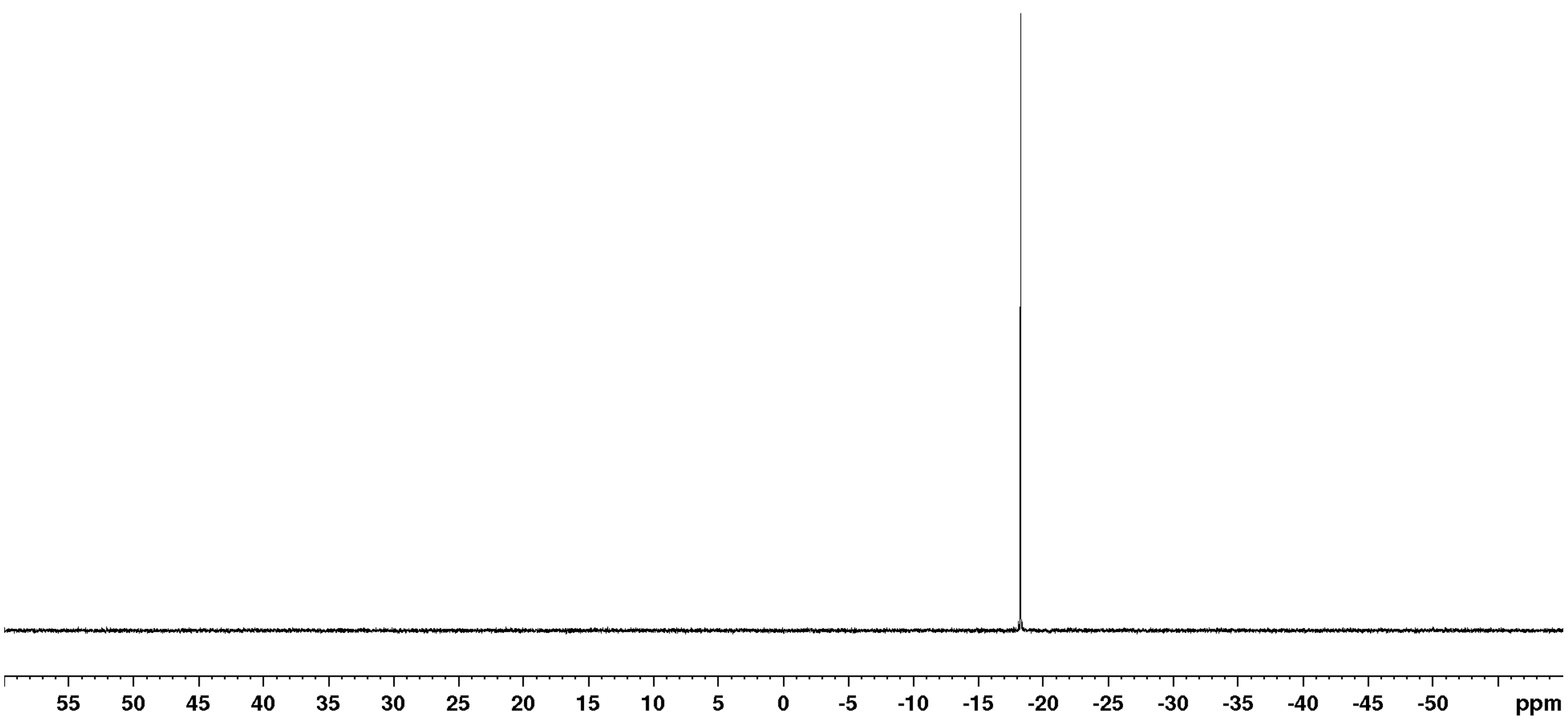

Figure S79. ^{1}H NMR (500 MHz, $CDCl_3$, 298 K) of 3-(Dimethyl(phenyl)silyl)-3-hexylcyclobut-1-en-1-yl diphenyl phosphate (**3h**)

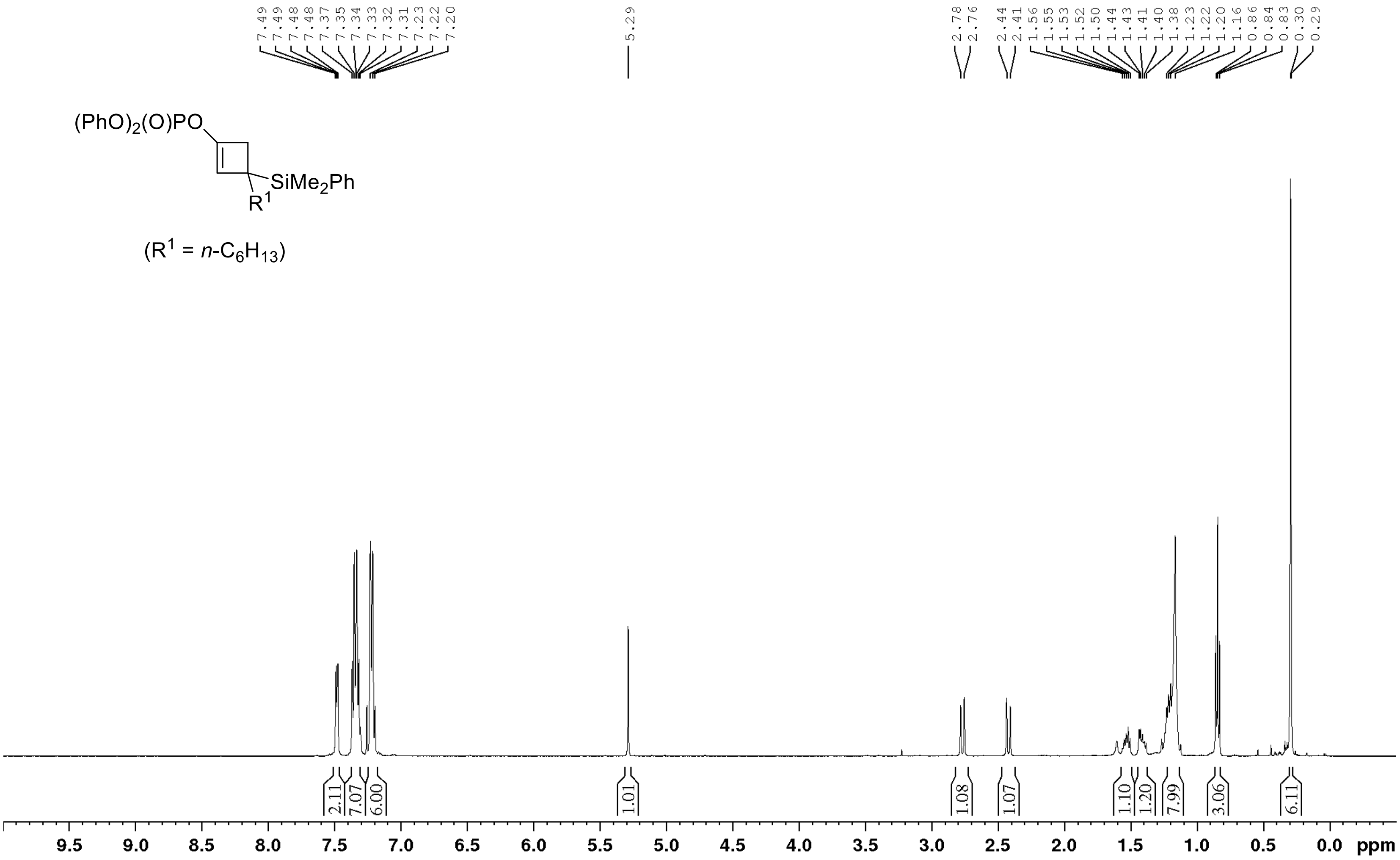

Figure S80. $^{13}C\{^{1}H\}$ NMR (126 MHz, $CDCl_3$, 298 K) of 3-(Dimethyl(phenyl)silyl)-3-hexylcyclobut-1-en-1-yl diphenyl phosphate (**3h**)

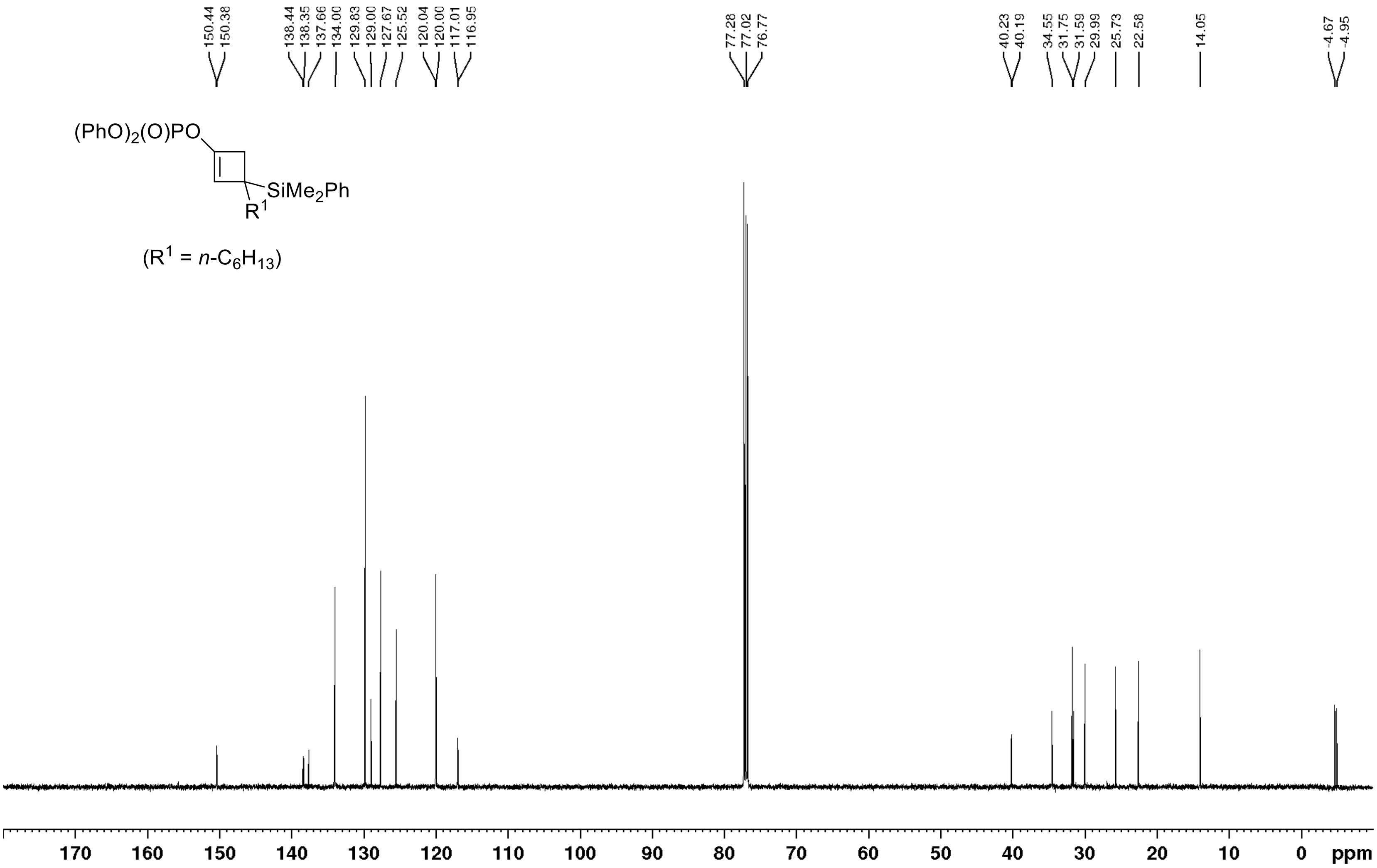

Figure S81. $^{29}Si\{^{1}H\}$ DEPT NMR (99 MHz, $CDCl_3$, 298 K) of 3-(Dimethyl(phenyl)silyl)-3-hexylcyclobut-1-en-1-yl diphenyl phosphate (**3h**)

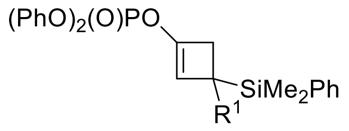

(R[1] = *n*-C_6H_{13})

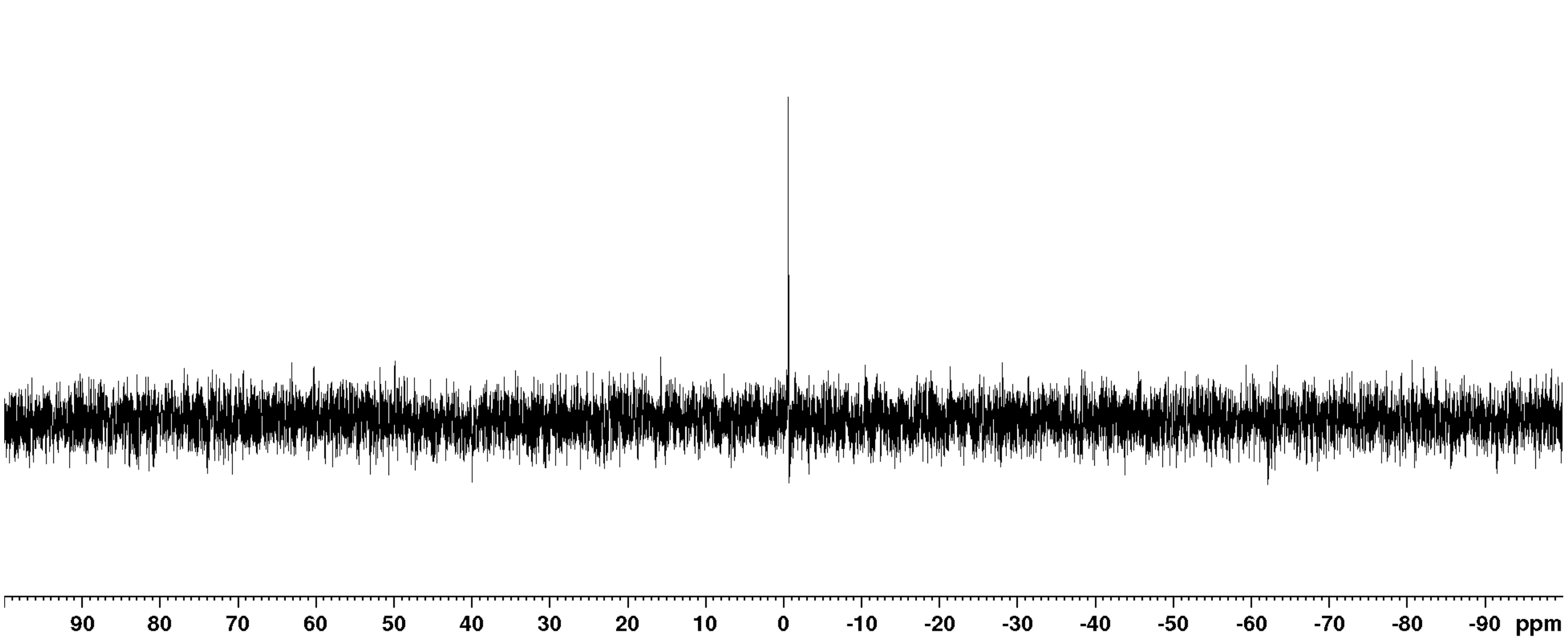

Figure S82. $^{31}P\{^{1}H\}$ NMR (202 MHz, $CDCl_3$, 298 K) of 3-(Dimethyl(phenyl)silyl)-3-hexylcyclobut-1-en-1-yl diphenyl phosphate (**3h**)

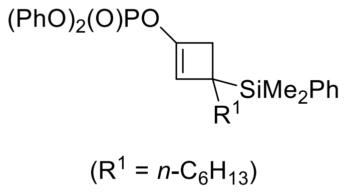

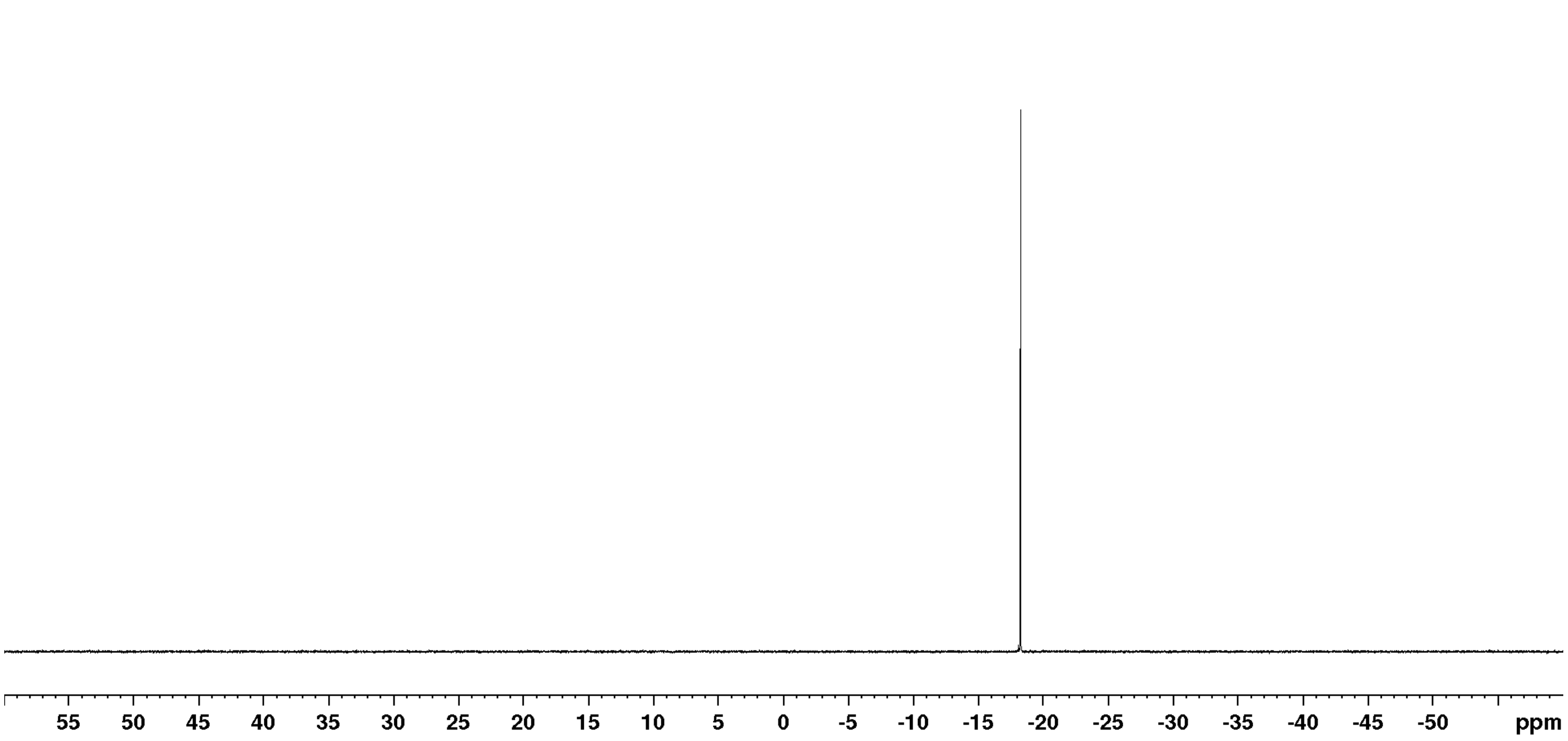

Figure S83. ^1H NMR (500 MHz, $CDCl_3$, 298 K) of 3-(Dimethyl(phenyl)silyl)-3-phenethylcyclobut-1-en-1-yl diphenyl phosphate (**3i**)

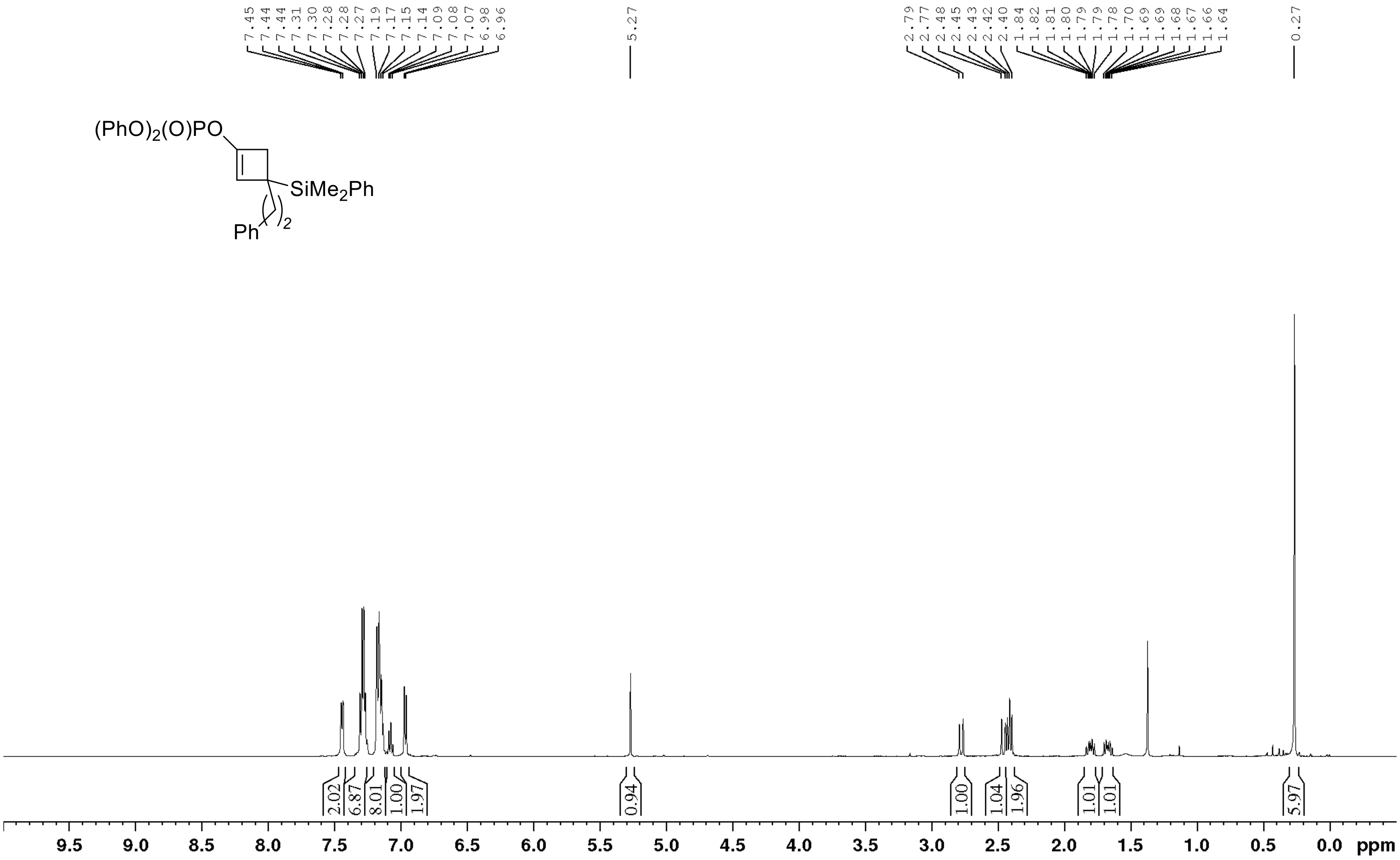

Figure S84. $^{13}C\{^{1}H\}$ NMR (126 MHz, $CDCl_3$, 298 K) of 3-(Dimethyl(phenyl)silyl)-3-phenethylcyclobut-1-en-1-yl diphenyl phosphate (**3i**)

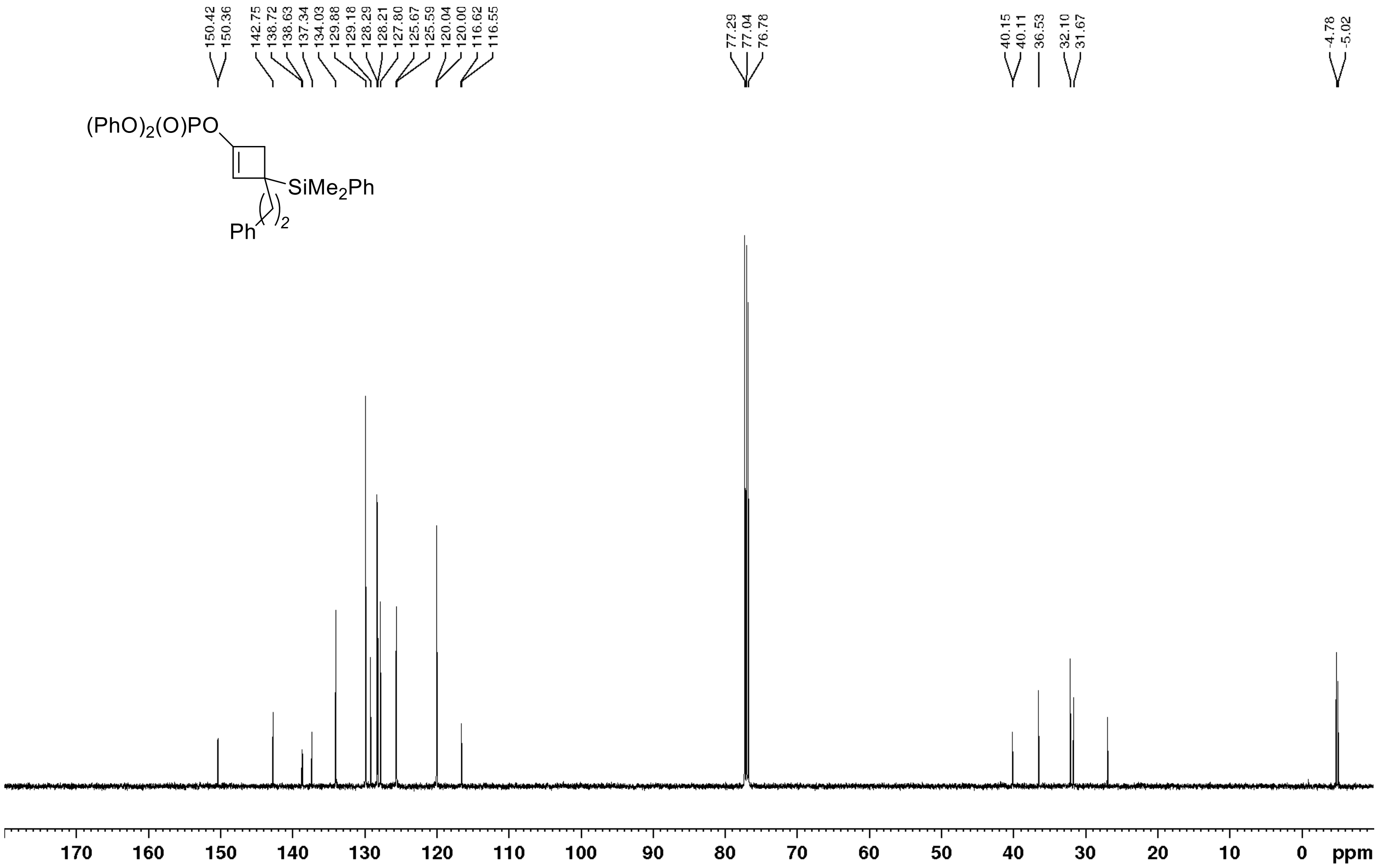

Figure S85. $^{29}Si\{^1H\}$ DEPT NMR (99 MHz, $CDCl_3$, 298 K) of 3-(Dimethyl(phenyl)silyl)-3-phenethylcyclobut-1-en-1-yl diphenyl phosphate (**3i**)

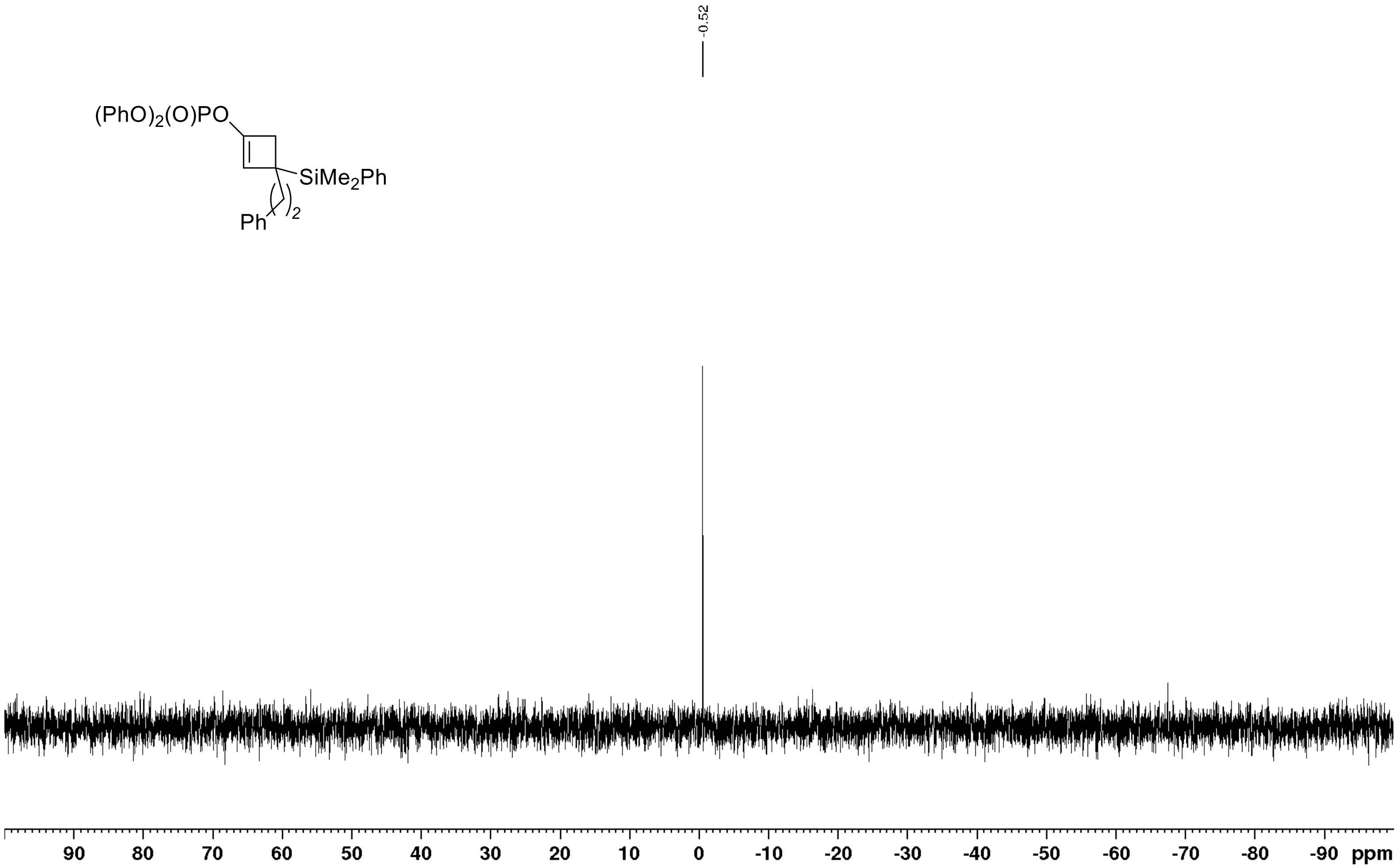

Figure S86. $^{31}P\{^1H\}$ NMR (202 MHz, $CDCl_3$, 298 K) of 3-(Dimethyl(phenyl)silyl)-3-phenethylcyclobut-1-en-1-yl diphenyl phosphate (**3i**)

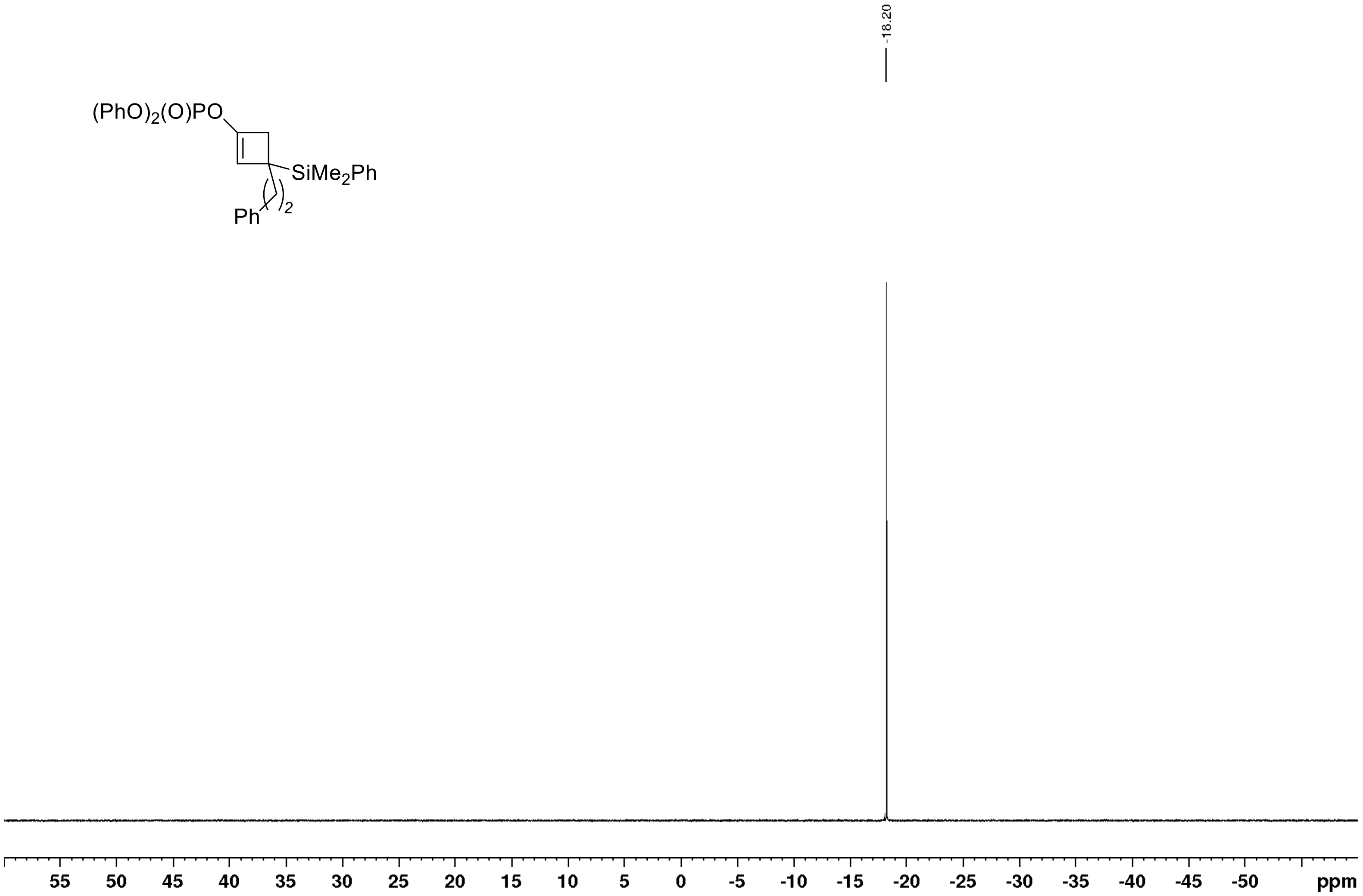

Figure S87. ^{1}H NMR (500 MHz, $CDCl_3$, 298 K) of 3-(Dimethyl(phenyl)silyl)-3-(3-phenylpropyl)cyclobut-1-en-1-yl diphenyl phosphate (**3j**)

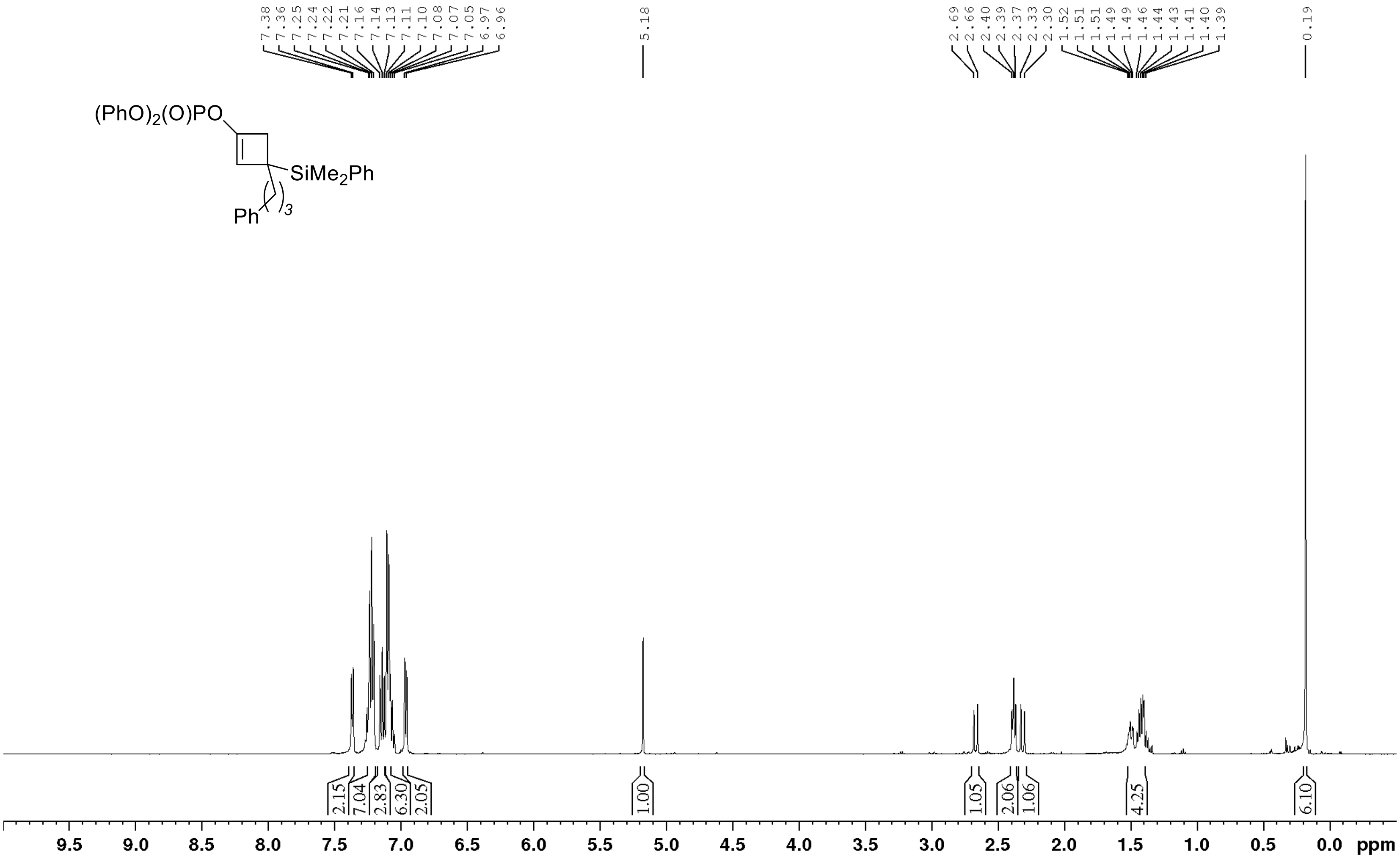

Figure S88. $^{13}C\{^{1}H\}$ NMR (126 MHz, $CDCl_3$, 298 K) of 3-(Dimethyl(phenyl)silyl)-3-(3-phenylpropyl)cyclobut-1-en-1-yl diphenyl phosphate (**3j**)

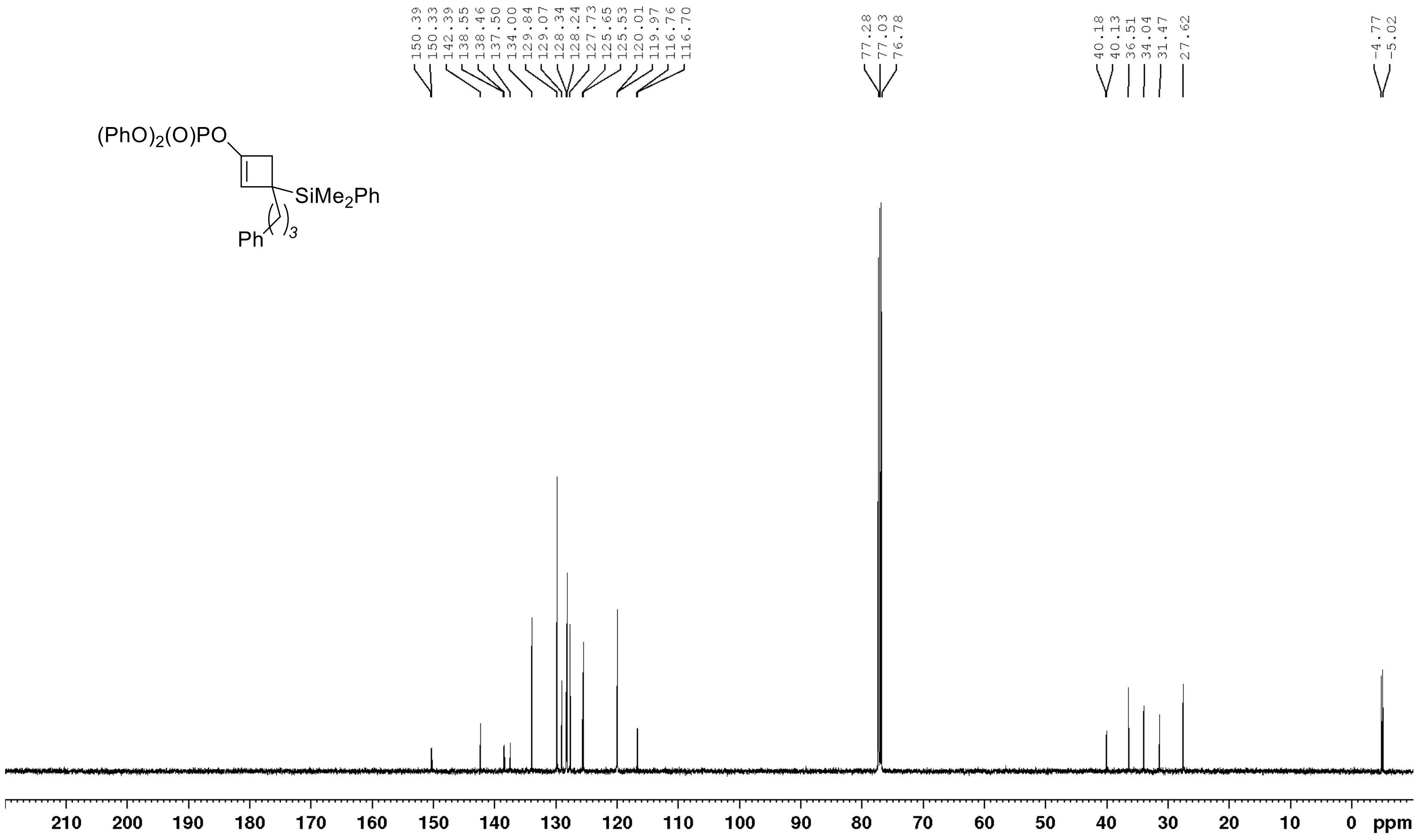

Figure S89. $^{29}Si\{^1H\}$ DEPT NMR (99 MHz, $CDCl_3$, 298 K) of 3-(Dimethyl(phenyl)silyl)-3-(3-phenylpropyl)cyclobut-1-en-1-yl diphenyl phosphate (**3j**)

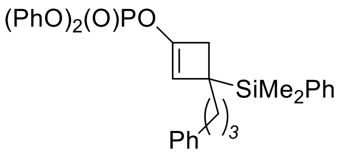

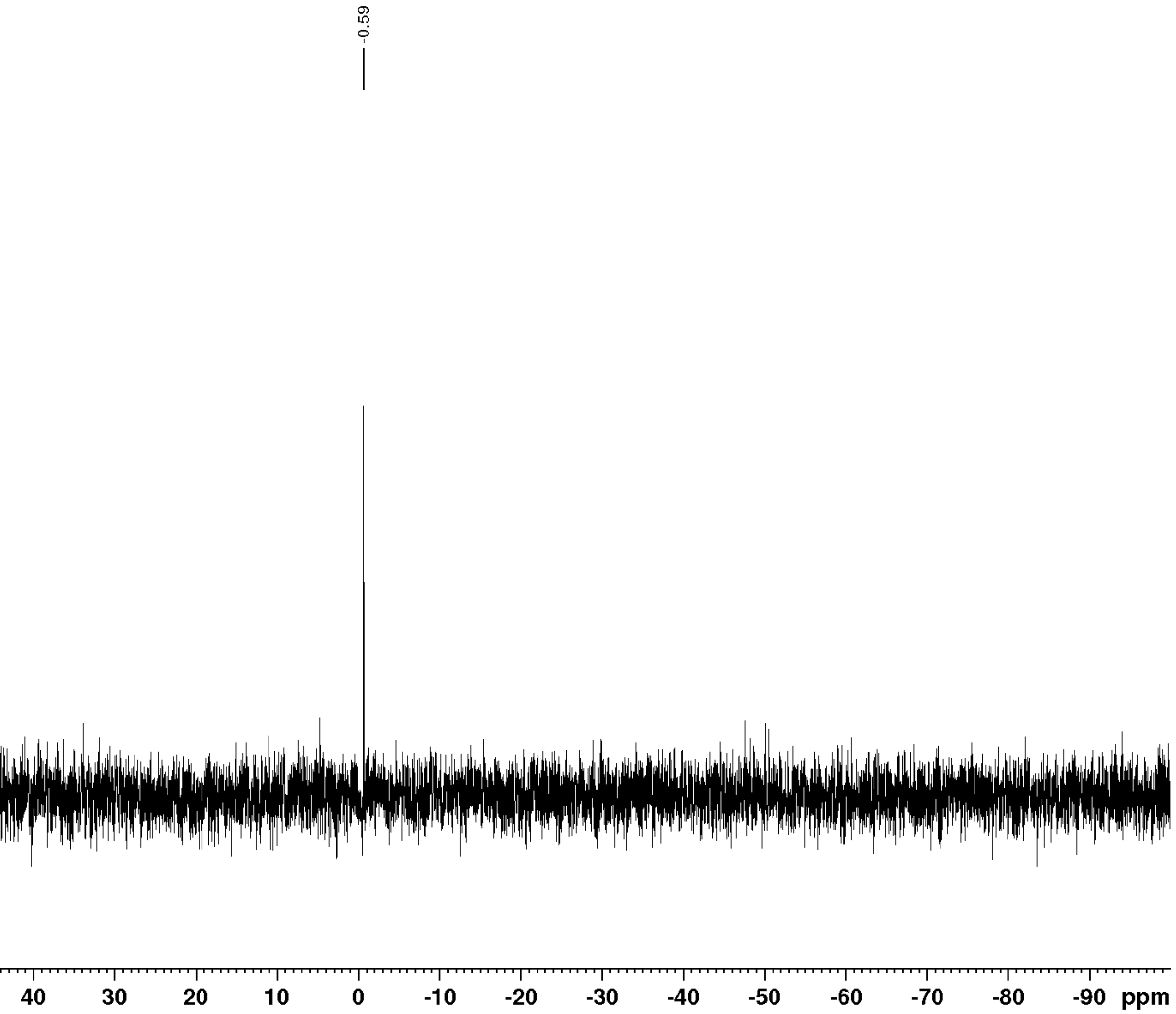

Figure S90. $^{31}P\{^1H\}$ NMR (202 MHz, $CDCl_3$, 298 K) of 3-(Dimethyl(phenyl)silyl)-3-(3-phenylpropyl)cyclobut-1-en-1-yl diphenyl phosphate (**3j**)

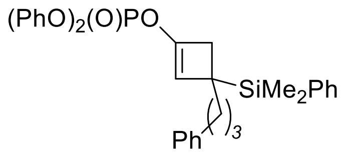

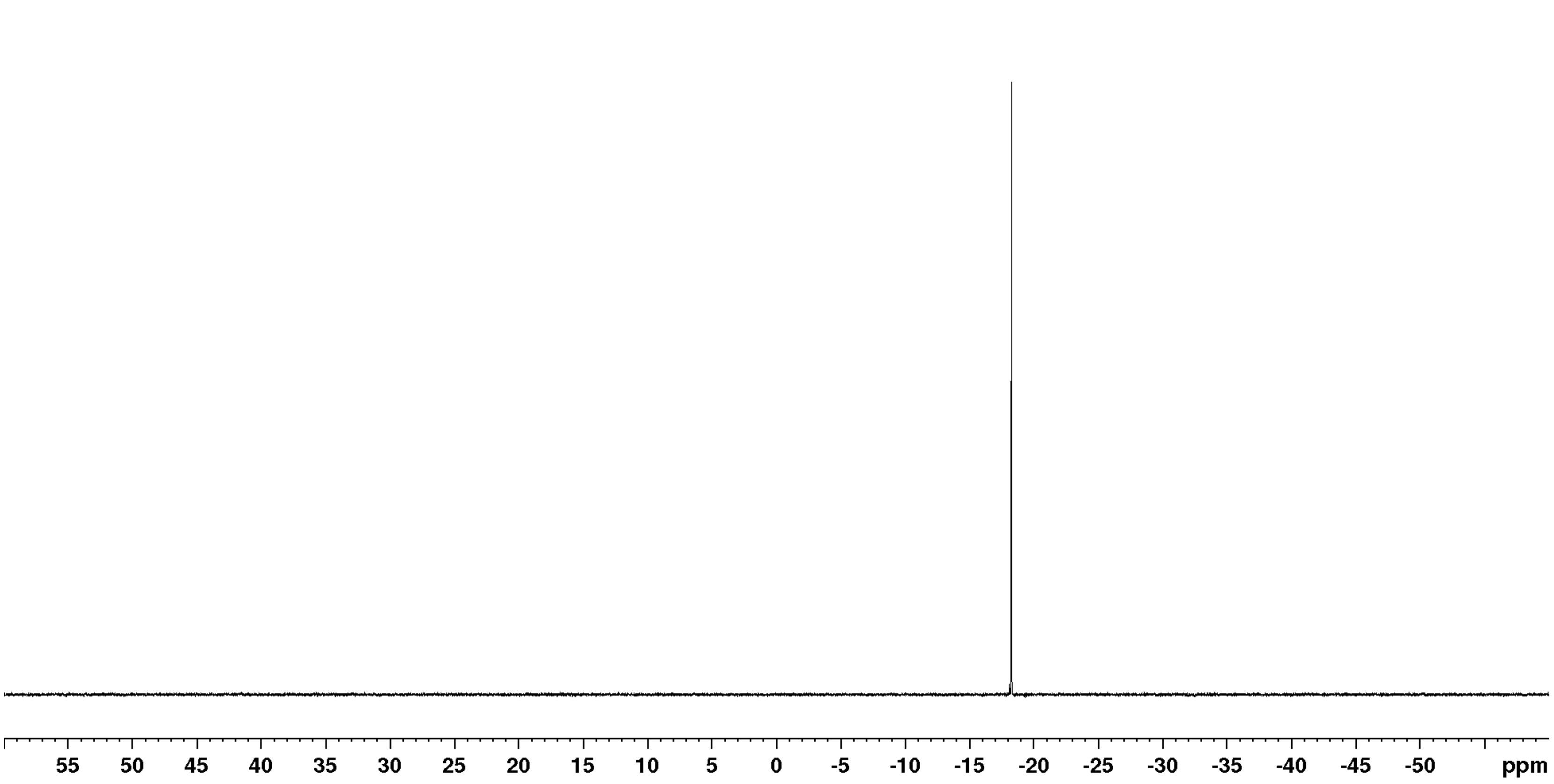

Figure S91. ^{1}H NMR (500 MHz, $CDCl_3$, 298 K) of 3-(4-Chlorobutyl)-3-(dimethyl(phenyl)silyl)cyclobut-1-en-1-yl diphenyl phosphate (**3k**)

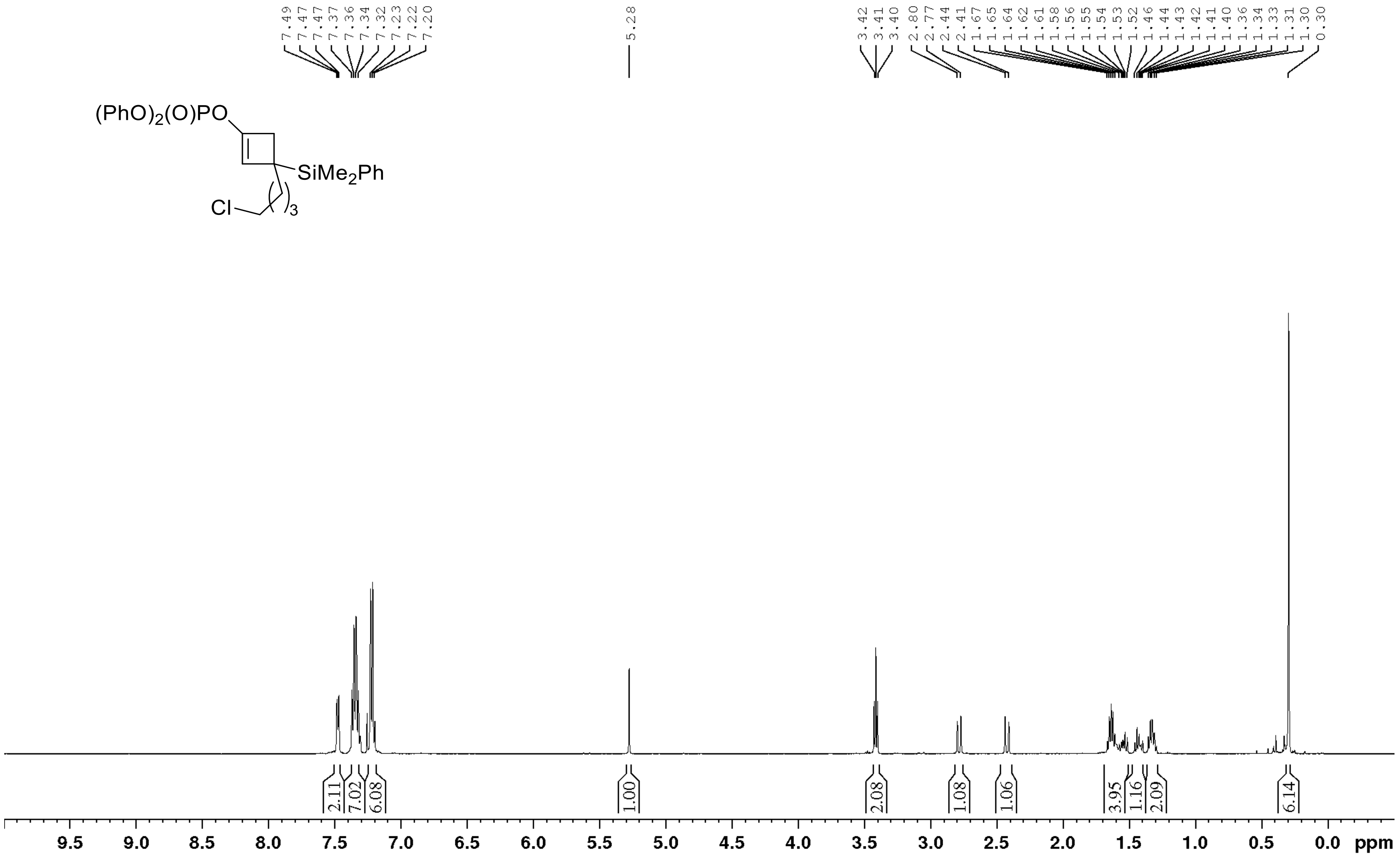

Figure S92. $^{13}C\{^{1}H\}$ NMR (126 MHz, $CDCl_3$, 298 K) of 3-(4-Chlorobutyl)-3-(dimethyl(phenyl)silyl)cyclobut-1-en-1-yl diphenyl phosphate (**3k**)

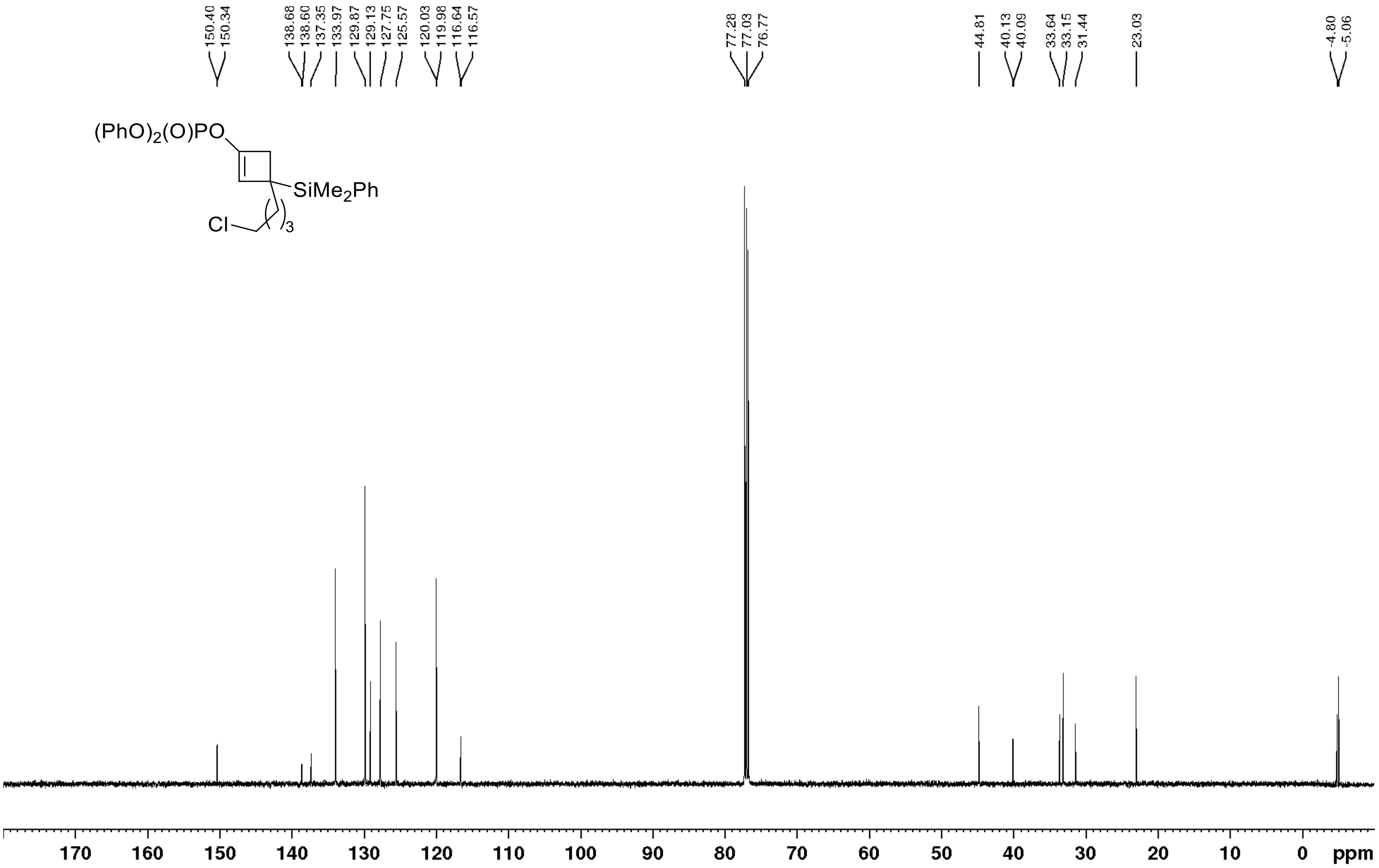

Figure S93. $^{29}Si\{^1H\}$ DEPT NMR (99 MHz, $CDCl_3$, 298 K) of 3-(4-Chlorobutyl)-3-(dimethyl(phenyl)silyl)cyclobut-1-en-1-yl diphenyl phosphate (**3k**)

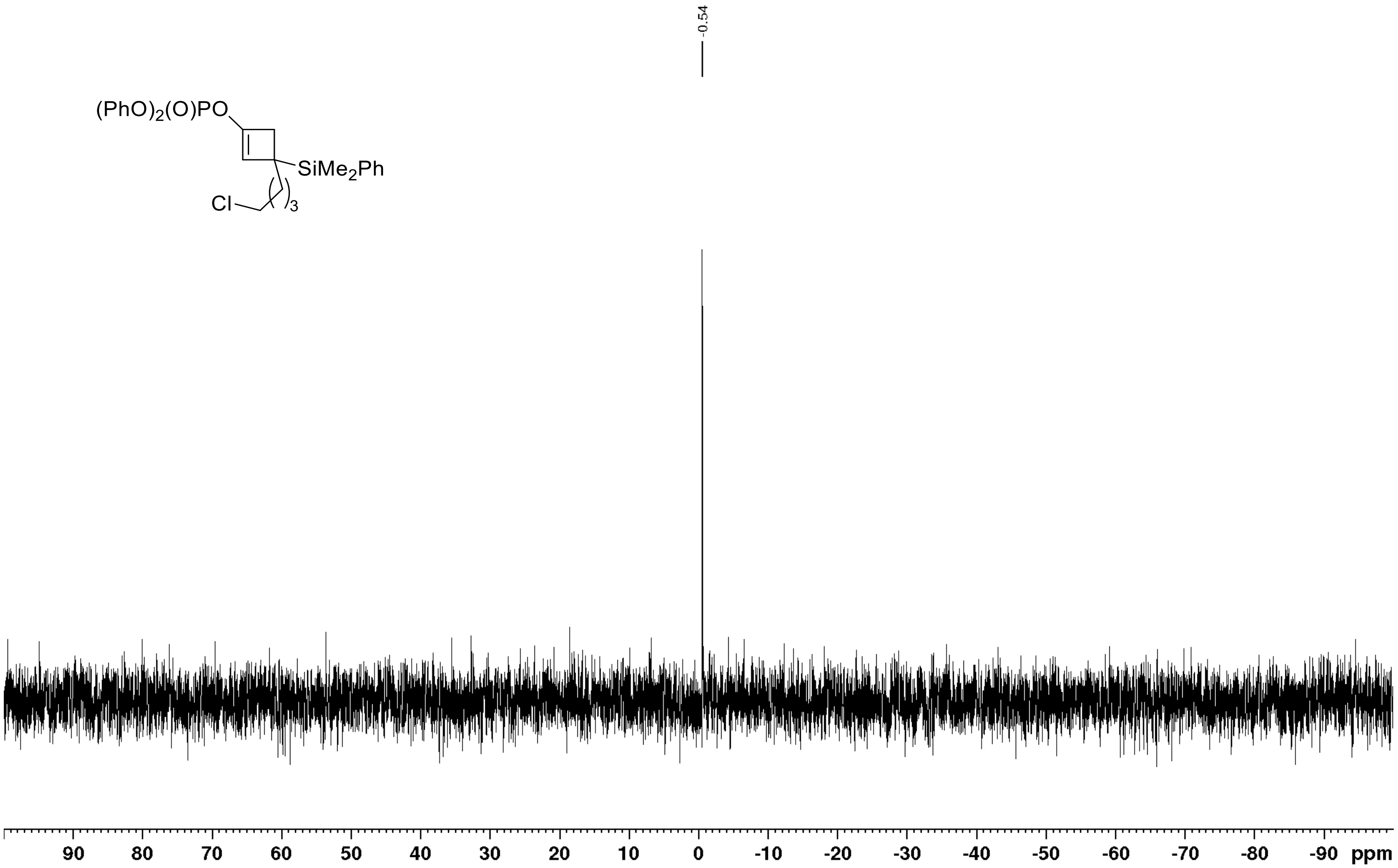

Figure S94. $^{31}P\{^{1}H\}$ NMR (202 MHz, $CDCl_3$, 298 K) of 3-(4-Chlorobutyl)-3-(dimethyl(phenyl)silyl)cyclobut-1-en-1-yl diphenyl phosphate (**3k**)

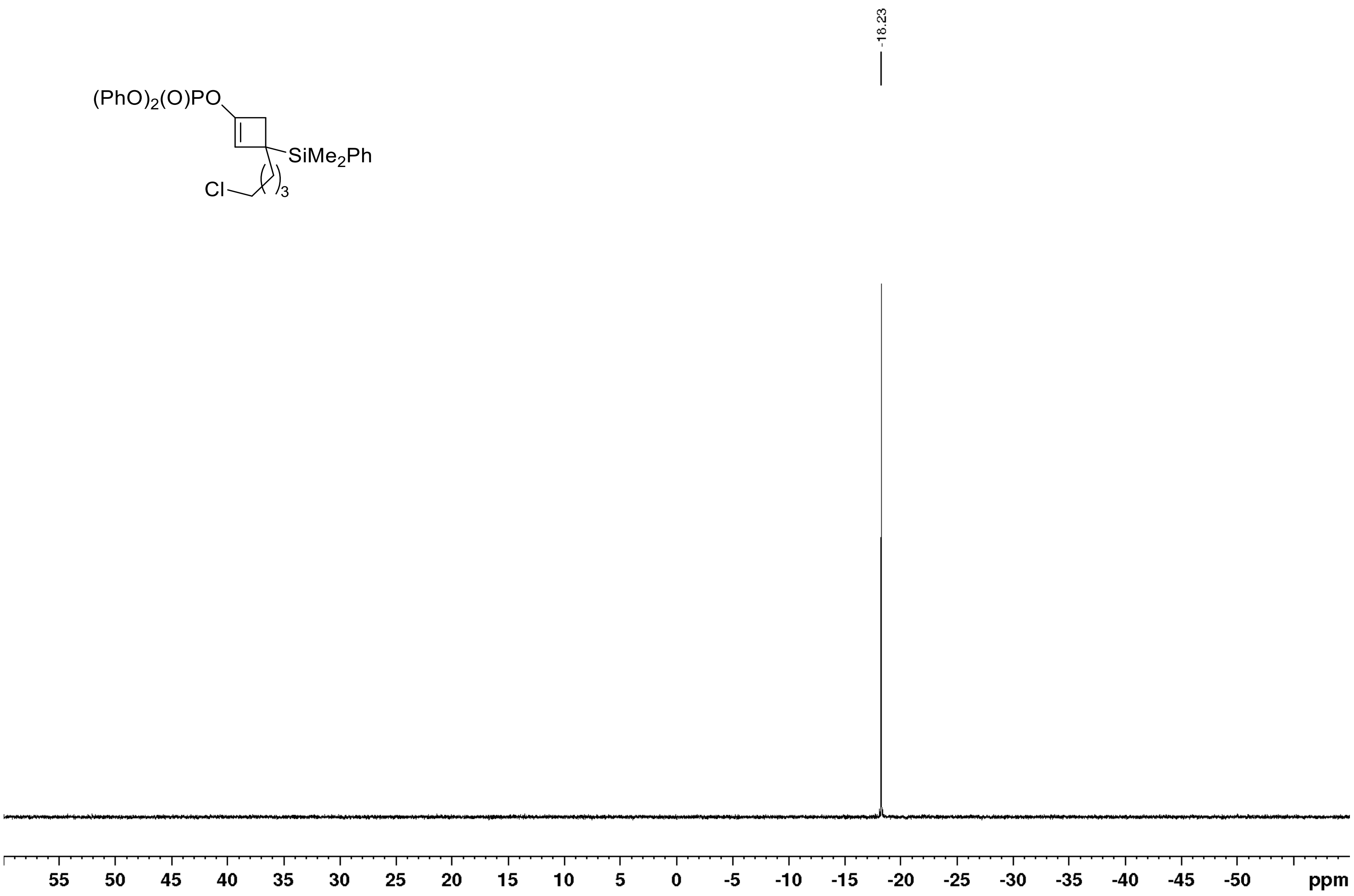

Figure S95. ^{1}H NMR (500 MHz, $CDCl_3$, 298 K) of 3-Cyclopropyl-3-(dimethyl(phenyl)silyl)cyclobut-1-en-1-yl diphenyl phosphate (**3l**)

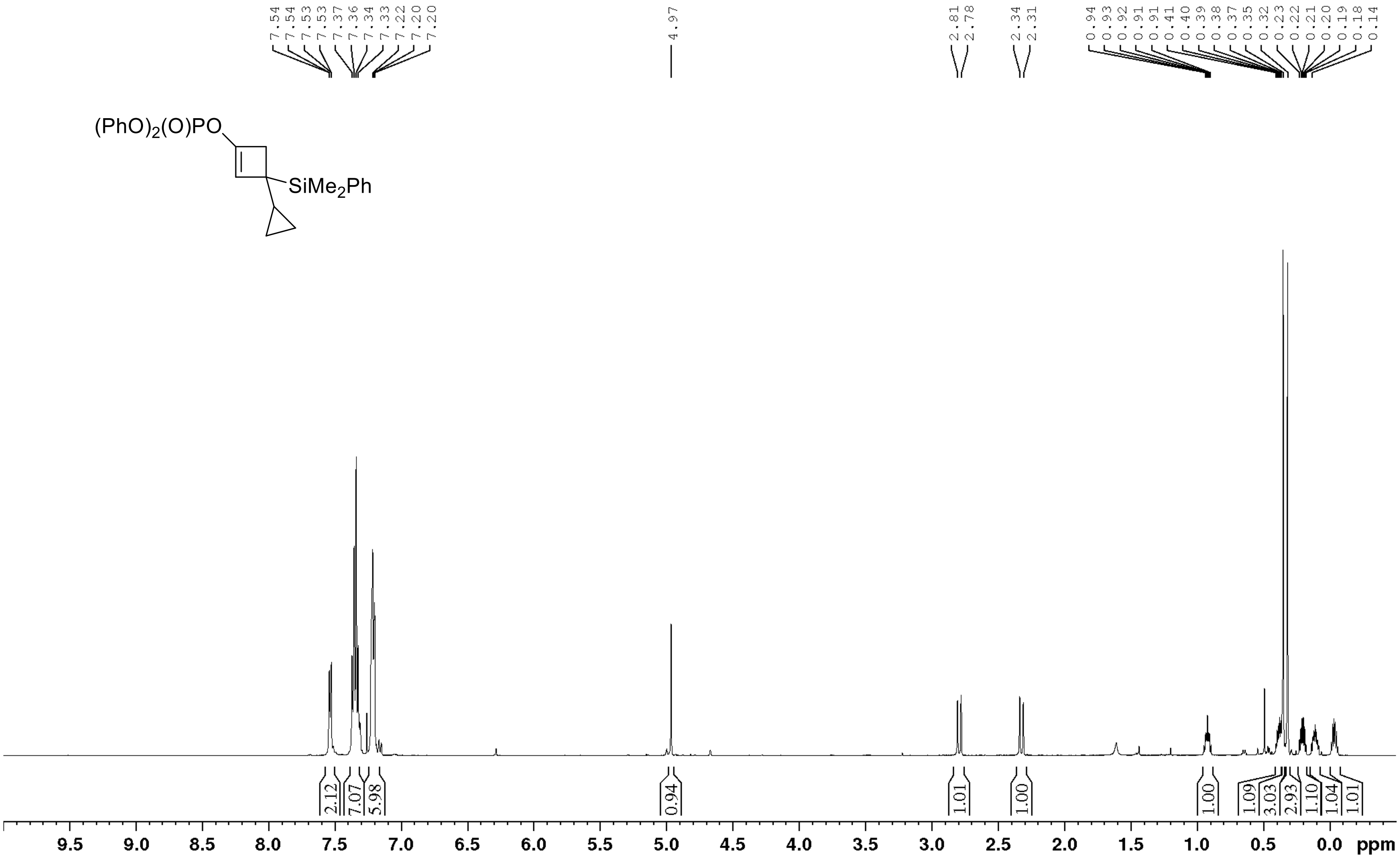

Figure S96. $^{13}C\{^{1}H\}$ NMR (126 MHz, $CDCl_3$, 298 K) of 3-Cyclopropyl-3-(dimethyl(phenyl)silyl)cyclobut-1-en-1-yl diphenyl phosphate (**3l**)

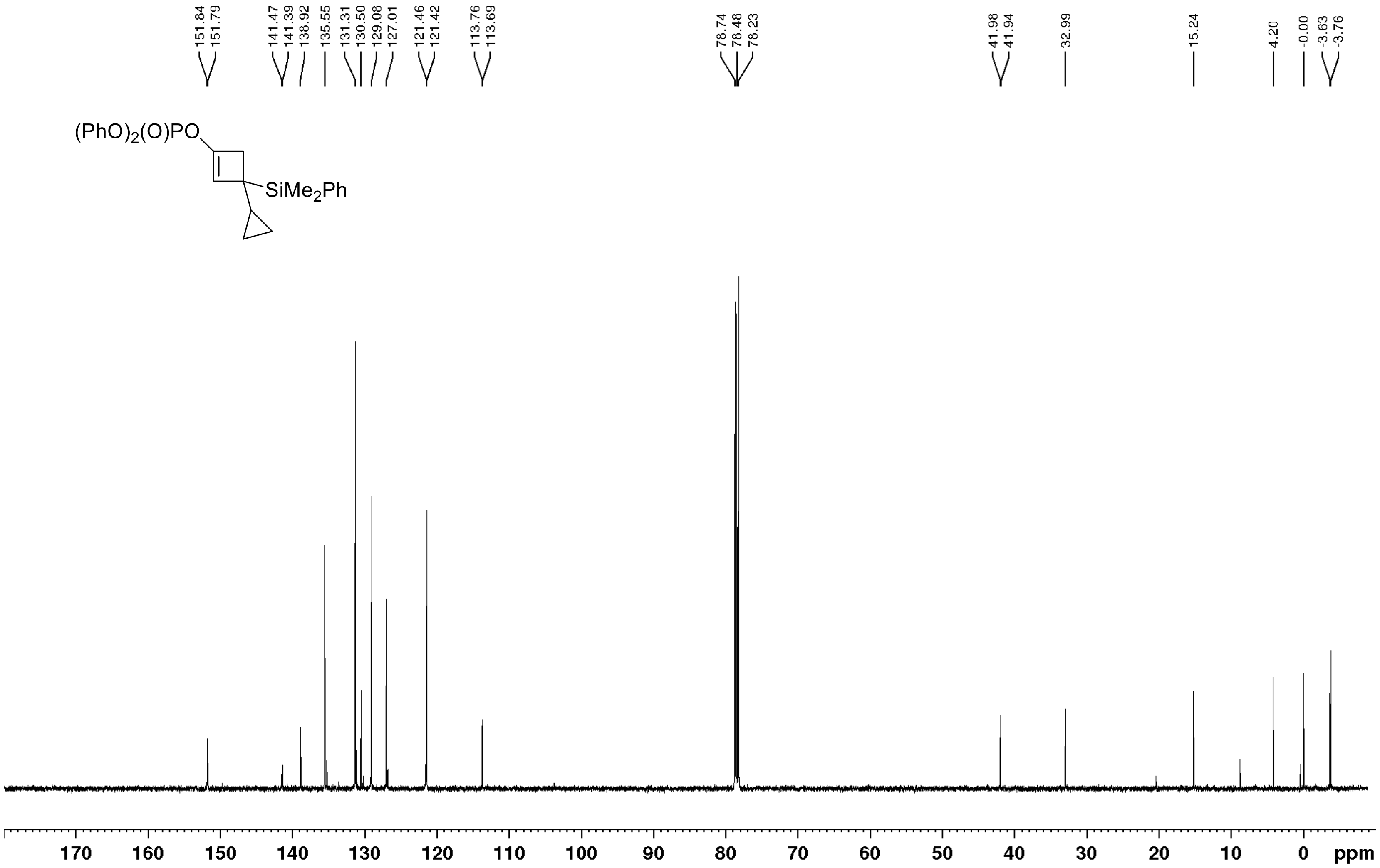

Figure S97. $^{29}Si\{^{1}H\}$ DEPT NMR (99 MHz, $CDCl_3$, 298 K) of 3-Cyclopropyl-3-(dimethyl(phenyl)silyl)cyclobut-1-en-1-yl diphenyl phosphate (**3l**)

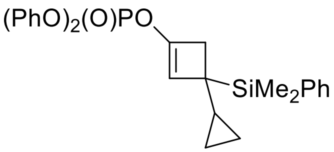

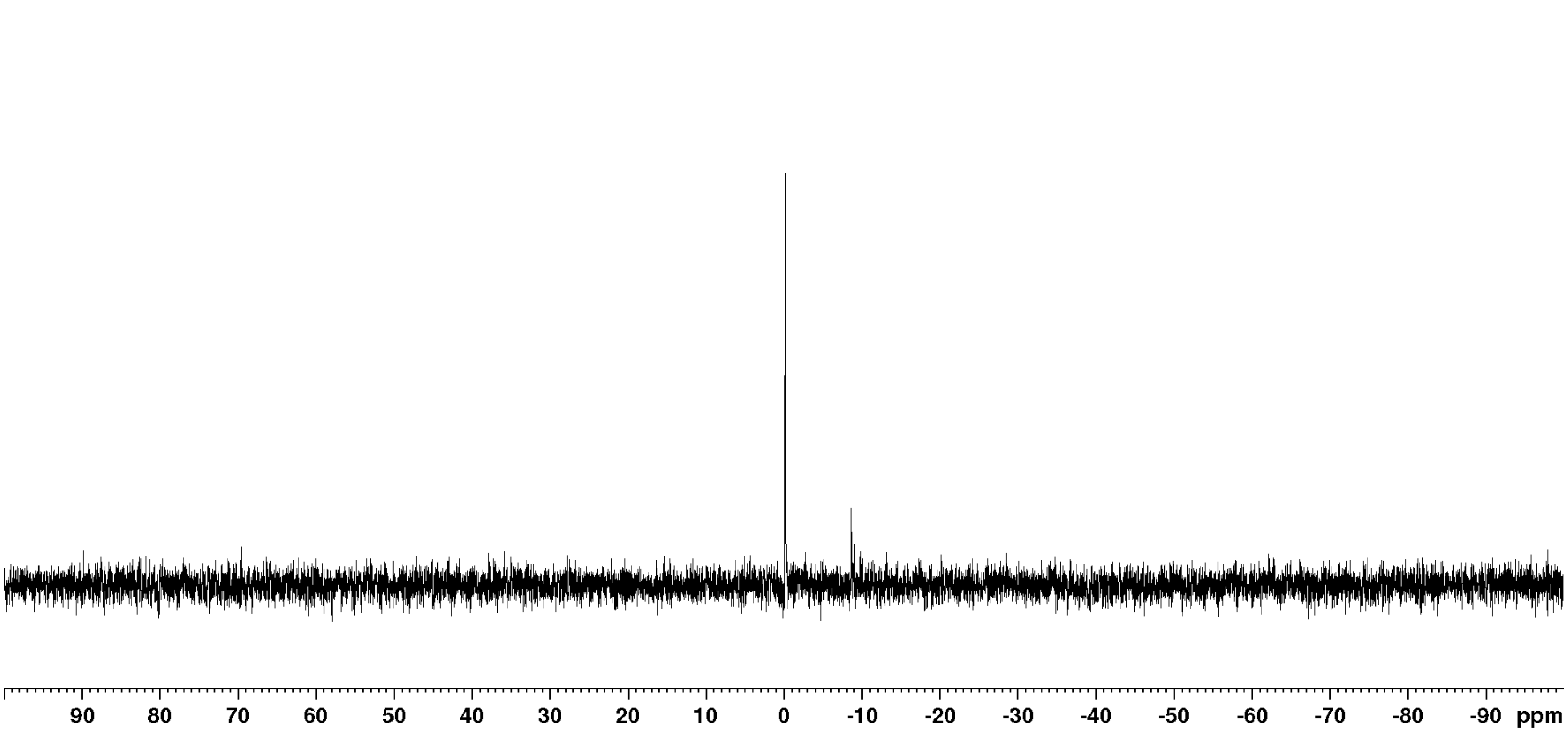

Figure S98. $^{31}P\{^{1}H\}$ NMR (202 MHz, $CDCl_3$, 298 K) of 3-Cyclopropyl-3-(dimethyl(phenyl)silyl)cyclobut-1-en-1-yl diphenyl phosphate (**3l**)

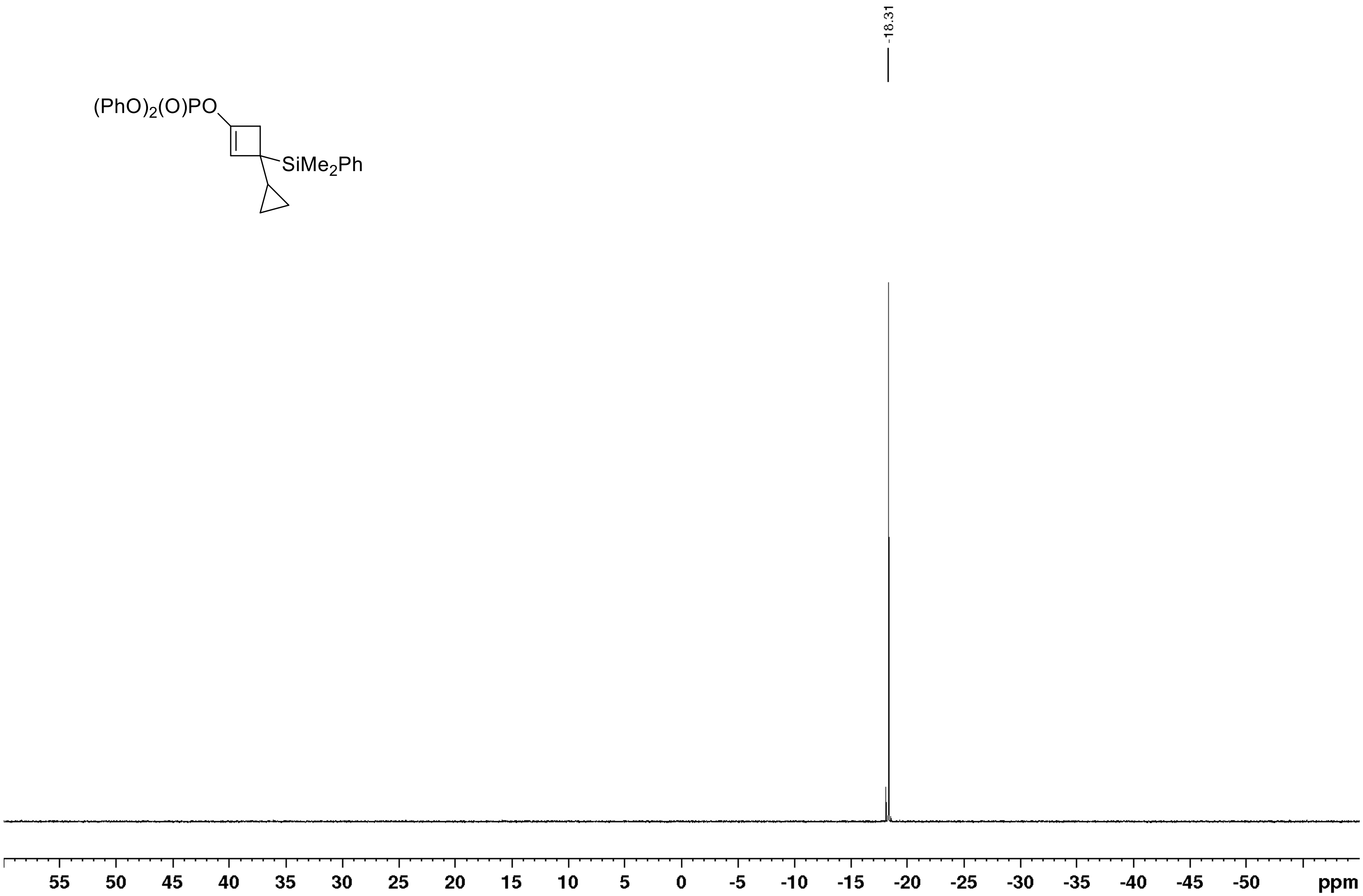

Figure S99. ^{1}H NMR (500 MHz, $CDCl_3$, 298 K) of 3-Cyclohexyl-3-(dimethyl(phenyl)silyl)cyclobut-1-en-1-yl diphenyl phosphate (**3m**)

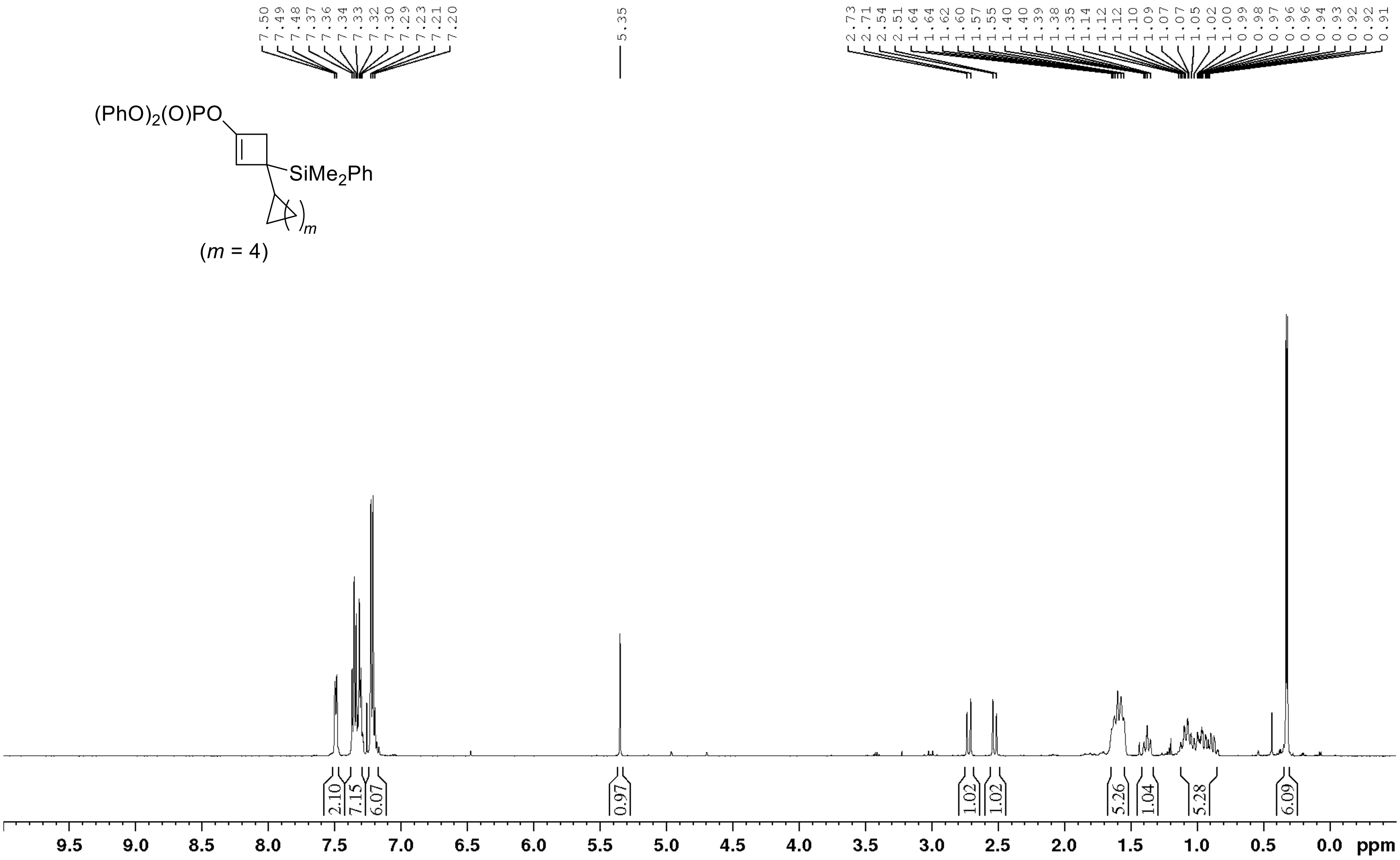

Figure S100. $^{13}C\{^1H\}$ NMR (126 MHz, $CDCl_3$, 298 K) of 3-Cyclohexyl-3-(dimethyl(phenyl)silyl)cyclobut-1-en-1-yl diphenyl phosphate (**3m**)

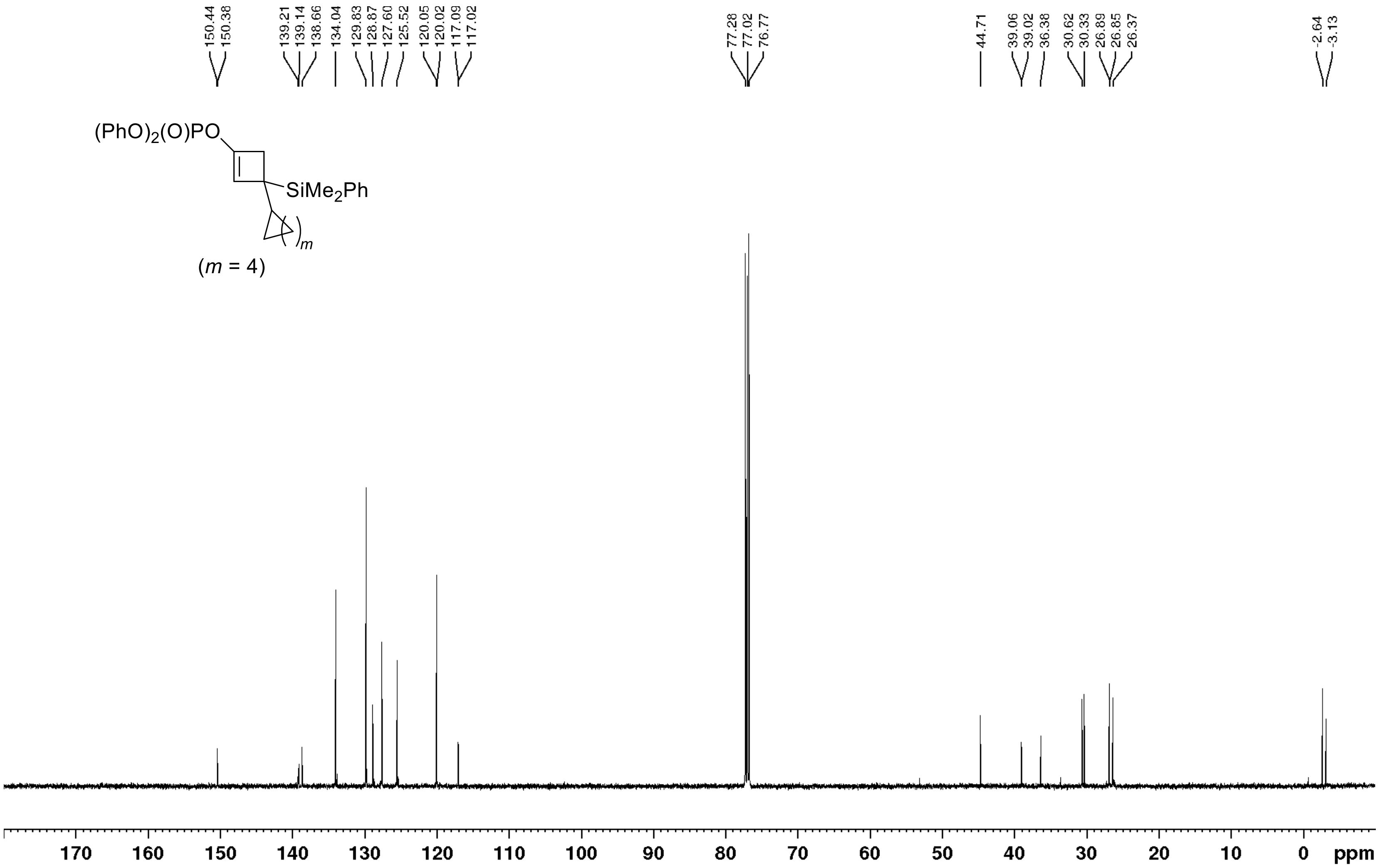

Figure S101. ^{29}Si{^{1}H} DEPT NMR (99 MHz, $CDCl_3$, 298 K) of 3-Cyclohexyl-3-(dimethyl(phenyl)silyl)cyclobut-1-en-1-yl diphenyl phosphate (**3m**)

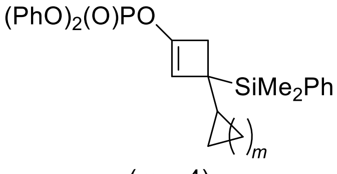

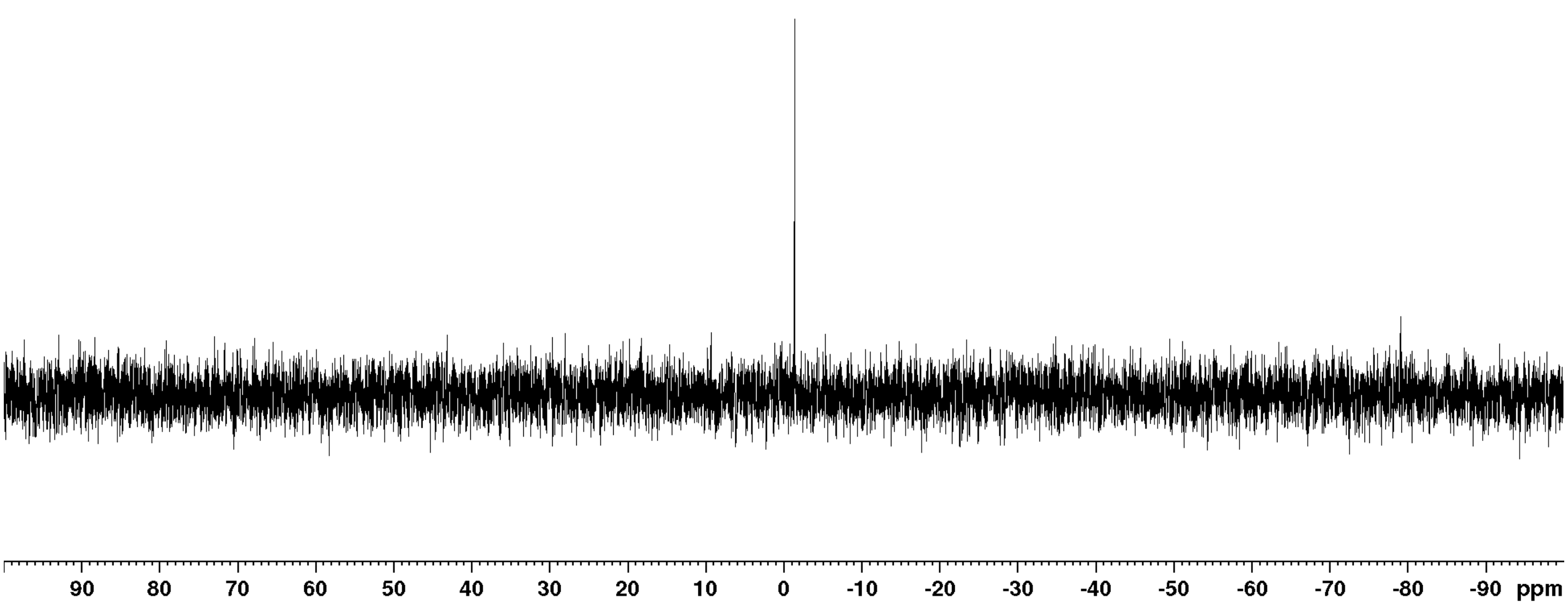

Figure S102. $^{31}P\{^{1}H\}$ NMR (202 MHz, $CDCl_3$, 298 K) of 3-Cyclohexyl-3-(dimethyl(phenyl)silyl)cyclobut-1-en-1-yl diphenyl phosphate (**3m**)

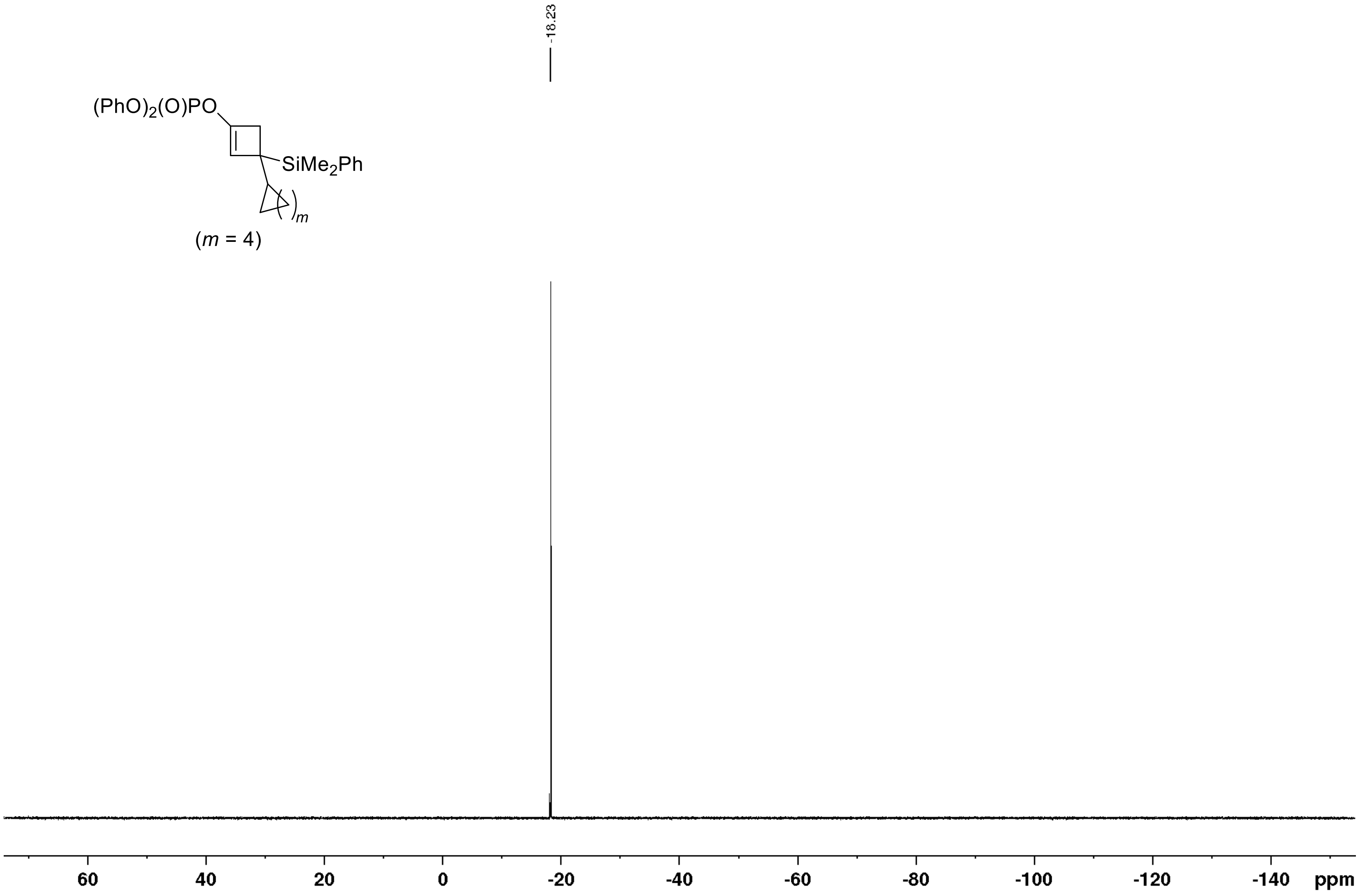

Figure S103. ^{1}H NMR (500 MHz, $CDCl_3$, 298 K) of 3-(Dimethyl(phenyl)silyl)-3-(trimethylsilyl)cyclobut-1-en-1-yl diphenyl phosphate (**3n**)

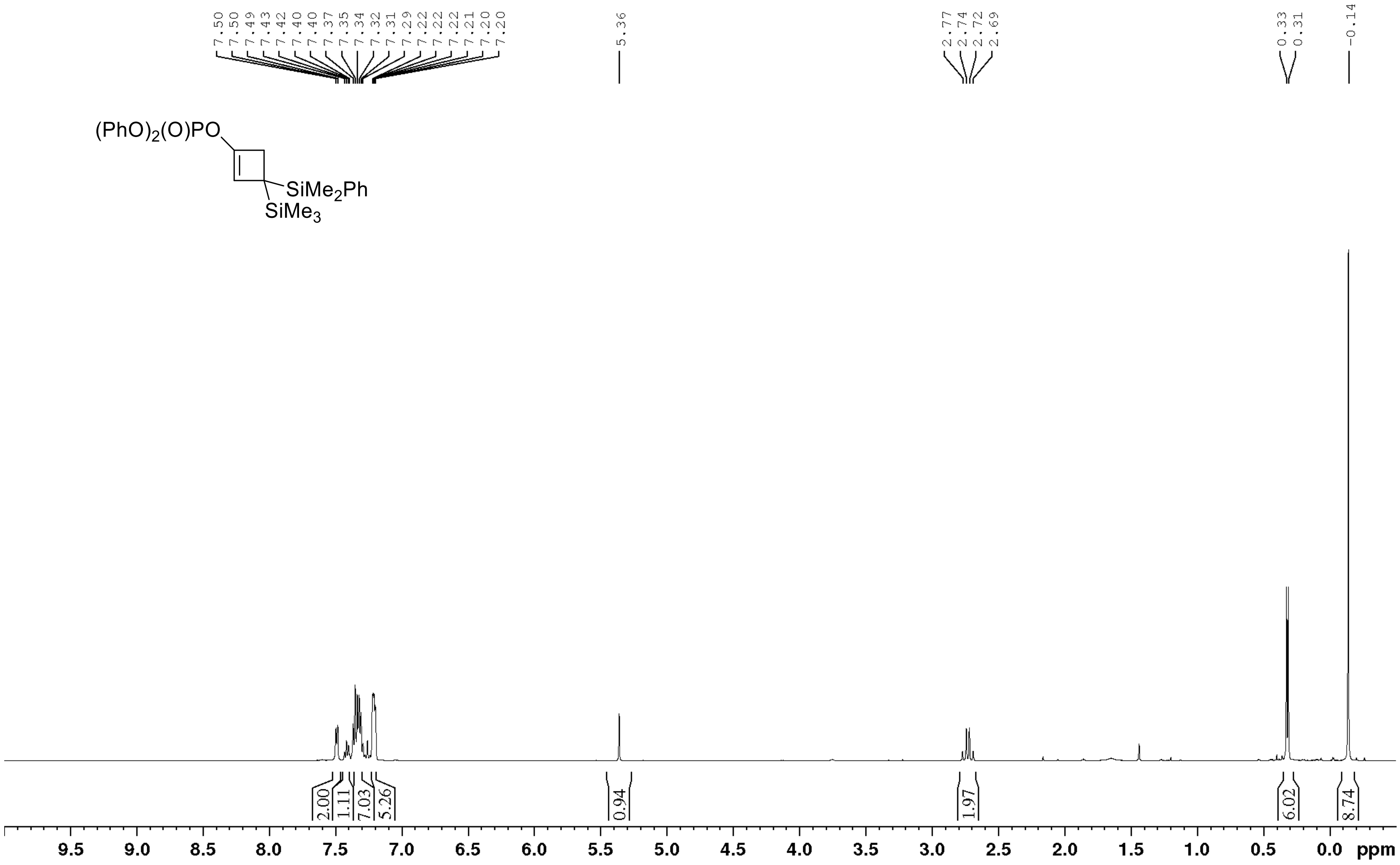

Figure S104. $^{13}C\{^1H\}$ NMR (500 MHz, $CDCl_3$, 298 K) of 3-(Dimethyl(phenyl)silyl)-3-(trimethylsilyl)cyclobut-1-en-1-yl diphenyl phosphate (**3n**)

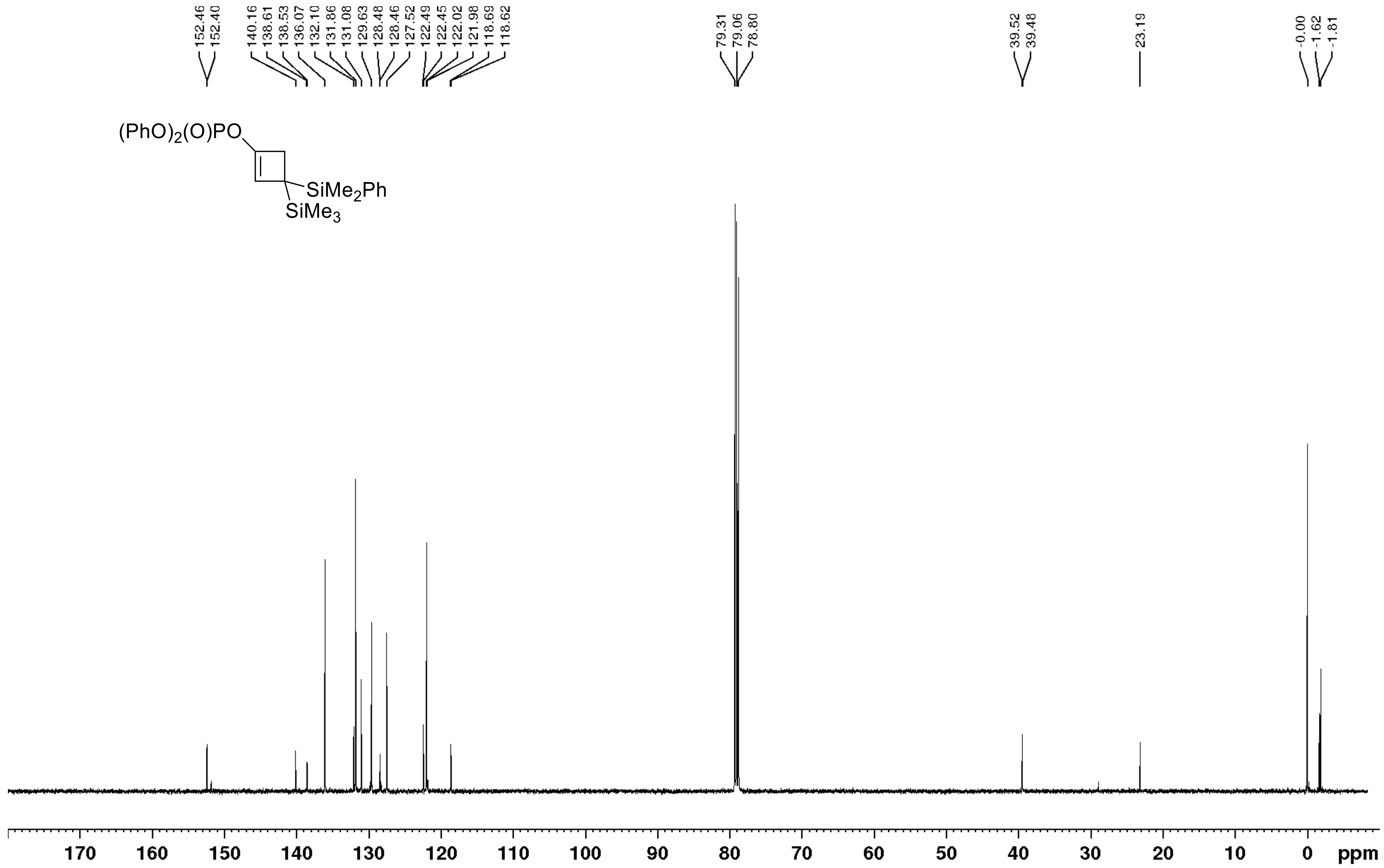

Figure S105. $^{29}Si\{^{1}H\}$ DEPT NMR (99 MHz, $CDCl_3$, 298 K) of 3-(Dimethyl(phenyl)silyl)-3-(trimethylsilyl)cyclobut-1-en-1-yl diphenyl phosphate (**3n**)

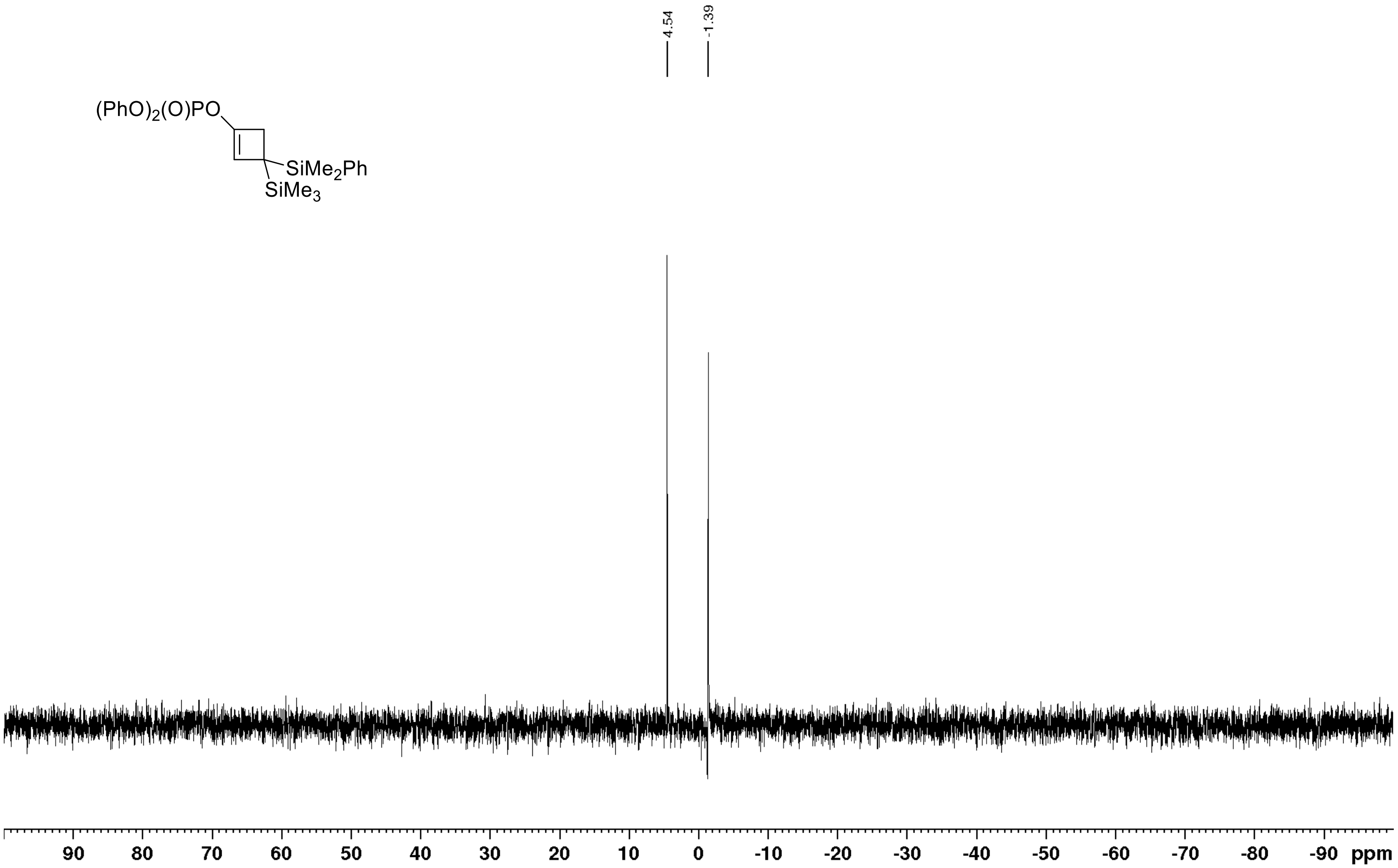

Figure S106. $^{31}P\{^{1}H\}$ NMR (202 MHz, $CDCl_3$, 298 K) of 3-(Dimethyl(phenyl)silyl)-3-(trimethylsilyl)cyclobut-1-en-1-yl diphenyl phosphate (**3n**)

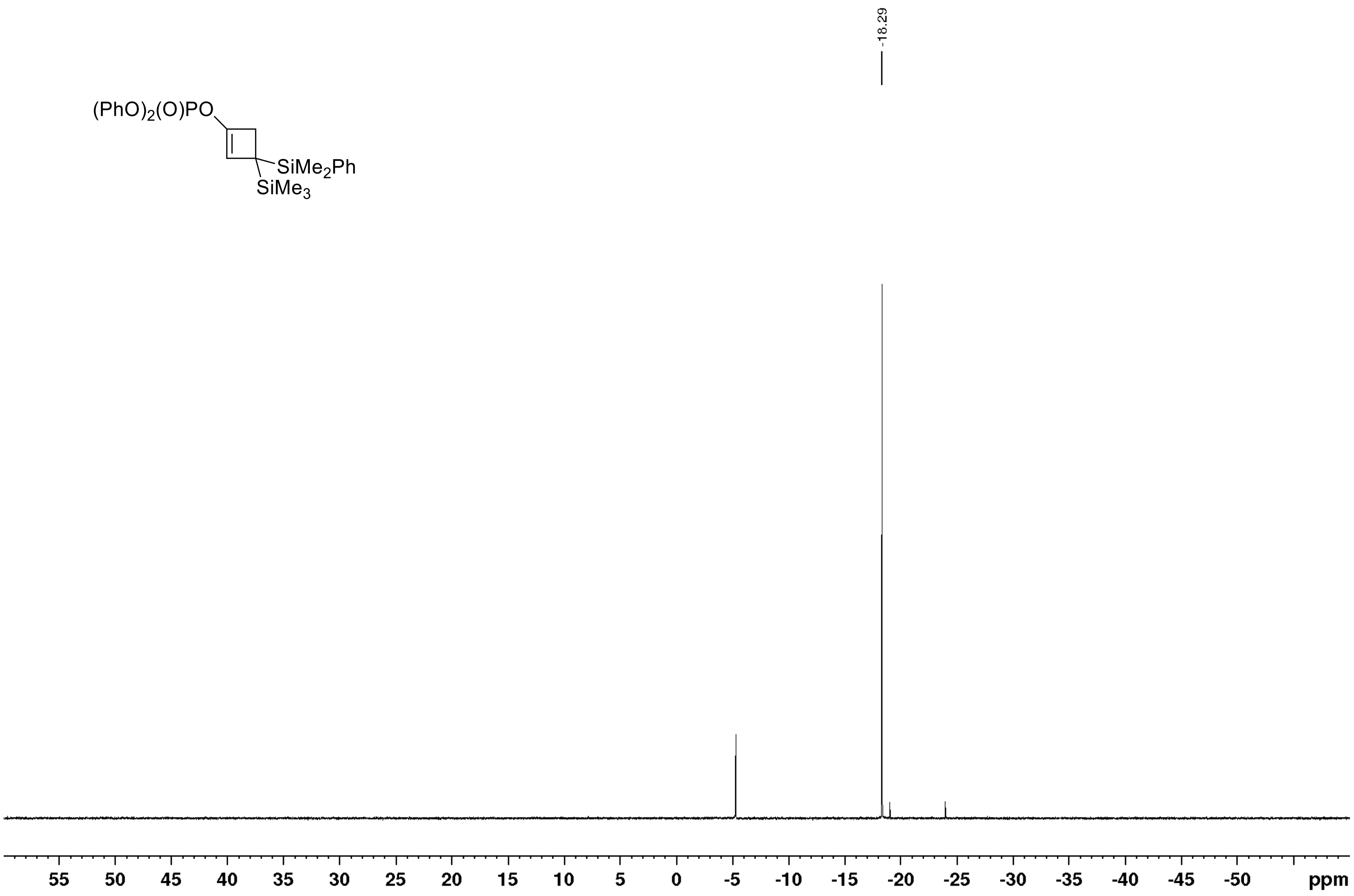

Figure S107. ^{1}H NMR (500 MHz, $CDCl_3$, 298 K) of 3-(Dimethyl(phenyl)silyl)-2-methyl-3-phenylcyclobut-1-en-1-yl diphenyl phosphate (**3o**)

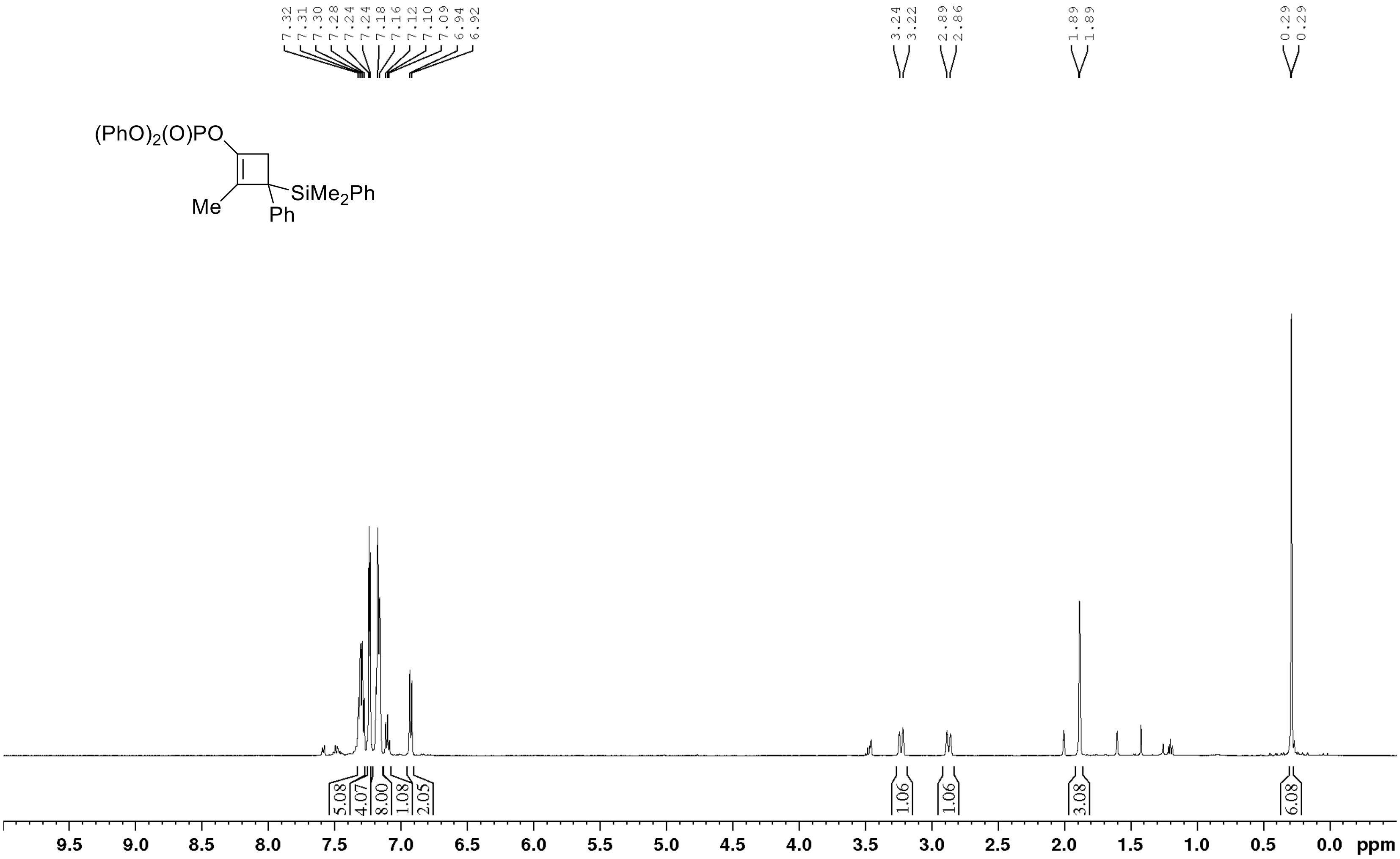

Figure S108. $^{13}C\{^{1}H\}$ NMR (126 MHz, $CDCl_3$, 298 K) of 3-(Dimethyl(phenyl)silyl)-2-methyl-3-phenylcyclobut-1-en-1-yl diphenyl phosphate (**3o**)

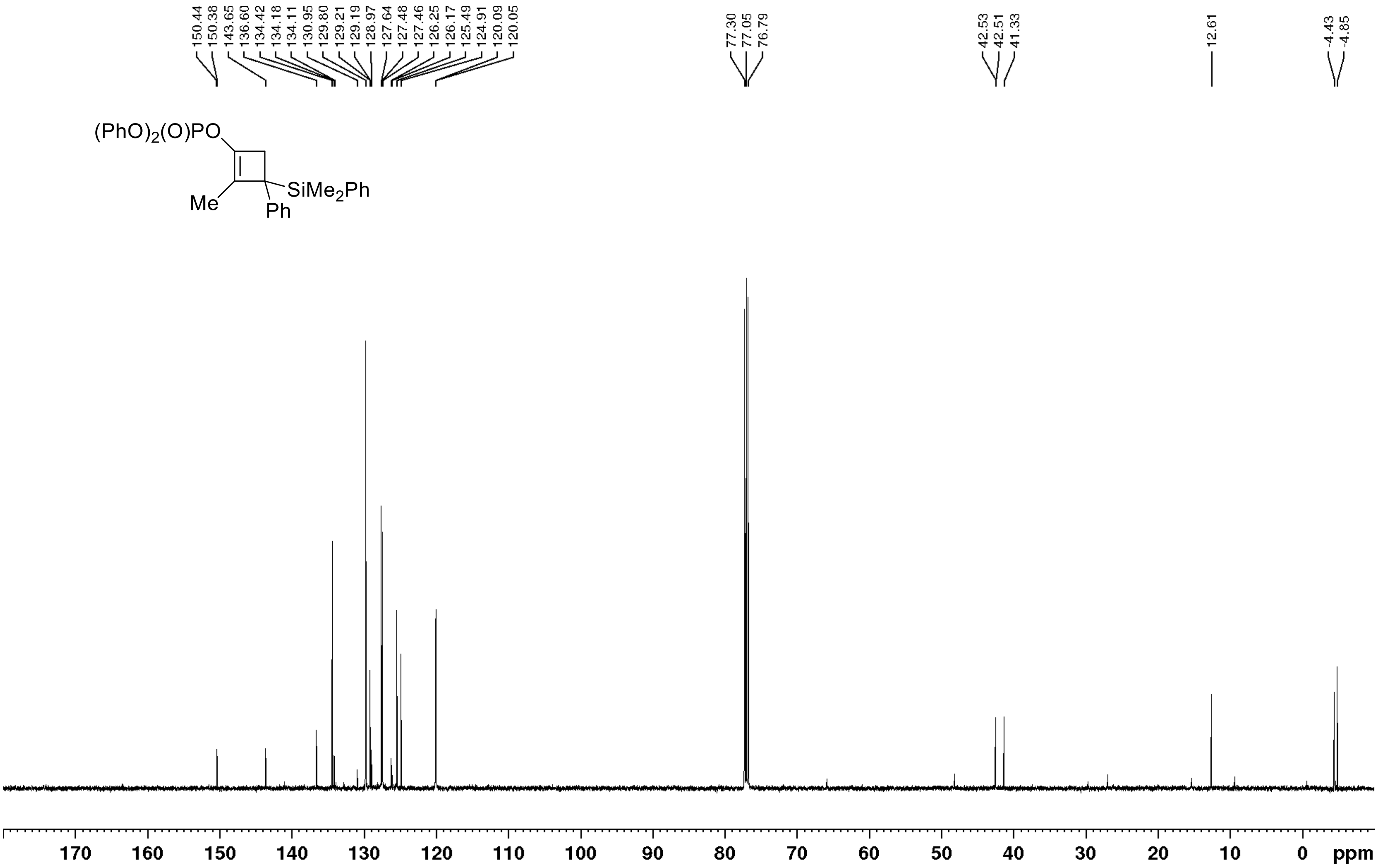

Figure S109. $^{29}Si\{^{1}H\}$ DEPT NMR (99 MHz, $CDCl_3$, 298 K) of 3-(Dimethyl(phenyl)silyl)-2-methyl-3-phenylcyclobut-1-en-1-yl diphenyl phosphate (**3o**)

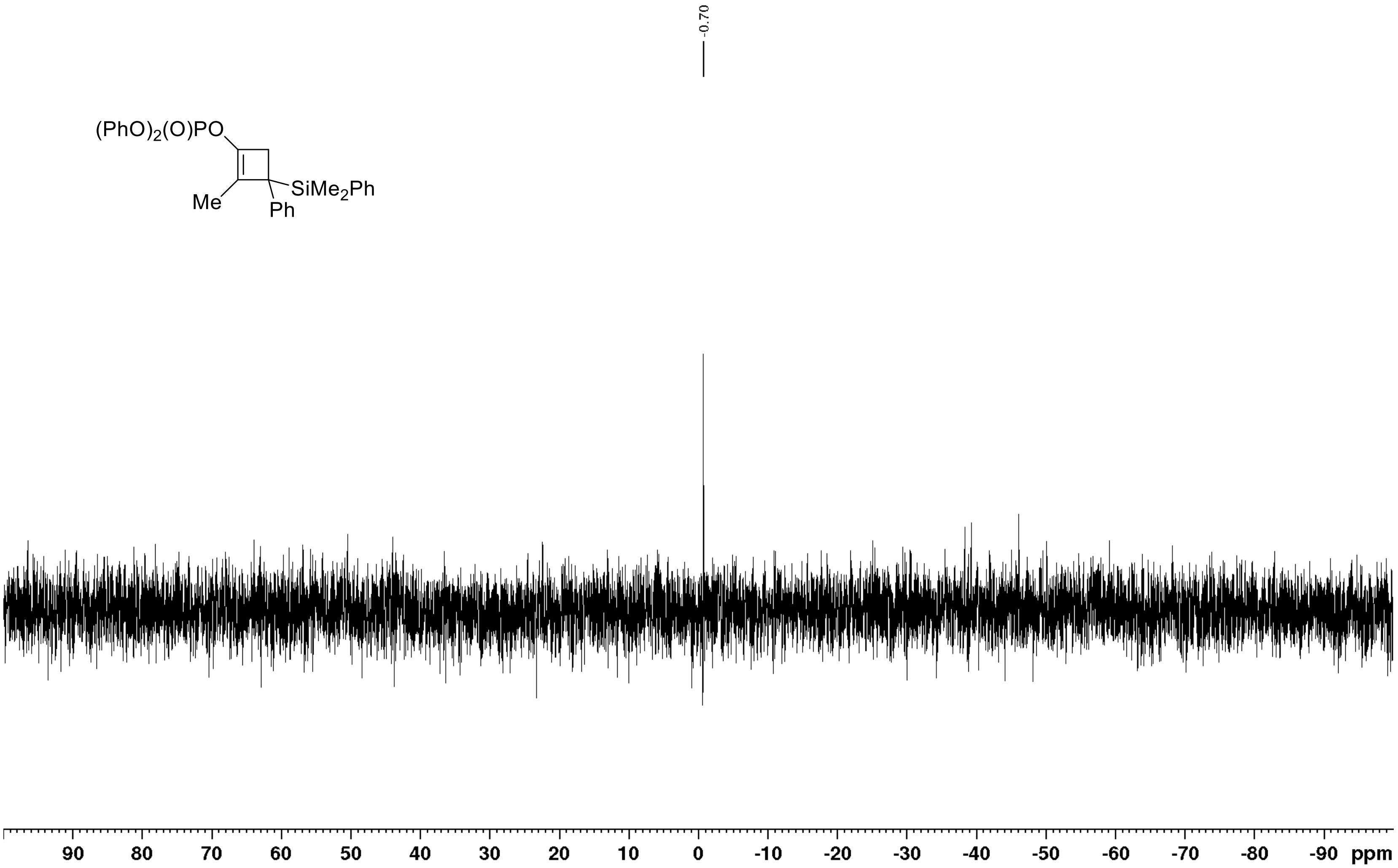

Figure S110. $^{31}P\{^{1}H\}$ NMR (202 MHz, $CDCl_3$, 298 K) of 3-(Dimethyl(phenyl)silyl)-2-methyl-3-phenylcyclobut-1-en-1-yl diphenyl phosphate (**3o**)

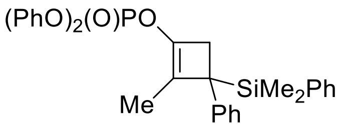

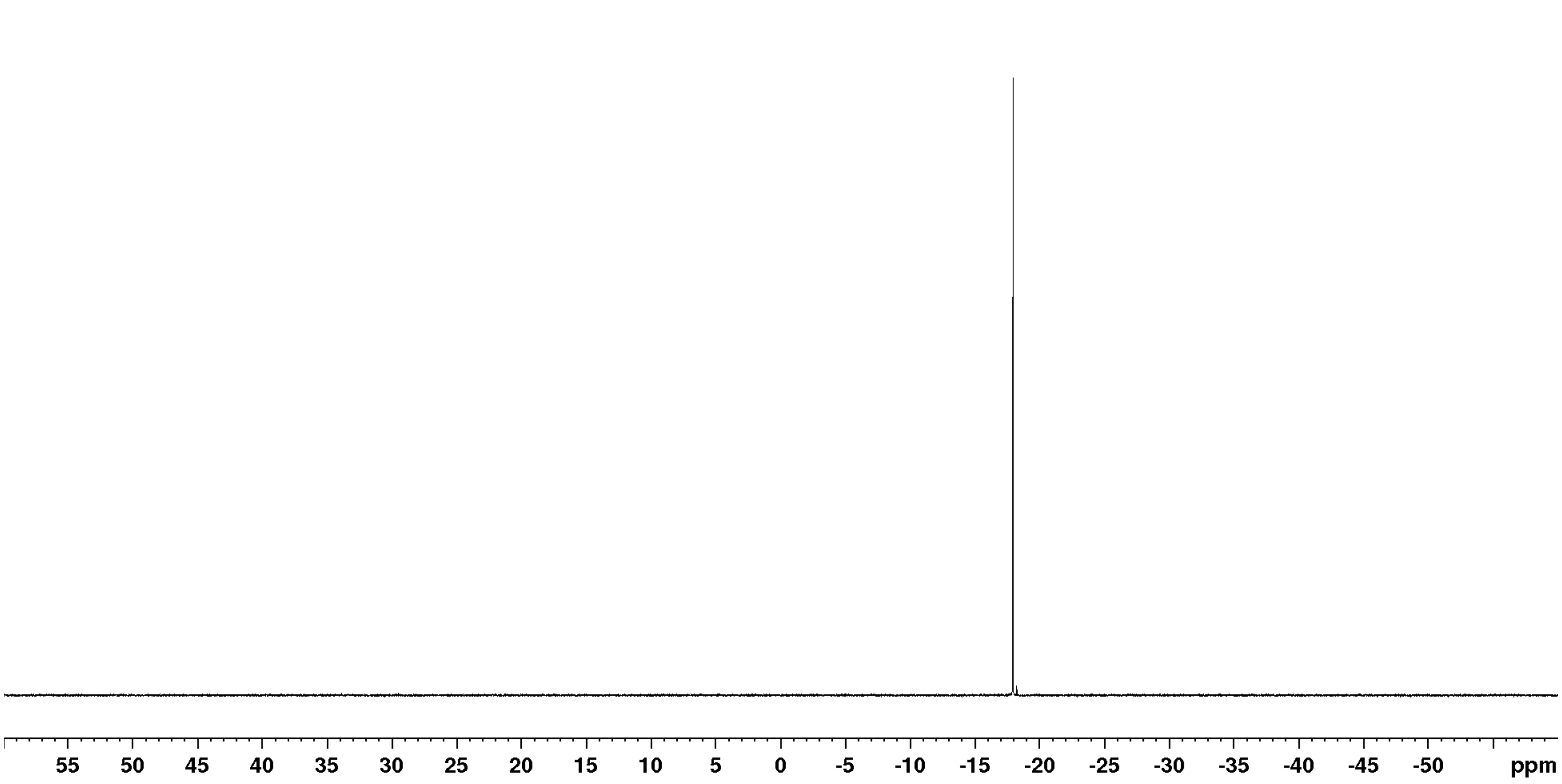

Figure S111. ^{1}H NMR (500 MHz, $CDCl_3$, 298 K) of 3-(Dimethyl(phenyl)silyl)-2,3-dipropylcyclobut-1-en-1-yl diphenyl phosphate (**3p**)

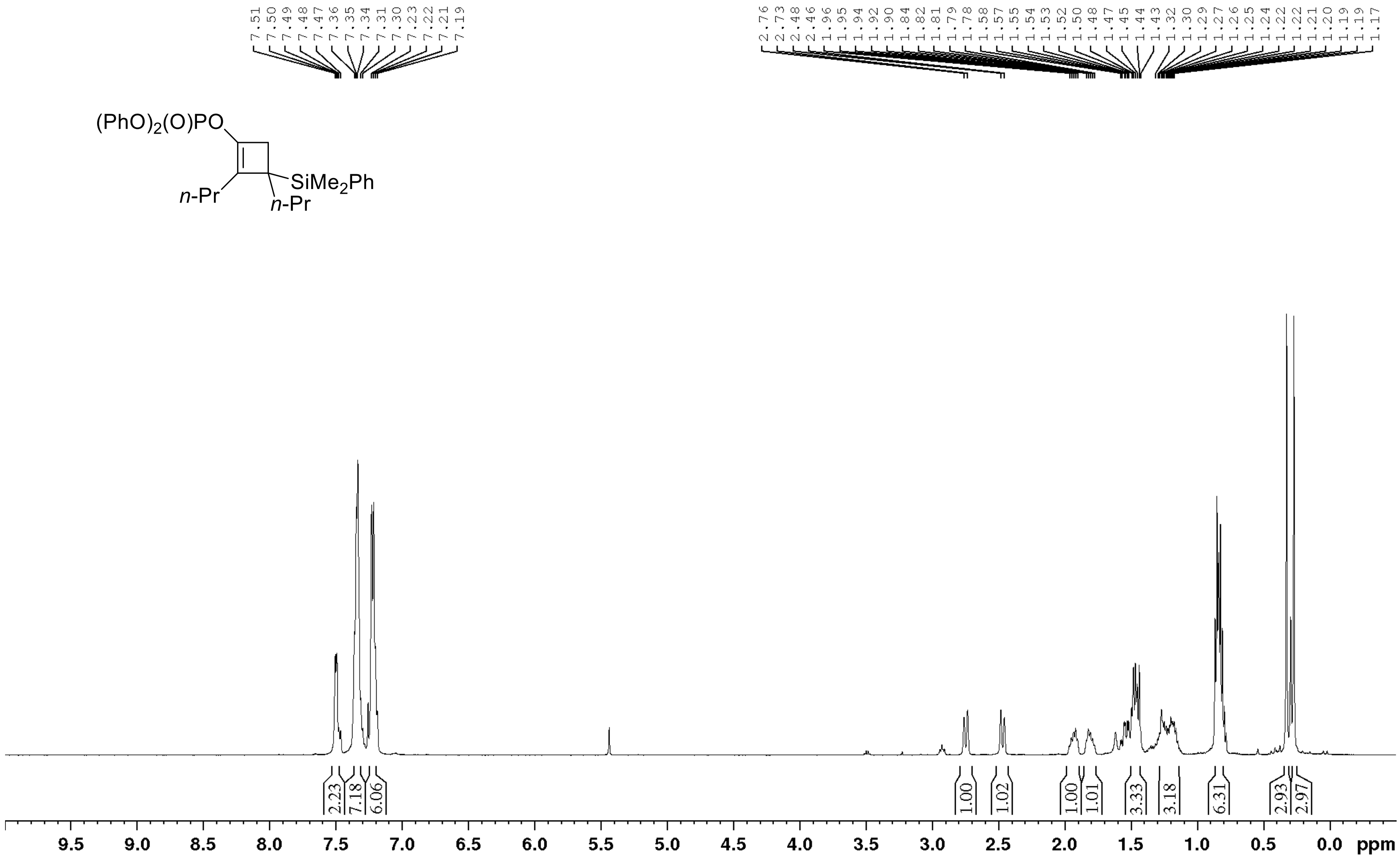

Figure S112. $^{13}C\{^1H\}$ NMR (126 MHz, $CDCl_3$, 298 K) of 3-(Dimethyl(phenyl)silyl)-2,3-dipropylcyclobut-1-en-1-yl diphenyl phosphate (**3p**)

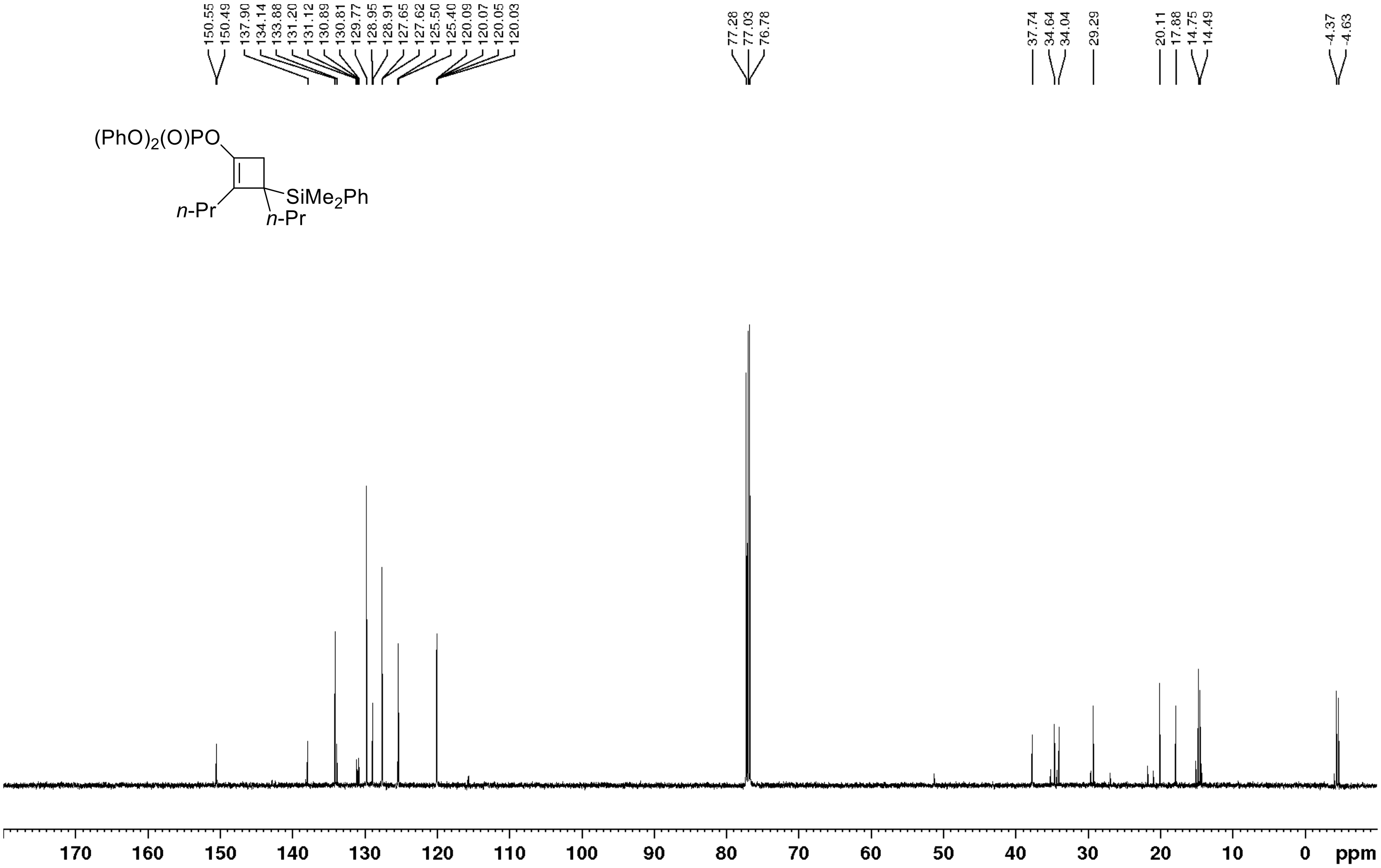

Figure S113. $^{29}Si\{^1H\}$ DEPT NMR (99 MHz, $CDCl_3$, 298 K) of 3-(Dimethyl(phenyl)silyl)-2,3-dipropylcyclobut-1-en-1-yl diphenyl phosphate (**3p**)

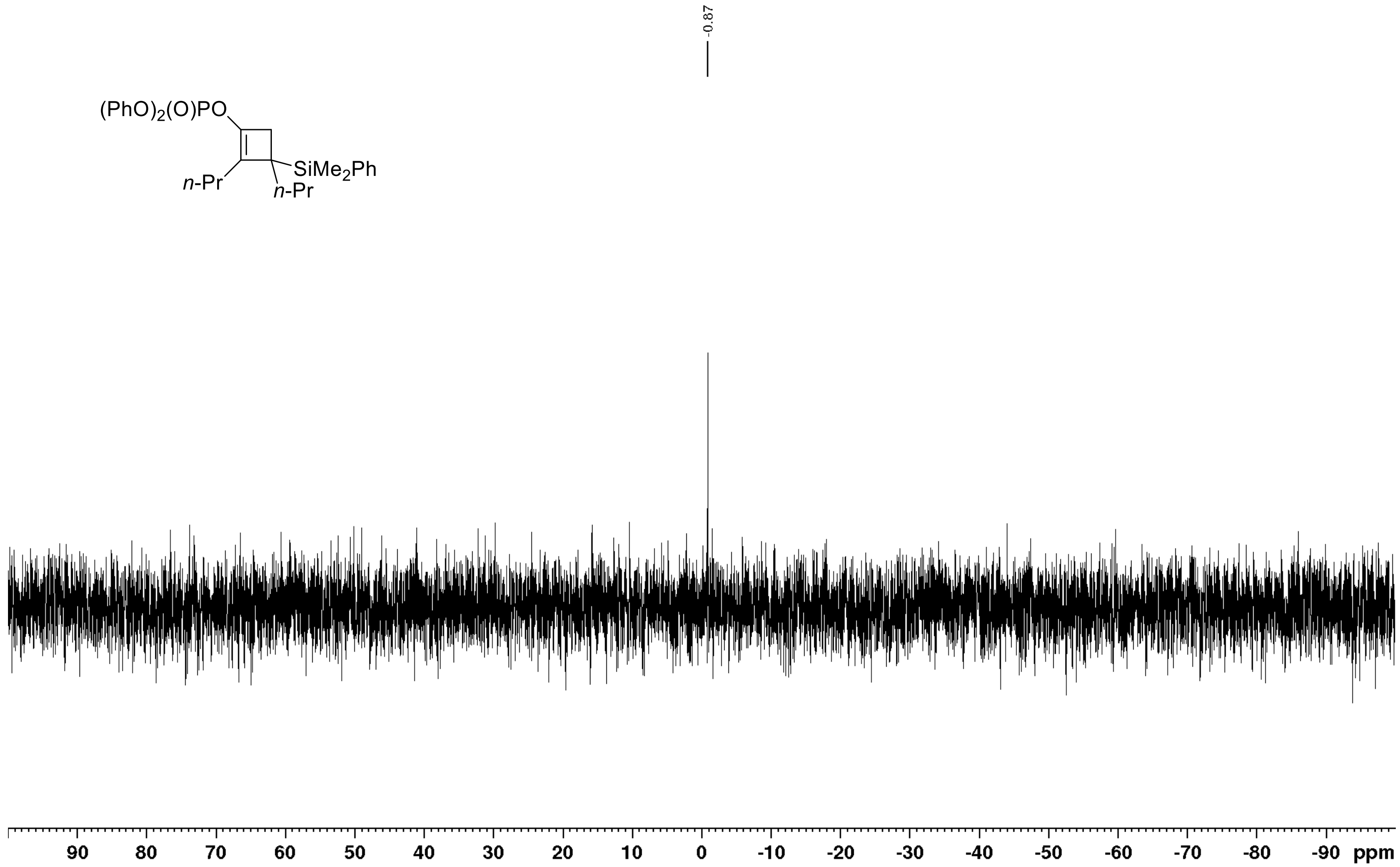

Figure S114. $^{31}P\{^1H\}$ NMR (202 MHz, $CDCl_3$, 298 K) of 3-(Dimethyl(phenyl)silyl)-2,3-dipropylcyclobut-1-en-1-yl diphenyl phosphate (**3p**)

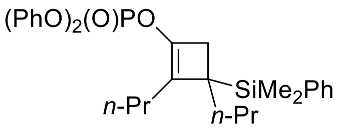

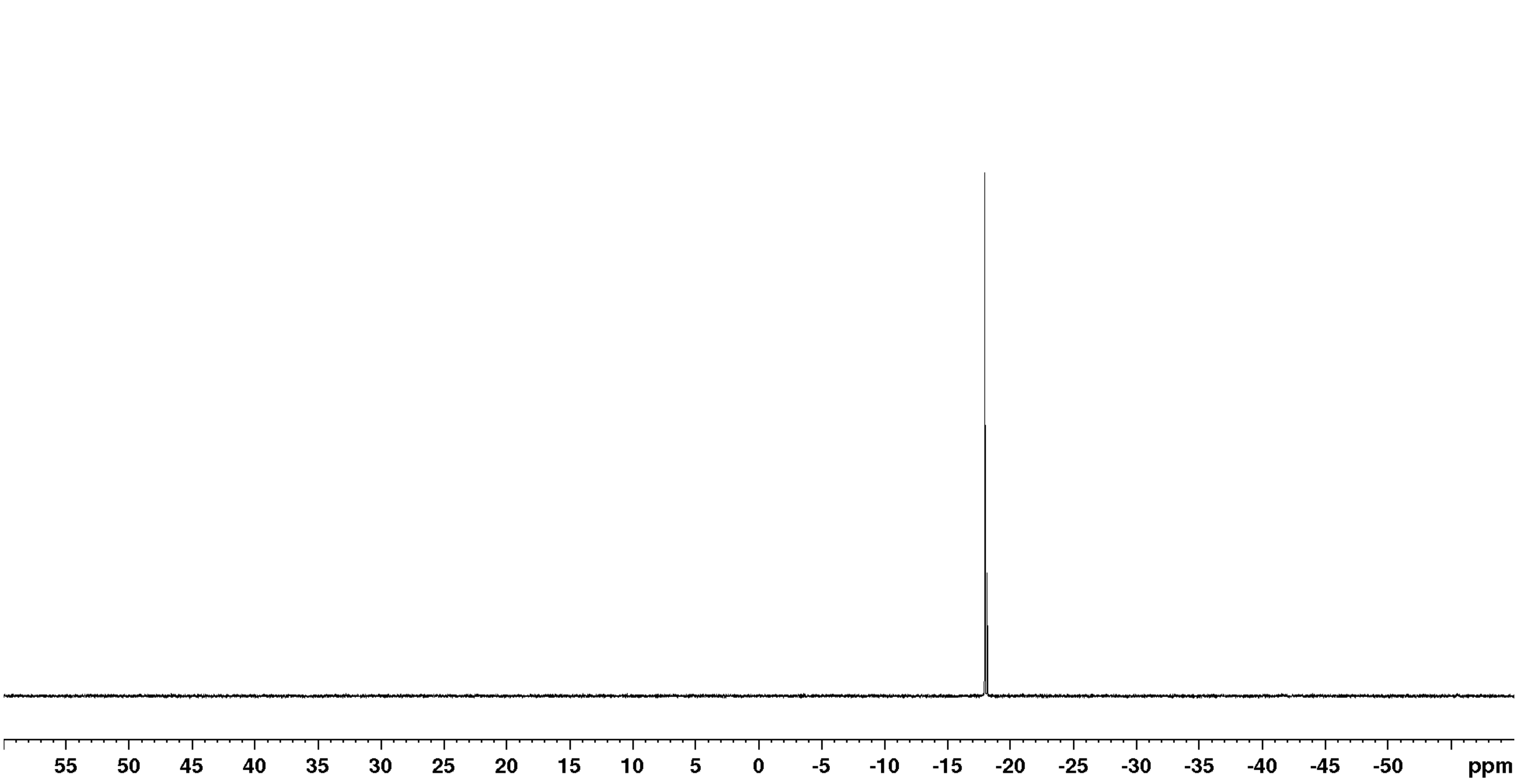

Figure S115. ^{1}H NMR (500 MHz, $CDCl_3$, 298 K) of (1,3-Diphenylcyclobut-2-en-1-yl)dimethyl(phenyl)silane (**4aa**)

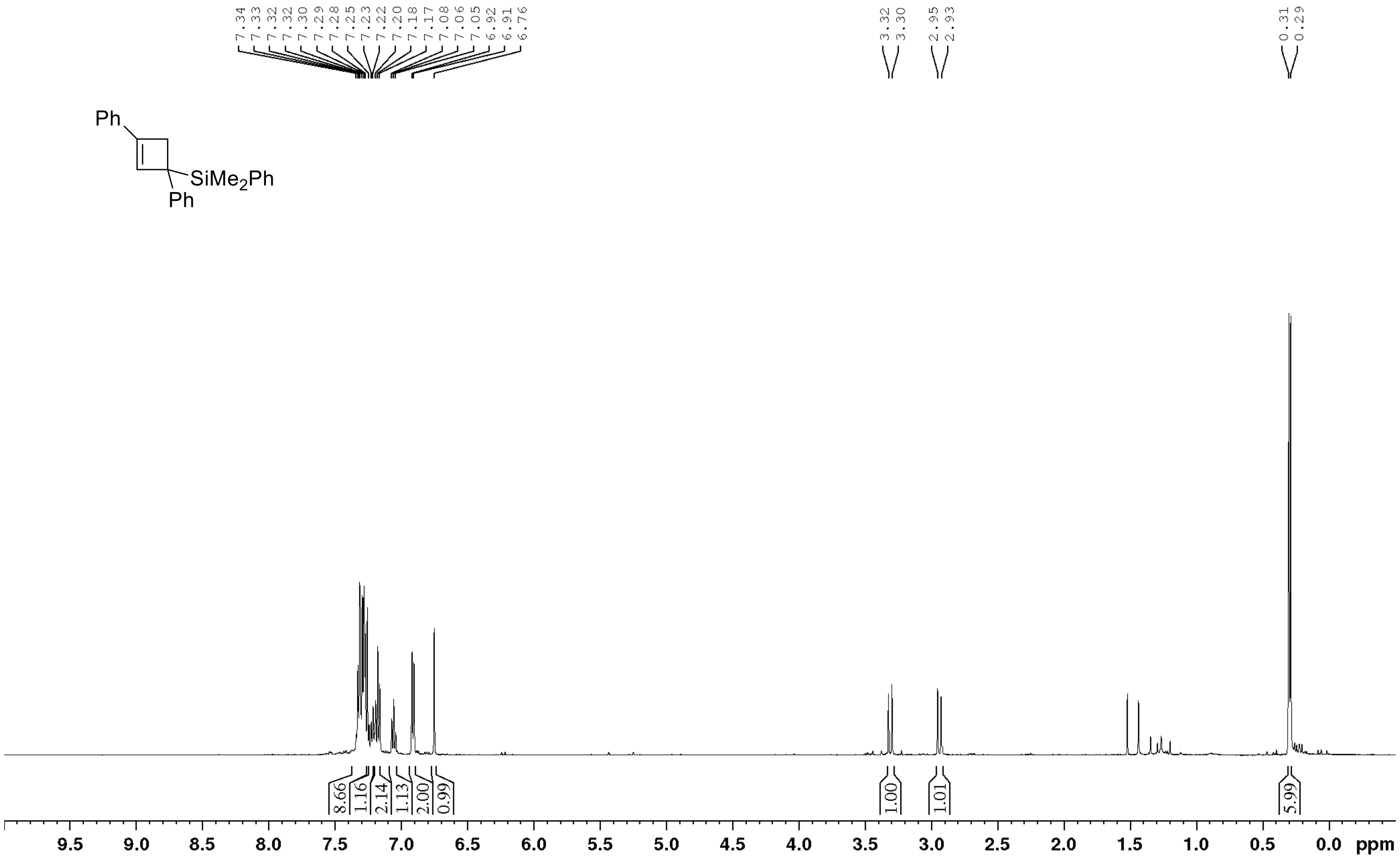

Figure S116. $^{13}C\{^{1}H\}$ NMR (126 MHz, $CDCl_3$, 298 K) of (1,3-Diphenylcyclobut-2-en-1-yl)dimethyl(phenyl)silane (**4aa**)

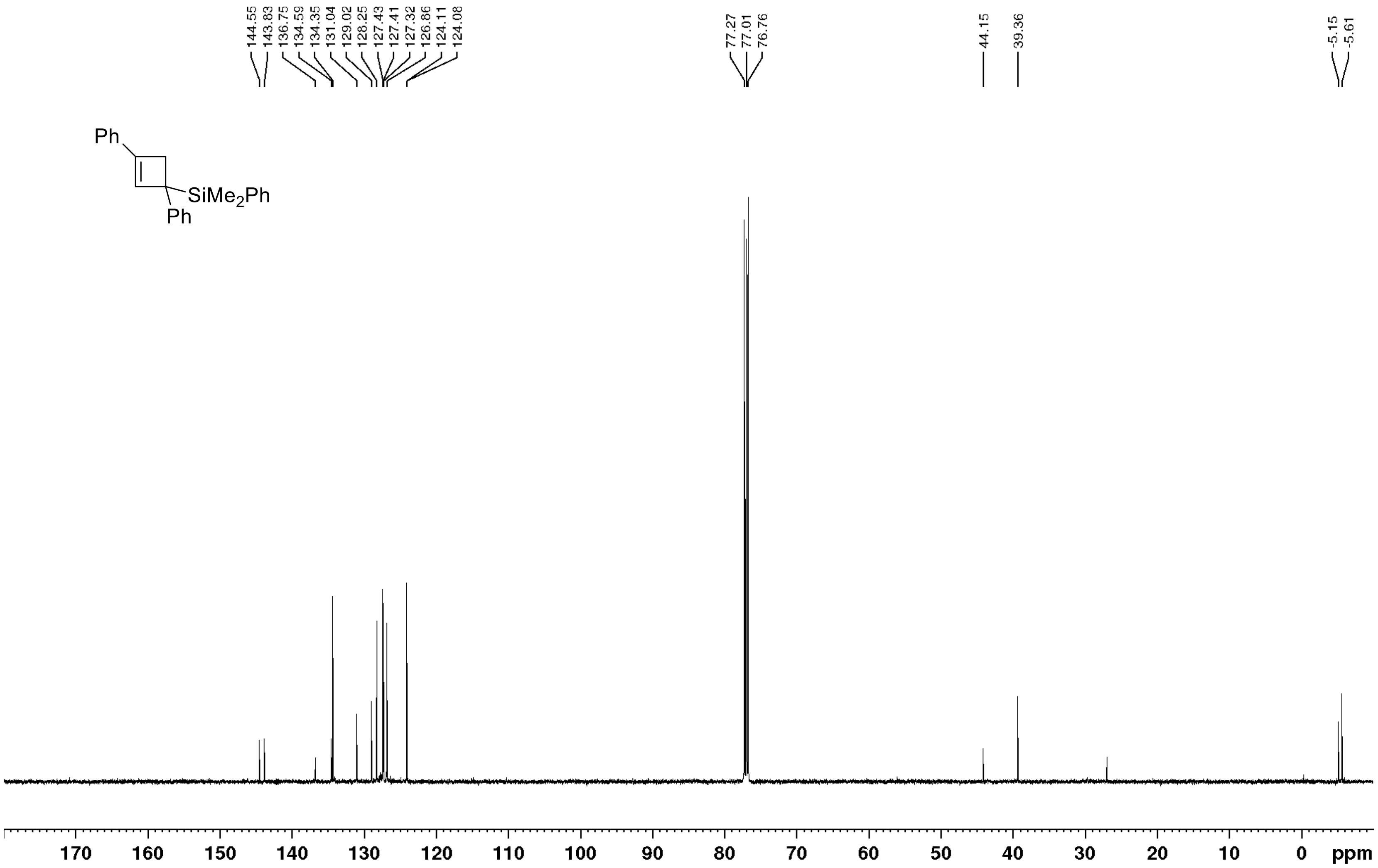

Figure S117. $^{29}Si\{^{1}H\}$ DEPT NMR (99 MHz, $CDCl_3$, 298 K) of (1,3-Diphenylcyclobut-2-en-1-yl)dimethyl(phenyl)silane (**4aa**)

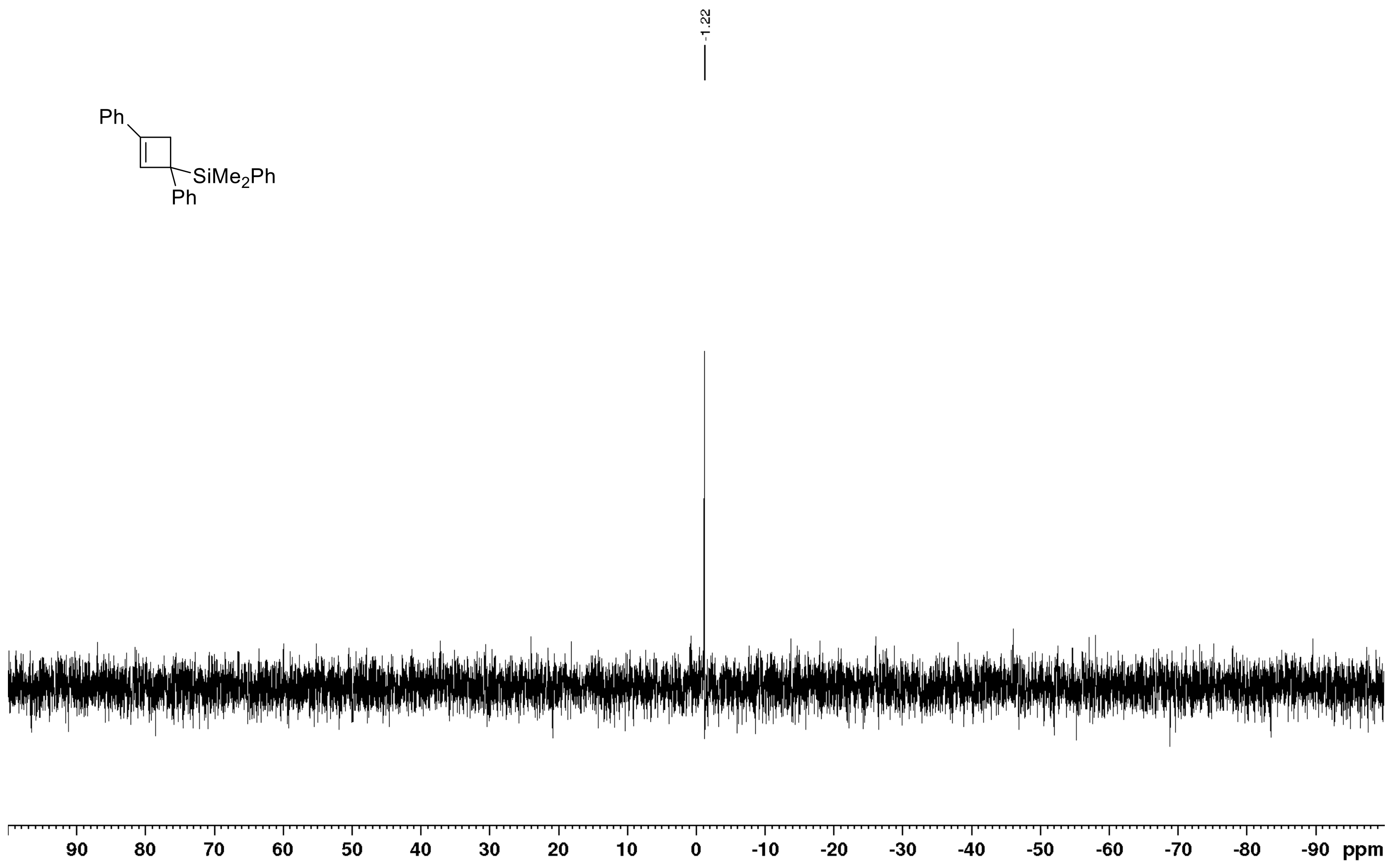

Figure S118. ^{1}H NMR (500 MHz, $CDCl_3$, 298 K) of (1-Butyl-3-phenylcyclobut-2-en-1-yl)dimethyl(phenyl)silane (**4ga**)

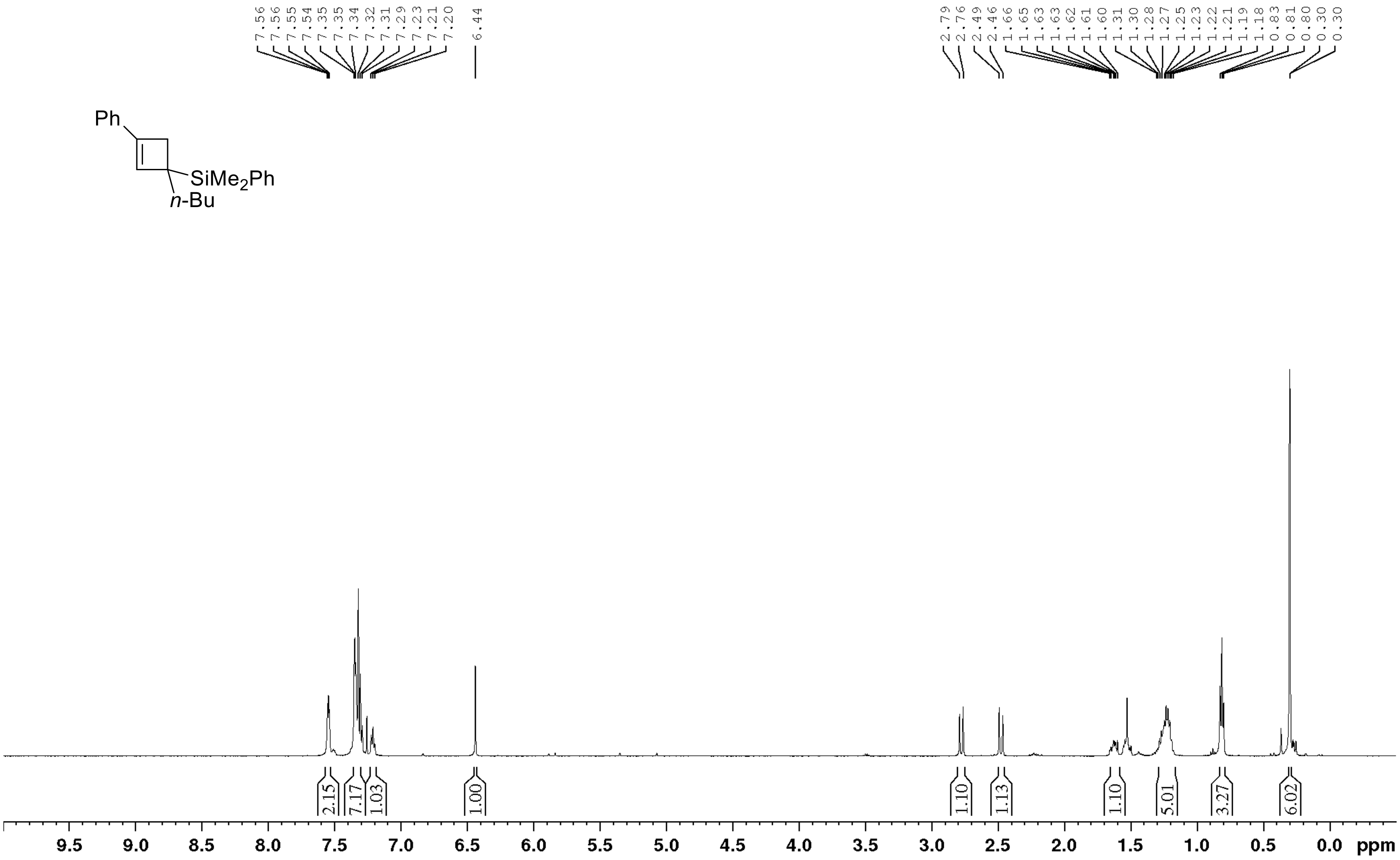

Figure S119. $^{13}C\{^{1}H\}$ NMR (126 MHz, $CDCl_3$, 298 K) of (1-Butyl-3-phenylcyclobut-2-en-1-yl)dimethyl(phenyl)silane (**4ga**)

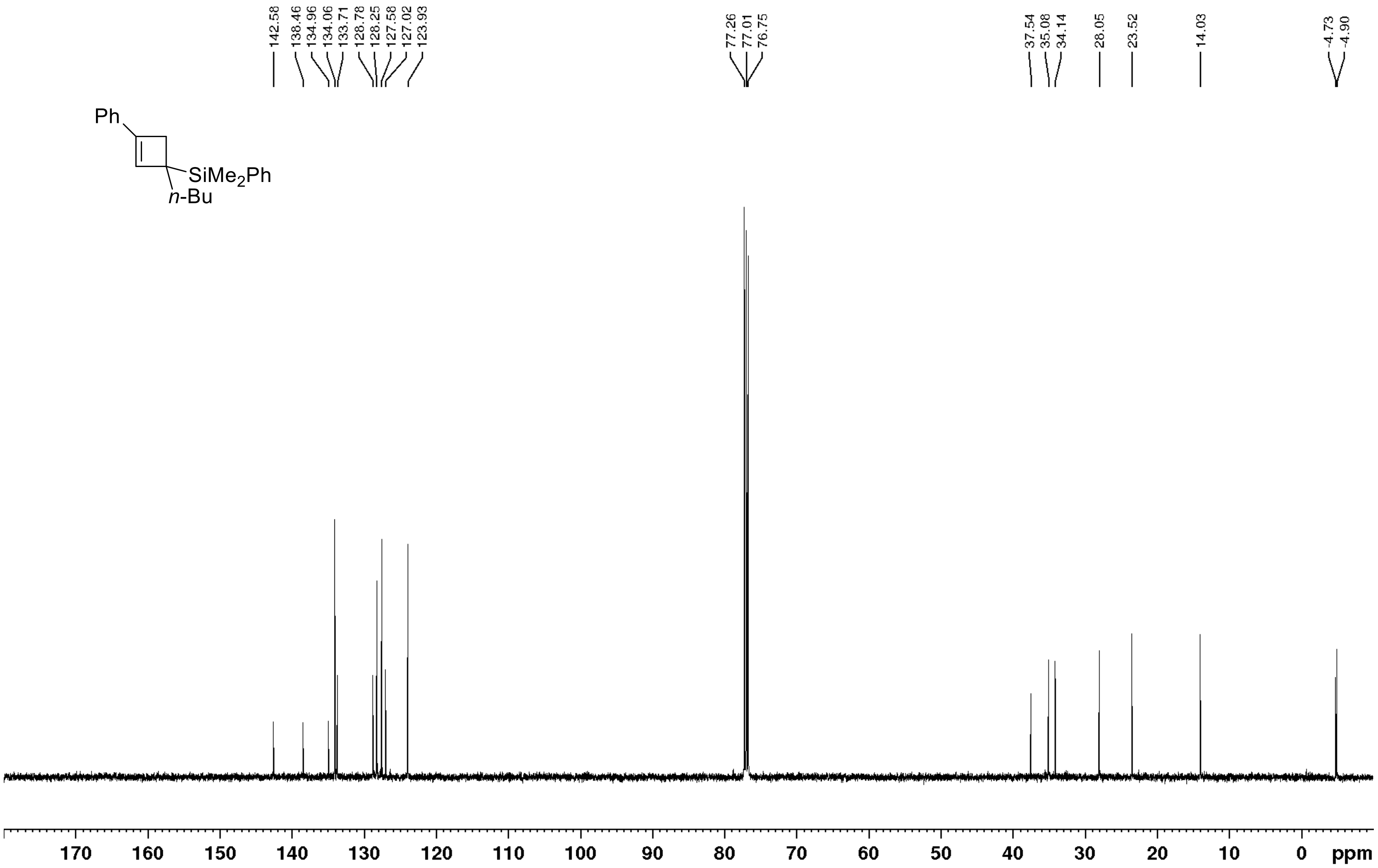

Figure S120. $^{29}Si\{^1H\}$ NMR (99 MHz, $CDCl_3$, 298 K) of (1-Butyl-3-phenylcyclobut-2-en-1-yl)dimethyl(phenyl)silane (**4ga**)

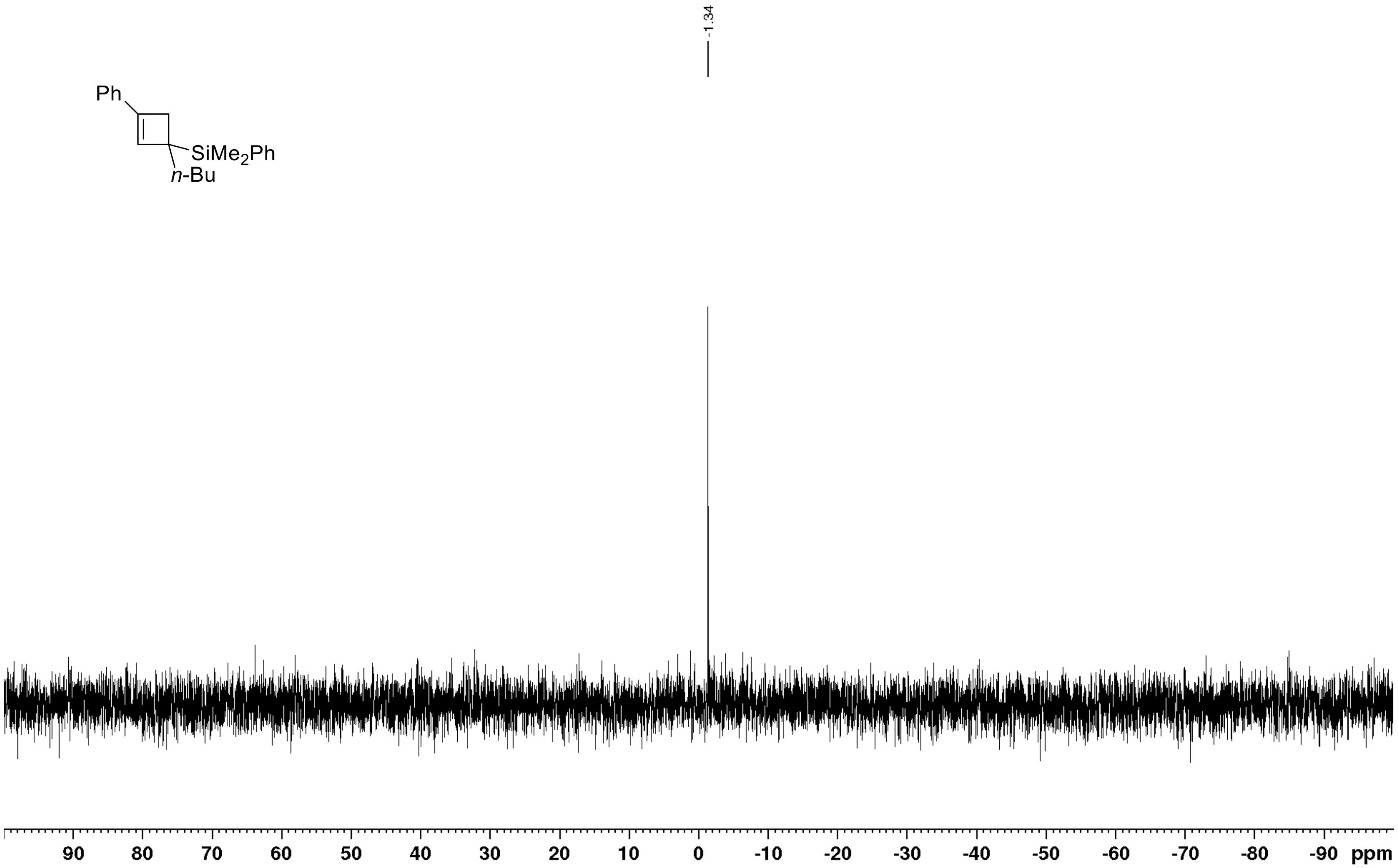

Figure S121. ^{1}H NMR (500 MHz, $CDCl_3$, 298 K) of (1,3-Diphenylcyclobut-2-en-1-yl)dimethyl(phenyl)silane (**4ja**)

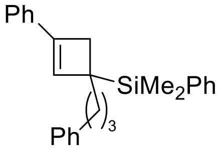

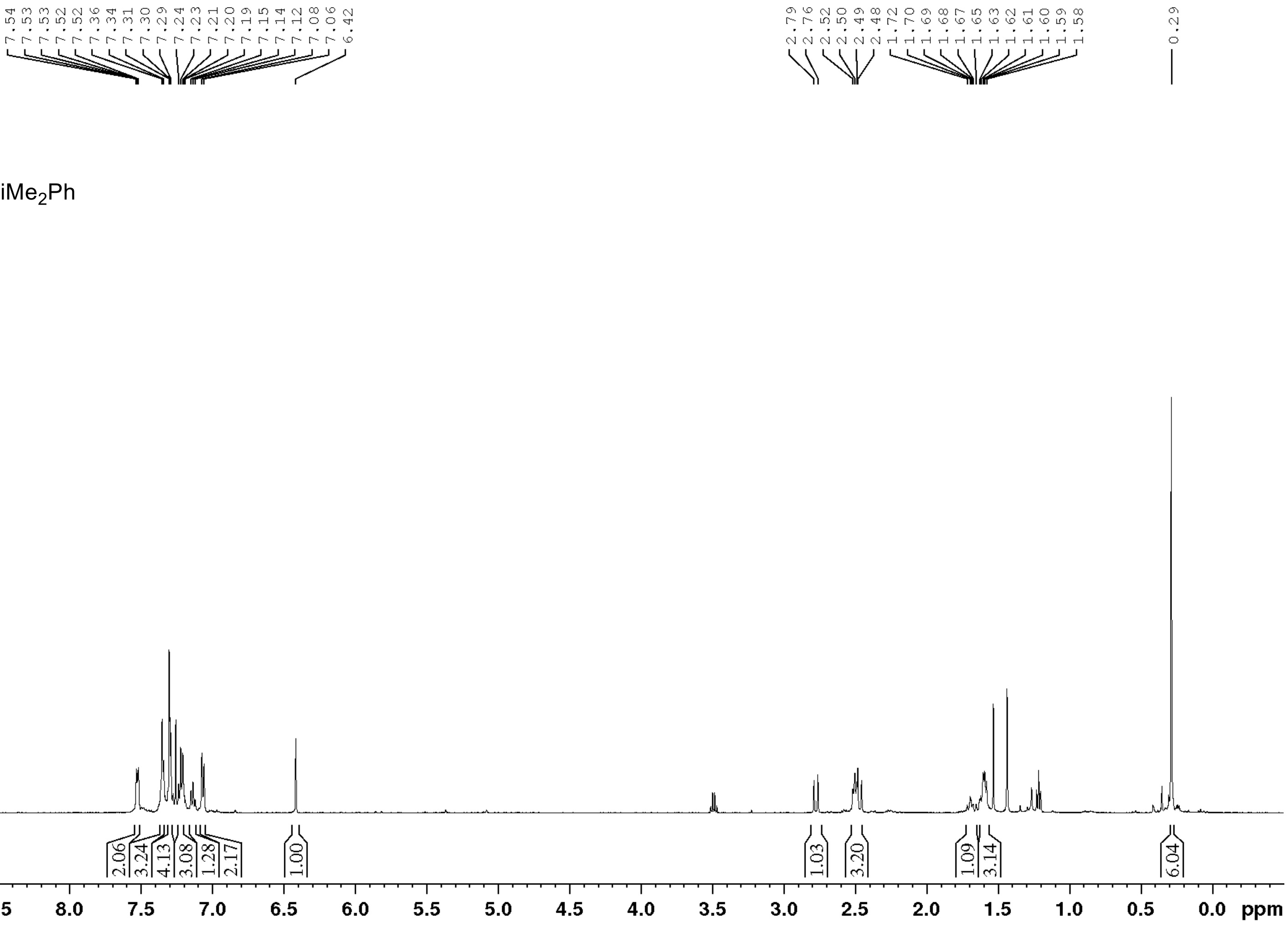

Figure S122. $^{13}C\{^{1}H\}$ NMR (126 MHz, $CDCl_3$, 298 K) of (1,3-Diphenylcyclobut-2-en-1-yl)dimethyl(phenyl)silane (**4ja**)

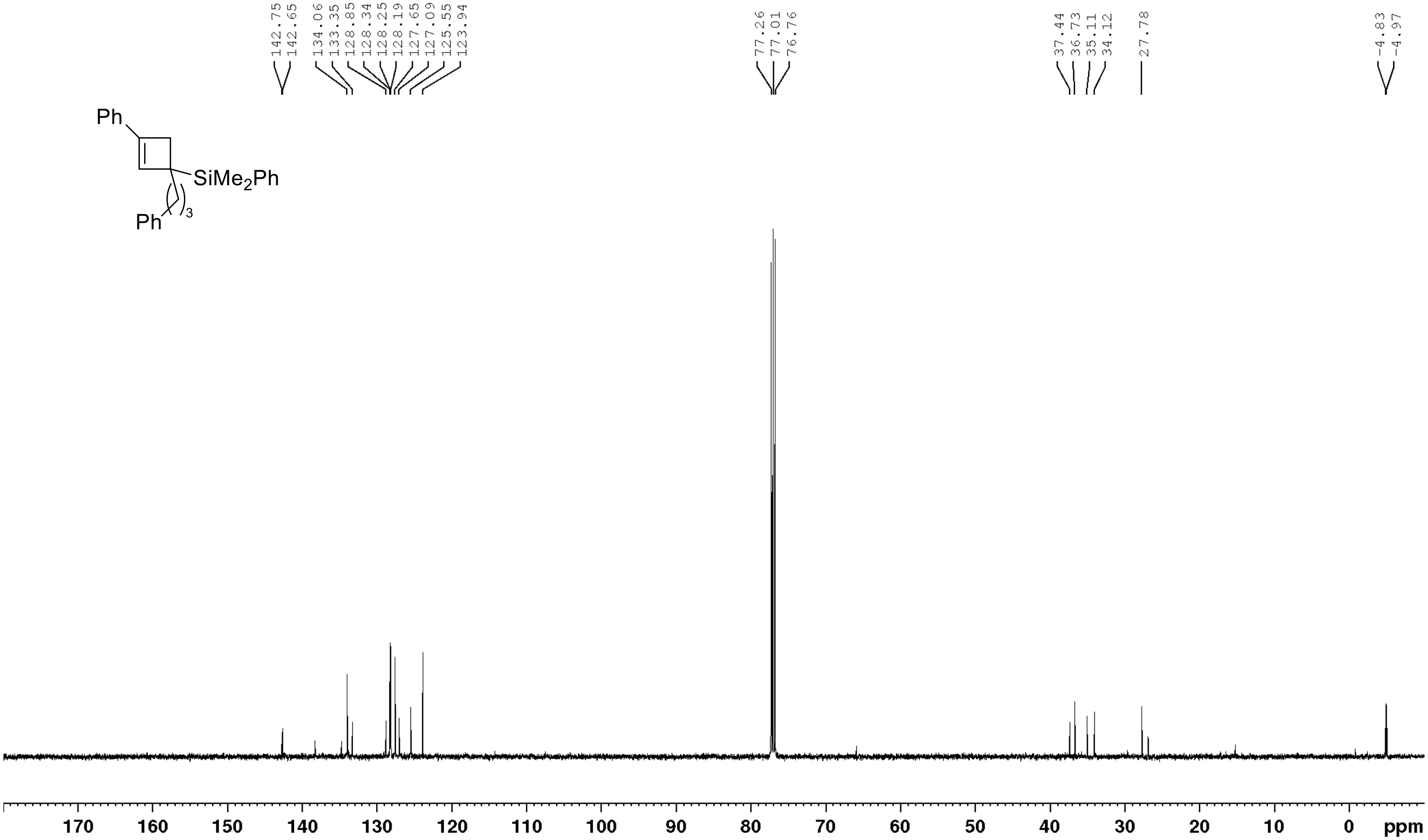

Figure S123. $^{29}Si\{^{1}H\}$ DEPT NMR (99 MHz, $CDCl_3$, 298 K) of (1,3-Diphenylcyclobut-2-en-1-yl)dimethyl(phenyl)silane (**4ja**)

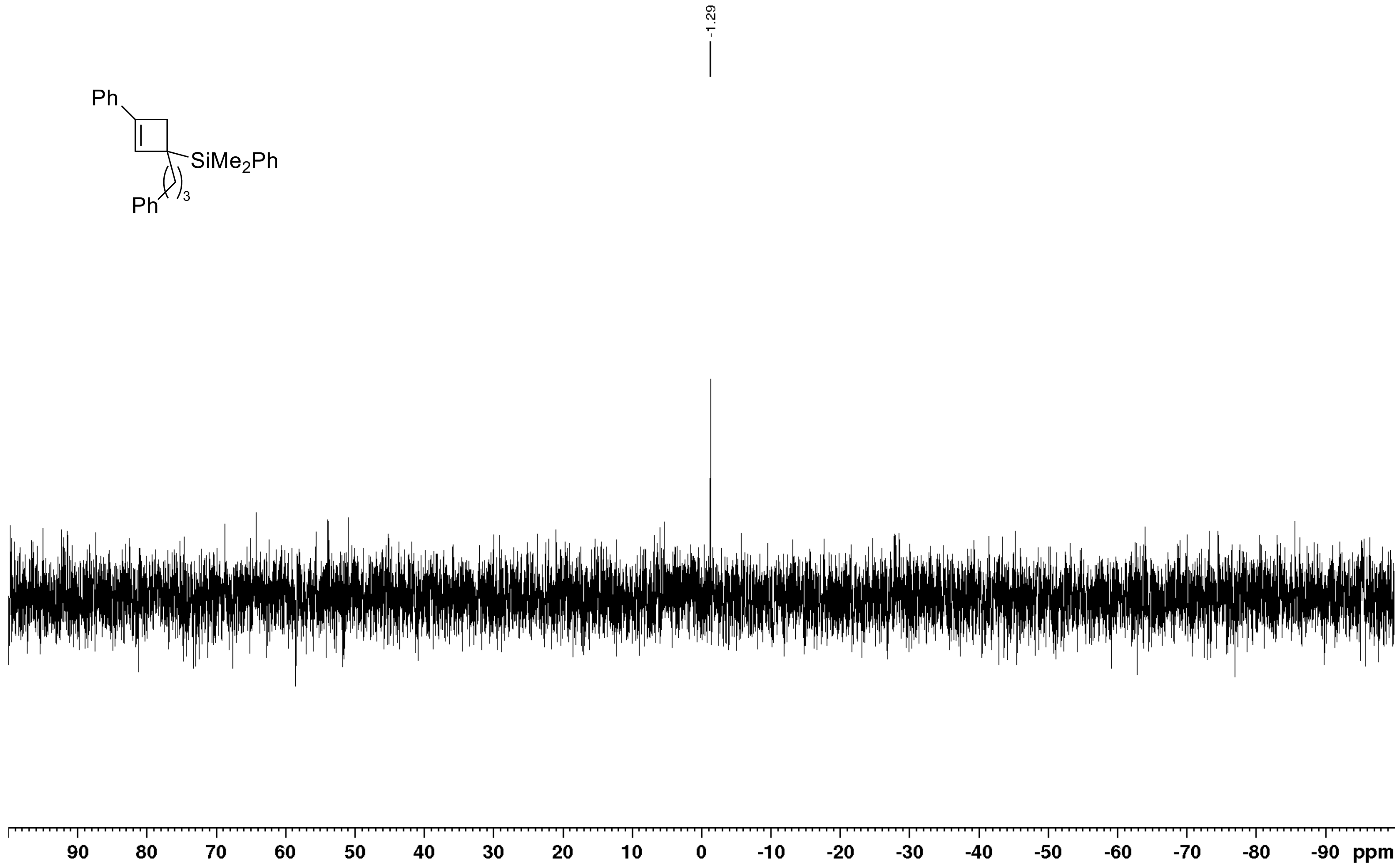

Figure S124. ^{1}H NMR (500 MHz, $CDCl_3$, 298 K) of (1-(4-Chlorobutyl)-3-phenylcyclobut-2-en-1-yl)dimethyl(phenyl)silane (**4ka**)

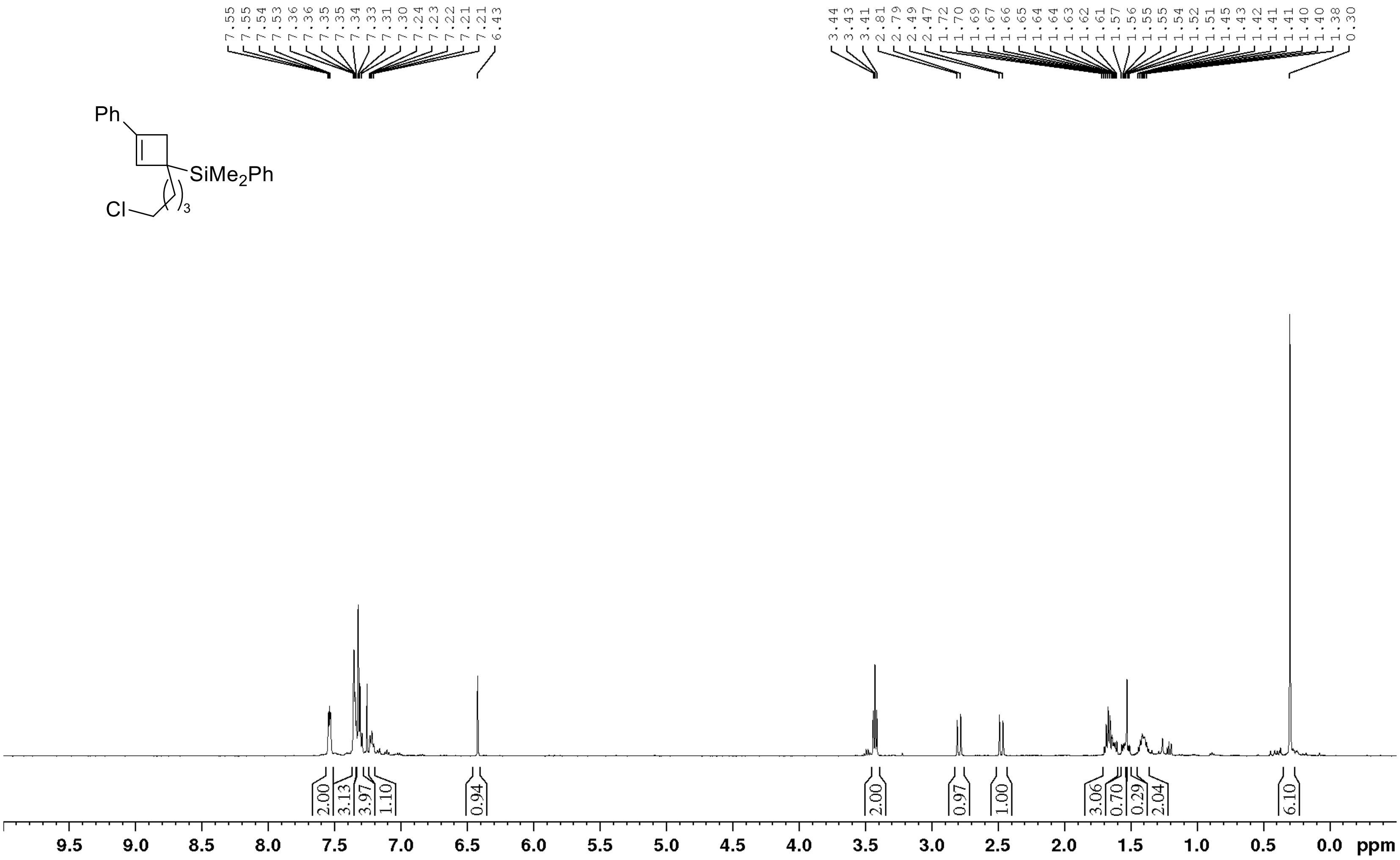

Figure S125. $^{13}C\{^{1}H\}$ NMR (126 MHz, $CDCl_3$, 298 K) of (1-(4-Chlorobutyl)-3-phenylcyclobut-2-en-1-yl)dimethyl(phenyl)silane (**4ka**)

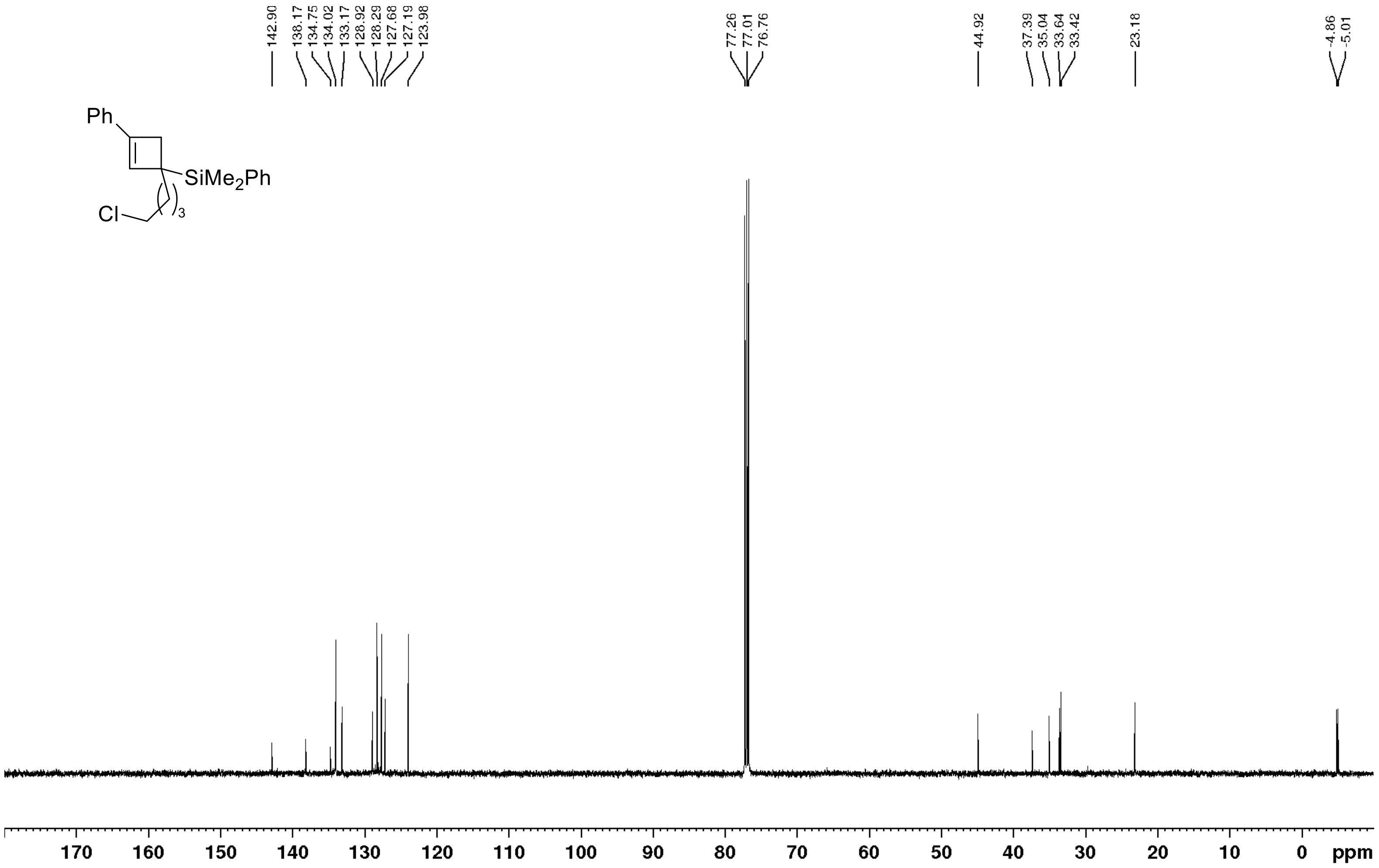

Figure S126. $^{29}Si\{^{1}H\}$ NMR (99 MHz, $CDCl_3$, 298 K) of (1-(4-Chlorobutyl)-3-phenylcyclobut-2-en-1-yl)dimethyl(phenyl)silane (**4ka**)

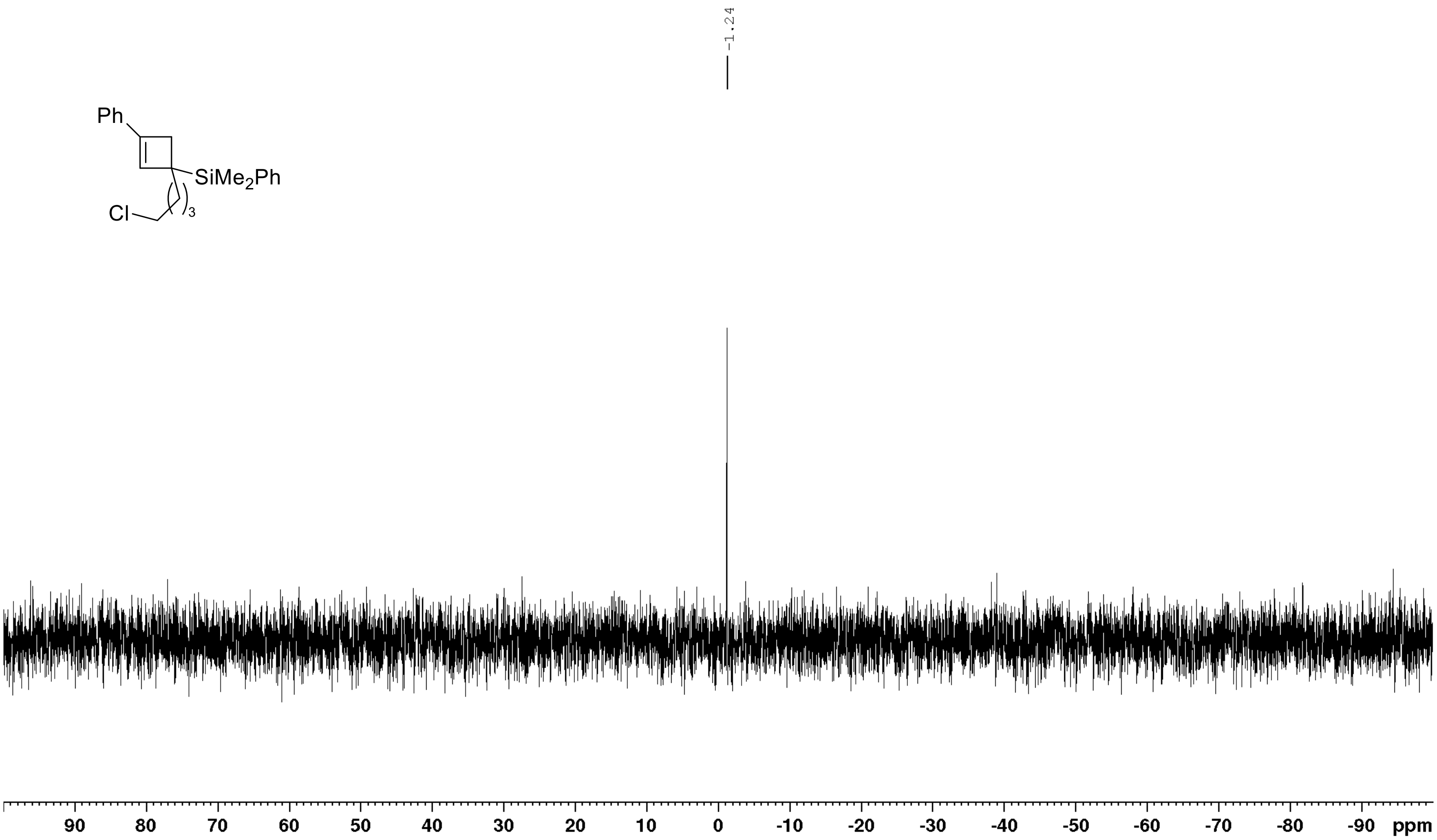

Figure S127. ^{1}H NMR (500 MHz, $CDCl_3$, 298 K) of (3-Hexyl-1-phenylcyclobut-2-en-1-yl)dimethyl(phenyl)silane (**4ab**)

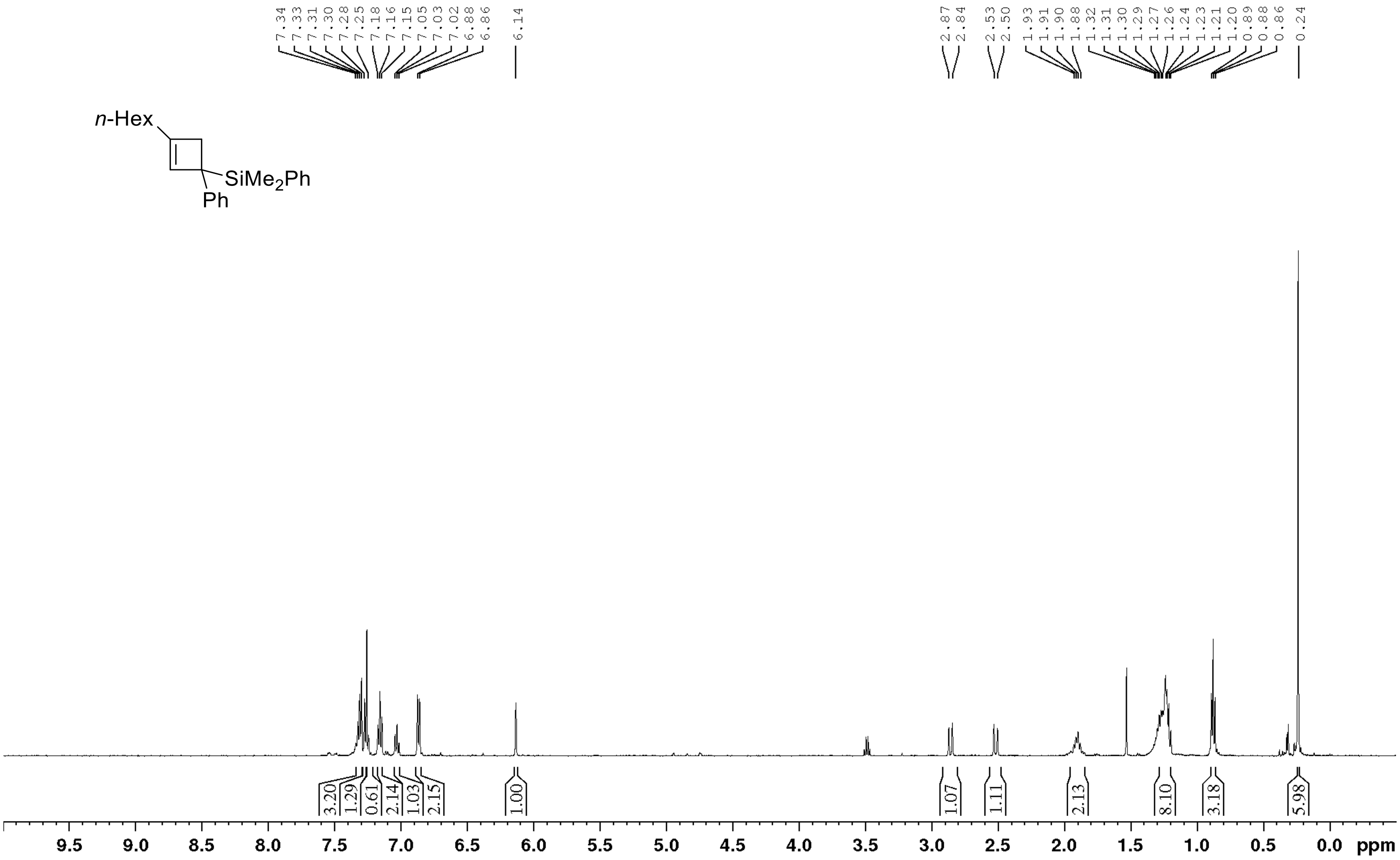

Figure S128. $^{13}C\{^{1}H\}$ NMR (126 MHz, $CDCl_3$, 298 K) of (3-Hexyl-1-phenylcyclobut-2-en-1-yl)dimethyl(phenyl)silane (**4ab**)

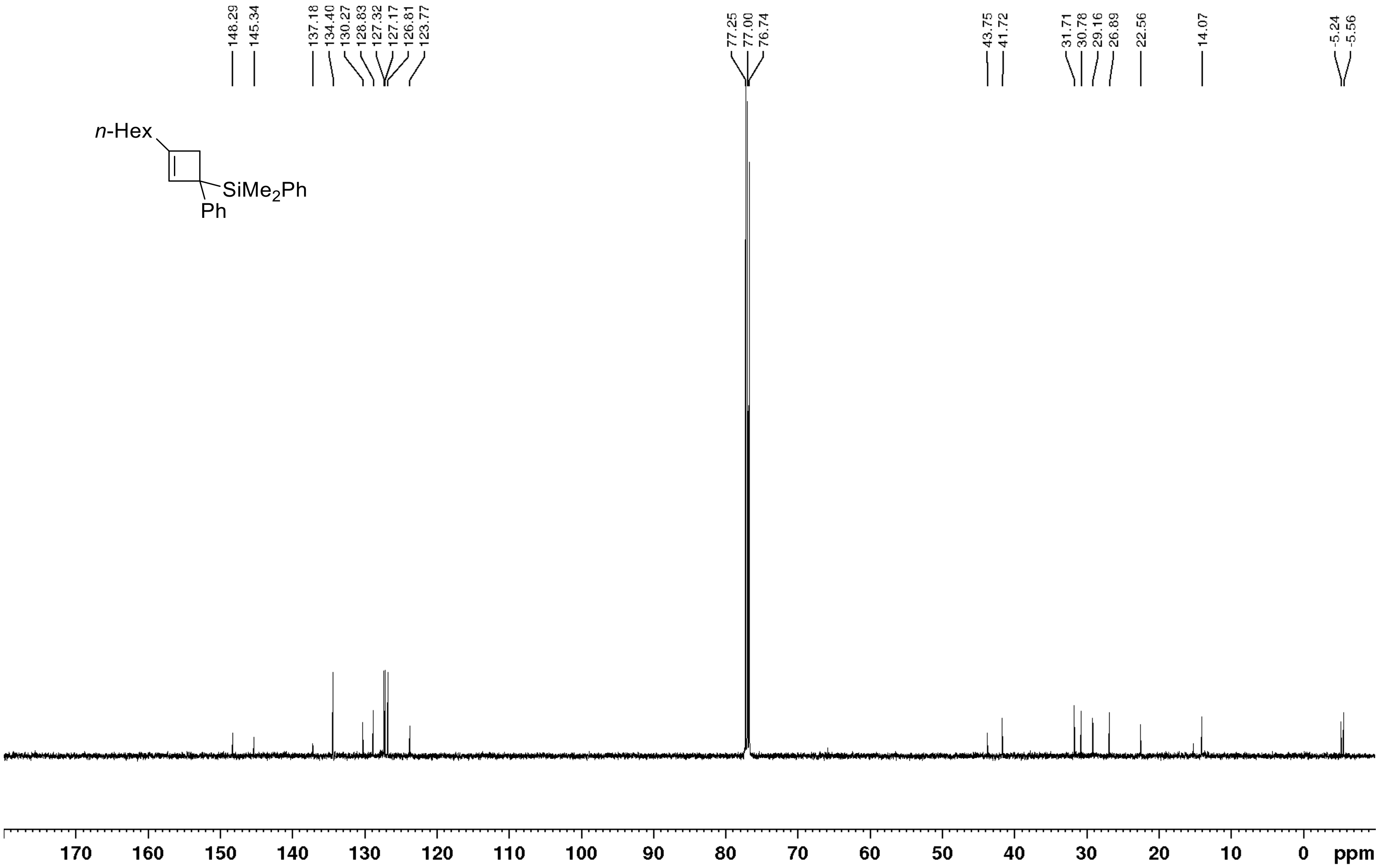

Figure S129. $^{29}Si\{^1H\}$ DEPT NMR (99 MHz, $CDCl_3$, 298 K) of (3-Hexyl-1-phenylcyclobut-2-en-1-yl)dimethyl(phenyl)silane (**4ab**)

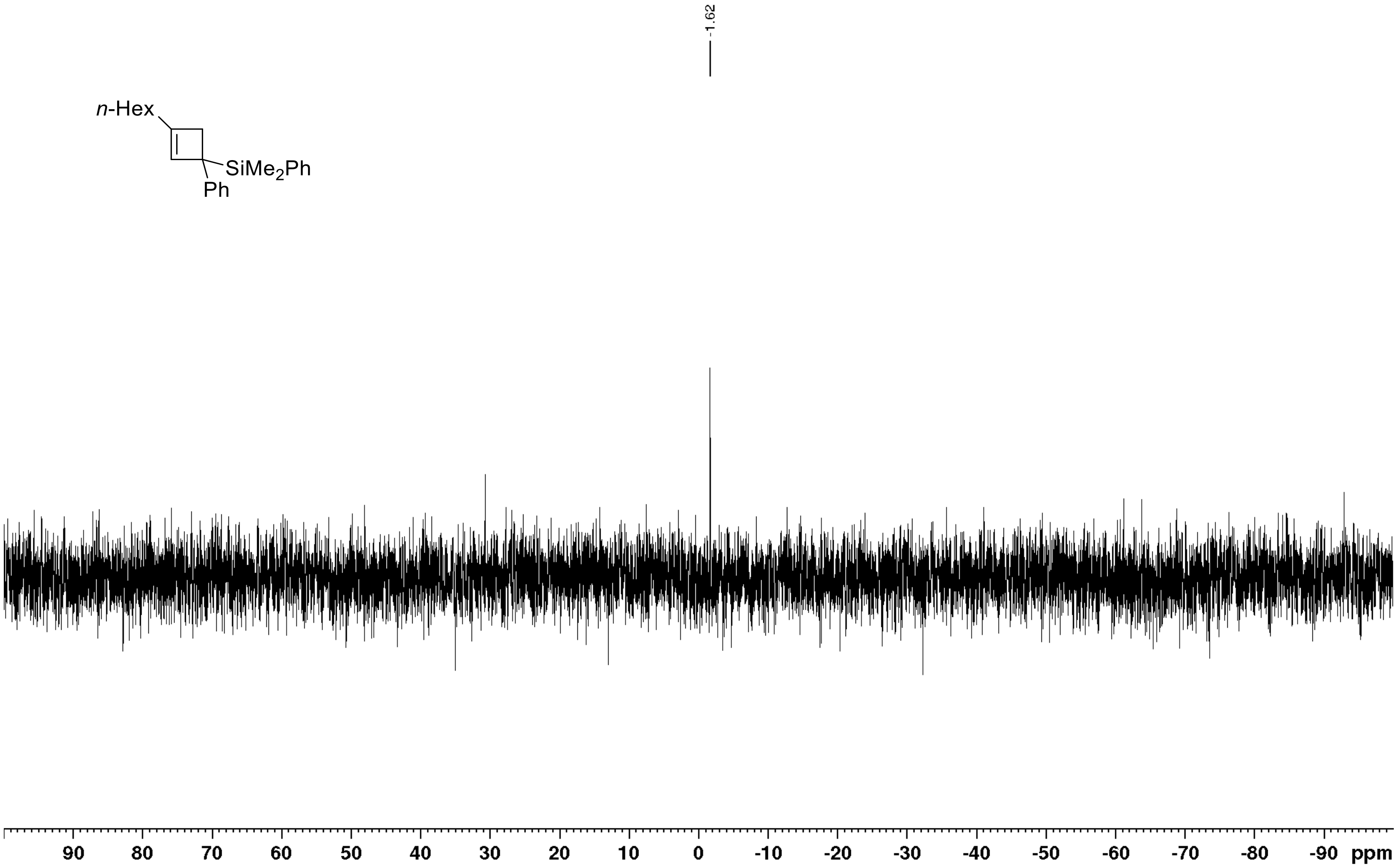

Figure S130. ^{1}H NMR (500 MHz, $CDCl_3$, 298 K) of (3-Cyclohexyl-1-phenylcyclobut-2-en-1-yl)dimethyl(phenyl)silane (**4ac**)

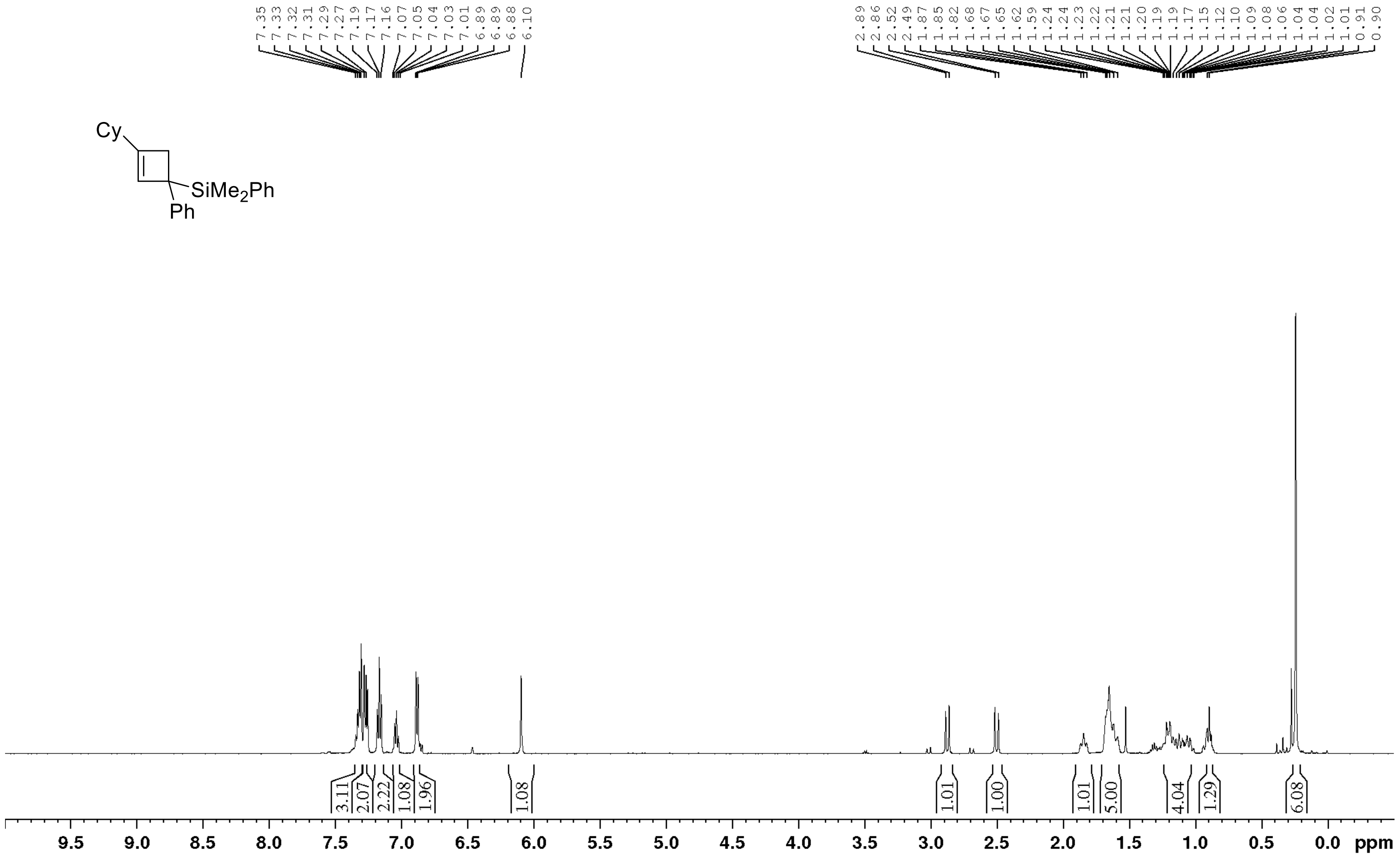

Figure S131. $^{13}C\{^1H\}$ NMR (126 MHz, $CDCl_3$, 298 K) of (3-Cyclohexyl-1-phenylcyclobut-2-en-1-yl)dimethyl(phenyl)silane (**4ac**)

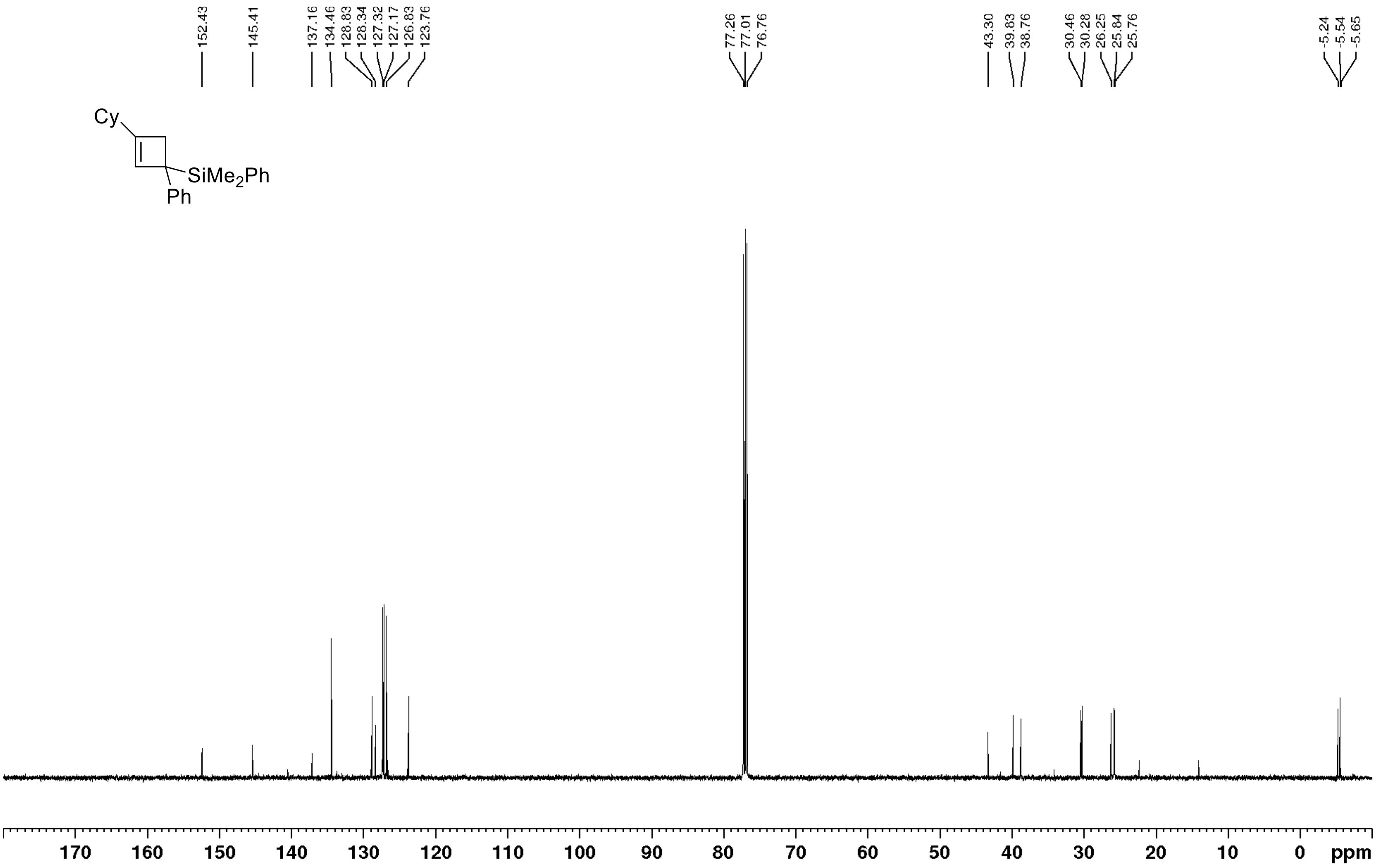

Figure S132. $^{29}Si\{^1H\}$ DEPT NMR (99 MHz, $CDCl_3$, 298 K) of (3-Cyclohexyl-1-phenylcyclobut-2-en-1-yl)dimethyl(phenyl)silane (**4ac**)

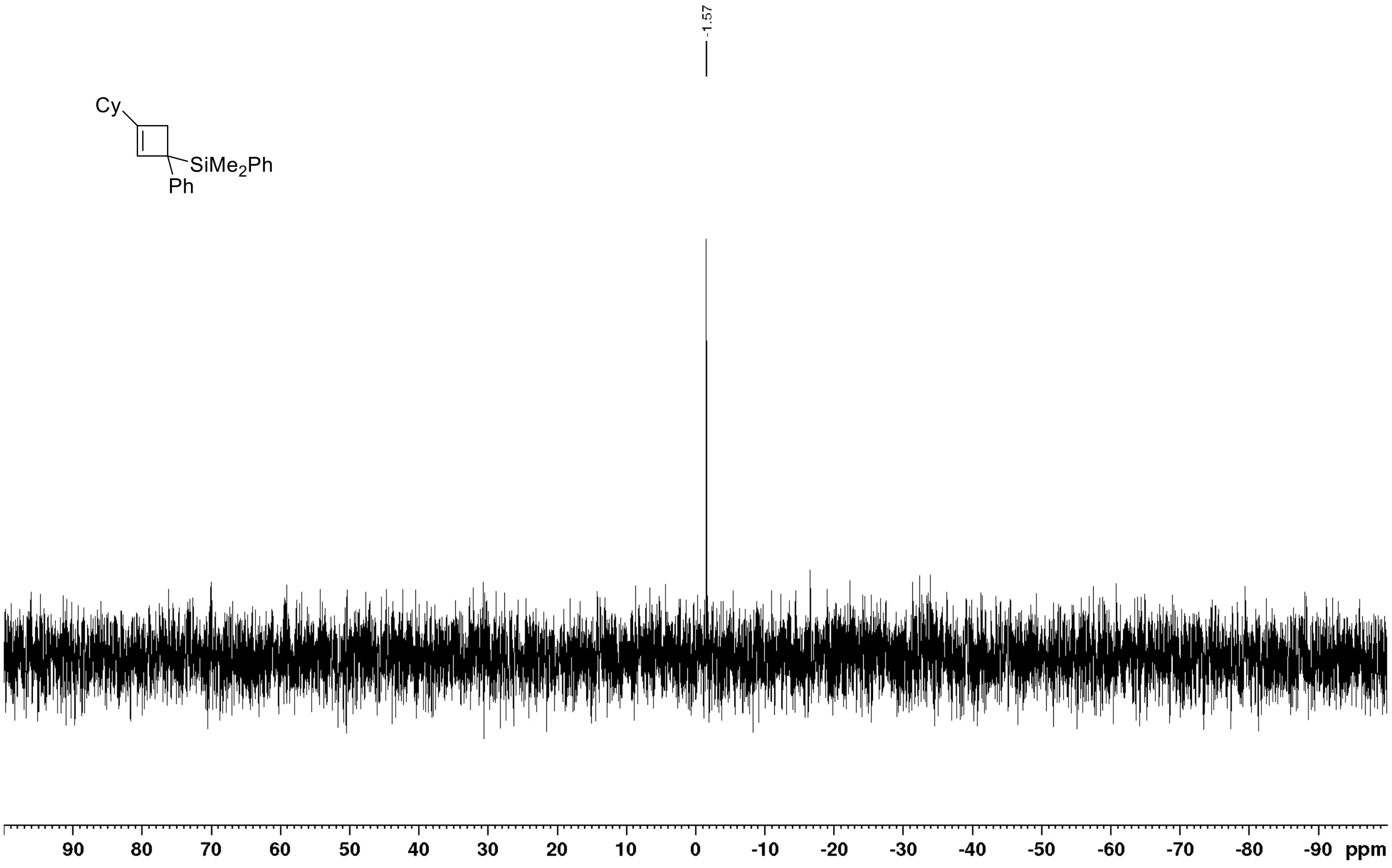

12. References

[S1] S. Rösel, J. Becker, W. D. Allen, P. R. Schreiner, *J. Am. Chem. Soc.* **2018**, *140*, 14421–14432.

[S2] L. Zhang, M. Oestreich, *Org. Lett.* **2018**, *20*, 8061–8063.

[S3] W. G. Kofron, L. M. Baclawski. *J. Org. Chem.* **1976**, *41*, 1879–1880.

[S4] A. Krasovskiy, P. Knochel, *Synthesis* **2006**, 890–891.

[S5] C. Fopp, E. Romain, K. Isaac, F. Chemla, F. Ferreira, O. Jackowski, M. Oestreich, A. Perez-Luna, *Org. Lett.* **2016**, *18*, 2054–2057.

[S6] C. Zhong, Y. Huang, H. Zhang, Q. Zhou, Y. Liu, P. Lu, *Angew. Chem. Int. Ed.* **2020**, *59*, 2750–2754; *Angew. Chem.* **2020**, *132*, 2772–2776.

[S7] For the preparation of substrates **1c**, **1n**, **1o**, and **1p**, see: a) substrate **1c**: H. Zhang, Y. Luo, D. Li, Q. Yao, S. Dong, X. Liu, X. Feng, *Org. Lett.* **2019**, *21*, 2388–2392; b) substrate **1n**: G. B. Stone, L. S. Liebeskind, *J. Org. Chem.* **1990**, *55*, 4614–4622; c) substrate **1o**: H. A. Clement, M. Boghi, R. M. McDonald, L. Bernier, J. W. Coe, W. Farrell, C. J. Helal, M. R. Reese, N. W. Sach, J. C. Lee, D. G. Hall, *Angew. Chem. Int. Ed.* **2019**, *58*, 18405–18409; *Angew. Chem.* **2019**, *51*, 18576–18570. d) substrate **1p**: R. L. Danheiser, S. Savariar, *Tetrahedron Lett.* **1987**, *28*, 3299–3302.
